# Development of 2-Aminoadenine-Based Proteolysis-Targeting Chimeras (PROTACs) as Novel Potent Degraders of Monopolar Spindle 1 and Aurora Kinases

**DOI:** 10.1021/acsptsci.4c00405

**Published:** 2024-10-19

**Authors:** Eleni Sflakidou, Bikash Adhikari, Christos Siokatas, Elmar Wolf, Vasiliki Sarli

**Affiliations:** † Department of Chemistry, Aristotle University of Thessaloniki, University Campus, 54124 Thessaloniki, Greece; ‡ Cancer Systems Biology Group, Chair of Biochemistry and Molecular Biology, Theodor Boveri Institute, University of Würzburg, 97074 Würzburg, Germany; § Institute of Biochemistry, University of Kiel, 24118 Kiel, Germany

**Keywords:** PROTACs, TTK, AURKA, AURKB, 2-aminoadenine, targeted protein degradation

## Abstract

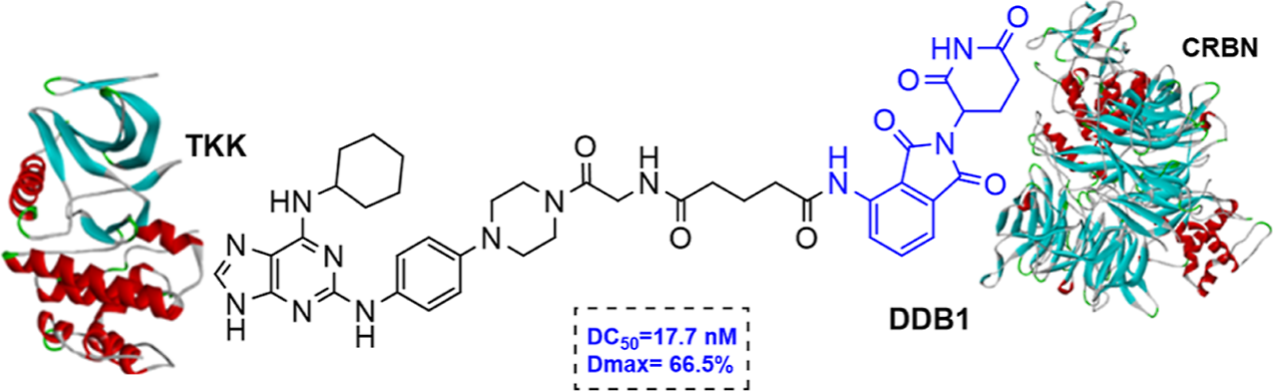

Monopolar spindle 1 (Mps1, also known as TTK) and Aurora kinase (AURK) A and B are critical regulators of mitosis and have been linked to the progression of various cancers. Here, we report the design, synthesis, and biological evaluation of a series of PROTACs (proteolysis-targeting chimeras) targeting TTK and AURKs. We synthesized various degrader molecules based on four different 2-aminoadenine-based ligands, recruiting either cereblon or VHL as the E3-ligase. Our research showed that the nature of the linker and modification of the ligand significantly influence the target specificity and degradation efficacy. Notably, compound **19**, among the most potent degraders, demonstrated robust proteasome-mediated degradation of TTK with *D*_max_ of 66.5% and DC_50_ value (6 h) of 17.7 nM as compared to its structurally akin inhibitor control, **23**. The cytotoxicity of most of the synthesized chimeras against acute myeloid leukemia cell line MV4-11 was lower than that of the corresponding parent inhibitors. However, we could also identify degraders such as **15** and **26** that induce potent AURKA degradation and display comparable antiproliferative activities to their parent compound **SF1**. Compound **15** degrades AURKA with low DC_50_ value of 2.05 nM, which is 77-fold and 21-fold more selective toward AURKB and TTK and has an EC_50_ value of 39 nM against cancer MV4-11 cells. Overall, the observations we made with the degrader molecules we developed can further aid in the design and development of optimized TTK or AURK degraders for cancer therapy.

Protein kinases play essential roles in mitosis, controlling mitotic progression and proper chromosome segregation. The organization and function of mitotic kinases during these dynamic processes occur by diverse mechanisms including protein–protein interactions and post-translational modifications, while their dysregulation often results in aneuploidy, genomic instability, and tumorigenesis.^1^ The most well-characterized mitotic kinases are Auroras, Cdks, Plks, Neks, Bubs, Haspin, and Mps1/TTK.^2^ The Aurora family of serine/threonine protein kinases (Aurora kinases (AURKs)) consists of three members AURKs A, B, and C. While AURKC is mainly active in meiotic cells, AURKA and AURKB play a pivotal role in mitotic progression and cell division.^3,4^ AURKA regulates mitotic events by phosphorylating multiple proteins, including Cyclin-B/Cdk-1, Eg5, and Polo-like kinase-1.^5^ AURKB is part of the chromosomal passenger complex and is important for chromosome condensation, segregation, and implementation of cytokinesis. AURKB is considered an important anticancer drug target since it is overexpressed in several human malignancies.^6^ Inhibition of AURKB is known to result in potent antiproliferative effects in vitro and in vivo. In addition, the activity of AURKs has been correlated with resistance to cancer chemotherapy, and AURKs are considered effective predictors of chemotherapy response and prognosis.^7^ It is believed that AURKB mediates Myc oncogenic effects in cancers and acts as a c-Myc transcription promoter, while MYC directly binds to AURKA.^8^

Monopolar spindle 1 (Mps1), also known as TTK, is a serine–threonine kinase that activates the spindle assembly checkpoint to ensure the proper distribution of chromosomes to daughter cells.^9,10^ Other significant functions of TTK include spindle pole duplication, the mitotic exit and cytokinesis, altogether underlying TTK’s prominent role in the regulation of cellular proliferation and division.^11^ Several studies have shown that inhibiting TTK has a direct impact on the survival of cancer cells. Even though TTK is hardly expressed in normal tissues and organs, its elevated levels have been detected in various malignancies and aggressive types of cancer, such as breast cancer,^12^ glioblastoma,^13^ and lung cancer.^14^ Based on these facts and considering TTK’s central role in cell propagation, it is not surprising that a rise in the development of anticancer agents targeting the process of mitosis and specifically TTK kinase has been observed.^15^

To date, there are several TTK and Aurora inhibitors (e.g., BAY-1161909, BAY1217389, AT9283, and Alisertib, CFI-402257) that have entered phase I/II clinical trials as antitumor agents.^16−19^ TTK inhibitors as well as Aurora inhibitors are mostly ATP-competitive, and their chemical structures include a variety of five- and six-membered heterocyclic scaffolds.^20^ Recently, a few promising AURKA and TTK targeting PROTACs (proteolysis targeting chimeras) have been reported, offering new opportunities since the PROTAC technology has opened a whole new field in drug discovery and has great potential to overcome acquired resistance to anticancer drugs.^21^ PROTACs exhibit a different mechanism of action compared to classical inhibitors as they can bind and degrade a target protein via a catalytic-type mechanism. JB170 is a potent and highly specific AURKA degrader (DC_50_ = 28 nM) that resulted from the conjugation of alisertib to thalidomide via a PEG linker (Figure 1).^22^ Another AURKA degrader is JB300 which is based on MK-5108 inhibitor and thalidomide (DC_50_ = 30 nM),^23^ while PROTAC LJB-1113157 is a TTK degrader based on a pyrido[2,3-*d*]pyrimidin-7(8*H*)-one inhibitor and lenalidomide (DC_50_ = 3.1 nM).^24^

**Figure 1:**
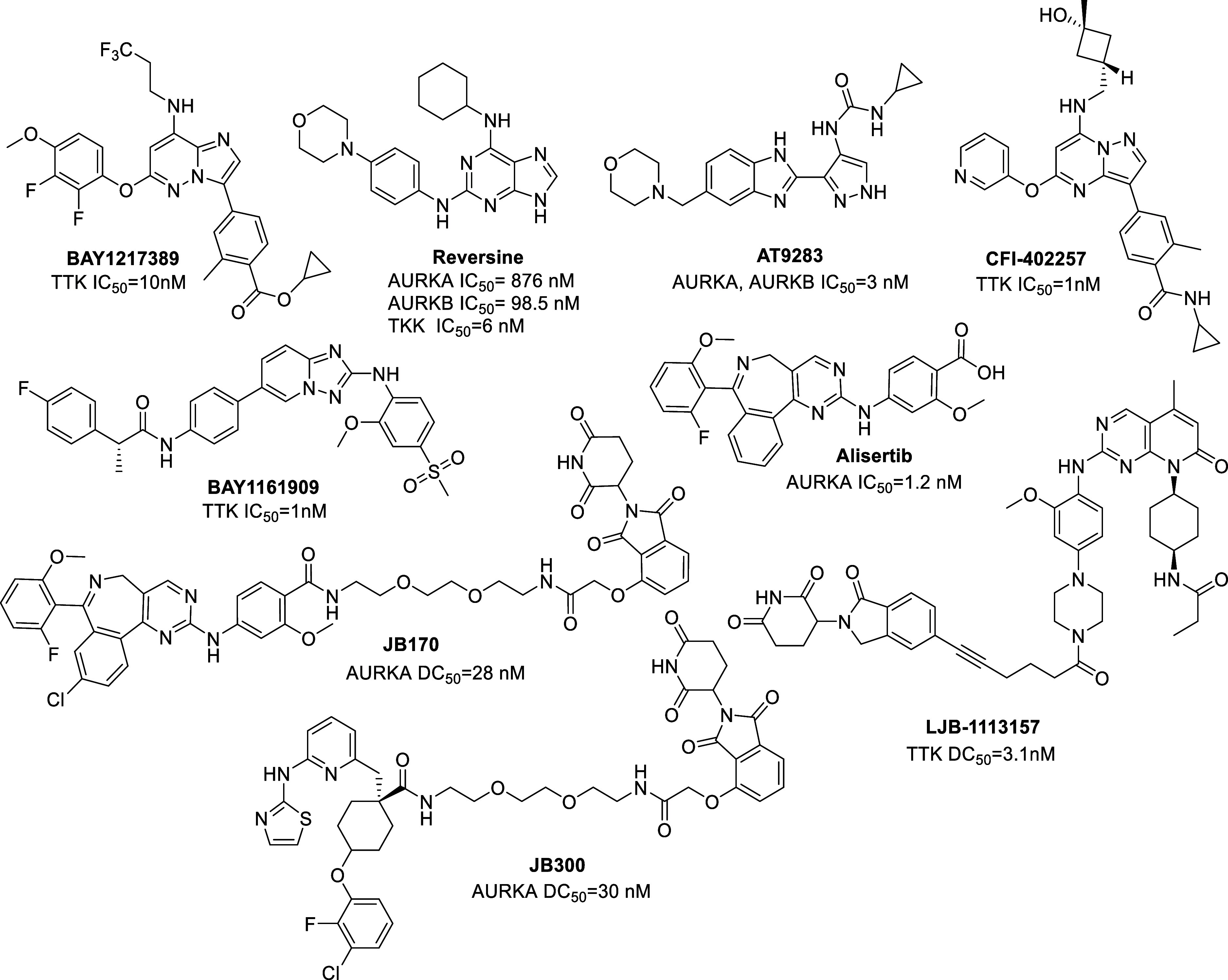
Small-molecule binders and PROTACS targeting AURKA, AURKB, and TTK.

In this paper, the synthesis and biological activity of 28 putative proteolytic chimeras targeting AURKs or TTK is reported, by employing a promiscuous kinase scaffold, 2-aminoadenine. 2-Aminoadenine is an adenine derivative that interacts with the hinge region in ATP-binding sites of kinases.^25,26^ Azareversine (a piperazine analogue of reversine) and the TTK-selective inhibitor **MPS1-IN-3** both contain the 2-aminoadenine core and were utilized herein as kinase binders for the synthesis of the PROTACs (Figure 2). Reversine is a promiscuous ATP-competitive inhibitor that has great affinity for TTK by forming three hydrogen bonds with the Gly605 and Glu603 residues of the hinge region, which is the binding domain of the adenine base of ATP.^26,27^ Considering the molecular targets of reversine, it inhibits the enzymic activity of AURKs A (IC_50_ = 876 nΜ), B (IC_50_ = 98.5 nΜ), C (IC_50_ = 400 nΜ), and human TTK with an IC_50_ value of 6 nΜ. On the other hand, **MPS1-IN-3** is a *N*-phenylpyrimidin-2-amine-based inhibitor that inhibits TTK with an IC_50_ of 50 nM. It was developed by Tannous et al. for the treatment of glioblastoma.^13^ The purine scaffold shared by azareversine and **MPS1-IN-3** is similar to the adenine base of ATP and is responsible for the formation of wide-spreading van der Waals forces with the residues Ile531, Val539, Met602, Ile586, and Leu654 of TTK’s binding pocket.^28^ Additionally, we developed two novel 2-aminoadenine-based hybrids, **SF1** and **SF2**, that incorporate structural features of azareversine and **MPS1-IN-3** to achieve different selectivity profiles against AURKs and TTK. During the development of MPS1-IN inhibitors against TKK, both the OMe and the 2-(isopropylsulfonyl)phenyl groups were defined as strong selectivity determining factors.^29^ The OMe forms a hydrogen bond with the hinge residue Gly605, and the 2-(isopropylsulfonyl)phenyl group forms various hydrophobic interactions. Pomalidomide and VHL-1 were used as cereblon (CRBN) and Von Hippel–Lindau (VHL) ligands for E3-ligase recruitment. Our research was based on the fact that promiscuous classical inhibitors can be turned into more selective chemical tools via their transformation to the corresponding PROTACs.^30^

**Figure 2:**
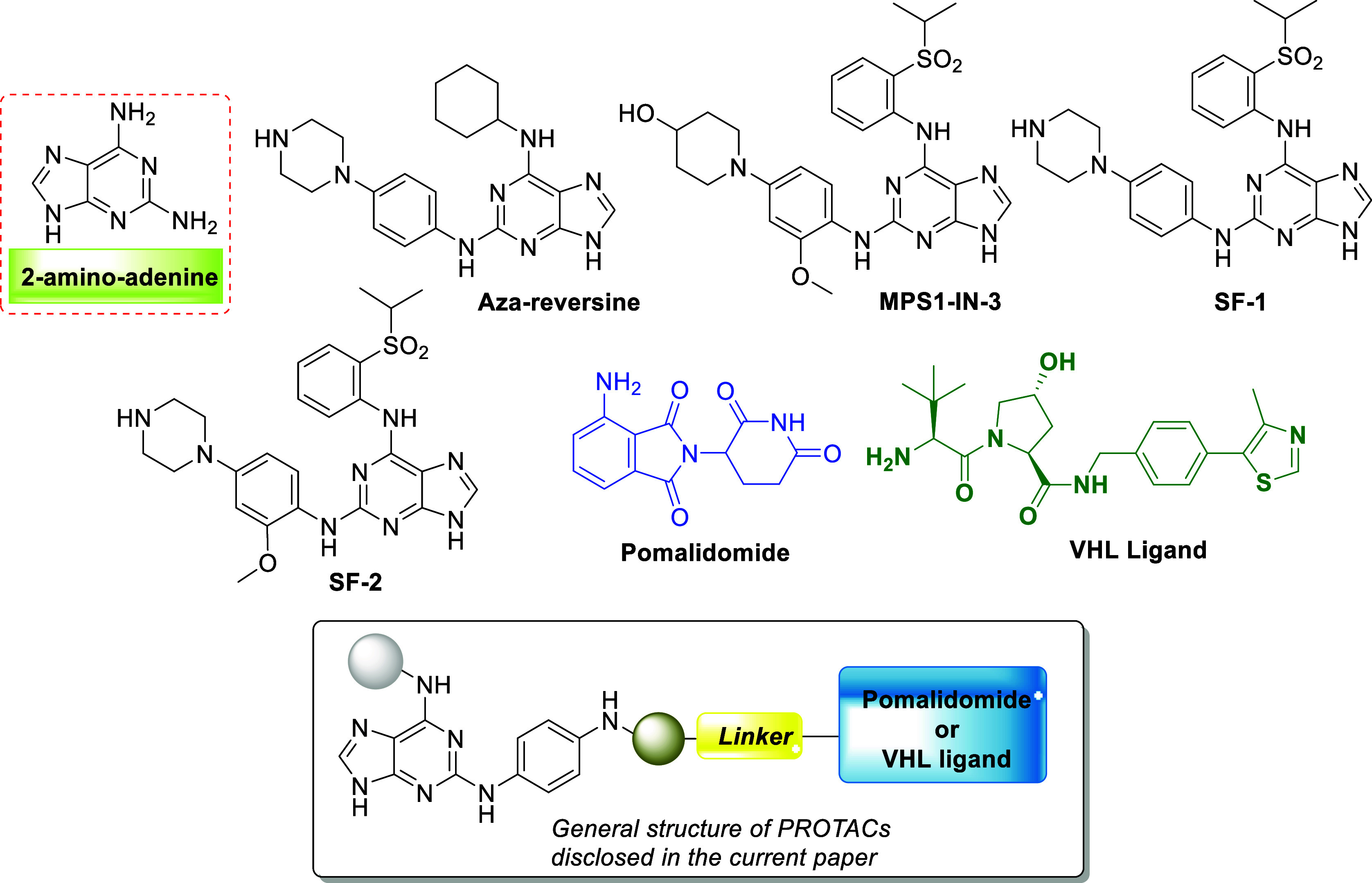
General structural units utilized herein to build AURK or TTK PROTACs.

## Results and Discussion

### Design and Synthesis of 2-Aminoadenine-Based PROTAC Degraders for AURKs and TTK

**Figure 3:**
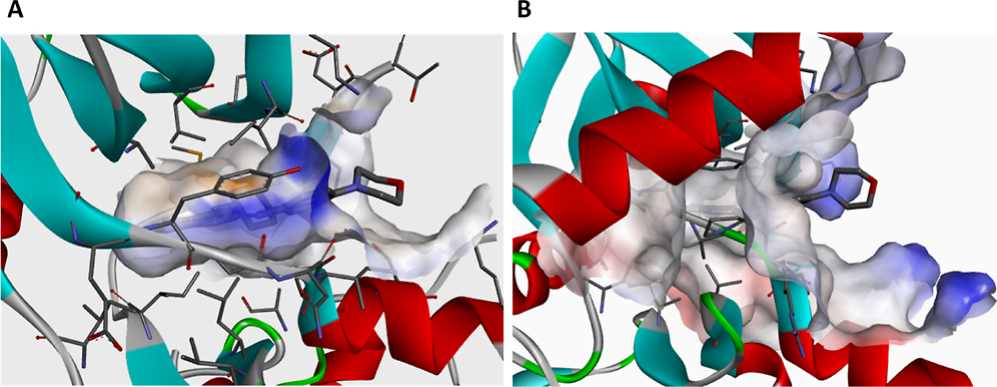
Crystal structures of reversine in complex with (A) TTK (PDB ID: 5LJJ) and (B) AURKB (PDB ID: 2VGO).

Examination of the available cocrystal structures of reversine in complex with TTK (PDB 5LJJ)^26^ and AURKB (PDB 2VGO)^31^ shows that the morpholine ring of reversine is solvent-exposed and can be considered as a possible point for linker attachment (Figure 3).

**Scheme 1:**
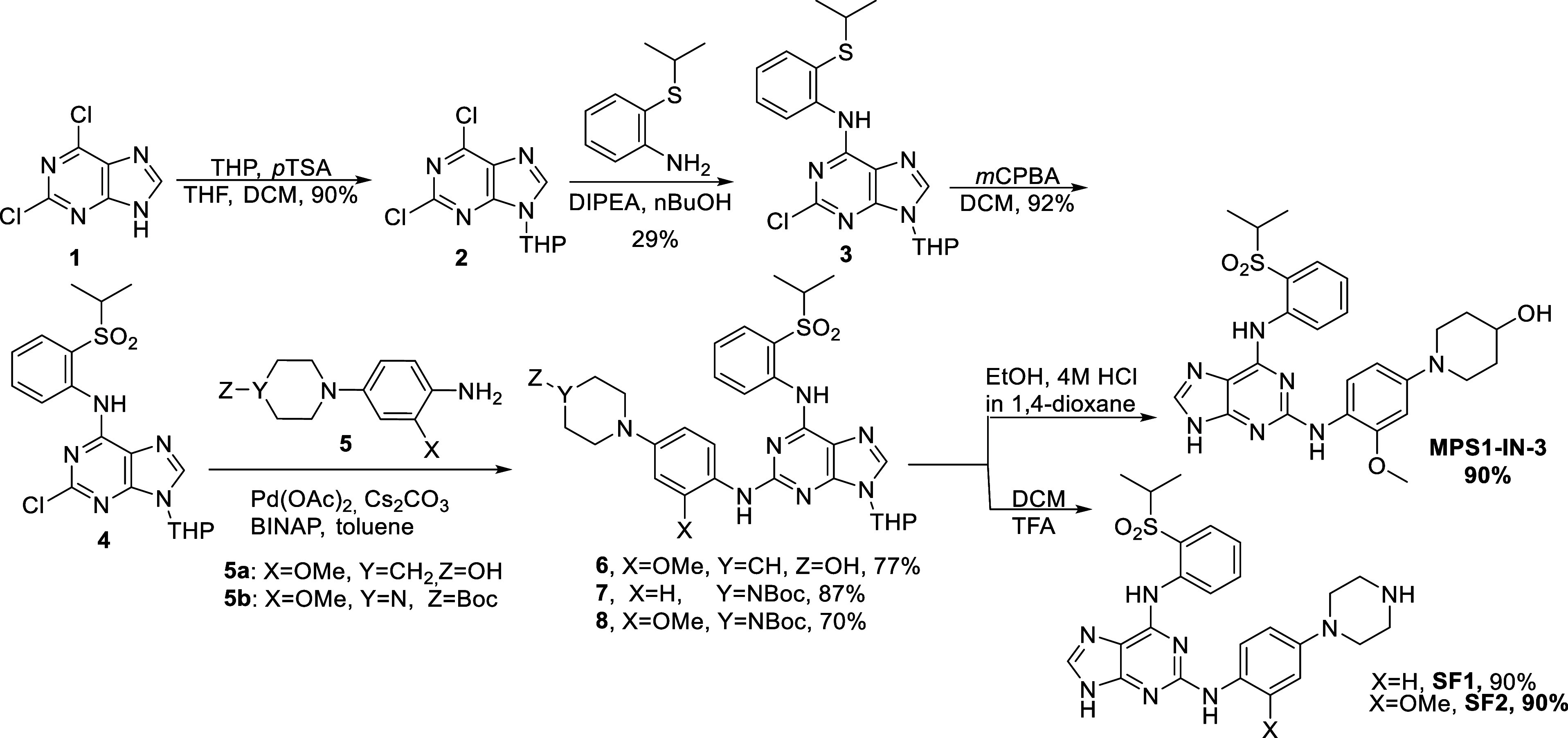
Synthesis of **MPS1-IN-3**, **SF1**, and **SF2**

Initially, azareversine and **MPS1-IN-3** inhibitors were synthesized according to previously reported studies.^13,32^ To get additional diverse ligands, two novel derivatives, **SF1** and **SF2**, were developed as hybrids of the two aforementioned inhibitors, bearing the isopropylsulfonyl-phenyl moiety of **MPS1-IN-3** and the aminophenyl piperazine moiety of azareversine. The synthesis of **MPS1-IN-3** inhibitor was performed according to Tannous et al.^13^ although the final step was replaced by a Buchwald–Hartwig reaction to afford the THP-protected inhibitor with an 87% yield, which was further deprotected. The synthesis of **SF1** and **SF2** derivatives was carried out in five steps with a 75% overall yield, utilizing once again the Buchwald–Hartwig reaction as the final step (Scheme 1). Afterward, a linker was introduced to azareversine, **SF1** and **SF2** through coupling with Boc-Gly-OH in high yields, as described in Scheme S1.

The two E3-ligands, pomalidomide and VH032, as well as their negative controls were synthesized as previously described.^33,34^ Then to the synthesized E3-ligands, miscellaneous linkers were introduced for the preparation of 28 derivatives of azareversine, **MPS1-IN-3**, and **SF1** and **SF2** compounds. The length and structure of the linkers varied between 3 and 13 atoms and included unsaturated carbon chains, etheric chains, or heterocycles. Amide bonds were selected for the conjugation of degraders to E3-ligands due to their stability, while the coupling reactions afforded the desired PROTACs in low to good yields. In total, eight VHL-bearing chimeras and 20 pomalidomide-bearing chimeras were prepared and are presented in Schemes S2, S6 and S8. The terminal hydroxyl group of inhibitor **MPS1-IN-3** was modified through an alkylation reaction with *tert*-butyl bromoacetate followed by TFA deprotection to add a carboxylic acid which would be used for the subsequent formation of the preferred amide bonds with the E3 ligands (Scheme S4).

### Biological Evaluation

**Figure 4:**
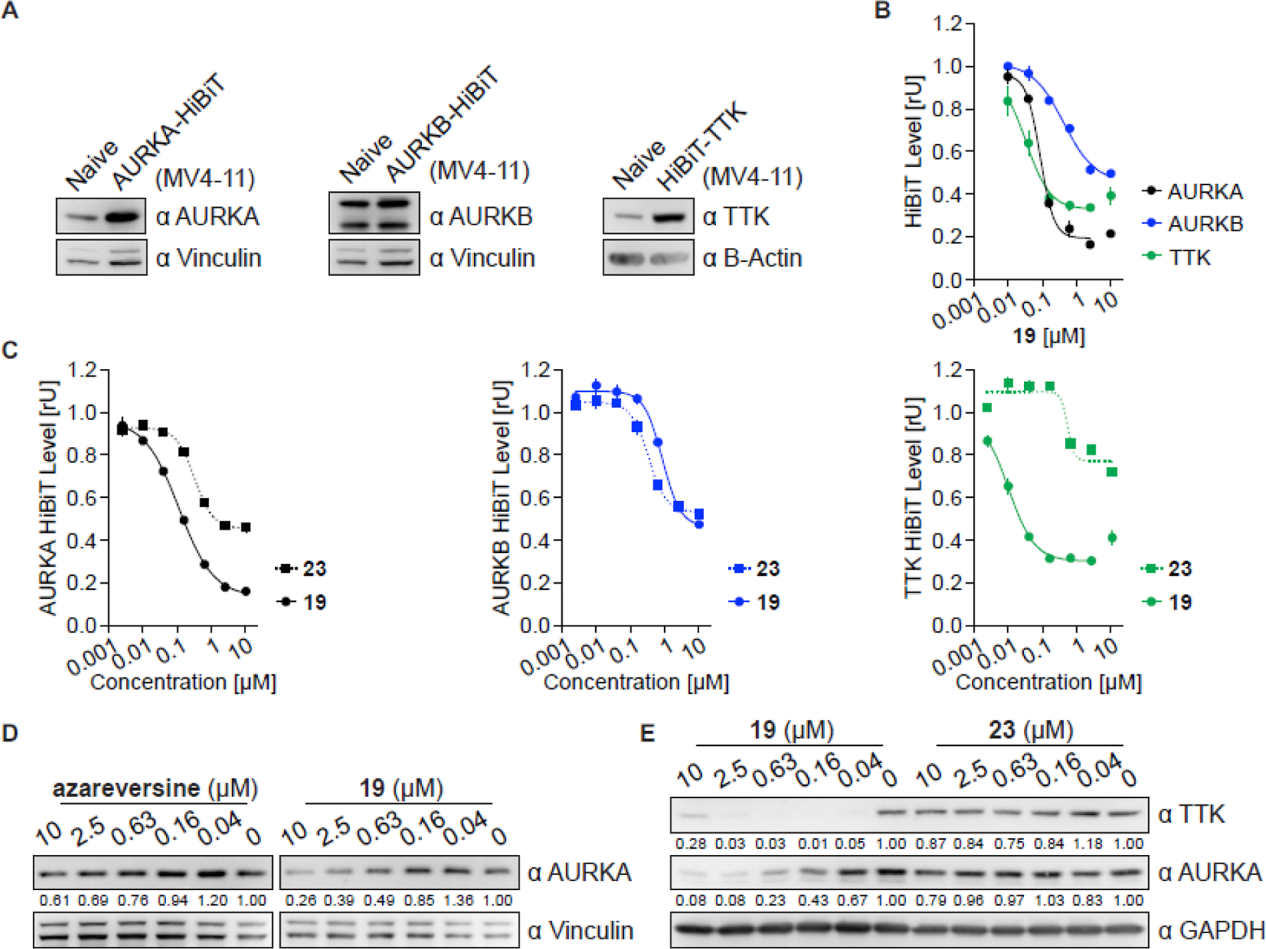
Cellular degradation studies of azareversine-based compounds. (A) Immunoblots of AURKA, AURKB, and TTK. HiBiT fragment were fused to AURKA, AURKB, and TTK kinases and stably expressed in MV4-11 cells. Naive MV4-11 and respective HiBiT MV4-11 cells are labeled. Vinculin and B-actin were used as loading controls (as in all other immunoblotting experiments, if not stated differently). (B,C) AURKA, AURKB, and TTK levels based on luciferase measurements. Indicated MV4-11 HiBiT cells were treated with different concentrations of **19** (B) and **19** or negative control **23** (C) for 6 h, lysed, complemented with the large luciferase fragment (largeBiT), and luciferase activity was measured. (D) Immunoblot of AURKA. AURKA-HiBiT cells were treated with different concentrations of azareversine and **19** for 6 h and compared with vehicle-treated cells. (E) Immunoblots of TTK and AURKA. Naive MV4-11 cells were treated with different concentrations of **19** and negative control **23** for 6 h and compared with vehicle-treated cells. All HiBiT data are represented as a mean ± s.d. from *n* = 2 replicates.

The effects of the synthesized compounds on AURKA, AURKB, and TTK mitotic kinases were then assessed in cellular systems. For this, HiBiT cell lines were generated for all three mitotic kinases. A small 11 amino acid peptide fragment of luciferase (HiBiT) was fused to the coding sequence of the proteins and stably transduced in the MV4-11 cell line (MV4-11^AURKA-HiBiT^, MV4-11^AURKB-HiBiT^, and MV4-11^HiBiT-TTK^). Immunoblots showed a slightly higher expression of the protein in the corresponding HiBiT cell line compared with the parental MV4-11 cells (Figure 4A). Even though, separate bands were not observed for the HiBiT-tagged and endogenous untagged protein, the higher expression in HiBiT cell lines demonstrated the expression of HiBiT-tagged protein.

First, we assessed a series of azareversine-based chimeras for the degradation of all three mitotic kinases. The depletion of the HiBiT-tagged kinases varied substantially between different degraders, with pomalidomide-bearing degraders being more active than the VHL-based compounds (Table 1). Some interesting but weak activity was recorded for VHL-based compound **43** with maximal degradation (*D*_max_) of 55% and DC_50_ of 684 nM against AURKA after 6 h of treatment. All three pomalidomide-based conjugates, **13**, **17**, and **19** displayed decent activities against all three kinases (Figures 4B and S1A). Of them, chimera **19** having a –COCH_2_NHCO(CH_2_)_3_CO– linker showed robust degradation of all three mitotic kinases (Figure 4B). The maximal depletion for AURKB, AURKA, and TTK was all induced by **19** with *D*_max_ of 43.6%, 78.8%, and 66.5% and DC_50_ of 570, 109, and 18 nM, respectively. Strikingly, when the parent compound azareversine was subjected to the HiBiT assay, it also showed a moderate decrease in all three kinases (Figure S1B, Table 1). Thus, to get further insight into the mode of action of CRBN-dependent PROTAC **19**, a comparable negative analog **23** was synthesized by incorporating the *N*-methyl group on the glutarimide ring of pomalidomide (Scheme S2). The compounds were then tested side by side in the HiBiT assay for degradation of AURKA, AURKB, and TTK. In comparison to **19**, **23** decreased the levels of all three kinases to a lower extent, with the most prominent difference being in TTK (Figure 4C). Negative control **23** showed a 34-fold lower activity (DC_50_) against TTK in comparison to that of the degrader **19** (Figure 4C, Table 1). Given the fact that synthesized derivative **23** induces depletion of all three kinases, it is probable that **19**-induced degradation of AURKA, AURKB, and TTK is only partly dependent on E3-ligase recruitment and subsequent ubiquitination.

**Table 1:**
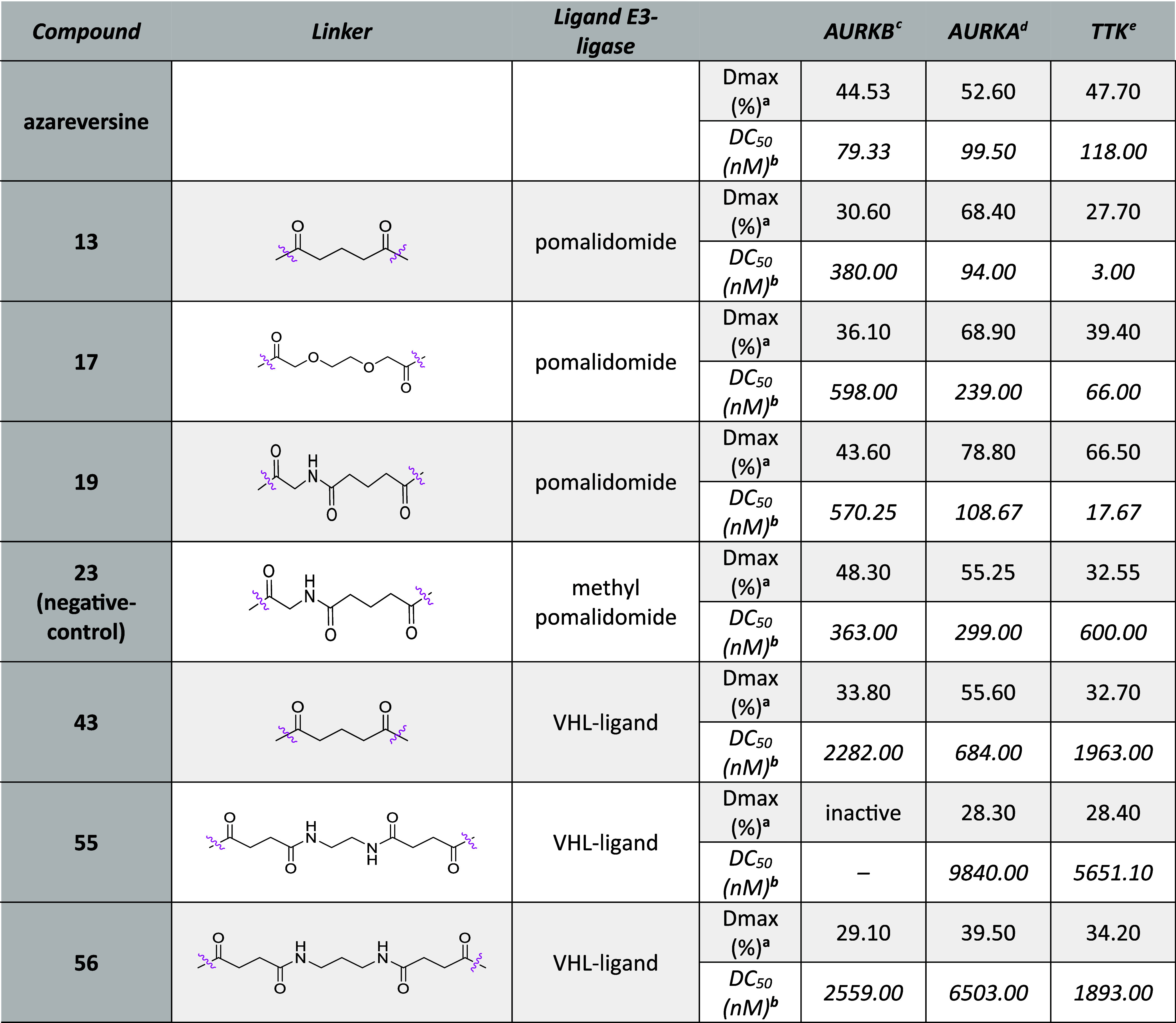
HiBiT Data of Azareversine-Based Chimeras after 6 h Treatment

a *D*_max_: maximal degradation, compounds with degradation less than 15% reported as “inactive”.

b DC_50_: half-maximal degradation concentration, calculated with the dose–response (four parameters) equation, compounds with degradation less than 25% were not calculated and reported as “–”.

c MV4-11^AURKB-HiBiT^ cells.

d MV4-11^AURKA-HiBiT^ cells.

e MV4-11^HiBiT-TTK^ cells.

In agreement with the luciferase-based HiBiT measurement, a dose-dependent decrease in the protein levels was observed by both compounds azareversine and **19** in MV4-11 HiBiT cell lines, as assessed by Western blot (Figures 4D and S1C). Next, we examined if the compounds led to the depletion of the endogenous untagged proteins similar to that of HiBiT-tagged proteins. As expected, compound **19** exhibited a robust decrease in AURKA and TTK levels while control **23** showed a moderate effect in naive MV4-11 cells (Figure 4E).

**Figure 5:**
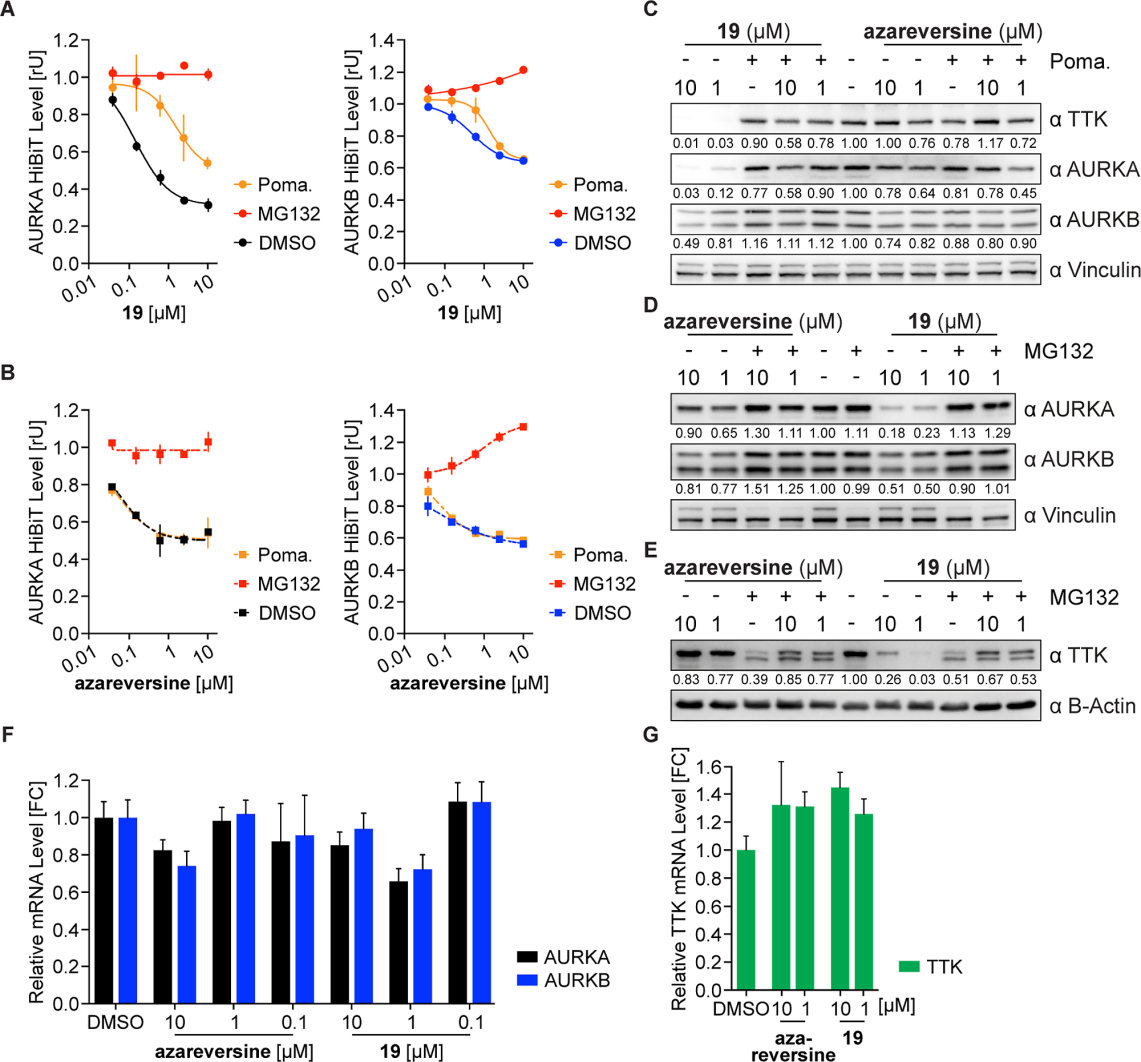
**19**-Induced depletion of TTK depends on the ubiquitin system. (A,B) AURKA and AURKB levels based on luciferase measurements. AURKA-HiBiT (left) and AURKB-HiBiT (right) cells were treated with various concentrations of **19** (A) or azareversine (B) in the presence or absence of proteasomal inhibitor MG132 (10 μM) or CRBN ligand pomalidomide (Poma., 10 μM) for 6 h. The cells were then subjected to HiBiT assay. (C) Immunoblots of AURKA, AURKB, and TTK. MV4-11 cells were treated for 6 h with indicated concentration of **19** or azareversine and pomalidomide (10 μM). (D,E) Immunoblots of AURKA, AURKB, and TTK. MV4-11 cells were treated for 6 h with indicated concentration of **19** or azareversine and MG132 (10 μM), and the level of AURKA and AURKB (D) and TTK (E) was compared with vehicle-treated cells. (F,G) Quantitative RT-PCR analysis of mRNA levels. RNA was extracted from MV4-11 cells incubated with various concentrations of **19** and azareversine for 6 h. AURKA and AURKB (F) and TTK (G) expression levels were normalized with a reference gene. Bars represent mean ± s.d. of *n* = 3 technical replicates. All HiBiT data are represented as a mean ± s.d. from *n* = 2 replicates.

To get a better understanding of the mechanism of action of azareversine-based chimeras, we decided to characterize chimera **19** further. As PROTAC-mediated degradation is reliant on the proteasomal system, AURKA- and AURKB-HiBiT cells were cotreated with degrader **19** and proteasomal inhibitor MG132. Inhibiting the proteasomal activity with MG132 completely rescued the **19**-mediated degradation of both proteins (Figure 5A). However, coincubation of **19** with pomalidomide only lowered the degradation efficacy of **19** against AURKA and AURKB in MV4-11 HiBiT cells (Figure 5A). Contrarily, only MG132 but not pomalidomide restored azareversine-mediated protein depletion, highlighting that the decrease in the protein level by azareversine is not mediated via CRBN (Figure 5B). Moreover, the degradation of TTK, AURKA, and AURKB by **19** was also rescued by proteasomal inhibition and competition with the E3-ligase ligand in naive MV4-11 cells (Figure 5C–E). Finally, to investigate the effect of azareversine and **19** on AURKA, AURKB, or TTK transcription, MV4-11 cells were treated with different concentrations of the compounds and mRNA levels were quantified by RT-qPCR. While both compounds induced depletion of cellular AURKA, AURKB, and TTK proteins, only mRNA levels of AURKA and AURKB were diminished (Figure 5F,G). However, the mRNA level decrease was to a lesser degree in comparison to protein levels and was observed to be concentration dependent. Interestingly, the mRNA level of TTK was increased at all used concentrations of both compounds (Figure 5G). We concluded that the decrease in the AURKA and AURKB protein levels by the chimeras could be due to the combinatorial effect of both degradation and a decrease in mRNA levels or any other mechanisms, whereas that of TTK was solely due to PROTAC-mediated degradation. The finding is in line with previous reports showing a reduction in AURKA and AURKB protein levels^35−37^ with inhibitor reversine.

Next, we proceeded to examine **MPS1-IN-3** and its six derivatives aiming to discover more selective degraders against one of the three kinases. Strikingly, none of the synthesized compounds exhibited significant AURKA, AURKB, or TTK degradation (Table 2). Only some weak activity was observed with **40**, however, the selectivity and efficiency were not improved compared to azareversine derivatives.

**Table 2:**
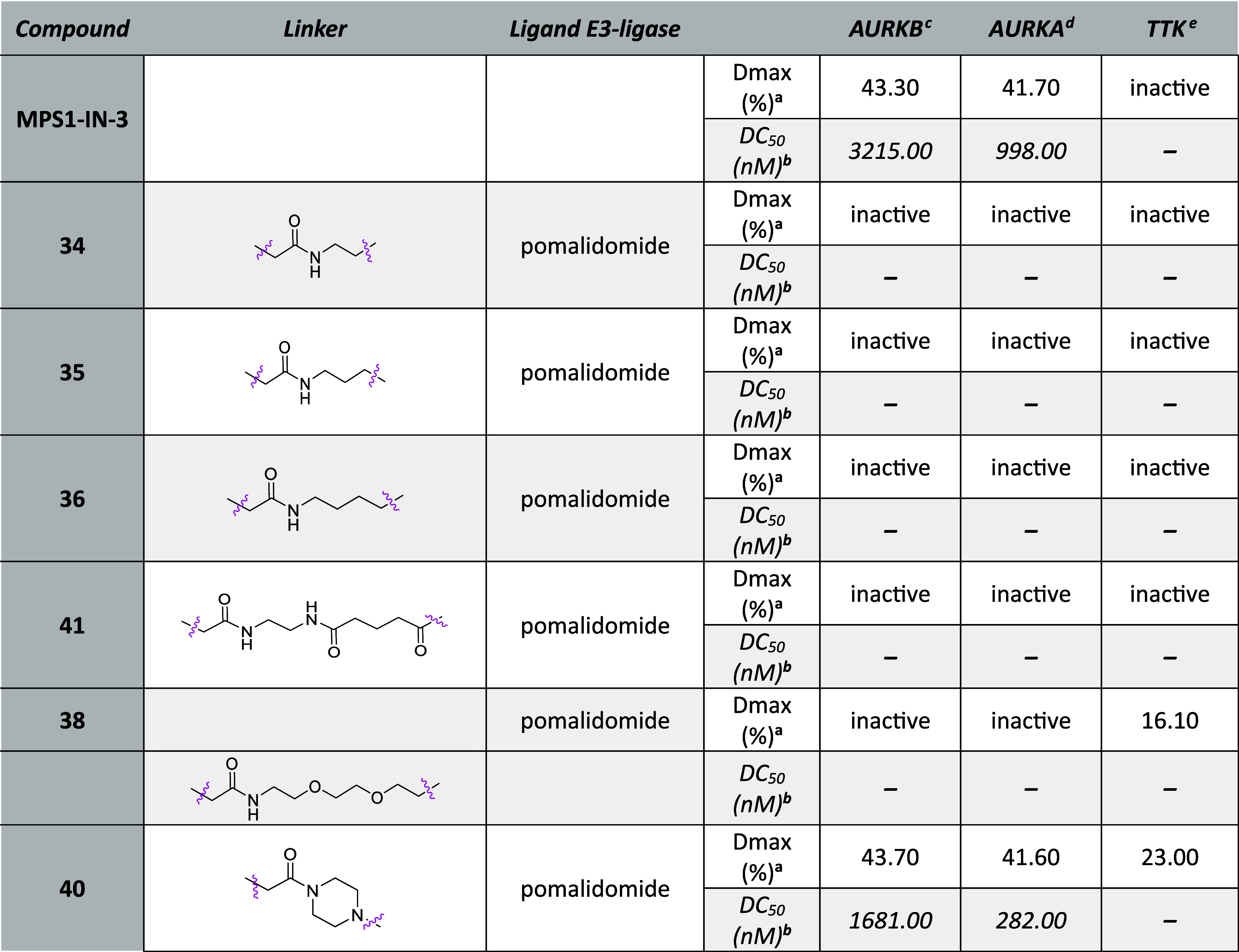
HiBiT Data of **MPS1-IN-3**-Based Chimeras after 6 h Treatment

a *D*_max_: maximal degradation, compounds with degradation less than 15% reported as “inactive”.

b DC_50_: half-maximal degradation concentration, calculated with the dose–response (four parameters) equation, compounds with degradation less than 25% were not calculated and reported as “–”.

c MV4-11^AURKB-HiBiT^ cells.

d MV4-11^AURKA-HiBiT^ cells.

e MV4-11^HiBiT-TTK^ cells.

**Figure 6:**
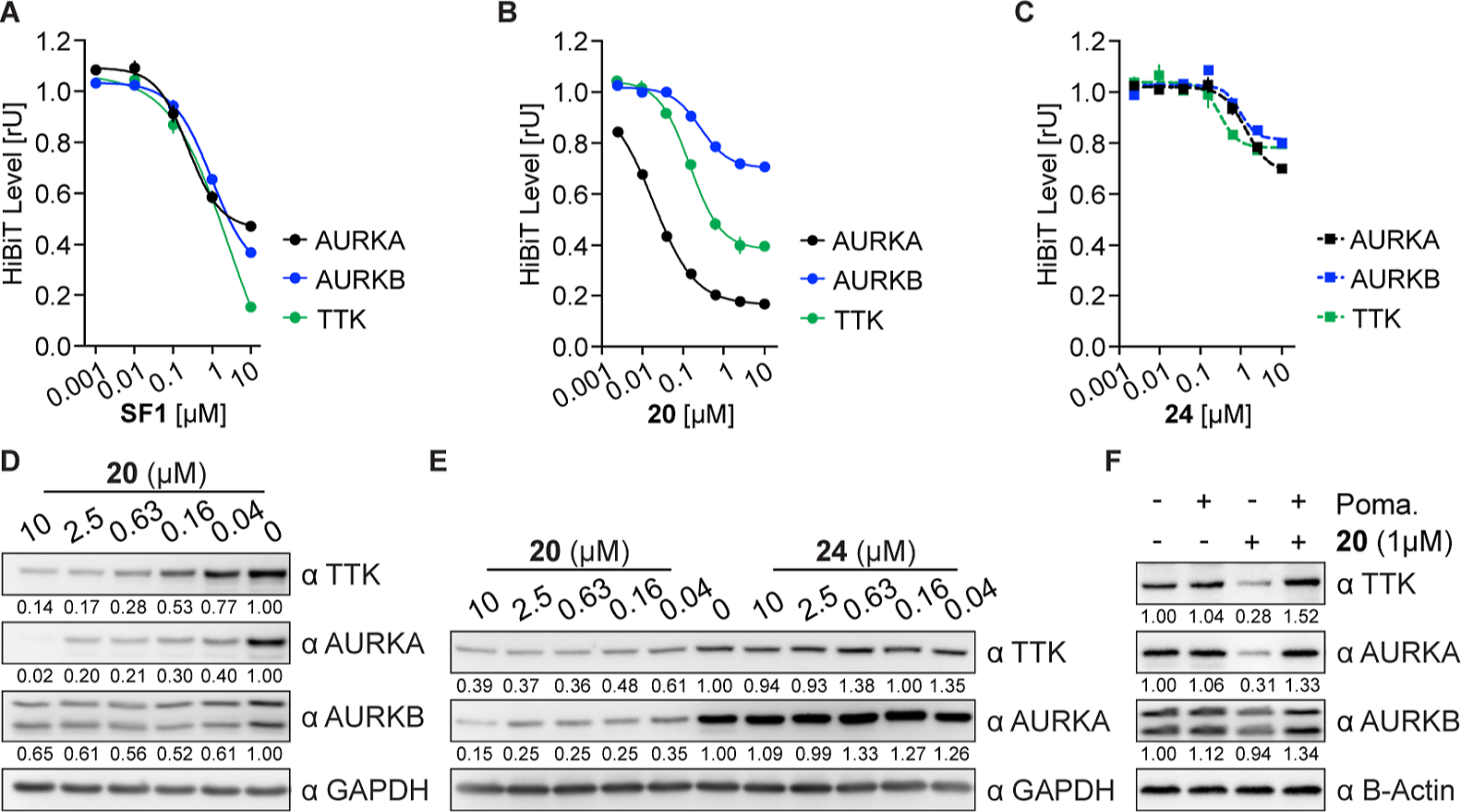
Cellular degradation studies of **SF1**-based compounds. (A–C) AURKA, AURKB, and TTK levels based on luciferase measurements. Indicated MV4-11 HiBiT cells were treated with different concentrations of **SF1** (A), **20** (B), and negative control **24** (C) for 6 h, lysed, complemented with the large luciferase fragment (largeBiT), and luciferase activity was measured. (D) Immunoblots of AURKA, AURKB, and TTK. HiBiT-TTK cells were treated with different concentration of **20** for 6 h and compared with vehicle-treated cells. GAPDH was used as a loading control. (E) Immunoblots of TTK and AURKA. Naive MV4-11 cells were treated with different concentrations of **20** and negative control **24** for 6 h and compared with vehicle-treated cells. (F) Immunoblots of AURKA, AURKB, and TTK. MV4-11 cells were treated for 6 h with 1 μM **20**, pomalidomide (10 μM) or combination of both. All HiBiT data are represented as a mean ± s.d. from *n* = 2 replicates.

Subsequently, we turned our attention to the **SF1** ligand and its derivatives. Interestingly, **SF1** ligand diminished AURKA, AURKB, and TTK kinase levels to an even greater degree than azareversine but with higher DC_50_ values (Figure 6A, Table 3). Like azareversine degraders, the pomalidomide-bearing derivatives of **SF1** were more active than the VHL ones, while the selectivity of the **SF1** derivatives was altered toward the AURKA kinase. The most potent pomalidomide compounds were **20**, **15**, **28**, and **27** which induced superior AURKA degradation with low nanomolar DC_50_ values (Figures 6B and S2A–C and Table 3). Compound **20** degraded preferentially AURKA with DC_50_ of 68 nM and *D*_max_ of 71% which is 3.7–4.2-fold selective and 1.5–2.4 times more efficient than toward AURKB and TTK. For better comparison, a negative control of **20** (**24**) bearing the –COCH_2_NHCO(CH_2_)_3_CO– linker and methyl pomalidomide was synthesized and tested. We found that **24** depleted all three proteins with minimal efficacy, suggesting that the degradation of the kinases induced by **20** is mediated by E3-ligase CRBN (Figure 6C). Moreover, the length and the chemical composition of the linker seem to determine the degradation potencies and selectivity profiles. Comparing the activities of **15** containing a shorter linker (4 atom chain length) with chimeras containing longer linker like **14** (5 atom length), **30** (7 atom length), and **20** (8 atom chain length), we found that the shorter linker favors selectivity against AURKA (Figures 6B and S2A and Table 3). Additionally, the absence of a carbonyl group in **27** and **28** compared with **15** and **14** increases the efficacy against TTK (Figure S2B,C and Table 3). On the other hand, the most potent VHL-based compound was **46** against AURKA and **49** against TTK (Table 3).

**Table 3:**
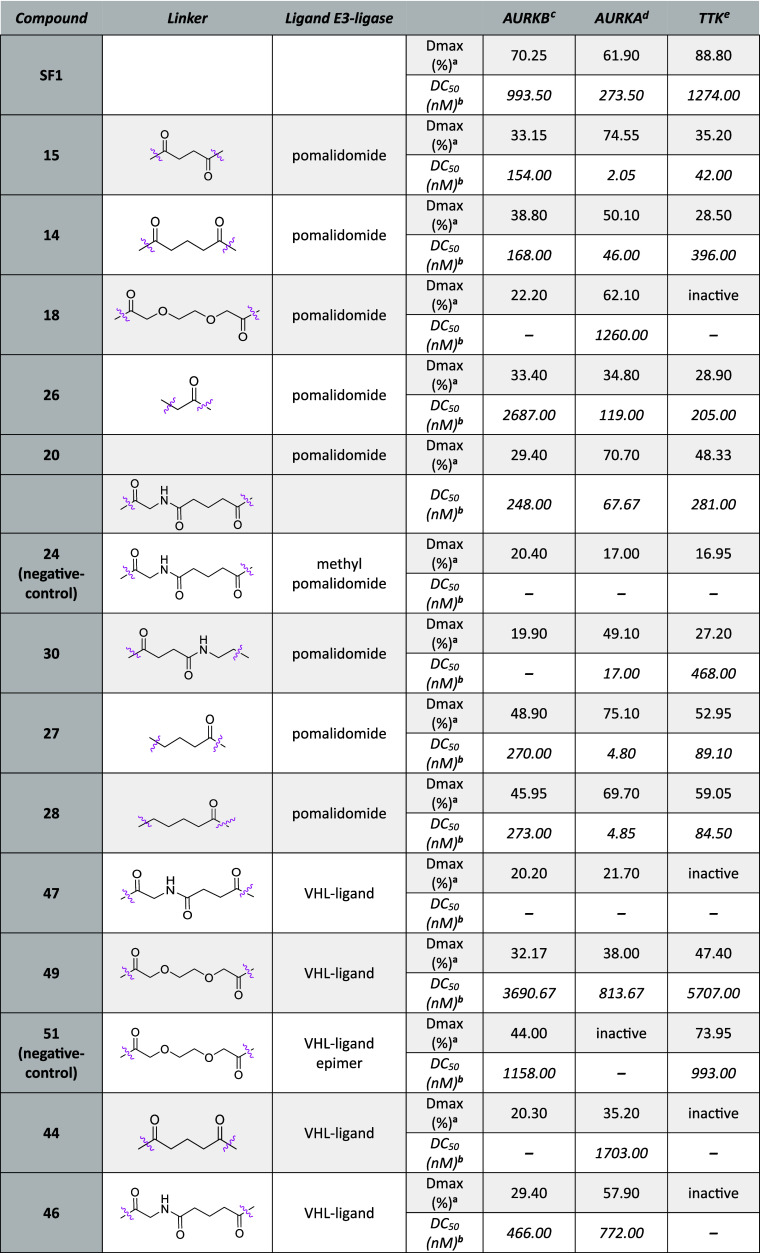
HiBiT Data of **SF1**-Based Chimeras after 6 h Treatment

a *D*_max_: maximal degradation, compounds with degradation less than 15% reported as “inactive”.

b DC_50_: half-maximal degradation concentration, calculated with the dose–response (four parameters) equation, compounds with degradation less than 25% were not calculated and reported as “–”.

c MV4-11^AURKB-HiBiT^ cells.

d MV4-11^AURKA-HiBiT^ cells.

e MV4-11^HiBiT-TTK^ cells.

The observed activities of **SF1**-based chimeras in the HiBiT assay were also verified by Western blot where the treatment of both MV4-11 HiBiT and naive cells showed a dose-dependent decrease in the protein levels (Figures 6D and S2D–G). Furthermore, comparing the immunoblot of **24**-treated cells and cotreatment with pomalidomide confirmed that **20** mediates the degradation of AURKA, AURKB, and TTK proteins via CRBN (Figure 6E,F). Finally, we also examined whether the degradation was limited to MV4-11 cells or if the chimeras were also functional in other cell lines. We thus treated the human non-small cell lung cancer cell line, CALU1 with **20** along with other compounds and observed similar depletion as seen in MV4-11 cells (Figure S2H–J).

In an effort to alter the selectivity profile of the degrader, we synthesized a close analogue of **20** (**21**) by employing another ligand **SF2**, bearing a methoxy group on the phenyl ring (Scheme 1, Table 4). HiBiT data show that the compound did not degrade AURKB but was active against AURKA and TTK (Figure 3A–C, Table 4). Even though the compound increased selectivity toward AURKA and TTK, the efficiency was not enriched compared to **20** or **19** bearing same linker and E3-ligand.

**Table 4:**
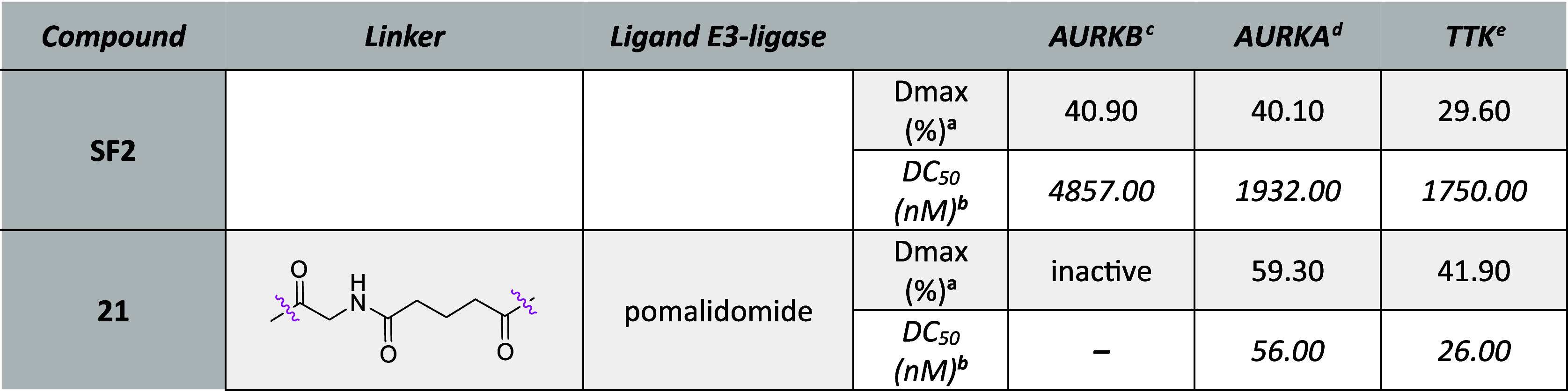
HiBiT Data of **SF2**-Based Chimera after 6 h Treatment

a *D*_max_: maximal degradation, compounds with degradation less than 15% reported as “inactive”.

b DC_50_: half-maximal degradation concentration, calculated with the dose–response (four parameters) equation, compounds with degradation less than 25% were not calculated and reported as “–”.

c MV4-11^AURKB-HiBiT^ cells.

d MV4-11^AURKA-HiBiT^ cells.

e MV4-11^HiBiT-TTK^ cells.

**Figure 7:**
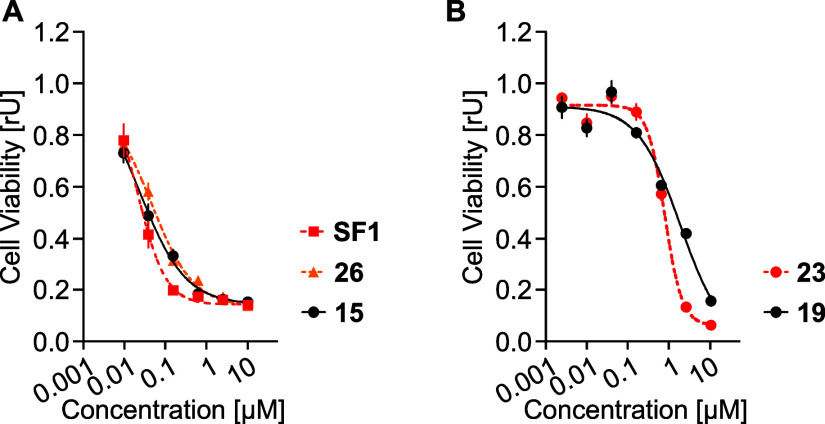
Cell viability studies of the chimeras. (A) Cell viability measurement. MV4-11 cells were incubated with various concentrations of **SF1**, **26**, or **15** for 72 h and compared. (B) Cell viability measurement. MV4-11 cells were incubated with various concentrations of **19** or negative control **23** for 72 h. Data are represented as a mean and ±s.d. from *n* = 3 replicates.

Finally, to study the effect of the compounds on cancer cell survival, MV4-11 cells were treated with various concentrations of the chimeras and the cell viability was assessed after 72 h. Interestingly, the parent inhibitors from each series were more toxic than their derivatives, with the most potent being **SF1** in terms of EC_50_ (Figures 7A and S7A–C and Table 5). As opposed to the parent inhibitor **MPS1-IN-3**, its derivatives except **40** were not cytotoxic (Figure S7B, Table 5). On the other hand, the azareversine-based compound **19** was cytotoxic with EC_50_ of 1.4 μΜ but was less active compared to its negative control **23** which had EC_50_ of 0.7 μΜ (Figure 7B, Table 5). In addition, most of the **SF1**-based derivatives affected the viability of MV4-11 cells in nanomolar concentrations (Table 5). Derivatives **15** and **26** were the most potent chimeras, displaying equipotent cytotoxicity with the **SF1** inhibitor (Figure 7A). The cytotoxic effect of compounds like **26** with moderate degradation of AURKs and TTK suggests a potential off target effect. Importantly, **15** was even more potent than the ligand azareversine. Moreover, a direct comparison of the potency of **20** with that of its negative controls showed no significant differences (Figure S7D, Table 5).

**Table 5:** Cell Viability Data of All Chimeras after 72 h Treatment in MV4-11 Cells (Naive)

compound	ligand E3-ligase	EC_50_ (nM)^a^	compound	ligand E3-ligase	EC_50_ (nM)^a^
azareversine		123	**15**	pomalidomide	39
**13**	pomalidomide	1208	**14**	pomalidomide	214
**17**	pomalidomide	506	**18**	pomalidomide	1127
**19**	pomalidomide	1384	**26**	pomalidomide	57
**23**	methyl pomalidomide	697	**20**	pomalidomide	577
**43**	VHL-ligand	584	**24**	methyl pomalidomide	618
**55**	VHL-ligand	6906	**30**	pomalidomide	405
**56**	VHL-ligand	6822	**27**	pomalidomide	91
**MPS1-IN-3**		1274	**28**	pomalidomide	126
**34**	pomalidomide	—	**47**	pomalidomide	—
**35**	pomalidomide	—	**49**	VHL-ligand	2556
**36**	pomalidomide	—	**51**	VHL-ligand epimer	421
**41**	pomalidomide	—	**44**	VHL-ligand	1161
**38**	pomalidomide	—	**46**	VHL-ligand	1983
**40**	pomalidomide	836	**SF2**		2060
**SF1**		28	**21**	pomalidomide	—

a EC_50_: effective concentration that decreases cell viability by 50%, calculated with the dose–response (four parameters) equation, and compounds with decrease of cell viability less than 50% were not calculated and reported as “—”.

### Chemostability Profile of Degrader **20**

Lastly, to gain a first insight into the stability of the synthesized compounds in a biological system, the stability of one of the degraders was examined at two different pH values and in human plasma (Figures S8 and S9). We chose one of the best degraders of the **SF1** series, **20** as it also resembled the best degrader from azareversine series **19** in terms of the linker and E3-binding moiety. All experiments were carried out at 37 °C, and aliquots were analyzed using LC-ESI-MS at predetermined time points. Chimera **20** was stable at both pH values for 8 h, while after 24 h, its residual concentration was significantly decreased and reached 56% of its initial concentration at pH 5.2 and 11.2% of its initial concentration at pH 7.4 (Figure S11A). Furthermore, **20** was less stable in human plasma with a half-life (*t*_1/2_) of 4.4 h (Figure S11B). We concluded from the stability profile of **20** that it is sufficient for further testing.

## Conclusions

In this study, we designed and synthesized a series of 2-aminoadenine-based chimeras and studied their effect on monopolar spindle 1 (Mps1/TTK) and AURKs. We linked four different target ligands to E3-ligase ligands by using various linkers. By varying the linker length and flexibility, 19 was identified as an azareversine-based TTK degrader with DC_50_ of 18 nM and *D*_max_ of 67% in MV4-11 HiBiT cells. In addition, the degradation of TTK by **19** was selective over that of AURKA and AURKB kinases. Moreover, we confirmed that **19** mediated the degradation of TTK through the ubiquitin-protease system. The decrease of AURKA and AURKB mRNA levels but not of TTK by **19** and azareversine suggested that their direct target is TTK. Surprisingly, when the substitution on the NH_2_ group of position 6 on the 9*H*-purine-2,6-diamine core structure was changed from cyclohexyl to 2-(isopropylsulfonyl)phenyl, the efficiency of the compounds reduced drastically. The 2-(isopropylsulfonyl)phenyl group is a structural feature of the selective TTK inhibitors, **MPS1-IN-3**. Intriguingly, the replacement of 1-(3-methoxyphenyl)piperidinol moiety of **MPS1-IN-3** with 1-phenyl piperazine moiety for the **SF1** inhibitor resulted in several chimeras with low nanomolar potencies. **SF1**-based PROTACs degrade AURKA, AURKB, and TTK kinases, while the selectivity of the **SF1** derivatives was altered toward the AURKA kinase. The most potent pomalidomide compound **15** induced AURKA degradation with low nanomolar DC_50_ value of 2.05 nM, which is 77-fold and 21-fold more selective toward AURKB and TTK, respectively. The cytotoxicity of the synthesized chimeras against MV4-11 cancer cells was lower than the parent compounds; however, we were able to identify compounds with very potent antiproliferative activities such as **15** with an EC_50_ value of 39 nM. Notably, certain parent inhibitors and negative controls demonstrated decreased steady-state protein levels without E3-ligase recruitment, indicating potential kinase destabilization through alternative mechanisms, as supported by recent literature.^38^ This finding emphasizes that these negative controls may not serve as suitable controls in cell biology experiments. Additionally, the observed discrepancies between degradation efficacy and cellular efficacy in some compounds underscore a critical need for further investigation into their underlying mechanism of action.

Collectively, these results suggest that **19** represents a novel TTK degrader that warrants further investigation. Degrader **19** is a valuable research tool compound for in vitro and in vivo studies and a promising lead compound for further optimization. Moreover, further investigations related to the potent **SF1**-based chimeras are warranted. Degrader **15** that induces potent AURKA degradation and possesses significant antiproliferative effects could be included in the AURKA-directed protein degradation tools, after a detailed study of the molecular mechanisms of action and optimization. Overall, our studies highlight the important role of the linker in the formation of a stable ternary complex between the target kinase AURKA, AURKB, or TTK and the E3-ligase. It was observed that the choice of the linker and modification in ligand could change the target degradation efficiency and specificity. Although the mechanism of action of the synthesized chimeras remains to be fully elucidated, and the negative controls show a degrading activity, several degraders of this study are compelling candidates for further exploration in anticancer research.

## Experimental Section

### Chemistry

Unless otherwise specified, all reactions for the synthesis of the conjugates were carried out under an atmosphere of argon in dried flasks or vials. Reagents were purchased from commercial suppliers and used without further purification. Reactions were monitored by thin-layer chromatography visualized by UV light and/or ethanolic *p*-anisaldehyde solution, aqueous ceric sulfate/phosphomolybdic acid, and heat. NMR spectra were recorded at °C on two spectrometers 500 MHz [Agilent 500 DD2 (500 MHz for ^1^H and 125 MHz ^13^C) and (300 MHz for ^1^H and 76 MHz ^13^C)] with tetramethylsilane as an internal standard. Chemical shifts are given in ppm from internal reference peaks (TMS ^1^H = 0.00 ppm; CDCl_3_^1^H = 7.26 ppm; CDCl_3_^13^C = 77.00 ppm; DMSO-*d*_6_^1^H = 2.50 ppm; DMSO-*d*_6_^13^C = 39.51 ppm; (CD_3_)_2_CO ^1^H = 2.05 ppm; (CD_3_)_2_CO ^13^C = 29.85 and 206.26 ppm). The LC–MS spectra were acquired using a LC20AD Shimadzu connected to Shimadzu LCMS-2010EV equipped with SUPELCODiscovery [C18, 25 cm × 4.6 mm, 5 μm] column. Infusion experiments were carried out on an Agilent Q-TOF Mass Spectrometer, G6540B a model with Dual AJS ESI-MS. All the compounds (dissolved in LC–MS grade, methanol) were introduced into the ESI source of the MS with a single injection of 20 μL of the sample and with a flow rate of 400 μL/min of 90% H_2_O + 0.1% HCOOH/10% ACN +0.1% HCOOH as a solvent in the binary pump. The experiments were run using a Dual AJS ESI source, operating in a positive ionization mode. Source operating conditions were 330 °C gas temp, 8 L/min gas flow, sheath gas temp 250 °C, sheath gas flow 10 L/min, and 50–100 V fragmentor. All accurate mass measurements of the [M + H]^+^ or [M + Na]^+^ ions were achieved at a mass rate of 50–1500 *m*/*z* in the positive mode. The Q-TOF was calibrated 1 h prior to the infusion experiments using a calibration mixture. Data were acquired in the external calibration mode. The purification of compounds by HPLC was achieved using an Agilent Infinity II 1080 connected to an automated fraction collector, equipped with a C18 analytical Reprospher 100 (C18-DE, 5 μm, 250 × 10 mm, Dr Maisch GmbH) column, and performed with Scientific Systems, Inc. instrumentation comprising 4-Q Grad Pumps connected to diode array UV–vis Thermo Finnigan Spectra system UV6000LP Drtector, Lab Alliance (NY, USA). HRMS experiments were carried out on a Q Exactive Plus Mass spectrometer; flow rate 0.5 mL/min; 90% ACN +0.1% HCOOH; spray voltage = 3 kV; capillary temperature = 300 °C.

### Compound Synthesis

The synthetic procedures for the synthesized compounds are reported in the Supporting Information Section.

## Biology

### Cell Culture

Human MV4-11 (male) and human Calu-1 (male) cells were cultured in RPMI-1640 medium, whereas human HEK293 (female) cells were cultured in DMEM medium at 37 °C in the presence of 5% CO_2_. Both RPMI-1640 and DMEM media were supplemented with 10% FBS and 1% penicillin/streptomycin.

### Cloning

AURKA-HiBiT vector was generated, as previously described (PMID: 32989298). AURKB-HiBiT was cloned by PCR amplification of cDNA (MV4-11 cells) as a template using forward CGCACCGGTATGGCCCAGAAGGAGAACTC and reverse primer CGCGACGCGTCTAGCTAATCTTCTTGAACAGCCGCCAGCCGCTCACACCGGAGCTCCCGGCGACAGATTGAAG. The PCR product was inserted into the pRRL-PGK vector using the AgeI/MluI site. The AURKB in the HiBiT construct used is isoform-5. Similarly, HiBiT-TTK was cloned by PCR amplification of cDNA (MV4-11 cells) as a template using a forward primer, CGCACCGGTGAATCCGAGGATTTAAGTGGC and a reverse primer, CGCGACGCGTTCATTTTTTTCCCCTTTTTTTTTC. The PCR product was digested and ligated into the pRRL-PGK-HiBiT entry vector.

### Cell Line Generation

The stable cell lines MV4-11^AURKA-HiBiT^, MV4-11^AURKB-HiBiT^, and MV4-11^HiBiT-TTK^ cells were generated by lentiviral infection. Lentivirus was produced using the respective HiBiT vector, plasmids psPAX2, and pMD2.G in HEK293 cells. The virus supernatant was filtered and MV4-11 cells were doubly infected with the supernatant. The cells were selected after 48 h of infection until the control cells were dead to get the stable cell line.

### HiBiT Assay

The cells stably expressing HiBiT-tagged protein were seeded into 96-well plates and treated with serial dilutions of compounds for the indicated time points. At the end point, the assay was performed using the Nano-Glo HiBiT Lytic Detection System (Promega) according to the manufacturer’s instruction. Luminescence was measured on a GloMax 96 Microplate Luminometer (Promega) or a Tecan Spark Multiplate reader (Tecan). DC_50_ was calculated using concentrations showing sigmoidal behavior with the dose–response (four parameters) equation in Prism (GraphPad).

### Immunoblotting

The cells were treated with compounds or vehicle for indicated time points and lysed in RIPA lysis buffer (50 mM HEPES pH 7.9, 140 mM NaCl, 1 mM EDTA, 1% Triton X-100, 0.1% SDS, 0.1% sodium deoxycholate) containing protease and phosphatase inhibitors (Sigma) for 20 min at 4 °C. Cell lysates were cleared by centrifugation, and protein concentration was determined with bicinchoninic acid assay. Equal amounts of protein per sample were separated by Bis-Tris-PAGE and transferred to PVDF membranes (Millipore). The membranes were blocked with 5% (w/v) nonfat dry milk in TBS-T [20 mM Tris–HCl, pH 7.5, 150 mM NaCl, 0.1% (v/v) Tween-20] for 1 h at room temperature and incubated with primary antibody at 4 °C overnight. Visualization was carried out using horseradish peroxidase (HRP)-labeled secondary antibodies and detected using a chemiluminescent HRP substrate (Millipore) in LAS3000 or LAS4000 mini (Fujifilm). Vinculin, GAPDH, or B-actin were used as a loading control. The loading control was probed along with the target antibody by cutting the membrane. For targets with similar molecular weights, the samples were run in duplicate and probed with their respective loading controls, although only one control was shown in the figure for all targets. The signal was quantified using ImageJ or Image Studio Lite against the corresponding loading controls. Antibodies used in this study were as follows: Aurora A/AIK (Cell Signaling Technology, 3092), Aurora B (Bethyl Laboratories, A300–431A), TTK (Santa Cruz Biotechnology, sc-56968), GAPDH (Cell Signaling Technology, 2118), B-Actin (Sigma, A5441), and Vinculin (Sigma, V9131).

### RT-qPCR

Total RNA was extracted after compound treatment using peqGOLD TriFast (Peqlab). Synthesis of cDNA was carried out using isolated RNA, random primers, Ribolock RNase inhibitor, dNTPs, and MLV reverse transcriptase. The cDNA was analyzed by qPCR on a StepOnePlus Real-Time PCR System (Thermo Fisher Scientific) using the SYBR Green Master Mix (Thermo Fisher Scientific). Equal amounts of cDNA and SYBR Green Master Mix were added along with primer pair for respective genes (AURKA—forward: TTCAGGACCTGTTAAGGCTACA, reverse: ATTTGAAGGACACAAGACCCG; AURKB—forward: ACCTGCACCATCCCAACATC, reverse: ATGATCGTGGCTGTTCGCTG; TTK—forward: CCGAGATTTGGTTGTGCCTGGA, reverse: CATCTGACACCAGAGGTTCCTTG; and β2MG—forward: GTGCTCGCGCTACTCTCTC, reverse: GTCAACTTCAATGTCGGAT). For analysis, expression was normalized to β2-microglobulin (β2MG) expression, and qPCR was carried out in technical triplicates.

### Cell Viability Assay

For the alamarBlue cell viability assay, cells were seeded in 96-well plates and treated with indicated concentrations of the compounds or vehicle. A few hours before the experimental end point (72 h, unless otherwise stated), the cells were incubated with the alamarBlue reagent. Finally, fluorescence (excitation: 550 nm and emission: 600 nm) or absorbance (570 nm with reference at 600 nm) was measured on a Tecan Spark Multiplate reader (Tecan).

## Supporting Information Available

Cellular degradation studies of azareversine-, **SF1**-, and **SF2**-based chimeras; cell viability studies of the chimeras; chemostability profile of compound **20**; synthetic procedure of all compounds; ^1^H NMR, ^13^C NMR spectra, HPLC analysis of all compounds; and uncropped images of all immunoblots (PDF)

## Abbreviations

TTK: threonine tyrosine kinase
Mps1: monopolar spindle 1
AURKs: Aurora kinases
AURKA: Aurora kinase A
AURKB: Aurora kinase B
PROTAC: proteolysis targeting chimera
VHL: von Hippel–Lindau
CRBN: cereblon
h: hour
DC_50_: half-maximal degradation concentration
*D*_max_: maximal degradation percentage
DMSO: dimethyl sulfoxide
*t*_1/2_: half-life
LC-ESI-MS: liquid chromatography-electrospray ionization-mass spectrometry
