# Supplementary material for: Development of 2-Aminoadenine-Based Proteolysis-Targeting Chimeras (PROTACs) as Novel Potent Degraders of Monopolar Spindle 1 and Aurora Kinases
Source: ACS Pharmacol Transl Sci. 2024 Oct 19;7(11):3488–501. doi: 10.1021/acsptsci.4c00405 (PMC11555526; doi:10.1021/acsptsci.4c00405)
Supplement: Supplementary file 1 — pt4c00405_si_001.pdf [file pt4c00405_si_001.pdf]

Supporting Information

Development of 2-Aminoadenine-Based Proteolysis-targeting Chimeras (PROTACs) as Novel Potent Degraders of Monopolar Spindle 1 and Aurora Kinases

Eleni Sflakidou,[a,#] Bikash Adhikari,[b,c,#] Christos Siokatas,[a] Elmar Wolf [b,c,*] and Vasiliki Sarli[a,*]

[a] Department of Chemistry, Aristotle University of Thessaloniki, University Campus, 54124, Thessaloniki, Greece

[b] Cancer Systems Biology Group, Chair of Biochemistry and Molecular Biology, Theodor Boveri Institute, University of Würzburg, 97074, Würzburg, Germany

[c] Institute of Biochemistry, University of Kiel, 24118, Kiel, Germany

[#] Both authors contributed equally to this manuscript.

Correspondence: elmar.wolf@biochem.uni-kiel.de; sarli@chem.auth.gr

CONTENTS

Supplementary Figures (Biological Part)	**4**
Synthetic procedures	**13-46**
Plasma stability	**47**
^{1}H-NMR and ^{13}C-NMR spectra for **6**	**49**
^{1}H-NMR for **MPS1 -IN-3**	**50**
^{1}H-NMR and ^{13}C-NMR spectra for **7**	**51**
^{1}H-NMR and ^{13}C-NMR spectra for **SF1**	**52**
HPLC, HRMS analysis for **SF1**	**53**
^{1}H-NMR and ^{13}C-NMR spectra for **8**	**54**
^{1}H-NMR and ^{13}C-NMR spectra for **SF2**	**55**
HPLC, HRMS analysis for **SF2**	**56**
^{1}H-NMR and ^{13}C-NMR spectra for **9**	**57**
^{1}H-NMR and ^{13}C-NMR spectra for **10**	**58**
^{1}H-NMR and ^{13}C-NMR spectra for **11**	**59**
^{1}H-NMR and ^{13}C-NMR spectra for **13**	**60**
HPLC, HRMS analysis for **13**	**61**
^{1}H-NMR and ^{13}C-NMR spectra for **14**	**62**
HPLC, HRMS analysis for **14**	**63**
^{1}H-NMR and ^{13}C-NMR spectra for **15**	**64**
HPLC, HRMS analysis for **15**	**65**
^{1}H-NMR and ^{13}C-NMR spectra for **17**	**66**
HPLC, HRMS analysis for **17**	**67**

^{1}H-NMR and ^{13}C-NMR spectra for **18**	**68**
HPLC, HRMS analysis for **18**	**69**
^{1}H-NMR and ^{13}C-NMR spectra for **19**	**70**
HPLC, HRMS analysis for **19**	**71**
^{1}H-NMR and ^{13}C-NMR spectra for **20**	**72**
HPLC, HRMS analysis for **20**	**73**
^{1}H-NMR and ^{13}C-NMR spectra for **21**	**74**
HPLC, HRMS analysis for **21**	**75**
^{1}H-NMR and ^{13}C-NMR spectra for **22**	**76**
HRMS analysis for **22**	**77**
^{1}H-NMR and ^{13}C-NMR spectra for **23**	**78**
HPLC, HRMS analysis for **23**	**79**
^{1}H-NMR and ^{13}C-NMR spectra for **24**	**80**
HPLC, HRMS analysis for **24**	**81**
^{1}H-NMR and ^{13}C-NMR spectra for **26**	**82**
HPLC, HRMS analysis for **26**	**83**
^{1}H-NMR and ^{13}C-NMR spectra for **27**	**84**
HPLC, HRMS analysis for **27**	**85**
^{1}H-NMR and ^{13}C-NMR spectra for **28**	**86**
HPLC, HRMS analysis for **28**	**87**
^{1}H-NMR and ^{13}C-NMR spectra for **29**	**88**
^{1}H-NMR and ^{13}C-NMR spectra for **30**	**89**
HPLC, HRMS analysis for **30**	**90**
HRMS analysis for **S8, 31**	**91**
^{1}H-NMR and ^{13}C-NMR spectra for **32**	**92**
HRMS analysis for **32**	**93**
^{1}H-NMR and ^{13}C-NMR spectra for **34**	**94**
HPLC, HRMS analysis for **34**	**95**
^{1}H-NMR and ^{13}C-NMR spectra for **35**	**96**
HPLC, HRMS analysis for **35**	**97**
^{1}H-NMR and ^{13}C-NMR spectra for **36**	**98**
HPLC, HRMS analysis for **36**	**99**
^{1}H-NMR and ^{13}C-NMR spectra for **38**	**100**
HPLC, HRMS analysis for **38**	**101**
^{1}H-NMR and ^{13}C-NMR spectra for **40**	**102**
HPLC, HRMS analysis for **40**	**103**
^{1}H-NMR and ^{13}C-NMR spectra for **41**	**104**
HPLC, HRMS analysis for **41**	**105**

^{1}H-NMR and ^{13}C-NMR spectra for **43**	**106**
HPLC, HRMS analysis for **43**	**107**
^{1}H-NMR and ^{13}C-NMR spectra for **44**	**108**
HPLC, HRMS analysis for **44**	**109**
^{1}H-NMR and ^{13}C-NMR spectra for **46**	**110**
HPLC, HRMS analysis for **46**	**111**
^{1}H-NMR and ^{13}C-NMR spectra for **S16**	**112**
ESI-MS analysis for **S16**	**113**
^{1}H-NMR and ^{13}C-NMR spectra for **47**	**114**
HPLC, HRMS analysis for **47**	**115**
^{1}H-NMR and ^{13}C-NMR spectra for **49**	**116**
HPLC, HRMS analysis for **49**	**117**
^{1}H-NMR and ^{13}C-NMR spectra for **S17**	**118**
HPLC, HRMS analysis for **S17**	**119**
^{1}H-NMR and ^{13}C-NMR spectra for **51**	**120**
HPLC, HRMS analysis for **51**	**121**
^{1}H-NMR and ^{13}C-NMR spectra for **52**	**122**
ESI-MS analysis for **52**	**123**
^{1}H-NMR and ^{13}C-NMR spectra for **53**	**124**
ESI-MS analysis for **53**	**125**
^{1}H-NMR and ^{13}C-NMR spectra for **55**	**126**
HPLC, HRMS analysis for **55**	**127**
^{1}H-NMR and ^{13}C-NMR spectra for **54**	**128**
HRMS analysis for **54**	**129**
^{1}H-NMR and ^{13}C-NMR spectra for **56**	**130**
HRMS analysis for **56**	**131**
Analytical methods for LC-MS / HPLC	**132**

Supplementary Figures (Biological Part)

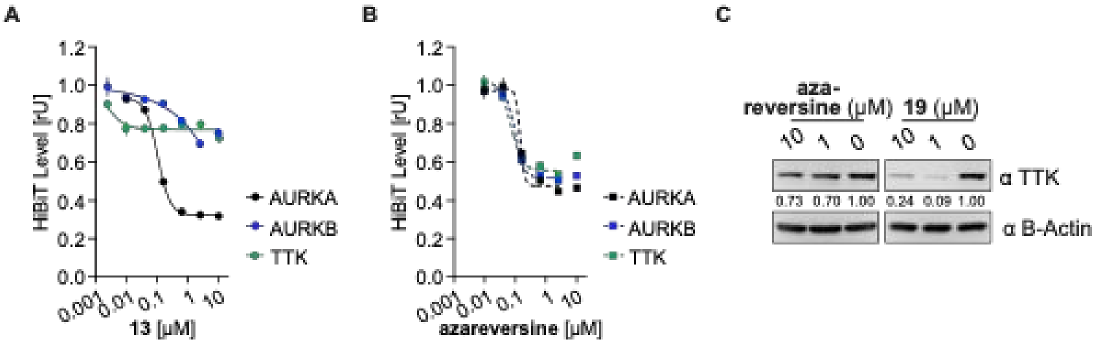

Uncropped original immunoblots

Figure S1C

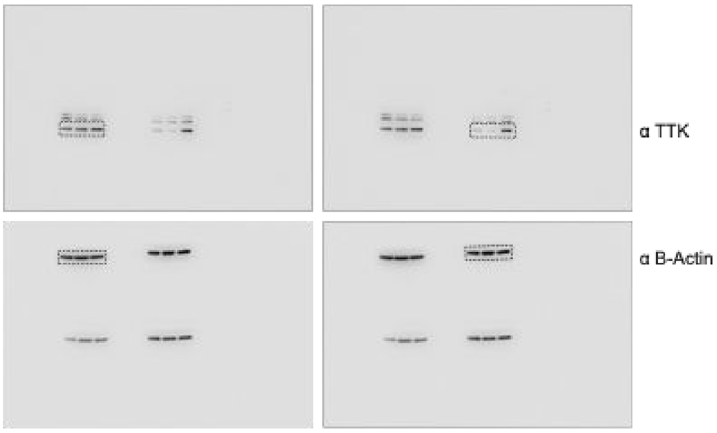

Figure S1: Cellular degradation studies of azareversine-based compounds. (A-B) AURKA, AURKB and TTK levels based on luciferase measurements. Indicated MV4-11 HiBiT cells were treated with different concentrations of **13** (A) and **azareversine** (B) for 6 h, lysed, complemented with the large luciferase fragment (largeBiT), and luciferase activity was measured. The data is represented as a mean ±s.d. from n=3 replicates. **(C)** Immunoblot of TTK. AURKA-HiBiT cells were treated with indicated concentration of azareversine and **19** for 6 h and compared with vehicle treated cells.

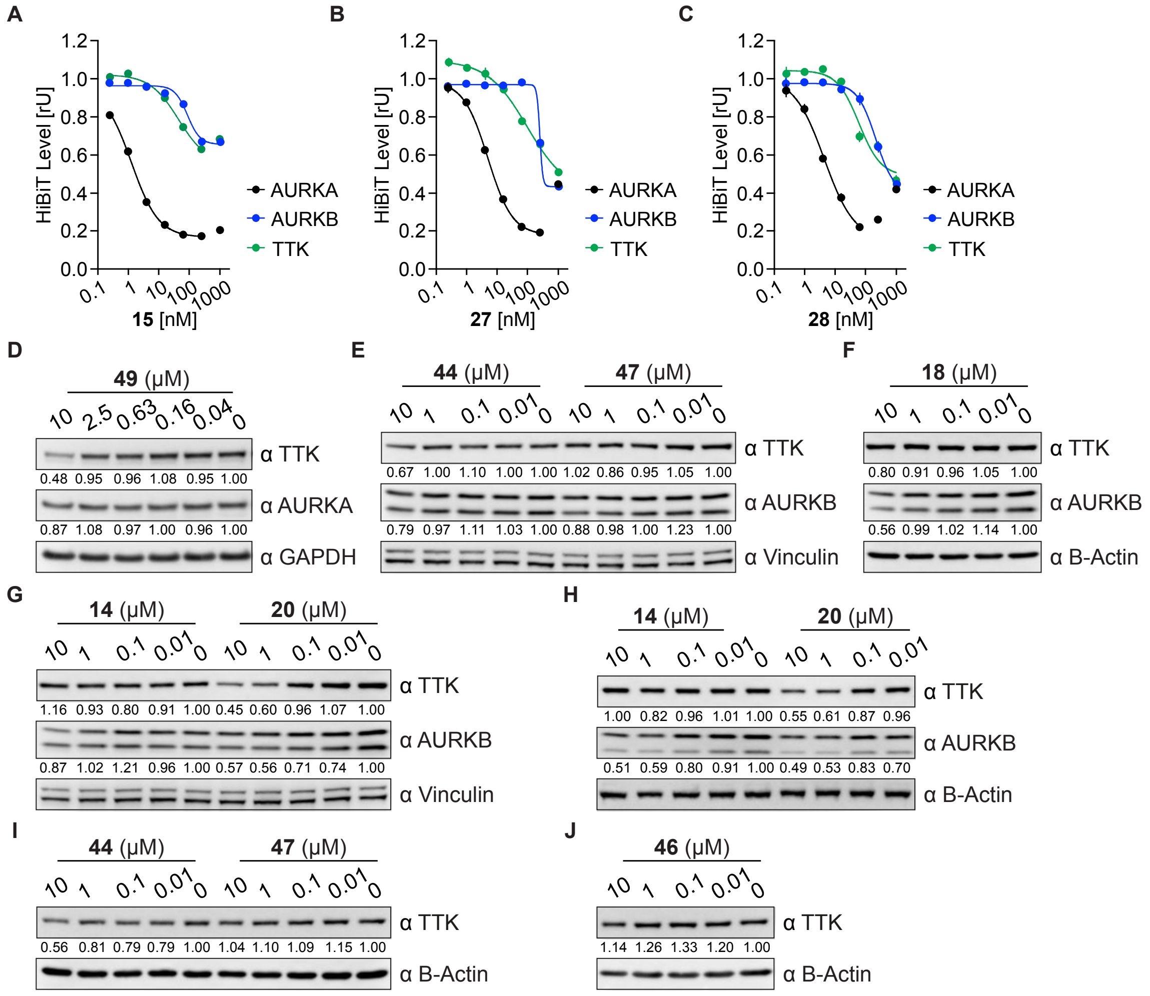

Figure S2: Cellular degradation studies of SF1-based compounds. (A-C) AURKA, AURKB and TTK levels based on luciferase measurements. Indicated MV4-11 HiBiT cells were treated with different concentrations of **15** (A), **27** (B), and **28** (C) for 6 h, lysed, complemented with the large luciferase fragment (largeBiT), and luciferase activity was measured. **(D)** Immunoblots of AURKA and TTK. HiBiT-TTK cells were treated with different concentration of **49** for 6 h and compared with vehicle treated cells. GAPDH was used as a loading control. **(E-G)** Immunoblots of TTK and AURKB. Naive MV4-11 cells were treated with indicated concentration of **44** or **47** (E)**, 18** (F), and **14** or **20** (G) for 6 h and compared with vehicle treated cells. **(H-J)** Immunoblots of TTK and AURKB. CALU1 cells were treated with indicated concentration of **14** or **20** (H)**, 44** or **47** (I), and **46** (J) for 6 h and compared with DMSO treated cells. All HiBiT data are represented as a mean ±s.d. from n=2 replicates.

Uncropped original immunoblots related to Figure S2

Figure S2D

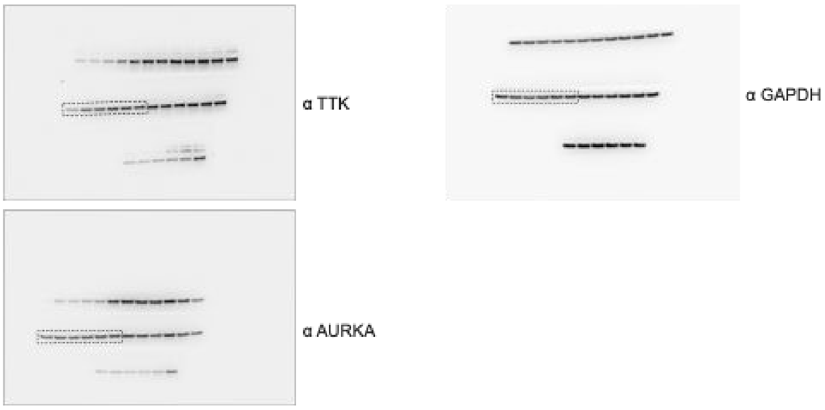

Figure S2E

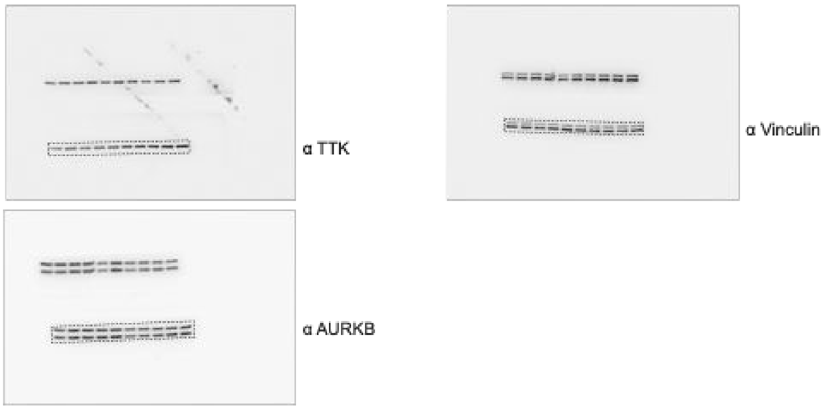

Figure S2F

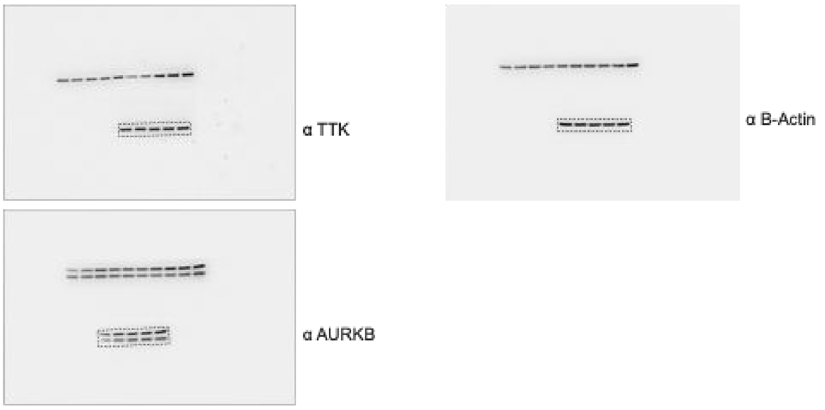

Figure S2G

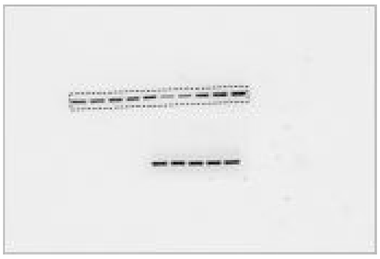

α TTK

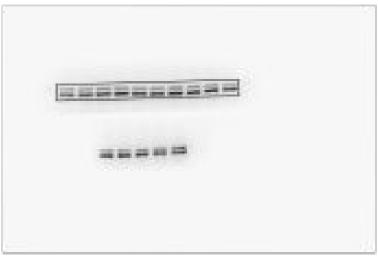

α Vinculin

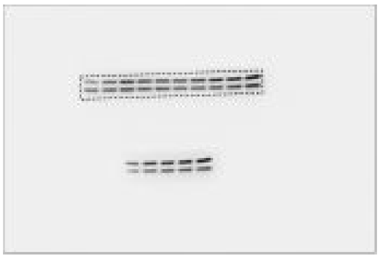

α AURKB

Figure S2H

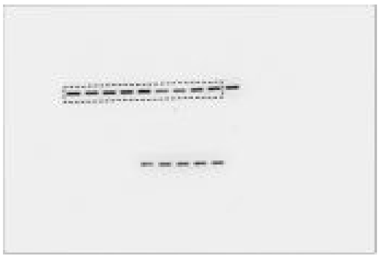

α TTK

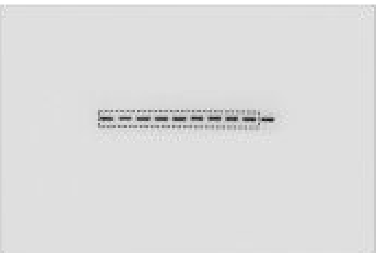

α B-Actin

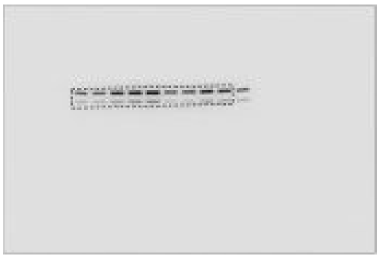

α AURKB

Figure S2I

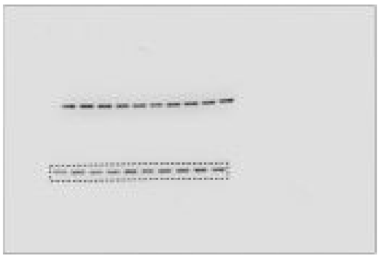

α TTK

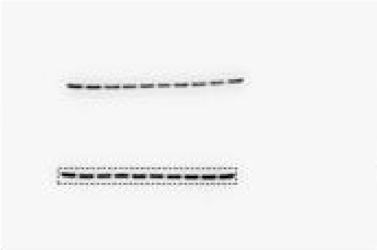

α B-Actin

Figure S2J

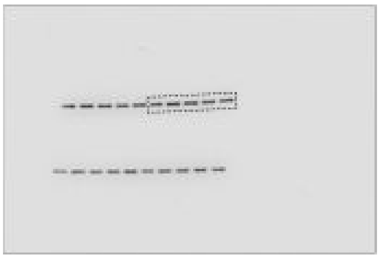

α TTK

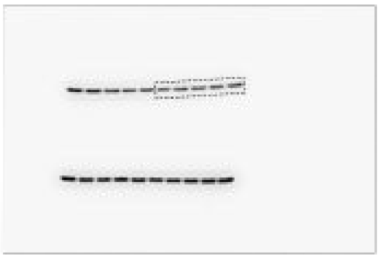

α B-Actin

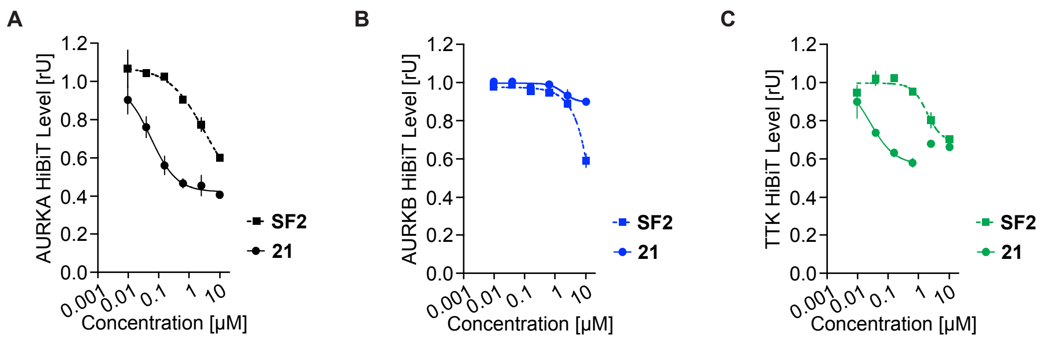

Figure S3: Cellular degradation studies of SF2-based compounds. (A-C) AURKA, AURKB and TTK levels based on luciferase measurements. AURKA- HiBiT (A), AURKB-HiBiT (B) and HiBiT-TTK (C) cells were treated with various concentrations of ligand **SF2** or chimera **21** for 6 h, lysed, complemented with the large luciferase fragment (largeBiT), and luciferase activity was measured. All HiBiT data are represented as a mean ±s.d. from n=2 replicates.

Figure S4. Uncropped original immunoblots related to Figure 4 of the main text

Figure 4A

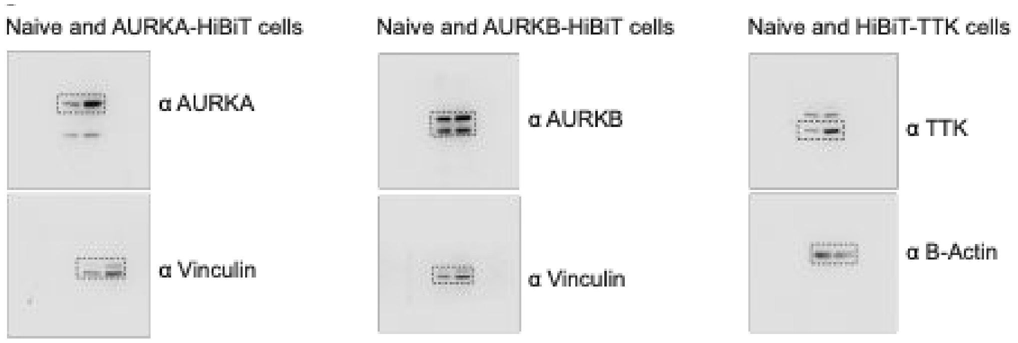

Figure 4D

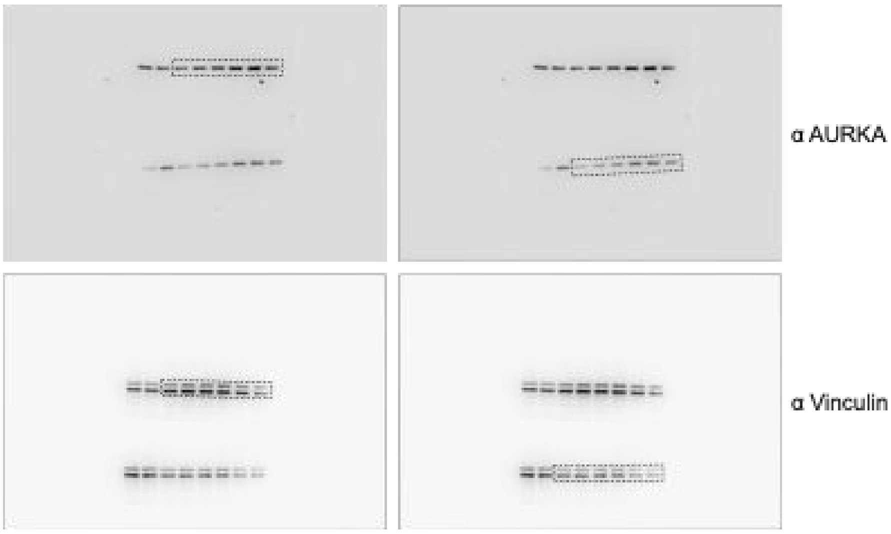

Figure 4E

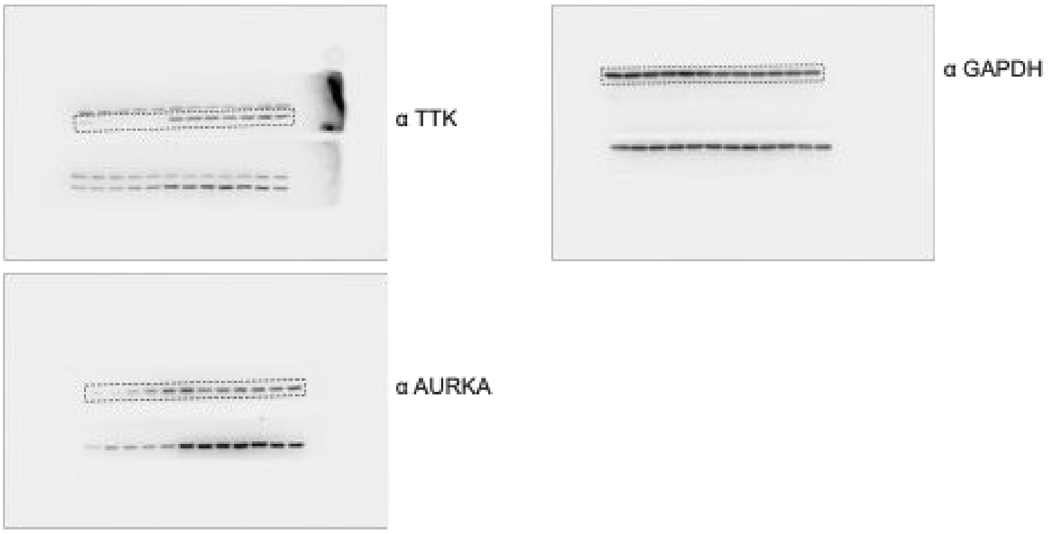

Figure S5. Uncropped original immunoblots related to Figure 5 of the main text

Figure 5C

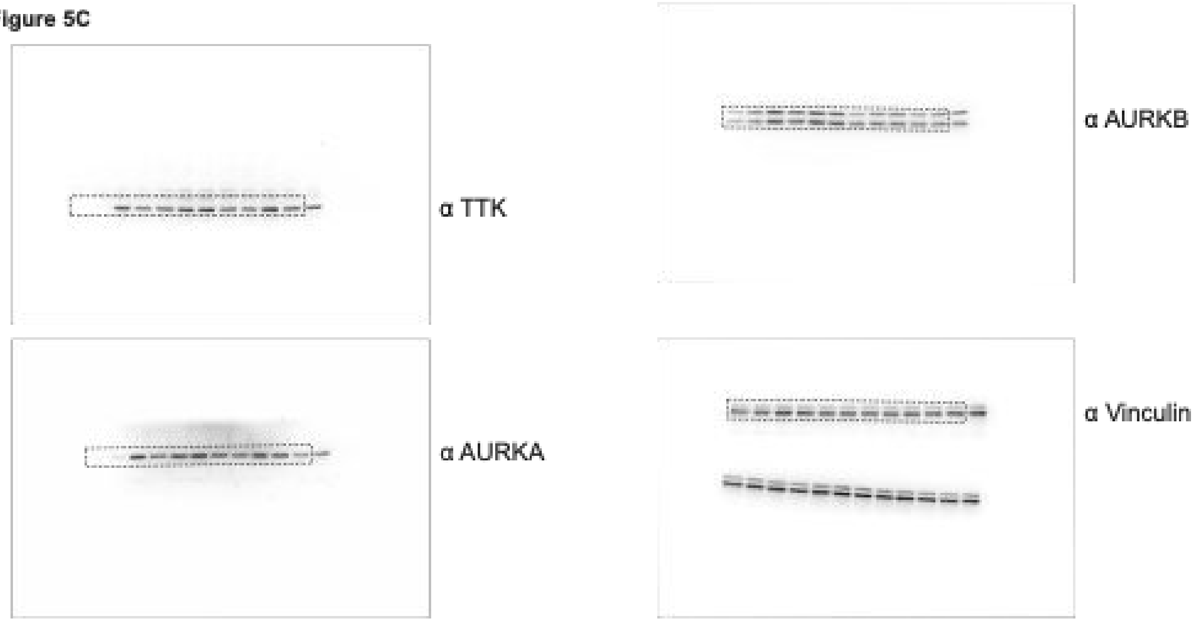

Figure 5D

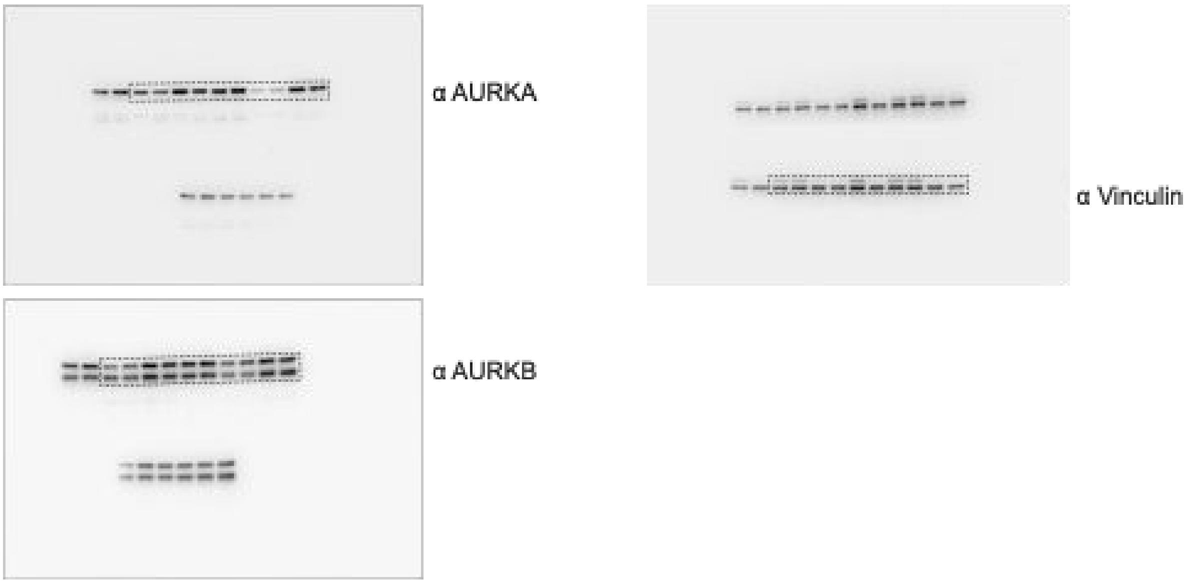

Figure 5E

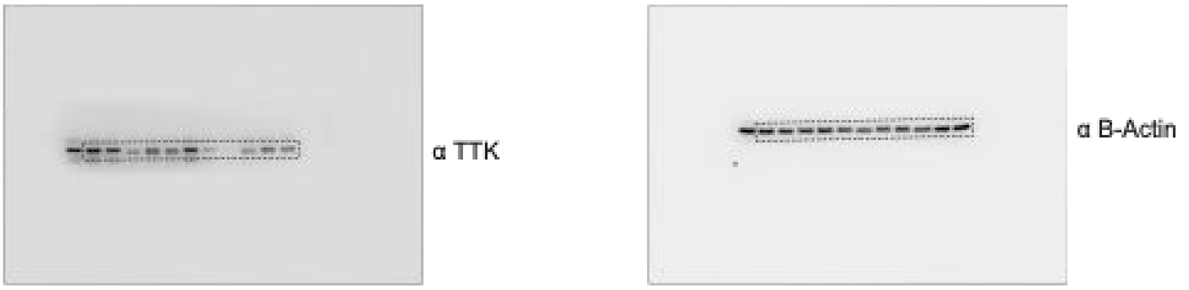

Figure S6 Uncropped original immunoblots related to Figure 6 of the main text

Figure 6D

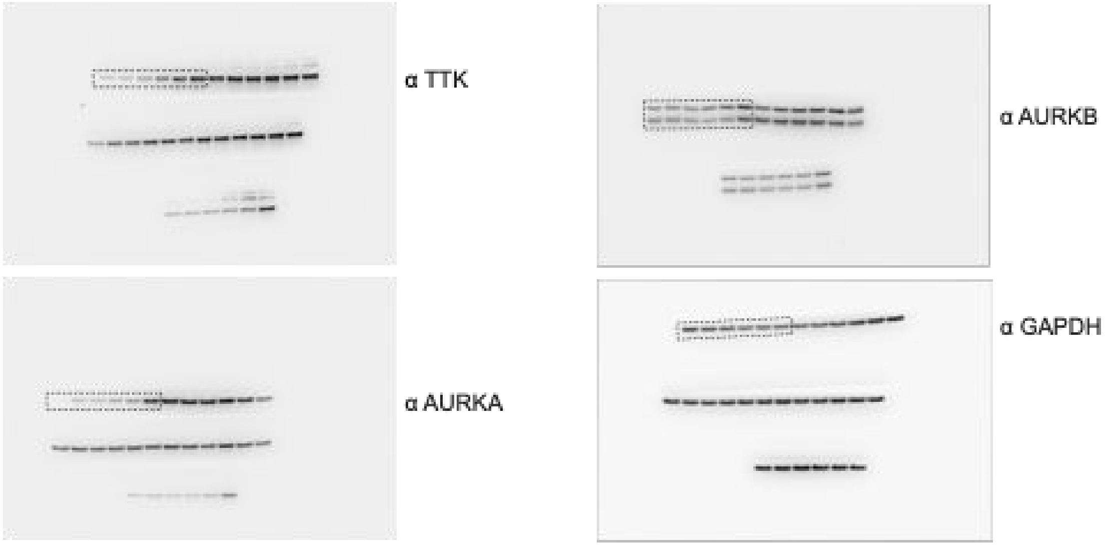

Figure 6E

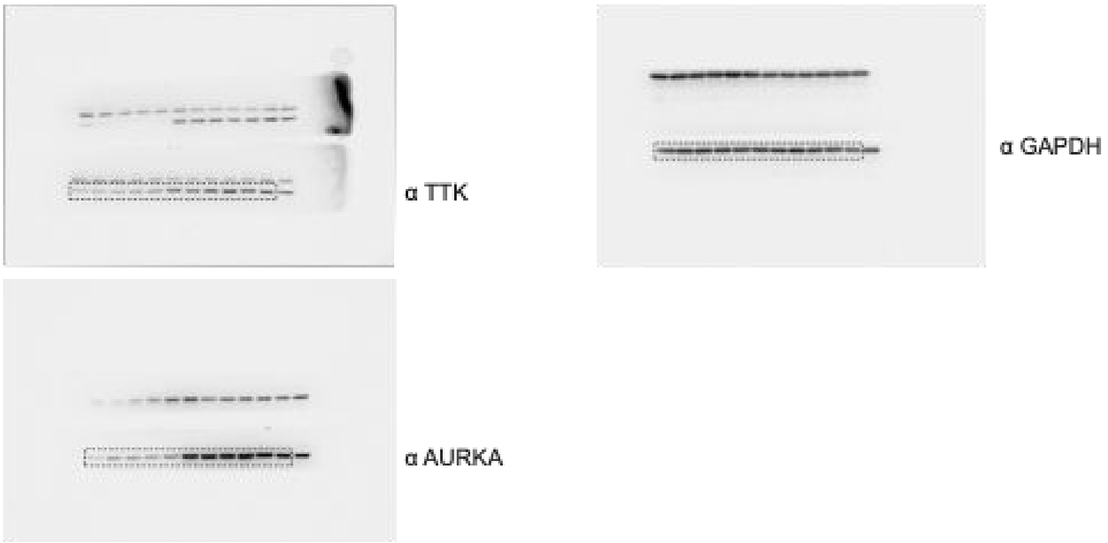

Figure 6F

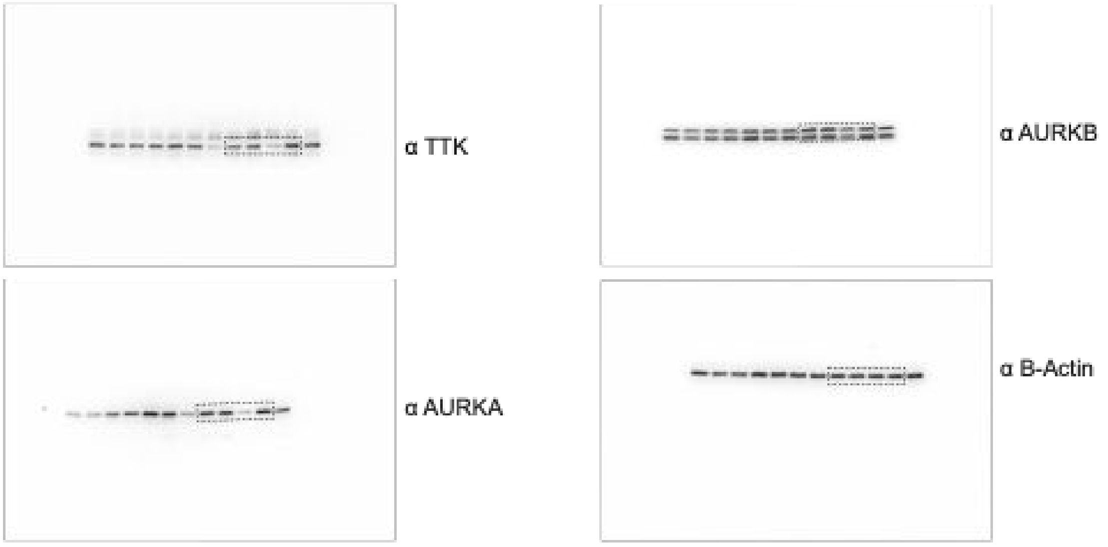

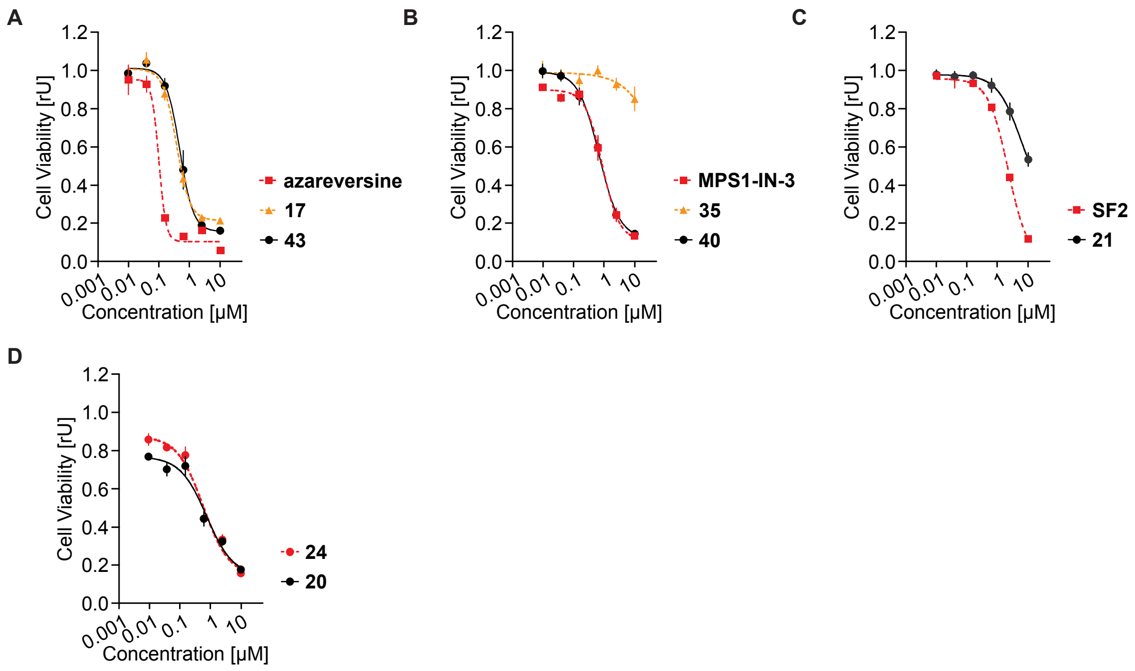

Figure S7: Cell viability studies of the chimeras. (A) Cell viability measurement of azareversine-based compounds. MV4-11 cells were incubated with various concentrations of **azareversine**, **17**, or **43** for 72 h and compared. **(B)** Cell viability measurement of **MPS1-IN-3**-based compounds. MV4-11 cells were incubated with indicated concentrations of **MPS1-IN-3**, **35**, or **40** for 72 h. **(C)** Cell viability measurement of **SF2**-based compounds. MV4-11 cells were incubated with different concentrations of **SF2**, or **21** for 72 h. **(D)** Cell viability measurement of **20**. MV4-11 cells were incubated with various concentrations of **20**, or its negative control **24** for 72 h. Data are represented as a mean and ±s.d. from n=3 replicates.

2-chloro-*N*-(2-(isopropylsulfonyl)phenyl)-9-(tetrahydro-2*H*-pyran-2-yl)-9*H*-purin-6-amine, 4: The synthesis of intermediate 2-chloro-*N*-(2-(isopropylsulfonyl)phenyl)-9-(tetrahydro-2*H*-pyran-2-yl)-9*H*-purin-6-amine **4** was carried out according to Tannous et al.[1]

1-(4-amino-3-methoxy phenyl)piperidin-4-ol, 5a and ***tert*-butyl 4-(4-amino-3-methoxyphenyl)piperazine-1-carboxylate, 5b** and**:** The synthesis of *tert*-butyl 4-(4-amino-3-methoxyphenyl)piperazine-1-carboxylate **5b** and 1-(4-amino-3-methoxyphenyl)piperidin-4-ol **5a** was carried out according to Lu et al.[2]

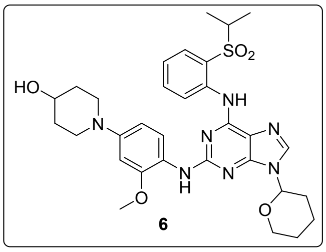

1-(4-((6-((2-(isopropylsulfonyl)phenyl)amino)-9-(tetrahydro-2*H*-pyran-2-yl)-9*H*-purin-2-yl)amino)-3-methoxyphenyl)piperidin-4-ol, 6: In a self-sealing vial 2-chloro-*N*-(2-(isopropylsulfonyl)phenyl)-9-(tetrahydro-2*H*-pyran-2-yl)-9*H*-purin-6-amine (100 mg, 0.229 mmol) was dissolved in dry toluene (2.5 mL). To the mixture 1-(4-amino-3-methoxyphenyl)piperidin-4-ol (56 mg, 0.252 mmol), caesium carbonate (300 mg, 0.917 mmol), (±)-2,2′-bis(diphenylphosphino)-1,1′-binaphthalene (30 mg, 0.057 mmol) and palladium acetate (7.5 mg, 0.038 mmol) were added under Ar atmosphere. Afterwards the system was degassed, and the reaction mixture was stirred for 16 h at 110 °C. Then, the mixture was cooled to room temperature, dissolved with EtOAc and washed with water. The organic layer was dried over anhydrous Na_2SO_4, filtered and the solvents were removed under reduced pressure. The crude product was purified by flash chromatography (eluent; dichloromethane/acetone, 6/1) to afford **6** as an off-white solid in 77% yield (110 mg).[1] **6**: ^{1}H NMR (500 MHz, $CDCl_3$) δ 9.95 (s, 1H), 8.86 (d, *J* = 8.4 Hz, 1H), 8.31 (d, *J* = 8.6 Hz, 1H), 7.89 (d, *J* = 8.4 Hz, 1H), 7.86 (s, 1H), 7.62 (t, *J* = 7.5 Hz, 1H), 7.34 (s, 1H), 7.19 (dd, *J* = 7.5 Hz, 1H), 6.60 (s, 1H), 6.58 (d, *J* = 8.5 Hz, 1H), 5.62 (m, 1H), 4.18 (d, *J* = 12.9 Hz, 1H), 3.91 (s, 3H), 3.89 – 3.84 (m, 1H), 3.81 – 3.74 (m, 2H), 3.53 – 3.47 (m, 2H), 3.28 (m, 1H), 2.96 – 2.88 (m, 2H), 2.08 (m, 4H), 1.83 – 1.70 (m, 4H), 1.66 (d, *J* = 7.4 Hz, 1H), 1.47 (d, *J* = 4.4 Hz, 1H), 1.31 (d, *J* = 3.1 Hz, 6H); ^{13}C NMR (126 MHz, $CDCl_3$) δ 156.4, 151.5, 151.4, 149.1, 147.14 139.5, 137.2, 134.7, 131.3, 123.6, 123.1, 123.0, 122.3, 120.0, 116.3, 108.7, 101.4, 81.7, 68.7, 67.9, 55.9, 55.8, 53.9, 48.8, 34.6, 31.5, 29.4, 25.1, 23.1, 15.6.

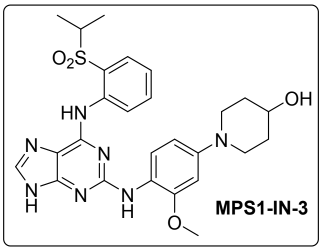

1-(4-((6-((2-(isopropylsulfonyl)phenyl)amino)-9*H*-purin-2-yl)amino)-3-methoxyphenyl)piperidin-4-ol, MPS1 -IN-3: To a solution of 1-(4-((6-((2-(isopropylsulfonyl)phenyl)amino)-9-(tetrahydro-2*H*-pyran-2-yl)-9*H*-purin-2-yl)amino)-3-methoxyphenyl)piperidin-4-ol (20 mg, 0.032 mmol) in ethanol (0.5 mL), 4N HCl in dioxane (0.3 mL) was added. The reaction mixture was stirred at room temperature for 1 hr. After removal of solvent, the residue was dissolved in ethyl acetate and washed with aqueous $NaHCO_3$ solution and brine. The organic phase was dried over Na_2SO_4 and the solvent was evaporated to afford 15 mg of the MPS1-IN-3 inhibitor (90% yield) without further purification.[1] ^{1}H NMR (500 MHz, $CDCl_3$) δ 11.68 (br, 1H), 10.01 (s, 1H), 8.89 (d, *J* = 8.3 Hz, 1H),

7.94 (d, J = 8.3 Hz, 1H), 7.89 (d, J = 7.8 Hz, 1H), 7.64 (dd, J = 8.0, 7.6 Hz, 1H), 7.23 – 7.17 (m, 2H), 7.02 (br, 1H), 6.61 (br, 1H), 6.52 (br, 1H), 3.90 (m, 4H), 3.85 (m, 2H), 3.57 – 3.56 (s, 1H), 3.28 (m, 2H), 2.96 – 2.94 (m, 2H), 2.03 – 1.97 (m, 1H), 1.71 (m, 2H), 1.31 (d, J=6.8Hz, 6H).

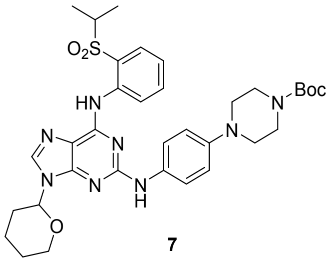

7

***tert*-butyl 4-(4-((6-((2-(isopropylsulfonyl)phenyl)amino)-9-(tetrahydro-2*H*-pyran-2-yl)-9*H*-purin-2-yl)amino)phenyl)piperazine-1-carboxylate, 7**: In a self-sealing vial 2-chloro-N-(2-(isopropylsulfonyl)phenyl)-9-(tetrahydro-2*H*-pyran-2-yl)-9*H*-purin-6-amine **4** (77 mg, 0.177 mmol) was dissolved in dry toluene (2 mL). To the *tert*-butyl 4-(4-aminophenyl)piperazine-1-carboxylate (53 mg, 0.194 mmol), caesium carbonate (230 mg, 0.706 mmol), (±)-2,2′-bis(diphenylphosphino)-1,1′-binaphthalene (27 mg, 0.043 mmol) and palladium acetate (5.7 mg, 0.025 mmol) were added under Ar atmosphere. Afterwards the system was degassed, and the reaction mixture was stirred for 16h at 110 °C. Then, the mixture was cooled to room temperature, dissolved with EtOAc and washed with water. The organic layer was dried over anhydrous Na_2SO_4, filtered and the solvents were removed in rotary. The crude product was purified by flash chromatography (eluent; dichloromethane/acetone, 8/1) to afford **7** as an off-white solid in 87% yield (104 mg). **7**: ^{1}H NMR (500 MHz, $CDCl_3$) δ 10.01 (s, 1H), 8.81 (d, J = 8.4 Hz, 1H), 7.87 (m, 2H), 7.55 – 7.51 (m, 3H), 7.17 (dd, J = 7.7, 7.5 Hz, 1H), 6.94 (m, 3H), 5.58 (d, J = 10.3 Hz, 1H), 4.16 (d, J = 11.0 Hz, 1H), 3.76 (dd, J = 11.0, 10.8 Hz, 1H), 3.61 (s, 4H), 3.26 (m, 1H), 3.10 (s, 4H), 2.13 – 2.04 (m, 2H), 1.81 – 1.70 (m, 2H), 1.69 – 1.57 (m, 2H), 1.49 (s, 9H), 1.30 (d, J = 6.2 Hz, 6H); ^{13}C NMR (126 MHz, $CDCl_3$) δ 156.3, 154.7, 151.4, 151.1, 146.8, 139.3, 137.2, 134.5, 133.5, 131.2, 123.3, 122.6, 121.1, 117.1, 117.5, 116.3, 81.6, 79.9, 68.6, 55.9, 50.4, 31.3, 29.3, 24.9, 22.9, 15.4; ESI-HRMS m/z for $C_{34}H_{45}N_8O_5S$ [M+H]+ calcd 677.3234, found 677.3215.

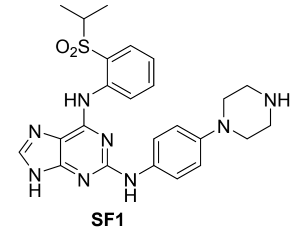

SF1

***N*^{6}-(2-(isopropylsulfonyl)phenyl)-*N*^{2}-(4-(piperazin-1-yl)phenyl)-9*H*-purine-2,6-diamine, SF1:** To a solution of *tert*-butyl 4-(4-((6-((2-(isopropylsulfonyl)phenyl)amino)-9-(tetrahydro-2*H*-pyran-2-yl)-9*H*-purin-2-yl)amino)phenyl)piperazine-1-carboxylate, **7** (50 mg, 0.074 mmol) in ethanol (1.1 mL), 4N HCl in dioxane (0.7 mL) was added. The reaction mixture was stirred at room temperature for 1 hr. After removal of solvent, the residue was dissolved in ethyl acetate and washed with aqueous $NaHCO_3$ solution and brine. The organic phase was dried over Na_2SO_4 and the solvent was evaporated to afford 32 mg of the **SF-1** (90% yield) without further purification. **SF-1:** ^{1}H NMR (300 MHz, DMSO-*d*6) δ 9.85 (s, 1H), 9.06 (d, J = 7.7 Hz, 2H), 7.97 (s, 1H), 7.82 (d, J = 7.9 Hz, 1H), 7.73 (m, 1H), 7.61 (d, J = 8.6 Hz, 2H), 7.29 (m, 1H), 6.92 (d, J = 8.7 Hz, 2H), 3.17 (m, 4H), 3.07 (m, 6H), 1.19 (d, J = 6.7 Hz, 6H) 2 protons missing due to overlapping with solvent peaks; ^{13}C NMR (75 MHz, DMSO-*d*6) δ 156.1, 152.3, 150.5, 145.6, 139.3, 138.4, 135.1, 133.9, 130.9, 122.3, 122.2, 122.1, 120.5, 116.4, 114.9, 55.2, 48.0, 43.9, 14.9; ESI-HRMS m/z for $C_{24}H_{29}N_8O_2S$ [M+H]$^+$ calcd 493.2134, found 493.2129.

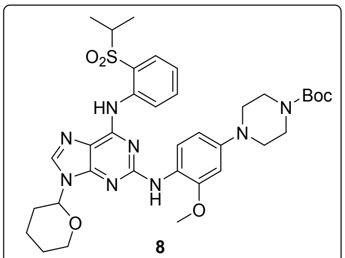

8

***tert*-butyl 4-(4-((6-((2-(isopropylsulfonyl)phenyl)amino)-9-(tetrahydro-2*H*-pyran-2-yl)-9*H*-purin-2-yl)amino)-3-methoxyphenyl)piperazine-1-carboxylate**, **8**: In a self-sealing vial 2-chloro-*N*-(2-(isopropylsulfonyl)phenyl)-9-(tetrahydro-2*H*-pyran-2-yl)-9*H*-purin-6-amine (100 mg, 0.229 mmol) was dissolved in dry toluene (2.5 mL). To the mixture *tert*-butyl 4-(4-amino-3-methoxyphenyl)piperazine-1-carboxylate (78 mg, 0.252 mmol), caesium carbonate (300 mg, 0.917 mmol), (±)-2,2′-bis(diphenylphosphino)-1,1′-binaphthalene (30 mg, 0.057 mmol) and palladium acetate (7.5 mg, 0.038 mmol) were added under Ar atmosphere. Afterwards the system was degassed and the reaction mixture was stirred for 16h at 110 °C. Then the mixture was cooled to room temperature, dissolved with EtOAc and washed with water. The organic layer was dried over anhydrous Na_2SO_4, filtered and the solvents were removed in rotary. The crude product was purified by flash chromatography (eluent; dichloromethane/acetone, 12/1) to afford the THP and Boc-protected **SF-2** a yellow foam in 70% yield (113 mg). **8**: ^{1}H NMR (500 MHz, $CDCl_3$) δ 9.95 (s, 1H), 8.84 (d, *J* = 8.4 Hz, 1H), 8.34 (d, *J* = 8.7 Hz, 1H), 7.88 (d, *J* = 7.9 Hz, 1H), 7.86 (s, 1H), 7.62 (dd, *J* = 8.4, 7.4 Hz, 1H), 7.36 (s, 1H), 7.19 (dd, *J* = 7.6 Hz, 1H), 6.58 (s, 1H), 6.55 (d, *J* = 8.8 Hz, 1H), 5.61 (d, *J* = 9.6 Hz, 1H), 4.17 (d, *J* = 12.6 Hz, 1H), 3.91 (s, 3H), 3.78 (t, *J* = 10.5 Hz, 1H), 3.66 – 3.54 (m, 4H), 3.27 (m, 1H), 3.10 (s, 4H), 2.20 – 2.13 (m, 1H), 2.09 (m, 2H), 1.84 – 1.71 (m, 2H), 1.69 – 1.61 (m, 1H), 1.49 (s, 9H), 1.30 (d, *J* = 3.5 Hz, 3H), 1.32 (d, *J* =3.4 Hz, 3H); ^{13}C NMR (126 MHz, $CDCl_3$) δ 156.2 154.7, 151.3, 151.2, 148.9, 146.8, 139.4, 137.2, 134.6, 131.2, 123.7, 123.5, 122.8, 122.2, 119.7, 116.3, 108.6, 101.3, 81.6, 79.9, 69.8, 68.7, 55.8, 55.7, 50.8, 31.3, 28.5, 24.9, 23.0, 15.5, 15.4; ESI-HRMS m/z for $C_{35}H_{47}N_8O_6S$ $[M+H]^+$ calcd 707.3339, found 707.3323.

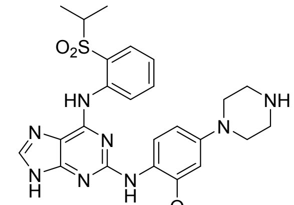

SF2

***N*^{6}-(2-(isopropylsulfonyl)phenyl)-*N*^{2}-(2-methoxy-4-(piperazin-1-yl)phenyl)-9*H*-purine-2,6-diamine, SF-2**: To a solution of tert-butyl 4-(4-((6-((2-(isopropylsulfonyl)phenyl)amino)-9-(tetrahydro-2*H*-pyran-2-yl)-9*H*-purin-2-yl)amino)-3-methoxyphenyl)piperazine-1-carboxylate, **8** (50 mg, 0.071 mmol) in ethanol (1.1 mL), 4N HCl in dioxane (0.7 mL) was added. The reaction mixture was stirred at room temperature for 1 hr. After removal of solvent, the residue was dissolved in ethyl acetate and washed with aqueous $NaHCO_3$ solution and brine. The organic phase was dried over Na_2SO_4, and the solvent was evaporated to afford 33 mg of the **SF-2** derivative (90% yield) without further purification. **SF-2**: ^{1}H NMR (500 MHz, DMSO-*d*6) δ 12.70 (s, 1H), 9.83 (s, 1H), 8.94 (d, *J* = 8.4 Hz, 1H), 8.24 (s, 1H), 7.94 (s, 1H), 7.80 (m, 2H), 7.66 (m, 2H), 7.26 (dd, *J* = 7.7, 7.6 Hz, 1H), 6.64 (d, *J* = 2.2 Hz, 1H), 6.49 (dd, *J* = 8.7, 2.2 Hz, 1H), 3.79 (s, 3H), 3.12 – 3.06 (m, 4H), 3.03 – 2.96 (m, 1H), 2.93 – 2.87 (m, 4H), 1.18 (d, *J* = 6.8 Hz, 6H); ^{13}C NMR (75 MHz, DMSO-*d*6) δ 156.9, 152.5, 151.6, 150.5, 148.2, 139.2, 138.3, 135.0, 130.9, 123.7, 122.2, 122.1, 122.0, 121.6, 114.9, 107.0, 100.5, 55.5, 55.2, 48.7, 44.6, 14.9; ESI-HRMS m/z for $C_{25}H_{32}N_8O_3S$ $[M+H]^+$ calcd 523.2240, found 523.2230.

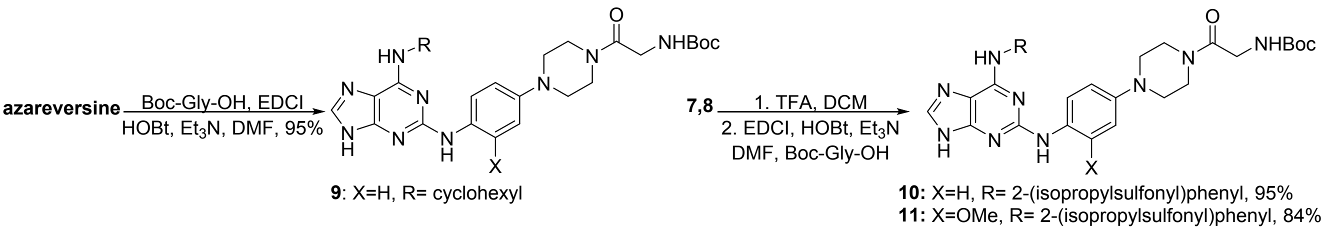

Scheme S1. Linker installation on azareversine, **7** and **8**.

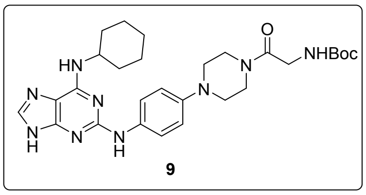

***tert*-butyl (2-(4-(4-((6-(cyclohexylamino)-9*H*-purin-2-yl)amino)phenyl)piperazin-1-yl)-2-oxoethyl)carbamate, 9**: In a dried flask Boc-Gly-OH (8 mg, 0.046 mmol) was dissolved in dry DMF (0.9 mL) and azareversine (20 mg, 0.051 mmol), HOBt (9 mg, 0.068 mmol), EDCI (13 mg, 0.068 mmol) and triethylamine (14 mg, 0.137 mmol) were added under Ar atmosphere. The reaction mixture was stirred for 18h at ambient temperature and upon completion of the reaction the solvent was removed under reduced pressure. The crude mixture was purified by flash chromatography (eluent; 3% MeOH in DCM) to afford the product as a beige solid (24 mg, yield 95%). **9**: ^{1}H NMR (500 MHz, $CDCl_3$) δ 7.43 (d, *J* = 8.3 Hz, 2H), 7.03 (br, 1H), 6.84 (d, *J* = 8.2 Hz, 2H), 6.72 (br, 1H), 5.74 (br, 1H), 5.69 (s, 1H), 4.04 (s, 1H), 3.99 (d, *J* = 4.1 Hz, 2H), 3.74 (br, 2H), 3.47 (br, 2H), 3.04 (s, 4H), 2.05 (d, *J* = 9.8 Hz, 2H), 1.79 – 1.71 (m, 2H), 1.67 – 1.59 (m, 1H), 1.43 (s, 9H), 1.39 – 1.32 (m, 2H), 1.31 – 1.15 (m, 4H); ^{13}C NMR (126 MHz, $CDCl_3$) δ 167.0, 156.9, 155.9, 154.2, 150.3, 146.8, 135.7, 133.5, 122.6, 117.9, 114.3, 79.7, 50.2, 50.0, 49.2, 44.2, 42.2, 41.9, 33.1, 28.3, 25.6, 24.9.

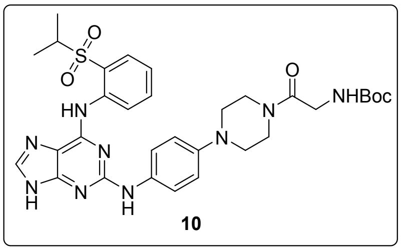

***tert*-butyl (2-(4-(4-((6-((2-(isopropylsulfonyl)phenyl)amino)-9*H*-purin-2-yl)amino)phenyl)piperazin-1-yl)-2-oxoethyl)carbamate, 10:** *tert*-butyl 4-(4-((6-((2-(isopropylsulfonyl)phenyl)amino)-9-(tetrahydro-2*H*-pyran-2-yl)-9*H*-purin-2-yl)amino)phenyl)piperazine-1-carboxylate (27 mg, 0.039 mmol) **7** was dissolved in a mixture of dichloromethane/TFA 3/1 (0.6 mL) and the solution was stirred for 2h at ambient temperature. Afterwards the solvents were removed *in vacuo* to afford the desired amine salt, which was used in the next step without further purification. In a dried flask, Boc-glycine (8 mg, 0.046 mmol) was dissolved in dry DMF (0.9 mL) and 4-(4-((6-((2-(isopropylsulfonyl)phenyl)amino)-9*H*-purin-2-yl)amino)phenyl)piperazin-1-ium 2,2,2-trifluoroacetate (20 mg, 0.051 mmol), HOBt (9 mg, 0.068 mmol), EDCI (13 mg, 0.068 mmol) and triethylamine (14 mg, 0.137 mmol) were added under Ar atmosphere. The reaction mixture was stirred for 18 h at ambient temperature and upon completion of the reaction the solvent was removed under reduced pressure. The crude mixture was purified by flash chromatography (eluent; 3% MeOH in DCM) to afford **10** as a beige solid (24 mg, yield 95%). **10:** ^{1}H NMR (500 MHz, $CDCl_3$) δ 10.07 (s, 1H), 8.85 (d, *J* = 8.5 Hz, 1H), 7.86 (d, *J* = 7.8 Hz, 1H), 7.58 (dd, *J* =8.5, 7.4 Hz, 1H), 7.49 – 7.43 (m, 2H), 7.18 (dd, *J* = 7.8, 7.4 Hz, 1H), 7.11 (s, 1H), 6.88 (d, *J* = 5.7

Hz, 2H), 5.65 – 5.59 (m, 1H), 4.01 (d, J = 4.5 Hz, 2H), 3.80 – 3.75 (m, 2H), 3.56 – 3.51 (m, 2H), 3.27 (m, 1H), 3.10 (s, 4H), 1.44 (s, 9H), 1.29 (d, J = 6.8 Hz, 6H); ^{13}C NMR (126 MHz, $CDCl_3$) δ 166.9, 162.6, 156.5, 155.9, 151.7, 151.6, 147.2, 139.3, 137.7, 134.6, 132.8, 131.3, 123.2, 122.8, 122.4, 122.3, 117.7, 79.8, 56.1, 50.1, 49.8, 44.3, 42.2, 41.9, 28.4, 15.4.

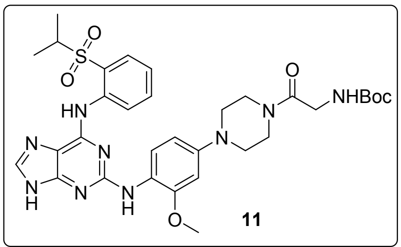

11

***tert*-butyl (2-(4-(4-((6-((2-(isopropylsulfonyl)phenyl)amino)-9*H*-purin-2-yl)amino)-3-methoxyphenyl) piperazin-1-yl)-2-oxoethyl)carbamate, 11:** *tert*-butyl 4-(4-((6-((2-(isopropylsulfonyl)phenyl)amino)-9-(tetrahydro-2H-pyran-2-yl)-9*H*-purin-2-yl)amino)-3-methoxyphenyl) piperazine-1-carboxylate (40 mg, 0.056 mmol) was dissolved in a mixture of dichloromethane/TFA 3/1 (0.85 mL) and the solution was stirred for 1h at ambient temperature. Afterwards the solvents were removed *in vacuo* to afford the desired amine salt, which was used in the next step without further purification. In a dried flask, Boc-Gly-OH (9 mg, 0.051 mmol) was dissolved in dry DMF (0.94 mL) 4-(4-((6-((2-(isopropylsulfonyl)phenyl)amino)-9*H*-purin-2-yl)amino)-3-methoxyphenyl)piperazin-1-ium 2,2,2-trifluoroacetate (36 mg, 0.056 mmol), HOBt (14 mg, 0.103 mmol), EDCI (20 mg, 0.103 mmol) and triethylamine (16 mg, 0.154 mmol) were added under Ar atmosphere. The reaction mixture was stirred for 18 h at ambient temperature and upon completion of the reaction the solvent was removed under reduced pressure. The crude mixture was purified by flash chromatography (eluent; 3% MeOH in DCM) to afford **11** as a yellow oil (32 mg, yield 84%). **11:** ^{1}H NMR (500 MHz, $CDCl_3$) δ 11.92 (br, 1H), 10.04 (s, 1H), 8.89 (d, J = 8.4 Hz, 1H), 8.09 (d, J = 8.6 Hz, 1H), 7.88 (d, J = 7.9 Hz, 1H), 7.64 (dd, J = 7.9, 7.8 Hz, 1H), 7.41 (s, 1H), 7.20 (dd, J = 8.4, 7.8 Hz, 1H), 7.16 (s, 1H), 6.56 (s, 1H), 6.48 (d, J = 8.6 Hz, 1H), 5.62 (s, 1H), 4.03 (d, J = 3.8 Hz, 2H), 3.89 (s, 3H), 3.80 (br, 2H), 3.56 (br, 2H), 3.34 – 3.24 (m, 1H), 3.12 (s, 4H), 1.45 (s, 9H), 1.31 (d, J = 6.8 Hz, 6H); ^{13}C NMR (126 MHz, $CDCl_3$) δ 167.1, 156.4, 156.0, 152.0, 151.6, 150.2, 147.2, 139.5, 137.7, 134.8, 131.4, 123.4, 123.1, 122.6, 122.4, 121.4, 116.1, 108.9, 101.5, 79.9, 56.1, 55.8, 50.7, 50.4, 44.5, 42.4, 42.1, 28.5, 15.6.

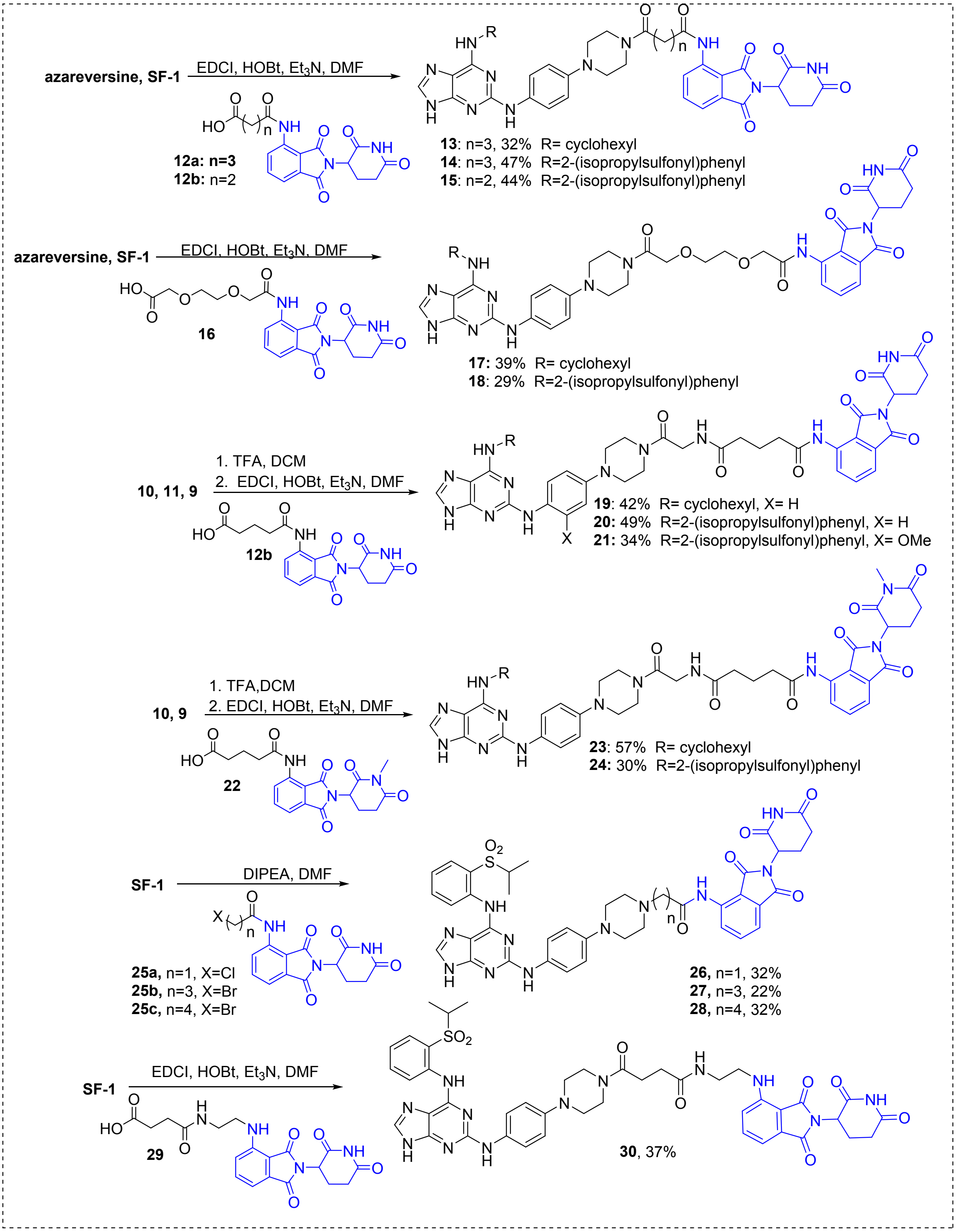

Scheme S2. Synthesis of pomalidomide-bearing chimeras

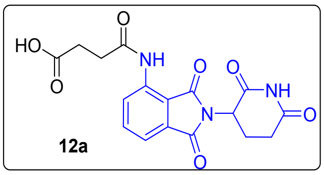

12a

4-((2-(2,6-dioxopiperidin-3-yl)-1,3-dioxoisoindolin-4-yl)amino)-4-oxobutanoic acid, **12a**: In a flask containing 4-amino-2-(2,6-dioxopiperidin-3-yl)isoindoline-1,3-dione (200 mg, 0.732 mmol) as a suspension in acetic acid (6 mL), potassium acetate (288 mg, 2.928 mmol) was added followed by the addition of succinic anhydride (293 mg, 2.928 mmol) and the reaction mixture stirred under reflux for 3h. After cooling at room temperature, acetic acid was removed under reduced pressure and the residue was extracted with ethyl acetate/H_2O. The organic phases were collected, dried with Na_2SO_4, filtered and solvents were removed under reduced pressure. The crude product was purified by silica gel column chromatography (eluent; 2.5-10% MeOH in ethyl acetate) to give the desired product as an off-white solid (87 mg, 32% yield).[3]

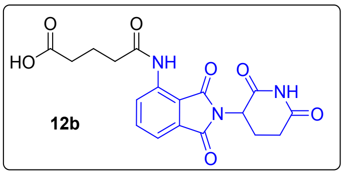

12b

5-((2-(2,6-dioxopiperidin-3-yl)-1,3-dioxoisoindolin-4-yl)amino)-5-oxopentanoic acid, 12b: In a flask containing 4-amino-2-(2,6-dioxopiperidin-3-yl)isoindoline-1,3-dione (200 mg, 0.732 mmol) as a suspension in acetic acid (6 mL), potassium acetate (288 mg, 2.928 mmol) was added followed by the addition of succinic anhydride (334 mg, 2.928 mmol) and the reaction mixture stirred under reflux for 3h. After cooling at room temperature, acetic acid was removed under reduced pressure and the residue was extracted with ethyl acetate/H_2O. The organic phases were collected, dried with Na_2SO_4, filtered and solvents were removed under reduced pressure. The crude product was purified by silica gel column chromatography (eluent; 2.5-10% MeOH in ethyl acetate) to give the desired product as an off-white solid (113 mg, 40%).[4] **12b**: ^{1}H NMR (500 MHz, DMSO-*d*6) δ 12.09 (s, 1H), 11.14 (s, 1H), 9.72 (s, 1H), 8.44 (d, *J* = 8.4 Hz, 1H), 7.99 – 7.75 (m, 1H), 7.62 (d, *J* = 7.2 Hz, 1H), 5.14 (dd, *J* = 12.8, 5.4 Hz, 1H), 2.89 (m, 1H), 2.65 – 2.52 (m, 4H), 2.31 (t, *J* = 7.3 Hz, 2H), 2.10 – 2.02 (m, 1H), 1.89 – 1.80 (m, 2H).

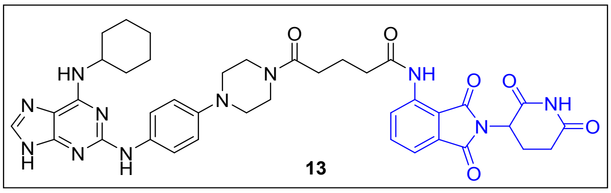

13

5-(4-(4-((6-(cyclohexylamino)-9*H*-purin-2-yl)amino)phenyl)piperazin-1-yl)-*N*-(2-(2,6-dioxopiperidin-3-yl)-1,3-dioxoisoindolin-4-yl)-5-oxopentanamide, 13: In a dried flask containing **12b** (16 mg, 0.041 mmol) dry DMF (0.9 mL), *N*6-cyclohexyl-*N*2-(4-(piperazin-1-yl)phenyl)-9*H*-purine-2,6-diamine (18 mg, 0.045 mmol), HATU (18 mg, 0.48 mmol) and DIPEA (28 mg, 0.218 mmol) were added under Ar atmosphere. The reaction mixture was stirred overnight at room temperature. After the reaction's completion the solvent was evaporated and the crude mixture was purified via preparative HPLC to afford the desired product as a white solid (10 mg, yield 32%). **13:** ^{1}H NMR (500 MHz, DMSO-*d*6) δ 11.15 (s, 1H), 9.73 (s, 1H), 8.53 (br, 1H), 8.45 (d, *J* = 8.4 Hz, 1H), 8.21 (s, 1H), 7.83 (t, *J* = 7.9 Hz, 1H), 7.75 (s, 1H), 7.67 (d, *J* = 8.7 Hz, 2H), 7.62 (d, *J* = 7.3 Hz, 1H), 7.05 (br, 1H), 6.85 (d, *J* = 8.9 Hz, 2H), 5.14 (dd, *J* = 12.8, 5.4 Hz, 1H), 4.05 (br, 1H), 3.60 (br, 4H), 3.00 (m, 2H), 2.96 (m, 2H) 2.92 – 2.82 (m, 1H), 2.64 – 2.57 (m, 1H), 2.55 – 2.51 (m, 2H), 2.46 (t, *J* = 7.3 Hz, 2H), 2.09 – 2.01 (m, 1H), 1.93 – 1.83

(m, 2H), 1.76 (m, 2H), 1.64 (d, *J* = 12.2 Hz, 1H), 1.43 – 1.29 (m, 5H), 1.20 – 1.11 (m, 1H); ^{13}C NMR (126 MHz, DMSO-*d6*) δ 172.7, 171.8, 170.1, 169.8, 167.6, 166.7, 163.8, 156.5, 144.9, 136.5, 136.1, 135.1, 131.5, 126.5, 119.2, 118.4, 117.2, 116.7, 50.1, 49.7, 48.9, 44.9, 41.0, 35.8, 32.6, 31.4, 30.9, 25.3, 25.2, 21.9, 20.5; ESI-HRMS m/z for $C_{39}H_{44}N_{11}O_6$ $[M/2+H]^+$ calcd 381.6766, found 381.6766.

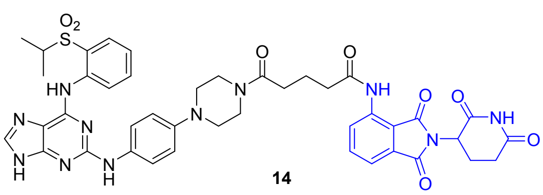

14

***N*-(2-(2,6-dioxopiperidin-3-yl)-1,3-dioxoisoindolin-4-yl)-5-(4-(4-((6-((2-(isopropylsulfonyl)phenyl)amino)-9*H*-purin-2-yl)amino)phenyl)piperazin-1-yl)-5-oxopentanamide, 14:** In a dried flask containing 5-((2-(2,6-dioxopiperidin-3-yl)-1,3-dioxoisoindolin-4-yl)amino)-5-oxopentanoic acid (14 mg, 0.037 mmol) dry DMF (0.9 mL), *N*6-(2-(isopropylsulfonyl)phenyl)-*N*2-(4-(piperazin-1-yl)phenyl)-9H-purine-2,6-diamine (22 mg, 0.044 mmol), HOBt (8 mg, 0.056 mmol), EDCI (11 mg, 0.056 mmol) and triethylamine (15 mg, 0.15 mmol) were added under Ar atmosphere. The reaction mixture was stirred overnight at room temperature. After the reaction's completion the solvent was evaporated and the crude mixture was purified by flash chromatography (eluent: 2% MeOH in dichloromethane) to afford the desired product as an orange solid (15 mg, yield 47%). **14:** ^{1}H NMR (500 MHz, DMSO-*d6*) δ 12.74 (br, 1H), 11.16 (br, 1H), 9.85 (s, 1H), 9.74 (s, 1H), 9.06 (d, *J* = 8.0 Hz, 1H), 9.03 (s, 1H), 8.45 (d, *J* = 8.4 Hz, 1H), 7.97 (s, 1H), 7.85 – 7.81 (m, 2H), 7.75 –7.72 (m, 1H), 7.65 – 7.54 (m, 3H), 7.30 – 7.27 (m, 1H), 6.92 (d, *J* = 8.6 Hz, 2H), 5.14 (dd, *J* = 12.7, 5.3 Hz, 1H), 3.61 (br, 4H), 3.43 (m, 1H), 3.06 (m, 2H), 3.03 (m, 2H), 2.97 – 2.85 (m, 1H), 2.60 – 2.44 (m, 6H), 2.06 (d, *J* = 10.1 Hz, 1H), 1.92 – 1.81 (m, 2H), 1.18 (d, *J* = 6.6 Hz, 6H); ^{13}C NMR (126 MHz, DMSO-*d6*) δ 172.8, 171.8, 170.1, 169.8, 167.6, 166.7, 156.1, 152.3, 150.5, 145.7, 139.3, 138.4, 136.5, 136.1, 135.1, 133.8, 131.5, 130.9, 126.5, 122.3, 122.1, 122.1, 120.5, 118.4, 117.2, 116.6, 109.5, 55.2, 49.8, 49.4, 48.9, 44.8, 41.0, 35.8, 31.4, 30.9, 22.0, 20.5, 15.0; ESI-HRMS m/z for $C_{42}H_{44}N_{11}O_8S$ $[M+H]^+$ calcd 862.3090, found 862.3113.

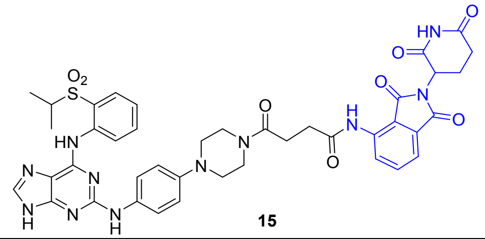

15

***N*-(2-(2,6-dioxopiperidin-3-yl)-1,3-dioxoisoindolin-4-yl)-4-(4-(4-((6-((2-(isopropylsulfonyl)phenyl)amino)-9*H*-purin-2-yl)amino)phenyl)piperazin-1-yl)-4-oxobutanamide, 15:** In a dried flask containing 4-((2-(2,6-dioxopiperidin-3-yl)-1,3-dioxoisoindolin-4-yl)amino)-4-oxobutanoic acid (21 mg, 0.056 mmol) dry DMF (1.3 mL), *N*6-(2-(isopropylsulfonyl)phenyl)-*N*2-(4-(piperazin-1-yl)phenyl)-9*H*-purine-2,6-diamine (33 mg, 0.067 mmol), HOBt (11 mg, 0.084 mmol), EDCI (16 mg, 0.084 mmol) and triethylamine (33 mg, 0.17 mmol) were added under Ar atmosphere. The reaction mixture was stirred overnight at room temperature. After the reaction's completion the solvent was evaporated, and the crude mixture was purified by flash chromatography (eluent: 2% MeOH in dichloromethane) to afford the desired product as an orange solid (21 mg, yield 44%). **15:** ^{1}H NMR (500 MHz, DMSO-*d6*) δ 12.75 (br, 1H), 11.16 (br, 1H), 9.85 (s, 1H), 9.76 (s, 1H), 9.06 (d, *J* = 8.1 Hz, 1H), 9.03 (s, 1H), 8.50 (d, *J* = 8.4 Hz, 1H), 7.97 (s, 1H), 7.82 (m, 2H), 7.75 – 7.72 (m, 1H), 7.60 (d, *J* = 7.6 Hz, 2H), 7.30 – 7.27 (m, 1H), 6.93 (d, *J* = 8.9 Hz, 2H), 5.15 (dd, *J* = 12.8, 5.4 Hz, 1H), 3.63 (m, 4H), 3.43 (m, 1H), 3.11 (br, 2H), 3.02 (br, 2H), 2.90 (m, 1H), 2.77 – 2.69 (m, 4H), 2.64 – 2.57 (m, 2H), 2.56 – 2.52 (m, 1H), 2.10 – 2.02 (m, 1H), 1.18 (d, *J* = 6.8 Hz, 6H); ^{13}C NMR (126 MHz, DMSO-*d6*) δ 173.2, 172.1, 170.2, 169.9, 168.2,

167.1, 156.6, 152.7, 150.9, 146.2, 139.7, 138.8, 137.1, 136.6, 135.6, 134.3, 131.9, 131.4, 126.3, 122.7, 122.5, 120.9, 118.6, 117.0, 116.9, 115.4, 109.9, 55.6, 50.2, 49.8, 49.4, 45.1, 41.6, 32.3, 31.4, 27.9, 22.4, 15.4; ESI-HRMS m/z for $C_{41}H_{42}N_{11}O_8S$ [M+H]+ calcd 848.2939, found 848.2936.

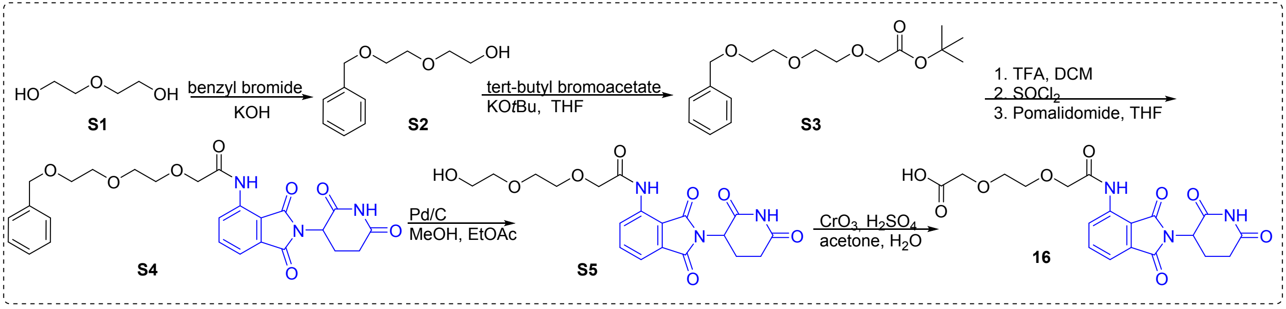

Scheme S3. Synthesis of pomalidomide intermediates

2-(2-(benzyloxy)ethoxy)ethan-1-ol, **S2**: To a dried self-sealing tube containing diethylene glycol (5.7 g, 53.9 mmol), KOH (992 mg, 17.7 mmol) was added. The mixture was heated to 90 °C until KOH was dissolved. Afterwards benzyl bromide (3.03 g, 17.7mmol) was added dropwise and the reaction was left stirring at 110 °C overnight. Upon completion of the reaction, the mixture was extracted with ethyl acetate and water. The organic phases were dried over Na_2SO_4, filtered and concentrated under reduced pressure. The crude was purified by silica gel chromatography (eluent; petroleum ether/ethyl acetate=2/1) to afford **S2** in 70% yield.[5]

***tert*-butyl 2-(2-(2-(benzyloxy)ethoxy)ethoxy)acetate**, **S3**: In a dried flask containing potassium tert-butoxide (1.17 g, 10.4 mmol) dry THF (52 mL) was added and the suspension was cooled to 0 °C. Then **S2** (1.97 g, 10 mmol) was added, and the mixture was heated at 40 °C for 30 minutes. Afterwards it was cooled to 0 °C and *tert*-butyl bromoacetate (1.95 g, 10 mmol) was added dropwise. The reaction was left stirring at ambient temperature overnight. Upon completion of the reaction, the mixture was extracted with ethyl acetate and water. The organic phases were dried over Na_2SO_4, filtered and concentrated under reduced pressure. The crude was purified by silica gel chromatography (eluent; petroleum ether/ethyl acetate=3/1) to afford CSI52 in 77% yield.[6] **S3:** ^{1}H NMR (500 MHz, $CDCl_3$) δ 7.40 –7.21 (m, 5H), 4.56 (d, *J*= 1.2 Hz, 2H), 4.02 (d, *J*= 1.3 Hz, 2H), 3.76 –3.58 (m, 9H), 1.47 (d, *J*= 1.3 Hz, 9H).

***N*-(2-(2,6-dioxopiperidin-3-yl)-1,3-dioxoisoindolin-4-yl)-2-(2-(2-hydroxyethoxy)ethoxy)acetamide, S5:** 750 mg (2.42 mmol) of **S3** were dissolved in a mixture of TFA (0.3 mL) and DCM (1 mL). The resulting solution was stirred at room temperature for 2 h. The solvents were evaporated, and the residue dissolved in $SOCl_2$ (1.5 mL) and heated at 60 °C for 1h. The solvent was evaporated, and the crude product was dissolved in dry THF (3 mL). To this solution was added pomalidomide (100 mg, 0.37 mmol). The resulting mixture was refluxed for 16 h. After cooling to room temperature, it was filtered through celite to give 95 mg of **S4** which was carried to the next step without further purification.

To a self-sealing tube **S4** (95 mg, 0.186 mmol) was diluted in a mixture of MeOH/ethyl acetate 1/1 and catalytic amount of Pd/C was added. Afterwards a hydrogen filled balloon was adjusted, the system was degassed, and the mixture was stirred at 110 °C overnight. Then the solvents were evaporated, and the crude mixture was purified by silica gel chromatography (eluent; dichloromethane/acetone=8/1), to afford **S5** as a yellow solid in 36% yield (28 mg).[6] **S5:** ^{1}H NMR (500 MHz, DMSO-*d*6) δ 11.12 (s, 1H), 10.34 (s, 1H), 8.70 (d, *J*= 8.5 Hz, 1H), 7.85 (t, *J*= 7.9 Hz, 1H), 7.61 (d, *J*= 7.3 Hz, 1H), 5.14 (dd, *J*= 12.8, 5.4 Hz, 1H), 4.51 (t, *J*= 5.3 Hz, 1H), 4.19 (s, 2H), 3.74 (dd, *J*= 5.8, 3.6 Hz, 2H), 3.64 (dd, *J*= 9.7, 5.5 Hz, 2H), 3.44 (m, 4H), 2.87 (m, 1H), 2.63 –2.52 (m, 2H), 2.09 –2.01 (m, 1H).

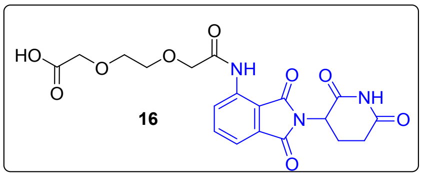

2-(2-(2-((2-(2,6-dioxopiperidin-3-yl)-1,3-dioxoisoindolin-4-yl)amino)-2-oxoethoxy)ethoxy)acetic acid, 16 : To a solution of **S5** (27 mg, 0.064 mmol) in acetone (0.7 mL) was added Jone`s reagent mixture of H_2SO_4 (16 µL) and CrO_3 (16 mg, 0.16 mmol) in water (0.05 mL) at 0°C. The mixture was stirred for 16 h. After the reaction was completed, the mixture was quenched with isopropanol (to reduce the toxic Chromium (VI)). The resulting solution was extracted with dichloromethane (x3) and the combined organic layers were dried over $MgSO_4$ and evaporated under vacuo. The crude product was used to the next step without further purification.[6]

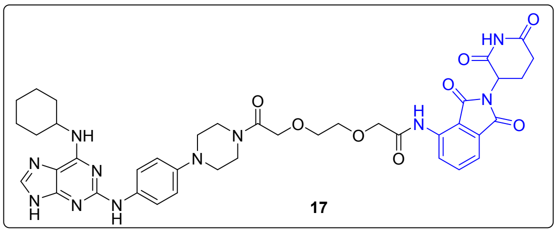

2-(2-(2-(4-(4-((6-(cyclohexylamino)-9*H*-purin-2-yl)amino)phenyl)piperazin-1-yl)-2-oxoethoxy)ethoxy)-N-(2-(2,6-dioxopiperidin-3-yl)-1,3-dioxoisoindolin-4-yl)acetamide, 17: In a dried flask containing 2-(2-(2-((2-(2,6-dioxopiperidin-3-yl)-1,3-dioxoisoindolin-4-yl)amino)-2-oxoethoxy)ethoxy)acetic acid **16** (11 mg, 0.025 mmol) dry DMF (0.6 mL), *N*6-cyclohexyl-*N*2-(4-(piperazin-1-yl)phenyl)-9*H*-purine-2,6-diamine (11 mg, 0.028 mmol), HATU (12 mg, 0.03 mmol) and DIPEA (20 mg, 0.15 mmol) were added under Ar atmosphere. The reaction mixture was stirred overnight at room temperature. After the reaction's completion the solvent was evaporated, and the crude mixture was purified via preparative HPLC to afford the desired product as a white solid (8 mg, yield 39%). **17:** ^1H NMR (500 MHz, DMSO-*d*6) δ 11.16 (s, 1H), 10.37 (s, 1H), 8.72 (d, *J* = 8.3 Hz, 1H), 8.51 (s, 1H), 7.89 – 7.82 (m, 1H), 7.75 (s, 1H), 7.70 – 7.56 (m, 3H), 7.07 (br, 1H), 6.79 (d, *J* = 9.0 Hz, 2H), 5.16 (dd, *J* = 13.0, 5.3 Hz, 1H), 4.23 (br, 4H), 3.83 – 3.78 (m, 2H), 3.74 – 3.70 (m, 2H), 3.57 – 3.48 (m, 4H), 2.98 – 2.93 (m, 4H), 2.91 – 2.85 (m, 1H), 2.64 – 2.58 (m, 1H), 2.58 – 2.51 (m, 1H), 2.13 – 2.04 (m, 1H), 1.91 (m, 2H), 1.76 (m, 2H), 1.64 (d, *J* = 12.3 Hz, 1H), 1.42 – 1.29 (m, 5H), 1.16 (m, 1H); ^{13}C NMR (126 MHz, DMSO-*d*6) δ 173.2, 170.2, 169.7, 168.7, 167.6, 167.1, 156.9, 152.3, 145.2, 136.9, 136.4, 135.6, 135.6, 131.7, 124.9, 119.6, 118.8, 117.1, 116.6, 71.0, 70.5, 70.2, 69.9, 50.4, 50.1, 49.4, 44.7, 41.5, 33.1, 31.4, 25.8, 25.6, 22.4; ESI-HRMS m/z for $C_{40}H_{46}N_{11}O_8$ $[M+H]^+$ calcd 808.3525, found 808.3544.

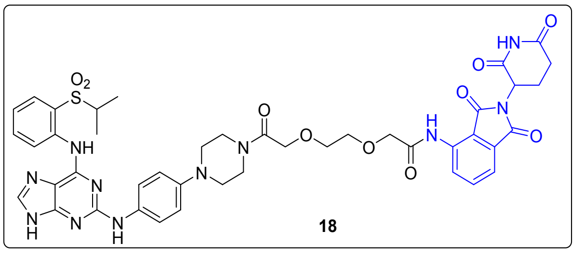

***N*-(2-(2,6-dioxopiperidin-3-yl)-1,3-dioxoisoindolin-4-yl)-2-(2-(2-(4-(4-((6-((2-(isopropylsulfonyl) phenyl)amino)-9*H*-purin-2-yl)amino)phenyl)piperazin-1-yl)-2-oxoethoxy)ethoxy)acetamide 18:** In a dried flask containing 2-(2-(2-((2-(2,6-dioxopiperidin-3-yl)-1,3-dioxoisoindolin-4-yl)amino)-2-oxoethoxy) ethoxy)acetic acid (12 mg, 0.028 mmol) **16** dry DMF (0.6 mL), *N*6-(2-(isopropylsulfonyl)phenyl)-*N*2-(4-(piperazin-1-yl)phenyl)-9*H*-purine-2,6-diamine (17 mg, 0.034 mmol), HOBt (4 mg, 0.042 mmol), EDCI (8 mg, 0.042 mmol) and triethylamine (11 mg, 0.11 mmol) were added under Ar atmosphere. The reaction mixture

was stirred overnight at room temperature. After the reaction's completion the solvent was evaporated and the crude mixture was purified by flash chromatography (eluent: 4% MeOH in dichloromethane) to afford the desired product as a yellow solid (8 mg, yield 29%). **18:** ^{1}H NMR (500 MHz, DMSO-*d6*) δ 12.73 (s, 1H), 11.16 (s, 1H), 10.37 (s, 1H), 9.85 (s, 1H), 9.05 (d, *J* = 8.5 Hz, 1H), 9.00 (s, 1H), 8.71 (d, *J* = 8.3 Hz, 1H), 7.96 (s, 1H), 7.88 – 7.79 (m, 2H), 7.75 – 7.70 (m, 1H), 7.61 (d, *J* = 7.3 Hz, 1H), 7.56 (d, *J* = 8.8 Hz, 2H), 7.30 – 7.27 (m, 1H), 6.85 (d, *J* = 9.0 Hz, 2H), 5.16 (dd, *J* = 12.8, 5.5 Hz, 1H), 4.24 (d, *J* = 4.6 Hz, 4H), 3.83 – 3.79 (m, 2H), 3.74 – 3.71 (m, 2H), 3.59 – 3.50 (m, 4H), 3.43 (m, 2H), 2.96 (br, 4H), 2.95 – 2.85 (m, 1H), 2.64 – 2.53 (m, 3H), 2.11 – 2.03 (m, 1H), 1.19 (d, *J* = 6.8 Hz, 6H); ^{13}C NMR (126 MHz, DMSO-*d6*) δ 172.7, 169.8, 169.3, 168.2, 167.2, 166.7, 156.1, 152.2, 150.5, 145.6, 139.3, 138.3, 136.5, 135.9, 135.1, 133.8, 131.3, 130.9, 124.4, 122.3, 122.1, 122.1, 120.4, 118.4, 116.5, 116.1, 114.9, 109.5, 70.6, 70.1, 69.8, 69.5, 55.2, 49.7, 49.3, 48.9, 44.2, 41.1, 30.9, 22.0, 14.9; ESI-HRMS m/z for $C_{43}H_{46}N_{11}O_{10}S$ [M+H]$^{+}$ calcd 908.3144, found 908.3168.

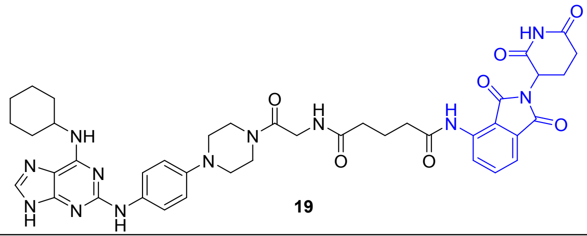

19

***N*1-(2-(4-(4-((6-(cyclohexylamino)-9*H*-purin-2-yl)amino)phenyl)piperazin-1-yl)-2-oxoethyl)-N5-(2-(2,6-dioxopiperidin-3-yl)-1,3-dioxoisoindolin-4-yl)glutaramide, 19:** *Tert*-butyl (2-(4-(4-((6-(cyclohexylamino)-9*H*-purin-2-yl)amino)phenyl)piperazin-1-yl)-2-oxoethyl)carbamate (25 mg, 0.047 mmol) **9** was dissolved in a mixture of dichloromethane/TFA 2/1 (0.4 mL) and the solution was stirred for 1h at ambient temperature. Afterwards the solvents were removed *in vacuo* to afford the desired salt, which was used in the next step without further purification. In a dried flask containing 5-((2-(2,6-dioxopiperidin-3-yl)-1,3-dioxoisoindolin-4-yl)amino)-5-oxopentanoic acid (17 mg, 0.044 mmol) dry DMF (1 mL), 2-(4-(4-((6-(cyclohexylamino)-9H-purin-2-yl)amino)phenyl)piperazin-1-yl)-2-oxoethan-1-aminium 2,2,2-trifluoroacetate (26 mg, 0.048 mmol), HATU (33 mg, 0.087 mmol) and DIPEA (34 mg, 0.26 mmol) were added under Ar atmosphere. The reaction mixture was stirred overnight at room temperature. After the reaction's completion the solvent was evaporated and the crude mixture was purified by flash chromatography (eluent: 5% MeOH in dichloromethane) to afford the desired product as a beige solid (15 mg, yield 42%). **19:** ^{1}H NMR (500 MHz, DMSO-*d6*) δ 11.16 (s, 1H), 9.72 (s, 1H), 8.85 (br, 1H), 8.47 (d, *J* = 8.4 Hz, 1H), 8.01 (t, *J* = 5.4 Hz, 1H), 7.93 (s, 1H), 7.85 – 7.82 (m, 1H), 7.62 (m, 2H), 6.89 (d, *J* = 8.5 Hz, 2H), 5.14 (dd, *J* = 12.8, 5.4 Hz, 1H), 3.99 (d, *J* = 5.5 Hz, 2H), 3.63 – 3.55 (m, 4H), 3.17 – 3.10 (m, 1H), 3.03 (m, 2H), 2.98 (m, 2H), 2.94 – 2.84 (m, 1H), 2.60 (m, 2H), 2.55 – 2.51 (m, 2H), 2.25 (t, *J* = 7.3 Hz, 2H), 2.06 (dd, *J* = 12.0, 6.3 Hz, 1H), 1.94 (d, *J* = 7.5 Hz, 2H), 1.89 – 1.82 (m, 2H), 1.77 (d, *J* = 5.5 Hz, 2H), 1.64 (d, *J* = 12.5 Hz, 1H), 1.42 – 1.29 (m, 5H); ^{13}C NMR (126 MHz, DMSO-*d6*) δ 172.8, 171.9, 171.8, 169.8, 167.6, 167.1, 166.7, 163.1, 158.0, 157.8, 157.5, 136.5, 136.1, 131.5, 126.5, 124.9, 118.5, 118.4, 117.2, 116.7, 116.1, 109.6, 53.6, 49.8, 49.5, 48.9, 44.1, 41.4, 40.4, 35.8, 34.3, 31.0, 29.1, 25.3, 25.1, 22.0, 21.1, 18.1, 16.8; ESI-HRMS m/z for $C_{41}H_{47}N_{12}O_{7}$ [M+H]$^{+}$ calcd 819.3685, found 819.3707.

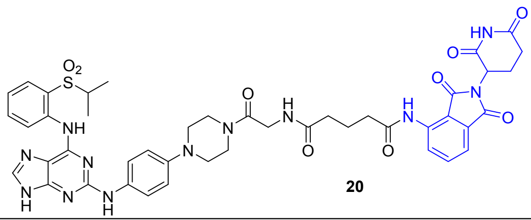

20

***N*1-(2-(2,6-dioxopiperidin-3-yl)-1,3-dioxoisoindolin-4-yl)-*N*5-(2-(4-(4-((6-((2-(isopropylsulfonyl)phenyl)amino)-9*H*-purin-2-yl)amino)phenyl)piperazin-1-yl)-2-oxoethyl)glutaramide, 20:** *Tert*-butyl (2-(4-(4-((6-((2-

(isopropylsulfonyl)phenyl)amino)-9*H*-purin-2-yl)amino)phenyl) piperazin-1-yl)-2-oxoethyl)carbamate (22 mg, 0.034 mmol) **10** was dissolved in a mixture of dichloromethane/TFA 2/1 (0.35 mL) and the solution was stirred for 1h at ambient temperature. Afterwards the solvents were removed *in vacuo* to afford the desired salt which was used in the next step without further purification. In a dried flask containing 5-((2-(2,6-dioxopiperidin-3-yl)-1,3-dioxoisoindolin-4-yl)amino)-5-oxopentanoic acid (12 mg, 0.031 mmol) dry DMF (0.7 mL), 2-(4-(4-((6-((2-(isopropylsulfonyl)phenyl)amino)-9H-purin-2-yl)amino)phenyl)piperazin-1-yl)-2-oxoethan-1-aminium 2,2,2-trifluoroacetate (23 mg, 0.034 mmol), HATU (14 mg, 0.037 mmol), and DIPEA (24 mg, 0.18 mmol) were added under Ar atmosphere. The reaction mixture was stirred overnight at room temperature. After the reaction's completion the solvent was evaporated and the crude mixture was purified by flash chromatography (eluent: 3% MeOH in dichloromethane) to afford the desired product as an orange solid (14 mg, yield 49%). **20**: ^{1}H NMR (500 MHz, DMSO-*d6*)) δ 12.79 (br, 1H), 11.17 (s, 1H), 9.85 (s, 1H), 9.73 (s, 1H), 9.06 (d, *J* = 10.9 Hz, 2H), 8.47 (d, *J* = 8.4 Hz, 1H), 8.03 (t, *J* = 5.2 Hz, 1H), 7.97 (s, 1H), 7.86 – 7.77 (m, 2H), 7.74 (dd, *J* = 8.1 Hz, 7.7 Hz, 1H), 7.66 – 7.54 (m, 3H), 7.30 – 7.27 (m, 1H), 6.93 (d, *J* = 8.8 Hz, 2H), 5.15 (dd, *J* = 12.8, 5.4 Hz, 1H), 4.00 (d, *J* = 5.4 Hz, 2H), 3.60 (d, *J* = 10.3 Hz, 4H), 3.43 (m, 1H), 3.07 (m, 2H), 3.02 (m, 2H) 2.94 – 2.83 (m, 1H), 2.60 – 2.51 (m, 4H), 2.26 (t, *J* = 7.2 Hz, 2H), 2.07 (m, 1H), 1.93 – 1.82 (m, 2H), 1.18 (d, J = 6.7 Hz, 6H); ^{13}C NMR (126 MHz, DMSO-*d6*) δ 173.2, 172.3, 172.2, 170.2, 168.0, 167.5, 167.1, 156.6, 152.7, 150.9, 146.1, 139.7, 138.8, 136.9, 136.5, 135.3, 134.3, 131.9, 131.4, 126.9, 122.7, 122.8, 122.5, 120.9, 118.8, 117.6, 117.1, 115.4, 55.6, 50.2, 49.8, 49.3, 44.5, 41.5, 40.8, 36.2, 34.7, 31.4, 22.4, 21.5, 15.4; ESI-HRMS m/z for $C_{44}H_{47}N_{12}O_9S$ $[M+H]^+$ calcd 919.3304, found 919.3338.

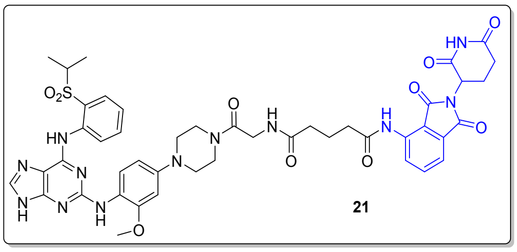

***N*^{1}-(2-(2,6-dioxopiperidin-3-yl)-1,3-dioxoisoindolin-4-yl)-*N*^{5}-(2-(4-(4-((6-((2-(isopropylsulfonyl) phenyl)amino)-9*H*-purin-2-yl)amino)-3-methoxyphenyl)piperazin-1-yl)-2-oxoethyl)glutaramide, 21:** *Tert*-butyl (2-(4-(4-((6-((2-(isopropylsulfonyl)phenyl)amino)-9*H*-purin-2-yl)amino)-3-methoxyphenyl)piperazin-1-yl)-2-oxoethyl)carbamate (32 mg, 0.047 mmol) **11** was dissolved in a mixture of dichloromethane/TFA 2/1 (0.7 mL) and the solution was stirred for 1h at ambient temperature. Afterwards the solvents were removed *in vacuo* to afford the desired salt which was used in the next step without further purification. In a dried flask containing 5-((2-(2,6-dioxopiperidin-3-yl)-1,3-dioxoisoindolin-4-yl)amino)-5-oxopentanoic acid (16 mg, 0.043 mmol) dry DMF (0.8 mL), 2-(4-(4-((6-((2-(isopropylsulfonyl)phenyl)amino)-9*H*-purin-2-yl)amino)-3-methoxyphenyl)piperazin-1-yl)-2-oxoethan-1-aminium 2,2,2-trifluoroacetate (33 mg, 0.047 mmol), EDCI (16 mg, 0.085 mmol), HOBt (11 mg, 0.085 mmol) and triethylamine (13 mg, 0.128 mmol) were added under Ar atmosphere. The reaction mixture was stirred overnight at room temperature. After the reaction's completion the solvent was evaporated and the crude mixture was purified by flash chromatography (eluent: 4% MeOH in dichloromethane) to afford the desired product as an orange solid (14 mg, yield 34%). **21**: ^{1}H NMR (500 MHz, DMSO-*d6*) δ 12.71 (s, 1H), 11.16 (s, 1H), 9.84 (s, 1H), 9.72 (s, 1H), 8.94 (d, *J* = 8.4 Hz, 1H), 8.47 (d, *J* = 8.4 Hz, 1H), 8.02 (t, *J* = 5.6 Hz, 1H), 7.95 (s, 1H), 7.86 – 7.77 (m, 3H), 7.73 (d, *J* = 8.5 Hz, 1H), 7.68 – 7.65 (m, 1H), 7.61 (d, *J* = 7.3 Hz, 1H), 7.28 – 7.25 (m, 1H), 6.70 (d, *J* = 2.1 Hz, 1H), 6.53 (dd, *J* = 8.7, 2.1 Hz, 1H), 5.15 (dd, *J* = 12.8, 5.4 Hz, 1H), 4.01 (d, *J* = 5.4 Hz, 2H), 3.81 (s, 3H), 3.65 – 3.56 (m, 4H), 3.42 (dt, *J* = 13.6, 6.8 Hz, 2H), 3.14 (m, 2H), 3.08 (m, 2H), 2.94 – 2.84 (m, 1H), 2.60 (d, *J* = 18.2 Hz, 1H), 2.53 (m, 2H), 2.26 (t, *J* = 7.3 Hz, 2H), 2.11 – 2.02 (m, 1H), 1.92 – 1.83 (m, 2H), 1.18 (d, *J* = 6.8 Hz, 6H); ^{13}C NMR (126 MHz, DMSO-*d6*) δ 172.8, 171.9, 171.7, 169.8, 167.6, 167.1, 166.7, 156.8, 152.5, 151.5, 150.5, 147.9, 139.2, 138.3, 136.5, 136.1, 135.1, 131.5, 130.9, 126.4, 123.7, 122.2, 122.0, 121.8, 118.3, 117.1, 114.9, 109.6, 107.4, 100.9, 55.6, 55.2, 49.6, 49.2, 48.9, 44.1,

41.4, 40.4, 35.8, 34.3, 31.0, 22.0, 21.1, 14.9; ESI-HRMS m/z for $C_{45}H_{49}N_{12}O_{10}S$ $[M+H]^+$ calcd 949.3415, found 949.3403.

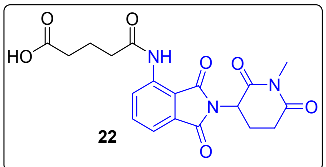

22

5-((2-(2,6-dioxopiperidin-3-yl)-1,3-dioxoisoindolin-4-yl)amino)-5-oxopentanoic acid, **22**: In a flask containing 4-amino-2-(1-methyl-2,6-dioxopiperidin-3-yl)isoindoline-1,3-dione (100 mg, 0.348 mmol) as a suspension in acetic acid (2.9 mL), potassium acetate (136 mg, 1.39 mmol) was added followed by the addition of glutaric anhydride (160 mg, 1.39 mmol) and the reaction mixture stirred under reflux for 3h. After cooling at room temperature, acetic acid was removed under reduced pressure and the residue was extracted with ethyl acetate/H_2O. The organic phases were collected, dried with $MgSO_4$, filtered and solvents were removed under reduced pressure. The crude product was purified by silica gel column chromatography (eluent; 2-10% MeOH in dichloromethane) to give the desire product as a white solid (44 mg, yield 32%). **22:** ^{1}H NMR (500 MHz, DMSO-*d6*) δ 9.73 (s, 1H), 8.44 (d, *J* = 8.4 Hz, 1H), 7.83 (dd, *J* = 8.4, 7.3 Hz, 1H), 7.62 (d, *J* = 7.3 Hz, 1H), 5.21 (dd, *J* = 13.1, 5.3 Hz, 1H), 3.02 (s, 3H), 3.00 – 2.90 (m, 2H), 2.78 (m, 2H), 2.60 – 2.52 (m, 1H), 2.31 (*t*, *J* = 7.2 Hz, 2H), 2.12 – 2.04 (m, 1H), 1.82 (m, 2H); ^{13}C NMR (126 MHz, DMSO-*d6*) δ 174.6, 172.2, 172.0, 169.9, 168.0, 167.1, 136.9, 136.5, 131.9, 127.0, 118.9, 117.7, 49.9, 35.9, 33.2, 31.5, 27.1, 21.6, 20.6; ESI-HRMS m/z for $C_{19}H_{20}N_3O_7$ $[M+H]^+$ calcd 402.1301, found 402.1295.

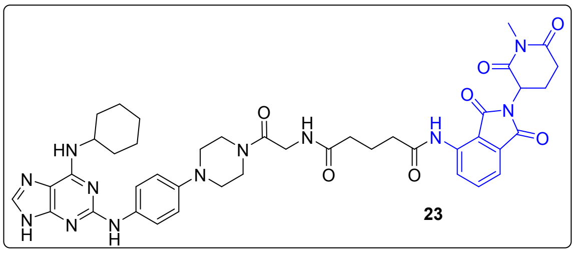

23

***N*1-(2-(4-(4-((6-(cyclohexylamino)-9*H*-purin-2-yl)amino)phenyl)piperazin-1-yl)-2-oxoethyl)-*N*5-(2-(1-methyl-2,6-dioxopiperidin-3-yl)-1,3-dioxoisoindolin-4-yl)glutaramide, 23:** *Tert*-butyl (2-(4-(4-((6-(cyclohexylamino)-9*H*-purin-2-yl)amino)phenyl)piperazin-1-yl)-2-oxoethyl)carbamate (33 mg, 0.06 mmol) **9** was dissolved in a mixture of dichloromethane/TFA 2/1 (0.55 mL) and the solution was stirred for 1h at ambient temperature. Afterwards the solvents were removed in rotary to afford the desired salt which was used in the next step without further purification. In a dried flask containing 5-((2-(1-methyl-2,6-dioxopiperidin-3-yl)-1,3-dioxoisoindolin-4-yl)amino)-5-oxopentanoic acid (20 mg, 0.049 mmol) dry DMF (1.2 mL), 2-(4-(4-((6-(cyclohexylamino)-9*H*-purin-2-yl)amino)phenyl)piperazin-1-yl)-2-oxoethan-1-aminium 2,2,2-trifluoroacetate (33 mg, 0.059 mmol), EDCI (19 mg, 0.099 mmol), HOBt (13 mg, 0.099 mmol) and triethylamine (15 mg, 0.149 mmol) were added under Ar atmosphere. The reaction mixture was stirred overnight at room temperature. After the reaction's completion the solvent was evaporated and the crude mixture was purified by flash chromatography (eluent: 4% MeOH in dichloromethane) to afford the desired product as an orange solid (24 mg, yield 57%). **23:** ^{1}H NMR (500 MHz, DMSO-d_6) δ 12.30 (s, 1H), 9.72 (s, 1H), 8.56 (br, 1H), 8.48 (d, *J* = 8.4 Hz, 1H), 8.00 (t, *J* = 5.4 Hz, 1H), 7.84 (t, *J* = 7.9 Hz, 1H), 7.74 (s, 1H), 7.67 (d, *J* = 8.7 Hz, 2H), 7.62 (d, *J* = 7.3 Hz, 1H), 7.09 (br, 1H), 6.86 (d, *J* = 8.9 Hz, 2H), 5.21 (dd, *J* = 13.0, 5.3 Hz, 1H), 3.99 (d, *J* = 5.3 Hz, 2H), 3.58 (m, 4H), 3.02 – 2.98 (m, 8H), 2.77 (d, *J* = 16.8 Hz, 1H), 2.60 – 2.52 (m, 2H), 2.25 (t, *J* = 7.2 Hz, 2H), 2.12 – 2.02 (m, 2H), 1.93 – 1.83 (m, 4H), 1.77 (d, *J* = 9.9 Hz, 2H), 1.64 (d, *J* = 12.2 Hz, 2H), 1.36 (m, 5H), 1.19 – 1.10 (m, 1H); ^{13}C NMR (126 MHz, DMSO-*d6*) δ 172.3, 172.2, 169.9, 168.1, 167.5, 167.1, 156.9, 154.1, 145.3, 137.0, 136.6, 136.3,

136.2, 135.6, 131.9, 126.9, 119.7, 118.8, 117.5, 117.2, 50.4, 50.1, 49.9, 44.6, 41.9, 40.8, 36.3, 34.7, 31.6, 27.2, 25.8, 25.7, 21.7, 21.5, one carbon missing due to overlapping; ESI-HRMS m/z for $C_{42}H_{49}N_{12}O_7$ [M+H]$^+$ calcd 833.3847, found 833.3831.

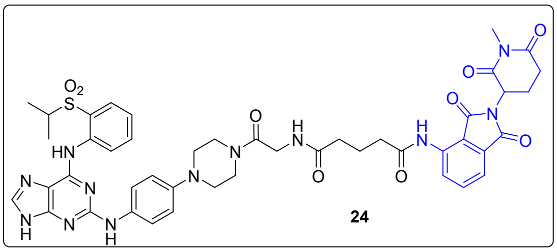

***N*1-(2-(4-(4-((6-((2-(isopropylsulfonyl)phenyl)amino)-9*H*-purin-2-yl)amino)phenyl)piperazin-1-yl)-2-oxoethyl)-*N*5-(2-(1-methyl-2,6-dioxopiperidin-3-yl)-1,3-dioxoisoindolin-4-yl)glutaramide, 24:** *Tert*-butyl (2-(4-(4-((6-((2-(isopropylsulfonyl)phenyl)amino)-9H-purin-2-yl)amino)phenyl)piperazin-1-yl)-2-oxoethyl)carbamate (40 mg, 0.062 mmol) **10** was dissolved in a mixture of dichloromethane/TFA 3/1 (0.5 mL) and the solution was stirred for 1h at ambient temperature. Afterwards the solvents were removed in rotary to afford the desired salt which was used in the next step without further purification. In a dried flask containing 5-((2-(1-methyl-2,6-dioxopiperidin-3-yl)-1,3-dioxoisoindolin-4-yl)amino)-5-oxopentanoic acid (23 mg, 0.057 mmol) dry DMF (1.4 mL), 2-(4-(4-((6-((2-(isopropylsulfonyl)phenyl)amino)-9H-purin-2-yl)amino)phenyl)piperazin-1-yl)-2-oxoethan-1-aminium 2,2,2-trifluoroacetate (41 mg, 0.063 mmol), HOBt (11 mg, 0.086 mmol), EDCI (16 mg, 0.086 mmol) and triethylamine (17 mg, 0.17 mmol) were added under Ar atmosphere. The reaction mixture was stirred overnight at room temperature. After the reaction's completion the solvent was evaporated and the crude mixture was purified by flash chromatography (eluent: 2% MeOH in dichloromethane) to afford the desired product as a beige solid (16 mg, yield 30%). **24:** ^{1}H NMR (500 MHz, DMSO-*d*6) δ 12.76 (s, 1H), 9.83 (s, 1H), 9.70 (s, 1H), 9.04 (d, *J* = 12.8 Hz, 2H), 8.46 (d, *J* = 8.4 Hz, 1H), 8.00 (t, *J* = 5.4 Hz, 1H), 7.95 (s, 1H), 7.81 (dd, *J* = 14.8, 7.4 Hz, 2H), 7.75 – 7.72 (m, 1H), 7.64 – 7.55 (m, 2H), 7.3 – 7.27 (m, 1H), 6.91 (d, *J* = 8.8 Hz, 2H), 5.20 (dd, *J* = 13.0, 5.3 Hz, 1H), 3.99 (d, *J* = 5.2 Hz, 2H), 3.58 (d, *J* = 14.9 Hz, 4H), 3.42 (m, 1H), 3.04 (m, 7H), 2.98 – 2.90 (m, 1H), 2.76 (d, *J* = 16.8 Hz, 1H), 2.59 – 2.50 (m, 2H), 2.24 (t, *J* = 7.2 Hz, 2H), 2.06 (m, 1H), 1.89 – 1.81 (m, 2H), 1.17 (d, *J* = 6.7 Hz, 6H); ^{13}C NMR (126 MHz, DMSO-*d*6) δ 172.3, 172.2, 169.9, 168.0, 167.5, 167.1, 156.5, 152.8, 150.9, 146.1, 139.7, 138.9, 136.9, 136.5, 135.6, 134.3, 131.9, 131.4, 126.9, 122.7, 122.6, 122.5, 120.9, 118.8, 117.5, 117.1, 115.4, 110.0, 55.6, 50.2, 49.9, 49.8, 44.5, 41.8, 40.8, 36.2, 34.7, 31.5, 27.1, 21.6, 21.5, 15.4; ESI-HRMS m/z for $C_{45}H_{49}N_{12}O_9S$ [M+H]$^+$ calcd 933.3461, found 933.3500.

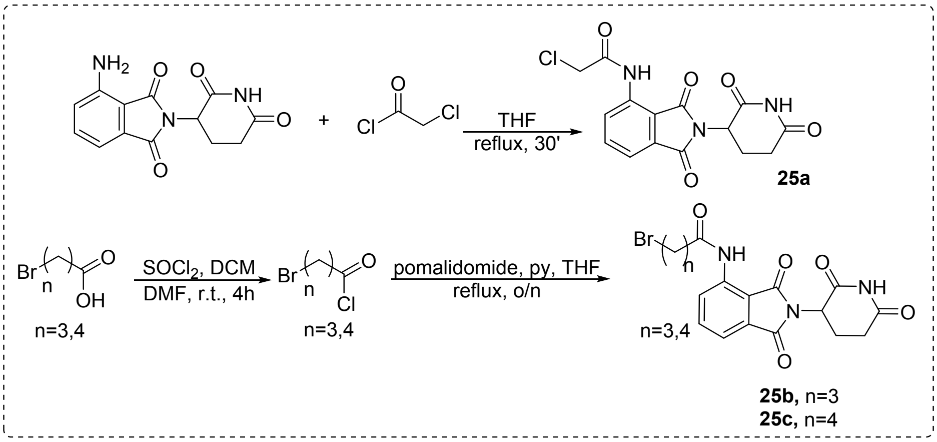

Scheme S4. Synthesis of pomalidomide derivatives

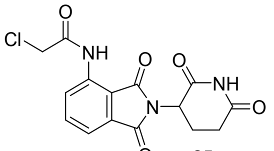

25a

2-chloro-*N*-(2-(2,6-dioxopiperidin-3-yl)-1,3-dioxoisoindolin-4-yl)acetamide, 25a: In a self-sealing tube containing 4-amino-2-(2,6-dioxopiperidin-3-yl)isoindoline-1,3-dione (160 mg, 0.589 mmol) as a suspension in THF (3 mL), chloroacetyl chloride (52 µL, 0.648 mmol) was added under inert atmosphere and the reaction mixture was heated under reflux conditions for 30 minutes. Solvent was removed under reduced pressure and the obtained solid was suspended in Et_2O and filtered to afford the desired product as a yellowish solid which was used onto the next step without any further purification (183 mg, yield 89%). The spectral data were in accordance with those reported in the literature.[4] **25a:** ^1H NMR (500 MHz, DMSO-d_6) δ 11.15 (s, 1H), 10.31 (s, 1H), 8.54 (dd, *J* = 8.4, 0.7 Hz, 1H), 7.88 (dd, *J* = 8.4, 7.8 Hz, 1H), 7.68 (d, *J* = 7.2 Hz, 1H), 5.17 (dd, *J* = 12.8, 5.3 Hz, 1H), 4.53 (s, 2H), 2.94 – 2.85 (m, 1H), 2.59 (dd, *J* = 23.9, 14.1 Hz, 2H), 2.11 – 2.03 (m, 1H).

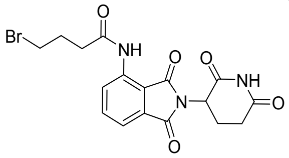

25b

4-bromo-*N*-(2-(2,6-dioxopiperidin-3-yl)-1,3-dioxoisoindolin-4-yl)butanamide, 25b: Into a 250-mL round-bottom flask, was placed 4-bromobutanoic acid (100 mg, 0.598 mmol), dichloromethane (1 mL), *N*,*N*-dimethylformamide (0.005 mL). This was followed by the addition of thionyl chloride (0.085 mL) dropwise at 0°C. The resulting solution was stirred for 4h at room temperature. The resulting mixture was concentrated under vacuum affording the bromobutanoyl chloride as light yellow oil with 100% yield.[7] To a solution of 4-amino-2-(2,6-dioxopiperidin-3-yl)isoindoline-1,3-dione (58 mg, 0.272 mmol) in dry THF (1 mL) was added into 4-bromobutanoyl chloride (111 mg, 0.598 mmol). The reaction was heated to reflux for 12h. The reaction mixture was concentrated, and the residue was extracted with DCM. The organic layer was washed with saturated $NaHCO_3$ and dried with anhydrous Na_2SO_4. The residue was purified by chromatography (DCM/Acetone, 15:1) to give the desired bromide **25b** as s white solid (46 mg, 40% yield).[8] **25b**: ^1H NMR (500 MHz, $CDCl_3$) δ 9.43 (s, 1H), 8.80 (d, *J* = 8.5 Hz, 1H), 8.37 (s, 1H), 7.71 (t, *J* = 7.9 Hz, 1H), 7.55 (d, *J* = 7.3 Hz, 1H), 4.96 (dd, *J* = 12.2, 5.3 Hz, 1H), 3.66 (t, *J* = 6.2 Hz, 1H), 3.52 (t, *J* = 6.3 Hz, 1H), 2.79 (m, 3H), 2.67 (t, *J* = 7.1 Hz, 2H), 2.30 – 2.03 (m, 3H).

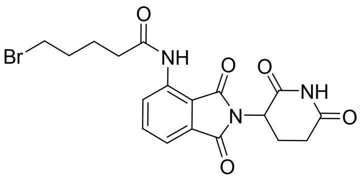

25c

5-bromo-*N*-(2-(2,6-dioxopiperidin-3-yl)-1,3-dioxoisoindolin-4-yl)pentanamide, 25c: Into a 250-mL round-bottom flask, was placed 5-bromopentanoic acid (150 mg, 0.829 mmol), dichloromethane (1.4 mL), *N*,*N*-dimethylformamide (0.006 mL). This was followed by the addition of thionyl chloride (0.12 mL) dropwise at 0°C. The resulting solution was stirred for 4h at room temperature. The resulting mixture was concentrated under vacuum affording the bromopentanoyl chloride as light yellow oil with 100% yield.[7] To a solution of 4-amino-2-(2,6-dioxopiperidin-3-yl)isoindoline-1,3-dione (80 mg, 0.376 mmol) in dry THF (1.3 mL) was added into 5-bromopentanoyl chloride (165 mg, 0.829 mmol). The reaction was heated to reflux for 12h. The reaction

mixture was concentrated, and the residue was extracted with DCM. The organic layer was washed with saturated $NaHCO_3$ and dried with anhydrous Na_2SO_4. The residue was purified by chromatography (DCM/Acetone, 10:1) to give the desired bromide **25c** as s white solid (81 mg, 50% yield).[8] **25c:** ^{1}H NMR (500 MHz, $CDCl_3$) δ 9.42 (s, 1H), 8.82 (d, *J* = 8.43 Hz, 1H), 8.44 (s, 1H), 7.72 (t, *J* = 7.74 Hz, 1H), 7.55 (d, *J* = 7.26 Hz, 1H), 4.96 (dd, *J* = 12.1, 4.9 Hz, 1H). 3.59 (t, *J* = 5.5 Hz, 1H), 3.45 (t, *J* = 6.3 Hz, 1H), 2.75-2.95 (m, 3H), 2.51 (t, *J* = 6.6 Hz, 2H), 2.15-2.21 (m, 1H), 1.86-2.00 (m, 4H).

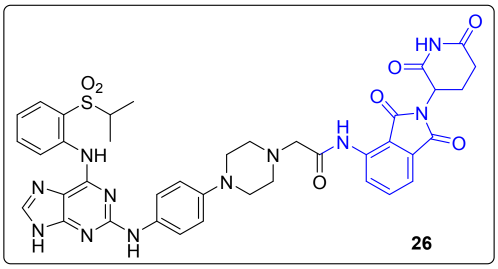

***N*-(2-(2,6-dioxopiperidin-3-yl)-1,3-dioxoisoindolin-4-yl)-2-(4-(4-((6-((2-(isopropylsulfonyl)phenyl)amino)-9*H*-purin-2-yl)amino)phenyl)piperazin-1-yl)acetamide, 26:** In a dried flask containing *N*6-(2-(isopropylsulfonyl)phenyl)-*N*2-(4-(piperazin-1-yl)phenyl)-9*H*-purine-2,6-diamine (29 mg, 0.058 mmol) in dry DMF (0.8 mL), dry DIPEA (38 mg, 0.158 mmol) was added, followed by the addition of 2-chloro-*N*-(2-(2,6-dioxopiperidin-3-yl)-1,3-dioxoisoindolin-4-yl)acetamide (20 mg, 0.058 mmol) and the reaction was stirred in ambient temperature for 48 hours. Upon completion of the reaction the solvent was removed under removed pressure and the desired product was crystallized from the crude mixture with a mixture of dichloromethane/MeOH as a beige solid (15 mg, 32% yield). **26:** ^{1}H NMR (500 MHz, DMSO-*d*6) δ 12.73 (s, 1H), 11.09 (s, 1H), 11.03 (s, 1H), 9.85 (s, 1H), 9.07 (d, *J* = 8.2 Hz, 1H), 9.01 (s, 1H), 8.83 (d, *J* = 8.5 Hz, 1H), 7.96 (s, 1H), 7.86-7.82 (m, 2H), 7.74 (m, 1H), 7.60 (d, *J* = 7.7 Hz, 2H), 7.30 – 7.29 (m, 1H), 6.93 (d, *J* = 8.8 Hz, 2H), 5.13 (dd, *J* = 12.6, 5.3 Hz, 1H), 3.43 (m, 2H), 3.30 (s, 1H), 3.27 (m, 4H), 2.90 – 2.81 (m, 2H), 2.76 (m, 4H), 2.54 (m, 2H), 2.05 (m, 1H), 1.19 (d, *J* = 6.7 Hz, 6H); ^{13}C NMR (126 MHz, DMSO-*d*6) δ 173.2, 170.5, 170.3, 168.5, 167.3, 156.7, 152.7, 150.9, 146.2, 139.7, 138.8, 136.9, 136.8, 135.6, 133.7, 131.8, 131.4, 124.7, 122.7, 122.6, 122.5, 121.1, 118.4, 116.2, 115.4, 109.9, 61.9, 55.7, 53.5, 49.3, 31.3, 22.3, 15.4. one carbon missing due to overlapping; ESI-HRMS m/z for $C_{39}H_{40}N_{11}O_7S$ $[M+H]^+$ calcd 806.2833, found 806.2830.

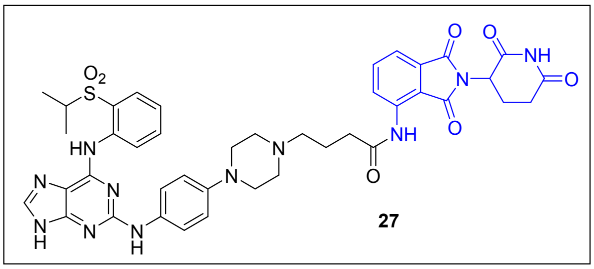

***N*-(2-(2,6-dioxopiperidin-3-yl)-1,3-dioxoisoindolin-4-yl)-4-(4-(4-((6-((2-(isopropylsulfonyl)phenyl)amino)-9*H*-purin-2-yl)amino)phenyl)piperazin-1-yl)butanamide, 27:** In a dried flask containing *N*6-(2-(isopropylsulfonyl)phenyl)-*N*2-(4-(piperazin-1-yl)phenyl)-9*H*-purine-2,6-diamine (26 mg, 0.053 mmol) in dry DMF (0.7 mL), dry DIPEA (20 mg, 0.15 mmol) was added, followed by the addition of 4-chloro-*N*-(2-(2,6-dioxopiperidin-3-yl)-1,3-dioxoisoindolin-4-yl)butanamide (20 mg, 0.053 mmol) and the reaction was stirred in ambient temperature for 48 hours. Upon completion of the reaction the solvent was removed under removed pressure and the crude mixture was purified via preparative HPLC to afford the desired product as a beige solid (8 mg, 18%). **27:** ^{1}H NMR (300 MHz, DMSO-*d*6) δ 11.16 (s, 1H), 9.84 (s, 1H), 9.72 (s, 1H), 9.06 (d, *J* = 8.3 Hz, 1H), 8.98 (s, 1H), 8.50 (d, *J* = 8.4 Hz, 1H), 8.34 (br, 1H), 7.96 (s, 1H), 7.82 (m, 2H), 7.72 (m, 1H), 7.57 (m, 3H), 7.30 – 7.27 (m, 1H), 6.85 (d, *J* = 8.9 Hz, 2H), 5.14 (dd, *J* = 12.8, 5.3 Hz, 1H), 3.50 (m, 4H), 3.03 (br, 4H), 2.96 – 2.82 (m, 2H), 2.59 (m, 2H), 2.40 (t, *J* = 6.5 Hz, 2H), 2.11 – 1.96 (m, 2H), 1.92 – 1.77 (m, 2H), 1.18 (d, *J* = 6.8 Hz,

6H); ^{13}C NMR (75 MHz, DMSO-*d*6) δ 173.2, 172.5, 170.2, 168.2, 167.1, 156.7, 151.0, 150.9, 146.5, 139.7, 138.8, 137.1, 136.6, 135.6, 133.6, 131.9, 131.4, 126.7, 122.7, 122.6, 122.6, 122.5, 121.0, 118.7, 117.3, 116.3, 57.4, 55.7, 53.2, 49.6, 49.4, 35.2, 31.4, 22.5, 22.5, 15.4; ESI-HRMS m/z for $C_{41}H_{44}N_{11}O_7S$ $[M+H]^+$ calcd 834.3146, found 834.3140.

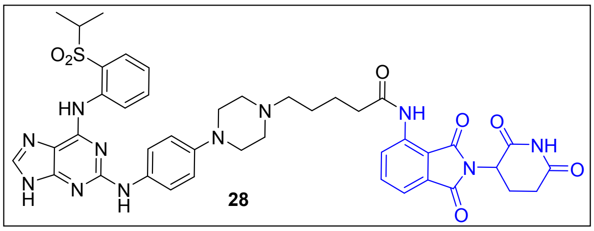

_N_-(2-(2,6-dioxopiperidin-3-yl)-1,3-dioxoisoindolin-4-yl)-5-(4-(4-((6-((2-(isopropylsulfonyl)phenyl)amino)-9_H_-purin-2-yl)amino)phenyl)piperazin-1-yl)pentanamide, 28: In a dried flask containing *N*6-(2-(isopropylsulfonyl)phenyl)-*N*2-(4-(piperazin-1-yl)phenyl)-9*H*-purine-2,6-diamine, **SF-1** (29 mg, 0.058 mmol) in dry DMF (0.8 mL), dry DIPEA (38 mg, 0.158 mmol) was added, followed by the addition of 5-chloro-*N*-(2-(2,6-dioxopiperidin-3-yl)-1,3-dioxoisoindolin-4-yl)pentanamide (20 mg, 0.058 mmol) and the reaction was stirred in ambient temperature for 48 hours. Upon completion of the reaction the solvent was removed under reduced pressure and the crude mixture was purified by flash chromatography (eluent: 5% MeOH in dichloromethane) to afford the desired product as an orange solid (15 mg, 32% over retrieved starting material). **28:** ^{1}H NMR (500 MHz, Acetone-*d*6) δ 11.66 (s, 1H), 9.99 (s, 1H), 9.53 (s, 1H), 9.09 (m, 1H), 8.82 (d, *J* = 8.5 Hz, 1H), 8.27 (s, 1H), 7.90 (s, 1H), 7.88 – 7.82 (m, 2H), 7.69 – 7.65 (m, 1H), 7.63 (d, J = 8.8 Hz, 2H), 7.56 (d, J = 7.3 Hz, 1H), 7.28 – 7.25 (m, 1H), 6.92 (d, *J* = 8.8 Hz, 2H), 5.15 (dd, *J* = 12.9, 5.4 Hz, 1H), 3.42 (m, 1H), 3.13 (m, 4H), 3.00 – 2.93 (m, 1H), 2.80 – 2.70 (m, 2H), 2.62 (t, *J* = 7.3 Hz, 2H), 2.57 (m, 4H), 2.44 (t, *J* = 6.7 Hz, 2H), 2.27 – 2.15 (m, 2H), 1.83 (m, 2H), 1.65 (m, 2H), 1.28 (d, *J* = 6.8 Hz, 6H); ^{13}C NMR (126 MHz, DMSO-*d*6) δ 172.9, 172.1, 169.9, 167.8, 166.8, 156.3, 152.3, 150.6, 146.2, 139.3, 138.4, 136.6, 136.2, 135.2, 133.2, 131.5, 131.0, 126.5, 122.2, 122.2, 120.7, 118.5, 117.1, 115.9, 115.0, 109.6, 57.5, 55.3, 52.9, 49.2, 48.9, 36.4, 31.0, 25.7, 22.9, 22.1, 15.0; ESI-MS m/z for $C_{42}H_{46}N_{11}O_7S$ $[M+H]^+$ calcd 848.33, found 848.15.

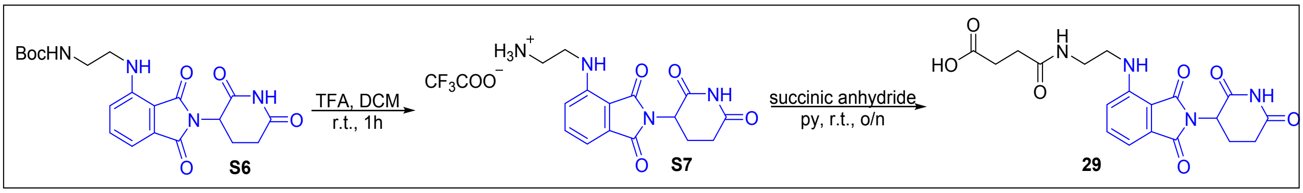

Scheme S5

4-((2-((2-(2,6-dioxopiperidin-3-yl)-1,3-dioxoisoindolin-4-yl)amino)ethyl)amino)-4-oxobutanoic acid (29): *Tert*-butyl (2-((2-(2,6-dioxopiperidin-3-yl)-1,3-dioxoisoindolin-4-yl)amino)ethyl)carbamate **S6** (21 mg, 0.05 mmol) was dissolved in a mixture of dichloromethane/TFA 3/1 (0.9 mL) and the solution was stirred for 2h at ambient temperature. Afterwards the solvents were removed in rotary to afford the desired salt which was used in the next step without further purification. In a flask containing 2-((2-(2,6-dioxopiperidin-3-yl)-1,3-dioxoisoindolin-4-yl)amino)ethan-1-aminium 2,2,2-trifluoroacetate **S7** (36 mg, 0.084 mmol) as a solution in dry pyridine (200 μL) and under inert atmosphere, succinic anhydride (17 mg, 0.167 mmol) was added and the reaction stirred overnight at room temperature. Pyridine was removed under reduced pressure and the crude mixture was purified via silica gel chromatography (eluent; 2-10% MeOH in dichloromethane) to give the desire product as a white solid (30 mg, yield 86%). **29:** ^{1}H NMR (500 MHz, acetone-*d*6) δ 9.91 (s, 1H), 7.62 – 7.54 (m, 2H), 7.21 (d, *J* = 8.6 Hz, 1H), 7.04 (d, *J* = 7.1 Hz, 1H), 6.61 (s, 1H), 5.06 (dd, *J* = 12.5, 5.4 Hz, 1H), 3.54 –

3.51 (m, 2H), 3.49 – 3.45 (m, 2H), 2.96 (m, 1H), 2.76 (m, 2H), 2.58 (m, 2H), 2.47 (t, *J* = 6.9 Hz, 2H), 2.23 – 2.17 (m, 1H); ^{13}C NMR (126 MHz, Acetone-d6) δ 173.2, 172.1, 171.8, 169.4, 169.2, 167.3, 146.9, 136.0, 132.7, 116.8, 110.8, 110.2, 48.9, 41.7, 38.7, 31.1, 30.1, 22.5, one carbon missing due to overlapping with acetone-*d6*.

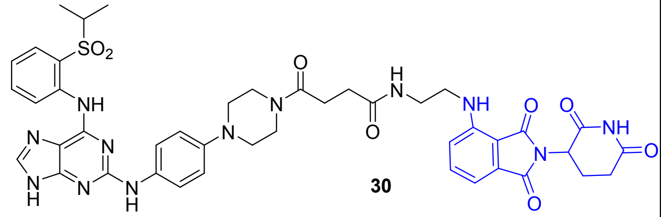

30

***N-(*2-((2-(2,6-dioxopiperidin-3-yl)-1,3-dioxoisoindolin-4-yl)amino)ethyl)-4-(4-(4-((6-((2-(isopropylsulfonyl)phenyl)amino)-9*H*-purin-2-yl)amino)phenyl)piperazin-1-yl)-4-oxobutanamide, 30:** In a dried flask containing 4-((2-((2-(2,6-dioxopiperidin-3-yl)-1,3-dioxoisoindolin-4-yl)amino)ethyl)amino)-4-oxobutanoic acid **29** (25 mg, 0.06 mmol) dry DMF (1.5 mL), 4-(4-((6-((2-(isopropylsulfonyl)phenyl)amino)-9H-purin-2-yl)amino)phenyl)piperazin-1-ium 2,2,2-trifluoroacetate **SF-1** (40 mg, 0.066 mmol), HOBt (13 mg, 0.09 mmol), EDCI (17 mg, 0.09 mmol) and triethylamine (18 mg, 0.18 mmol) were added under Ar atmosphere. The reaction mixture was stirred overnight at room temperature. After the reaction's completion the solvent was evaporated and the crude mixture was purified by flash chromatography (eluent: 2% MeOH in dichloromethane) to afford the desired product as a yellow solid (20 mg, yield 37%). **30:** ^{1}H NMR (500 MHz, DMSO-*d6*) δ 12.76 (br, 1H), 11.11 (s, 1H), 9.85 (s, 1H), 9.07 – 9.04 (m, 2H), 8.11 (t, *J* = 5.6 Hz, 1H), 7.97 (s, 1H), 7.81 (dd, *J* = 7.9, 1.2 Hz, 1H), 7.73 (m, 1H), 7.60 – 7.55 (m, 2H), 7.30 – 7.27 (m, 1H), 7.18 (d, *J* = 8.7 Hz, 1H), 7.03 (d, *J* = 7.0 Hz, 1H), 6.92 (d, *J* = 8.9 Hz, 2H), 6.75 (dd, *J* = 6.0, 6.2 Hz, 1H), 5.06 (dd, *J* = 12.7, 5.4 Hz, 1H), 3.59 (m, 4H), 3.43 (m, 2H), 3.26 – 3.22 (m, 2H), 3.03 (d, *J* = 33.6 Hz, 4H), 2.88 (m, 1H), 2.77 – 2.64 (m, 2H), 2.61 – 2.51 (m, 4H), 2.33 (t, *J* = 7.0 Hz, 2H), 2.05 – 1.99 (m, 1H), 1.18 (d, *J* = 6.8 Hz, 6H); ^{13}C NMR (126 MHz, DMSO-*d6*) δ 173.3, 172.5, 171.7, 170.6, 170.2, 167.8, 156.6, 152.7, 150.9, 146.8, 146.2, 139.7, 138.8, 136.6, 135.6, 134.3, 132.6, 131.4, 122.7, 122.5, 120.9, 117.6, 117.0, 115.4, 110.9, 109.7, 55.7, 50.2, 49.8, 48.9, 43.2, 41.8, 41.5, 38.6, 31.4, 30.9, 28.3, 22.6, 15.4; ESI-HRMS m/z for $C_{43}H_{47}N_{12}O_8S$ $[M+H]^+$ calcd 891.3355, found 891.3383.

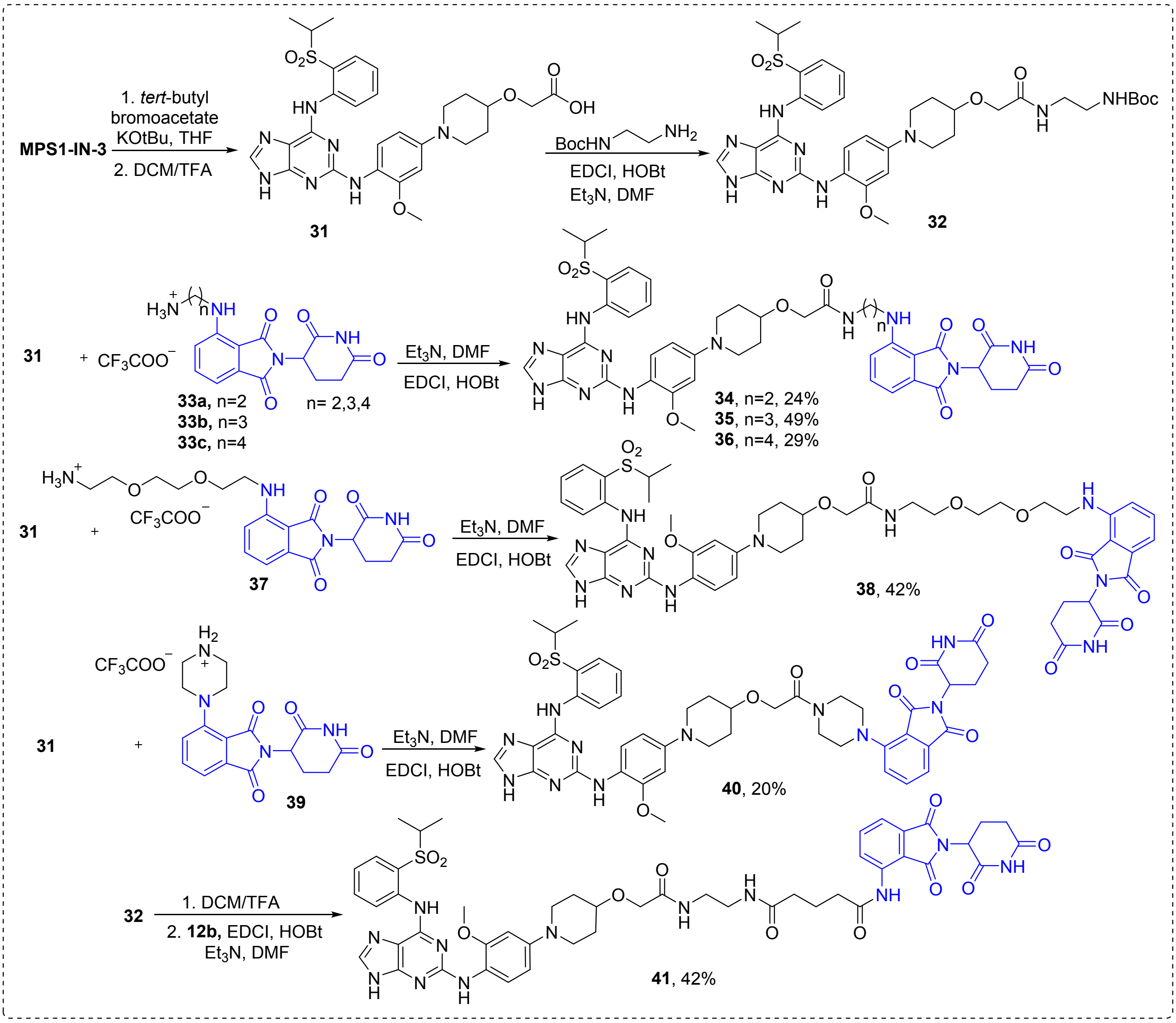

Scheme S6. Synthesis of MPS1-IN-3 based chimeras containing pomalidomide.

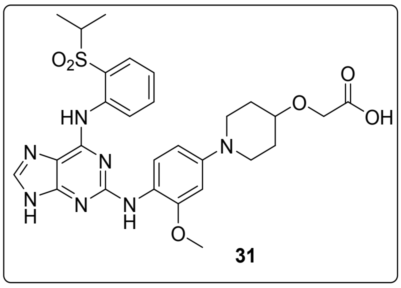

2-((1-(4-((6-((2-(isopropylsulfonyl)phenyl)amino)-9*H*-purin-2-yl)amino)-3-methoxyphenyl)piperidin-4-yl)oxy)acetic acid, 31: In a dried flask potassium *tert*-butoxide (22 mg, 0.19 mmol) was suspended in dry THF (0.4 mL) and the suspension was cooled to 0 °C. Then 1-(4-((6-((2-(isopropylsulfonyl)phenyl)amino)-9-(tetrahydro-2*H*-pyran-2-yl)-9*H*-purin-2-yl)amino)-3-methoxyphenyl)piperidin-4-ol **MPS1-IN-3** (40 mg, 0.064 mmol) was added and the mixture was heated at 40 °C for 30 minutes. Finally, the mixture was again cooled to 0 °C and *tert*-butyl-bromoacetate (38 mg, 0.19 mmol) was added dropwise. The reaction mixture was left stirring at r.t. for 14h. The reaction was monitored by LC/MS and upon completion the mixture was extracted

with water and EtOAc. The organic layer was dried over anhydrous Na_2SO_4 and filtered. The solvent was evaporated to afford *tert*-butyl 2-((1-(4-((6-((2-(isopropylsulfonyl)phenyl)amino)-9-(tetrahydro-2*H*-pyran-2-yl)-9*H*-purin-2-yl)amino)-3-methoxyphenyl)piperidin-4-yl)oxy)acetate **S8,** which was used in the next step without further purification. **S8:** ESI-HRMS m/z for $C_{37}H_{50}N_7O_7S$ $[M+H]^+$ calcd 736.3492, found 736.3474. **S8** (30 mg, 0.016 mmol) was dissolved in a mixture of 3/1 dichoromethane and TFA (0.4 mL) and the reaction mixture was stirred at ambient temperature for 2h. Afterwards the solvents were removed under reduced pressure to afford the desired acid **31**. **31:** ESI-HRMS m/z for $C_{28}H_{34}N_7O_6S$ $[M+H]^+$ calcd 596,2291, found 596,2275.

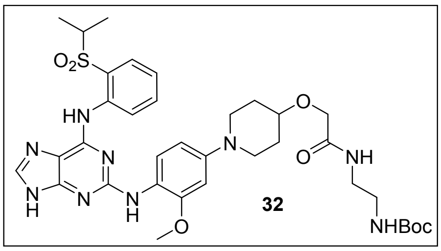

***tert*-butyl (2-(2-((1-(4-((6-((2-(isopropylsulfonyl)phenyl)amino)-9H-purin-2-yl)amino)-3-methoxyphenyl)piperidin-4-yl)oxy)acetamido)ethyl)carbamate, 32:** In a dried flask containing 2-((1-(4-((6-((2-(isopropylsulfonyl)phenyl)amino)-9*H*-purin-2-yl)amino)-3-methoxyphenyl)piperidin-4-yl)oxy)acetic acid **31** (30 mg, 0.05 mmol) dry DMF (1.1 mL), *tert*-butyl (2-aminoethyl)carbamate (9 mg, 0.055 mmol), HOBt (10 mg, 0.076 mmol), EDCI (15 mg, 0.076 mmol) and triethylamine (15 mg, 0.15 mmol) were added under Ar atmosphere. The reaction mixture was stirred overnight at room temperature. After the reaction's completion the solvent was evaporated and the crude mixture was purified by flash chromatography (eluent: 2% MeOH in dichloromethane) to afford the desired product as a yellow oil (23 mg, yield 62%). **32:** ^1H NMR (300 MHz, $CDCl_3$) δ 8.10 (d, *J* = 7.6 Hz, 1H), 7.88 (br, 1H), 7.76 (br, 1H), 7.63 – 7.58 (m, 1H), 6.83 (br, 1H), 6.48 (s, 1H), 6.13 (br, 1H), 5.53 (br, 1H), 5.30 – 5.28 (m, 1H), 4.41 (d, *J* = 16.9 Hz, 1H), 3.91 – 3.82 (m, 1H), 3.80 (s, 3H), 3.41 (d, *J* = 6.5 Hz, 4H), 3.27 – 3.17 (m, 3H), 3.13 – 3.02 (m, 1H), 2.85 (m, 2H), 2.01 – 1.98 (m, 4H), 1.84 – 1.63 (m, 4H), 1.39 (s, 9H), 0.92 (d, *J* = 6.7 Hz, 6H); ^{13}C NMR (75 MHz, $CDCl_3$) δ 170.3, 156.5, 155.8, 155.7, 153.9, 153.8, 144.6, 144.4, 136.4, 135.5, 135.1, 135.1, 133.9, 132.3, 130.7, 130.2, 128.3, 128.2, 128.2, 114.8, 108.5, 100.9, 79.6, 67.6, 56.6, 55.8, 54.0, 48.5, 40.2, 39.9, 34.0, 29.8, 28.5, 16.8, 13.3; ESI-HRMS m/z for $C_{35}H_{48}N_9O_7S$ $[M+H]^+$ calcd 738.3397, found 738.3379.

General procedure for synthesis of 33a-c, 37

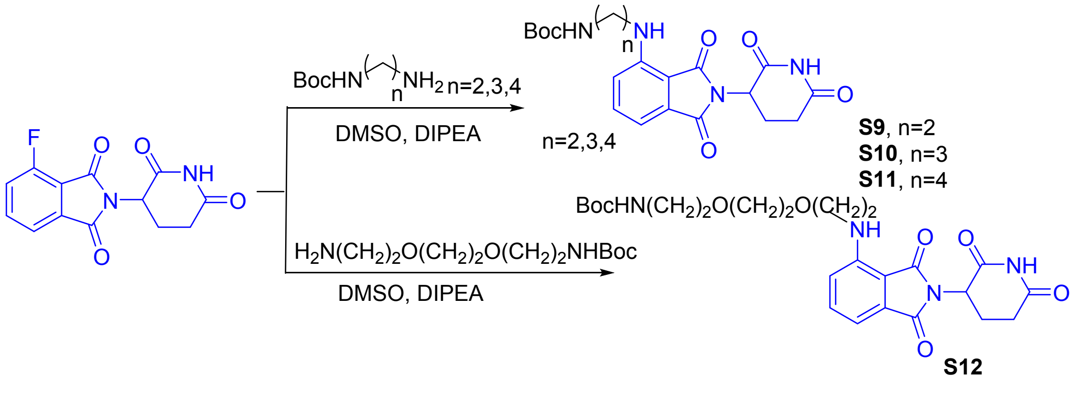

Scheme S7

The appropriate mono-Boc protected diamine (1.2 eq) was added to a stirred solution of 2-(2,6-dioxopiperidin-3-yl)-4-fluoroisoindoline-1,3-dione (1.0 eq) in DMSO (1 M) and DIPEA (3.0 eq). The reaction mixture was stirred

at 130°C for 1-3h. Then the mixture was cooled to room temperature and the addition of water resulted in the precipitation of a green solid which was filtered off and washed with cold water to afford the desired product in good to high yields (60-90%). The spectral data were in accordance with those reported in the literature.[9,4,10]

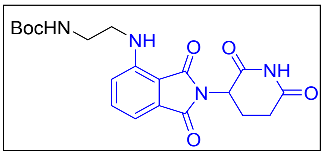

S9: ^{1}H NMR (500 MHz, acetone-d_6) δ 9.9 (br, 1H), 7.59 (dd, *J* = 8.6, 7.1 Hz, 1H), 7.19 (d, *J* = 8.6 Hz, 1H), 7.04 (d, *J* = 7.1 Hz, 1H), 6.61 (s, 1H), 6.33 (s, 1H), 5.06 (dd, *J* = 12.5, 5.4 Hz, 1H), 3.52 (q, *J* = 6.3 Hz, 2H), 3.35 (q, *J* = 6.2 Hz, 2H), 3.02 – 2.88 (m, 1H), 2.75 (m, 2H), 2.05 (m, 1H), 1.40 (s, 9H).

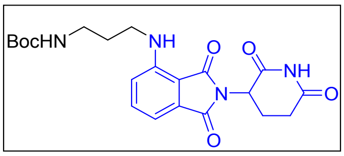

S10: ^{1}H NMR (500 MHz, $CDCl_3$) δ 8.54 (s, 1H), 7.47 (dd, *J* = 7.8 Hz, 1H), 7.07 (d, *J* = 7.0 Hz, 1H), 6.87 (d, *J* = 8.5 Hz, 1H), 6.31 (br, 1H), 4.91 (dd, *J* = 12.1, 5.1 Hz, 1H), 4.76 (br, 1H), 3.32 (dd, *J* = 12.7, 6.4 Hz, 2H), 3.23 (m, 2H), 2.87 (m, 1H), 2.83 – 2.67 (m, 2H), 2.14 – 2.08 (m, 1H), 1.86 – 1.78 (m, 2H), 1.43 (s, 9H).

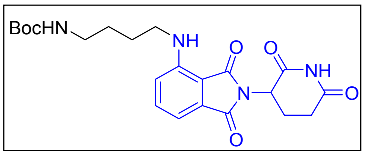

S11: H NMR (500 MHz, $CDCl_3$) δ 8.62 (s, 1H), 7.48 (dd, J = 7.8 Hz, 1H), 7.08 (d, J = 7.1 Hz, 1H), 6.89 (d, J = 8.5 Hz, 1H), 6.25 (t, *J* = 5.2 Hz, 1H), 4.93 (dd, J = 12.2, 5.2 Hz, 1H), 4.70 (s, 1H), 3.30 (q, *J* = 6.1 Hz, 2H), 3.22–3.13 (m, 2H), 2.90–2.70 (m, 3H), 2.13 (dd, *J* = 13.0, 5.1 Hz, 1H), 1.69 (m, 2H), 1.60 (m, 2H), 1.45 (s, 9H).

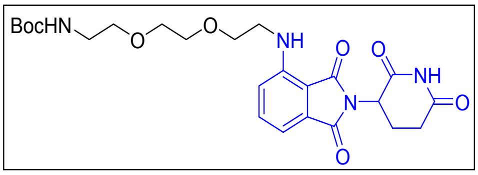

S12: ^{1}H NMR (500 MHz, $CDCl_3$) δ 8.30 (br, 1H), 7.50 (dd, *J* = 7.8, 8.5 Hz, 2H), 7.11 (d, *J* = 7.1 Hz, 1H), 6.91 (d, *J* = 8.5 Hz, 2H), 6.52 (s, 1H), 5.04 (br, 1H), 4.96 – 4.86 (m, 1H), 3.73 (t, *J* = 5.2 Hz, 2H), 3.66 (br, 4H), 3.59 – 3.55 (m, 2H), 3.48 (dd, *J* = 10.0, 4.9 Hz, 2H), 3.33 (m, 2H), 2.91 – 2.85 (m, 1H), 2.83 – 2.68 (m, 2H), 2.14 (m, 1H), 1.43 (s, 9H).

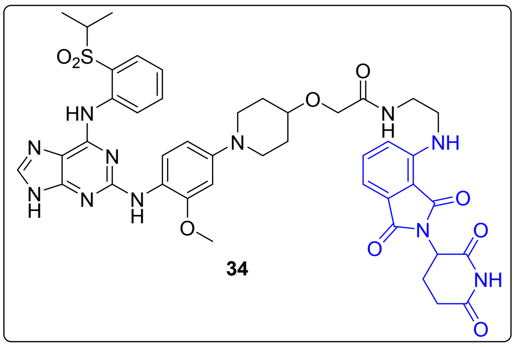

***N*-(2-((2-(2,6-dioxopiperidin-3-yl)-1,3-dioxoisoindolin-4-yl)amino)ethyl)-2-((1-(4-((6-((2-(isopropylsulfonyl)phenyl)amino)-9*H*-purin-2-yl)amino)-3-methoxyphenyl)piperidin-4-yl)oxy)acetamide, 34:** *Tert*-butyl (2-((2-(2,6-dioxopiperidin-3-yl)-1,3-dioxoisoindolin-4-yl)amino)ethyl)carbamate **S9** (21 mg, 0.053 mmol) was dissolved in a mixture of dichloromethane/TFA 3/1 (0.5 mL) and the solution was stirred for 1h at ambient temperature. Afterwards the solvents were removed in rotary to afford the desired salt which was used in the next step without further purification. In a dried flask containing 2-((1-(4-((6-((2-(isopropylsulfonyl)phenyl)amino)-9*H*-purin-2-yl)amino)-3-methoxyphenyl)piperidin-4-yl)oxy)acetic acid **31** (30 mg, 0.050 mmol) dry DMF (1.1 mL), 2-((2-(2,6-dioxopiperidin-3-yl)-1,3-dioxoisoindolin-4-yl)amino)ethan-1-aminium 2,2,2-trifluoroacetate **33a** (22 mg, 0.055 mmol), HOBt (10 mg, 0.075 mmol), EDCI (15 mg, 0.075 mmol) and triethylamine (15 mg, 0.15 mmol) were added under Ar atmosphere. The reaction mixture was stirred overnight at room temperature. After the reaction's completion the solvent was evaporated and the crude mixture was purified via preparative HPLC to afford the desired product as a mixture of diastereomers (yellow solid, 11 mg, yield 24%). **34:** ESI-HRMS m/z for $C_{43}H_{48}N_{11}O_9S$ $[M+H]^+$ calcd 894.3352, found 894.3377.

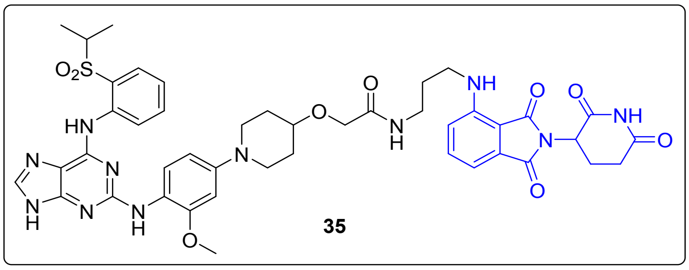

***N*-(3-((2-(2,6-dioxopiperidin-3-yl)-1,3-dioxoisoindolin-4-yl)amino)propyl)-2-((1-(4-((6-((2-(isopropylsulfonyl)phenyl)amino)-9*H*-purin-2-yl)amino)-3-methoxyphenyl)piperidin-4-yl)oxy)acetamide, 35:** *Tert*-butyl (3-((2-(2,6-dioxopiperidin-3-yl)-1,3-dioxoisoindolin-4-yl)amino)propyl)carbamate **S10** (15 mg, 0.036 mmol) was dissolved in a mixture of dichloromethane/TFA 3/1 (0.4 mL) and the solution was stirred for 1h at ambient temperature. Afterwards the solvents were removed in rotary to afford the desired salt which was used in the next step without further purification. In a dried flask containing 2-((1-(4-((6-((2-(isopropylsulfonyl)phenyl)amino)-9*H*-purin-2-yl)amino)-3-methoxyphenyl)piperidin-4-yl)oxy)acetic acid **31** (20 mg, 0.034 mmol) dry DMF (0.7 mL), 3-((2-(2,6-dioxopiperidin-3-yl)-1,3-dioxoisoindolin-4-yl)amino)propan-1-aminium 2,2,2-trifluoroacetate **33b** (16 mg, 0.037 mmol), HOBt (7 mg, 0.05 mmol), EDCI (10 mg, 0.05 mmol) and triethylamine (14 mg, 0.13 mmol) were added under Ar atmosphere. The reaction mixture was stirred overnight at room temperature. After the reaction's completion the solvent was evaporated and the crude mixture was purified via preparative HPLC to afford the desired product as a as a mixture of diastereomers (yellow solid, 15 mg, yield 49%). **35:** ESI-HRMS m/z for $C_{44}H_{50}N_{11}O_9S$ $[M+H]^+$ calcd 908.3508, found 908.3534.

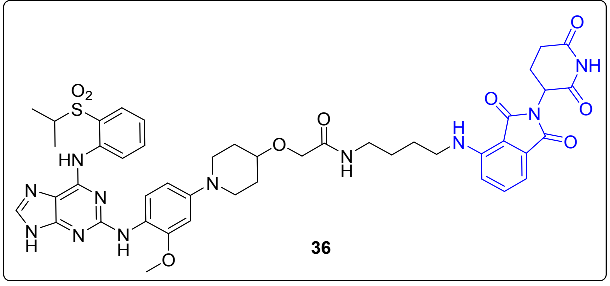

***N*-(4-((2-(2,6-dioxopiperidin-3-yl)-1,3-dioxoisoindolin-4-yl)amino)butyl)-2-((1-(4-((6-((2-(isopropylsulfonyl)phenyl)amino)-9*H*-purin-2-yl)amino)-3-methoxyphenyl)piperidin-4-yl)oxy)acetamide, 36:** *Tert*-butyl (4-((2-(2,6-dioxopiperidin-3-yl)-1,3-dioxoisoindolin-4-yl)amino)butyl)carbamate **S11** (32 mg, 0.066 mmol) was dissolved in a mixture of dichloromethane/TFA 3/1 (0.6 mL) and the solution was stirred for 1h at ambient temperature. Afterwards the solvents were removed in rotary to afford the desired salt which was used in the next step without further purification. In a dried flask containing 2-((1-(4-((6-((2-(isopropylsulfonyl)phenyl)amino)-9*H*-purin-2-yl)amino)-3-methoxyphenyl)piperidin-4-yl)oxy)acetic acid **31** (36 mg, 0.06 mmol) dry DMF (1.4 mL), 4-((2-(2,6-dioxopiperidin-3-yl)-1,3-dioxoisoindolin-4-yl)amino)butan-1-aminium 2,2,2-trifluoroacetate **33c** (32 mg, 0.067 mmol), HOBt (12 mg, 0.09 mmol), EDCI (17 mg, 0.09 mmol) and triethylamine (24 mg, 0.24 mmol) were added under Ar atmosphere. The reaction mixture was stirred overnight at room temperature. After the reaction's completion the solvent was evaporated, and the crude mixture was purified by flash chromatography (eluent: 3% MeOH in dichloromethane) to afford the desired product as a yellow solid (16 mg, yield 29%). **36:** ESI-HRMS m/z for $C_{45}H_{52}N_{11}O_9S$ $[M+H]^+$ calcd 922.3670, found 922.3659.

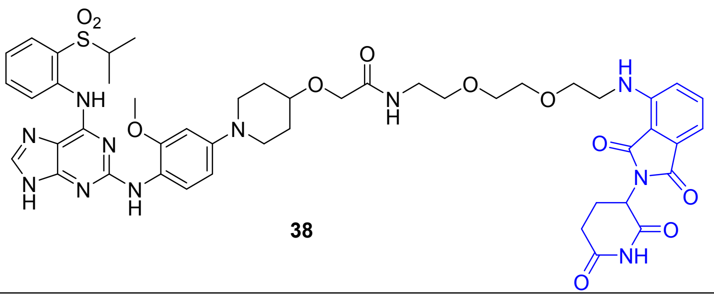

***N*-(2-(2-(2-((2-(2,6-dioxopiperidin-3-yl)-1,3-dioxoisoindolin-4-yl)amino)ethoxy)ethoxy)ethyl)-2-((1-(4-((6-((2-(isopropylsulfonyl)phenyl)amino)-9*H*-purin-2-yl)amino)-3-methoxyphenyl)piperidin-4-yl)oxy)acetamide, 38:** *Tert*-butyl (2-(2-(2-((2-(2,6-dioxopiperidin-3-yl)-1,3-dioxoisoindolin-4-yl)amino)ethoxy)ethoxy)ethyl)carbamate **S12** (27 mg, 0.05 mmol) was dissolved in a mixture of dichloromethane/TFA 3/1 (0.55 mL) and the solution was stirred for 1h at ambient temperature. Afterwards the solvents were removed in rotary to afford the desired salt which was used in the next step without further purification. In a dried flask containing 2-((1-(4-((6-((2-(isopropylsulfonyl)phenyl)amino)-9*H*-purin-2-yl)amino)-3-methoxyphenyl)piperidin-4-yl)oxy)acetic acid **31** (27 mg, 0.047 mmol) dry DMF (1.1 mL), 2-(2-(2-((2-(2,6-dioxopiperidin-3-yl)-1,3-dioxoisoindolin-4-yl)amino)ethoxy)ethoxy)ethan-1-aminium 2,2,2-trifluoroacetate **37** (27 mg, 0.051 mmol), HOBt (10 mg, 0.07 mmol), EDCI (13 mg, 0.07 mmol) and triethylamine (19 mg, 0.19 mmol) were added under Ar atmosphere. The reaction mixture was stirred overnight at room temperature. After the reaction's completion the solvent was evaporated, and the crude mixture was purified by flash chromatography (eluent: 3% MeOH in dichloromethane) to afford the desired product as a yellow solid (19 mg, yield 42%). **38:** ESI-HRMS m/z for $C_{47}H_{56}N_{11}O_{11}S$ $[M+H]^+$ calcd 982.3876, found 982.3917.

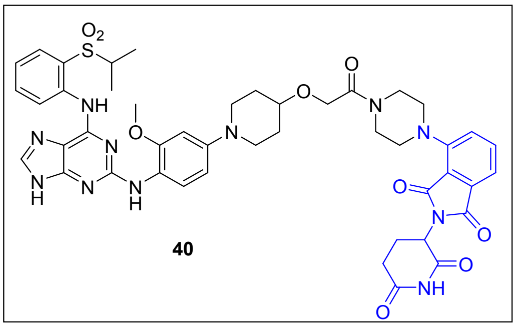

2-(2,6-dioxopiperidin-3-yl)-4-(4-(2-((1-(4-((6-((2-(isopropylsulfonyl)phenyl)amino)-9*H*-purin-2-yl)amino)-3-methoxyphenyl)piperidin-4-yl)oxy)acetyl)piperazin-1-yl)isoindoline-1,3-dione, 40: *Tert*-butyl 4-(2-(2,6-dioxopiperidin-3-yl)-1,3-dioxoisoindolin-4-yl)piperazine-1-carboxylate **S13 (**24 mg, 0.053 mmol) was dissolved in a mixture of dichloromethane/TFA 3/1 (0.5 mL) and the solution was stirred for 1h at ambient temperature. Afterwards the solvents were removed in rotary to afford the desired salt which was used in the next step without further purification. In a dried flask containing 2-((1-(4-((6-((2-(isopropylsulfonyl)phenyl)amino)-9*H*-purin-2-yl)amino)-3-methoxyphenyl)piperidin-4-yl)oxy)acetic acid **31** (27 mg, 0.045 mmol) dry DMF (0.65 mL), 4-(2-(2,6-dioxopiperidin-3-yl)-1,3-dioxoisoindolin-4-yl)piperazin-1-ium 2,2,2-trifluoroacetate **39** (25 mg, 0.055 mmol), HOBt (12 mg, 0.09 mmol), EDCI (17 mg, 0.09 mmol) and triethylamine (24 mg, 0.14 mmol) were added under Ar atmosphere. The reaction mixture was stirred overnight at room temperature. After the reaction's completion the solvent was evaporated and the crude mixture was purified by flash chromatography (eluent: 5% MeOH in dichloromethane) to afford the desired product as a yellow solid (8.2 mg, yield 20%). **40:** ESI-HRMS m/z for $C_{45}H_{50}N_{11}O_9S$ $[M+H]^+$ calcd 920.3514, found 920.3511.

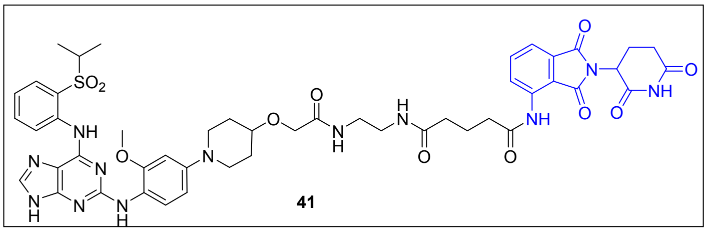

***N*1-(2-(2,6-dioxopiperidin-3-yl)-1,3-dioxoisoindolin-4-yl)-N5-(2-(2-((1-(4-((6-((2-(isopropylsulfonyl)phenyl)amino)-9*H*-purin-2-yl)amino)-3-methoxyphenyl)piperidin-4-yl)oxy)acetamido)ethyl)glutaramide, 41:** *Tert*-butyl (2-(2-((1-(4-((6-((2-(isopropylsulfonyl)phenyl)amino)-9*H*-purin-2-yl)amino)-3-methoxyphenyl)piperidin-4-yl)oxy)acetamido)ethyl)carbamate (22 mg, 0.03 mmol) was dissolved in a mixture of dichloromethane/TFA 3/1 (0.5 mL) and the solution was stirred for 2h at ambient temperature. Afterwards the solvents were removed in rotary to afford the desired salt which was used in the next step without further purification. In a dried flask containing 5-((2-(2,6-dioxopiperidin-3-yl)-1,3-dioxoisoindolin-4-yl)amino)-5-oxopentanoic acid **12c** (11 mg, 0.028 mmol) dry DMF (0.6 mL), 2-(2-((1-(4-((6-((2-(isopropylsulfonyl)phenyl)amino)-9*H*-purin-2-yl)amino)-3-methoxyphenyl)piperidin-4-yl)oxy)acetamido) ethan-1-aminium 2,2,2-trifluoroacetate **32** (23 mg, 0.031 mmol), HOBt (6 mg, 0.042 mmol), EDCI (8 mg, 0.042 mmol) and triethylamine (11 mg, 0.111 mmol) were added under Ar atmosphere. The reaction mixture was stirred overnight at room temperature. After the reaction's completion the solvent was evaporated, and the crude mixture was purified by flash chromatography (eluent: 3% MeOH in dichloromethane) to afford the desired product as a beige solid (13 mg, yield 42%). **41:** ESI-HRMS m/z for $C_{48}H_{55}N_{12}O_{11}S$ $[M/2+H]^+$ calcd 504.1956, found 504.1948.

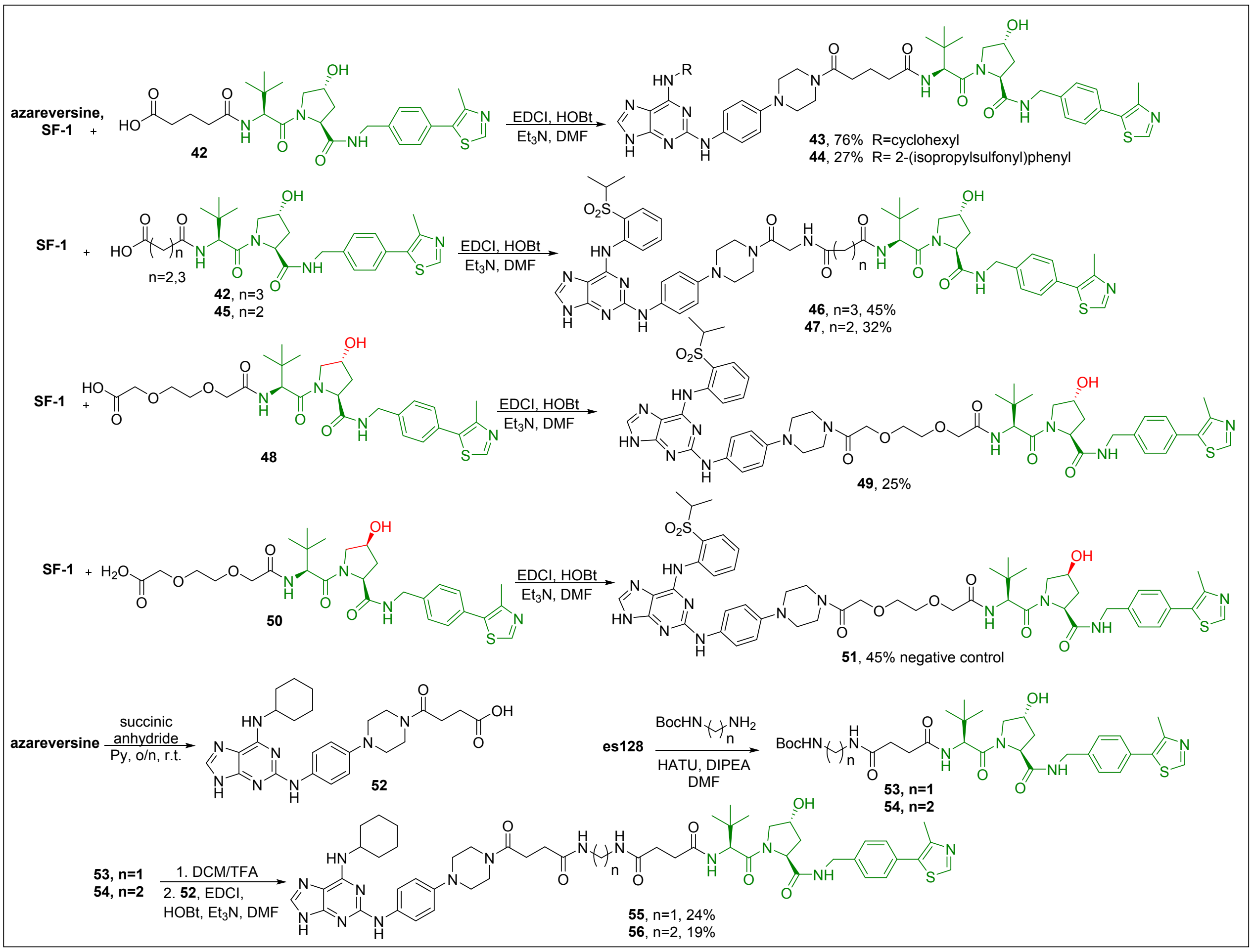

Scheme S8. Synthesis of chimeras containing VHL-1.

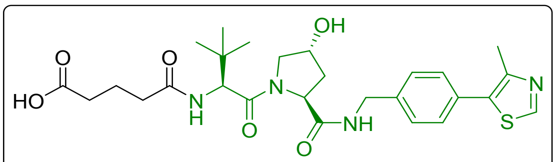

42

5-(((*S*)-1-((2*S*,4*R*)-4-hydroxy-2-((4-(4-methylthiazol-5-yl)benzyl)carbamoyl)pyrrolidin-1-yl)-3,3-dimethyl-1-oxobutan-2-yl)amino)-5-oxopentanoic acid, 42: In a flask containing (*S*)-1-((2*S*,4*R*)-4-hydroxy-2-((4-(4-methylthiazol-5-yl)benzyl)carbamoyl)pyrrolidin-1-yl)-3,3-dimethyl-1-oxobutan-2-aminium 2,2,2-trifluoroacetate **S14** (18.6 mg, 0.034 mmol) as a solution in dry pyridine (300 µL) and under inert atmosphere, glutaric anhydride (7.8 mg, 0.068 mmol) was added and the reaction stirred overnight at room temperature. Pyridine was removed under reduced pressure and the crude mixture was purified via silica gel chromatography (eluent; 2-10% MeOH in ethyl acetate) to give the desire product as a white solid (16.7 mg, yield 90%). The spectral data were in accordance with those reported in the literature.[11] **42:** ^{1}H NMR (500 MHz, acetone-*d6*) δ 8.85 (s, 1H), 7.87 (t, *J* = 5.9 Hz, 1H), 7.47 (d, *J* = 8.0 Hz, 2H), 7.41 (d, *J* = 7.9 Hz, 2H), 7.28 (d, *J* = 8.9 Hz, 1H), 4.66-4.62 (m, 3H), 4.59 – 4.53 (m, 2H), 4.37 – 4.33 (m, 1H), 3.93 (d, *J* = 10.7 Hz, 1H), 3.76 (dd, *J* = 10.8, 3.9 Hz, 1H), 2.47 (s, 3H), 2.38 – 2.28 (m, 5H), 2.18– 2.16 (m, 2H), 1.91 – 1.84 (m, 2H), 1.00 (s, 9H).

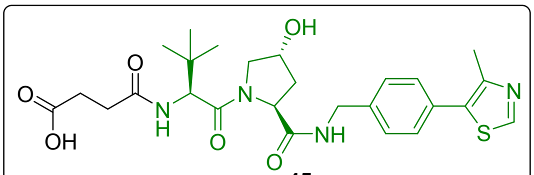

45

4-(((*S*)-1-((2*S*,4*R*)-4-hydroxy-2-((4-(4-methylthiazol-5-yl)benzyl)carbamoyl)pyrrolidin-1-yl)-3,3-dimethyl-1-oxobutan-2-yl)amino)-4-oxobutanoic acid, 45: In a flask containing (*S*)-1-((2*S*,4*R*)-4-hydroxy-2-((4-(4-methylthiazol-5-yl)benzyl)carbamoyl)pyrrolidin-1-yl)-3,3-dimethyl-1-oxobutan-2-aminium 2,2,2-trifluoroacetate **S14** (50 mg, 0.092 mmol) as a solution in dry pyridine (200 µL) and under inert atmosphere, succinic anhydride (18 mg, 0.184 mmol) was added and the reaction stirred overnight at room temperature. Pyridine was removed under reduced pressure and the crude mixture was purified via silica gel chromatography (eluent; 2-10% MeOH in ethyl acetate) to give the desire product as a white solid (32 mg, yield 66%). The spectral data were in accordance with those reported in the literature.[12] **45:** ^{1}H NMR (500 MHz, acetone-*d6*) δ 8.85 (s, 1H), 7.89 (t, *J* = 6.0 Hz, 1H), 7.47 (d, *J* = 8.2 Hz, 2H), 7.41 (d, *J* = 8.3 Hz, 2H), 7.37 (d, *J* = 9.0 Hz, 1H), 4.67 – 4.61 (m, 3H), 4.59 – 4.53 (m, 2H), 4.35 (dd, *J* = 15.5, 5.4 Hz, 1H), 3.89 (d, *J* = 10.3 Hz, 1H), 3.75 (dd, *J* = 10.8, 4.1 Hz, 1H), 2.65 – 2.49 (m, 5H), 2.47 (s, 3H), 2.17 – 2.15 (m, 2H), 1.00 (s, 9H).

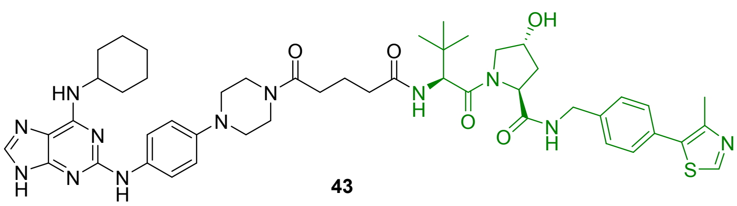

43

(2*S*,4*R*)-1-((S)-2-(5-(4-(4-((6-(cyclohexylamino)-9*H*-purin-2-yl)amino)phenyl)piperazin-1-yl)-5-oxopentanamido)-3,3-dimethylbutanoyl)-4-hydroxy-*N*-(4-(4-methylthiazol-5-yl)benzyl)pyrrolidine-2-carboxamide, 43: In a dried flask containing 5-(((*S*)-1-((2*S*,4*R*)-4-hydroxy-2-((4-(4-methylthiazol-5-yl) benzyl)carbamoyl)pyrrolidin-1-yl)-3,3-dimethyl-1-oxobutan-2-yl)amino)-5-oxopentanoic acid **42** (31 mg, 0.057 mmol) dry DMF (1.2 mL), azareversine (27 mg, 0.068 mmol), HATU (43mg, 0.114 mmol) and DIPEA (44mg, 0.34 mmol) were added under Ar atmosphere. The reaction mixture was stirred overnight at room temperature. After the reaction's completion the solvent was evaporated, and the crude mixture was purified by flash chromatography (eluent: 5% MeOH in dichloromethane) to afford the desired product as a white solid (40 mg, yield 76%). **43:** ^{1}H NMR (500 MHz, DMSO-*d6*) δ 9.49 (br, 1H), 8.97 (s, 1H), 8.85 (br, 1H), 8.56 (t, *J* = 6.0 Hz, 1H), 7.90 (d, *J* = 9.3 Hz, 2H), 7.63 (d, *J* = 8.9 Hz, 2H), 7.40 (d, *J* = 8.1 Hz, 2H), 7.36 (d, *J* = 8.2 Hz, 2H), 6.88 (d, *J* = 8.9 Hz, 2H), 5.14 (br, 1H), 4.55 (d, *J* = 9.3 Hz, 1H), 4.43 (m, 1H), 4.36 (br, 1H), 4.22 (dd, *J* = 15.8, 5.4 Hz, 1H), 3.67 (s, 2H), 3.58 (m, 4H), 3.08 (m, 2H), 3.01 (m, 4H), 2.44 (s, 3H), 2.33 (t, *J* = 7.6 Hz, 2H), 2.30 – 2.17 (m, 3H), 2.08 – 2.01 (m, 1H), 1.98 – 1.85 (m, 3H), 1.81 – 1.69 (m, 4H), 1.63 (d, *J* = 11.7 Hz, 1H), 1.42 – 1.29 (m, 5H), 0.95 (s, 9H); ^{13}C NMR (126 MHz, DMSO-*d6*) δ 174.6, 172.0, 171.9, 171.4, 170.4, 169.8, 152.0, 151.5, 147.7, 146.0, 139.5, 133.1, 131.2, 129.7, 128.9, 128.7, 128.1, 127.5, 120.7, 116.6, 72.5, 68.9, 58.8, 56.5, 56.5, 49.8, 49.5, 44.9, 42.8, 41.7, 41.0, 38.0, 35.2, 34.3, 31.8, 26.4, 25.2, 24.9, 21.4, 15.9. one carbon missing due to overlapping; ESI-HRMS m/z for $C_{48}H_{63}N_{12}O_5S$ $[M+H]^+$ calcd 919.4760, found 919.4786.

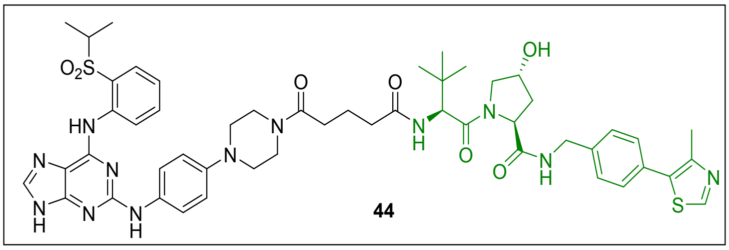

44

(2*S*,4*R*)-4-hydroxy-1-((S)-2-(5-(4-(4-((6-((2-(isopropylsulfonyl)phenyl)amino)-9*H*-purin-2-yl)amino)phenyl)piperazin-1-yl)-5-oxopentanamido)-3,3-dimethylbutanoyl)-*N*-(4-(4-methylthiazol-5-yl)benzyl)pyrrolidine-2-carboxamide, 44: In a dried flask containing 5-(((*S*)-1-((2*S*,4*R*)-4-hydroxy-2-((4-(4-methylthiazol-5-yl)benzyl)carbamoyl)pyrrolidin-1-yl)-3,3-dimethyl-1-oxobutan-2-yl)amino)-5-oxopentanoic acid **42** (30 mg, 0.055 mmol) dry DMF (1.2 mL), *N*6-(2-(isopropylsulfonyl)phenyl)-*N*2-(4-(piperazin-1-yl)phenyl)-9*H*-purine-2,6-diamine **SF-1** (30 mg, 0.061 mmol), HATU (25 mg, 0.066 mmol) and DIPEA (43 mg, 0.33 mmol) were added under Ar atmosphere. The reaction mixture was stirred overnight at room temperature. After the reaction's completion the solvent was evaporated and the crude mixture was purified by flash chromatography (eluent: 2% MeOH in dichloromethane) to afford the desired product as a yellow glacial solid (15 mg, yield 27%). **44:** ^{1}H NMR (500 MHz, DMSO-*d*6) δ 12.77 (br, 1H), 9.85 (s, 1H), 9.08 – 9.03 (m, 2H), 8.97 (s, 1H), 8.58 (m, 1H), 7.97 (s, 1H), 7.92 (d, *J* = 9.2 Hz, 1H), 7.81 (dd, *J* = 8.0, 1.2 Hz, 1H), 7.73 (m, 1H), 7.60 (d, *J* = 8.7 Hz, 2H), 7.42 (d, *J* = 8.1 Hz, 2H), 7.37 (d, *J* = 8.2 Hz, 2H), 7.30 – 7.27 (m, 1H), 6.92 (d, *J* = 8.9 Hz, 2H), 5.17 (s, 1H), 4.55 (d, *J* = 9.3 Hz, 1H), 4.43 (dd, *J* = 15.7, 7.2 Hz, 2H), 4.36 (br, 1H), 4.22 (dd, *J* = 15.9, 5.4 Hz, 1H), 3.68 (s, 2H), 3.59 (m, 2H), 3.56 (m, 2H), 3.04 (d, *J* = 24.0 Hz, 4H), 2.43 (s, 3H), 2.34 (t, *J* = 7.5 Hz, 2H), 2.24 (m, 3H), 2.05 (m, 1H), 1.95 – 1.87 (m, 1H), 1.79 – 1.71 (m, 2H), 1.18 (d, *J* = 6.8 Hz, 6H), 0.95 (s, 9H); ^{13}C NMR (126 MHz, DMSO-*d*6) δ 172.03, 171.97, 170.39, 169.77, 156.18, 152.30, 151.50, 150.55, 147.75, 145.78, 139.53, 139.29, 138.41, 135.17, 133.85, 131.22, 130.98, 129.67, 128.91, 128.68, 127.46, 122.28, 122.16, 120.51, 116.65, 115.03, 68.95, 58.77, 56.50, 56.45, 55.27, 49.90, 49.50, 44.94, 41.70, 41.02, 40.11, 40.02, 35.22, 34.30, 31.82, 26.45, 21.41, 15.99, 14.98; ESI-HRMS m/z for $C_{51}H_{63}N_{12}O_7S_2$ $[M+H]^+$ calcd 1019.4379, found 1019.4401.

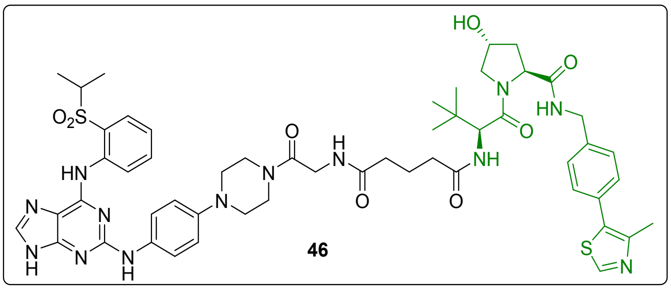

46

***N*1-((S)-1-((2*S*,4*R*)-4-hydroxy-2-((4-(4-methylthiazol-5-yl)benzyl)carbamoyl)pyrrolidin-1-yl)-3,3-dimethyl-1-oxobutan-2-yl)-*N*5-(2-(4-(4-((6-((2-(isopropylsulfonyl)phenyl)amino)-9*H*-purin-2-yl)amino)phenyl)piperazin-1-yl)-2-oxoethyl)glutaramide, 46:** In a dried flask containing 5-(((*S*)-1-((2*S*,4*R*)-4-hydroxy-2-((4-(4-methylthiazol-5-yl)benzyl)carbamoyl)pyrrolidin-1-yl)-3,3-dimethyl-1-oxobutan-2-yl)amino)-5-oxopentanoic acid **42** (11 mg, 0.021 mmol) dry DMF (0.5 mL), 2-(4-(4-((6-((2-(isopropylsulfonyl)phenyl)amino)-9*H*-purin-2-yl)amino) phenyl)piperazin-1-yl)-2-oxoethan-1-aminium 2,2,2-trifluoroacetate **SF-1** (15 mg, 0.023 mmol), HATU (9 mg, 0.025 mmol) and DIPEA (16 mg, 0.12 mmol) were added under Ar atmosphere. The reaction mixture was stirred overnight at room temperature. After the reaction's completion the solvent was evaporated and the crude mixture was purified by flash chromatography (eluent: 2% MeOH in dichloromethane) to afford the desired product as a brown solid (10 mg, yield 45%). **46:** ^{1}H NMR (500 MHz, DMSO-*d*6) δ 12.74 (br, 1H), 9.84 (s, 1H), 9.05 (d, *J* = 8.6 Hz, 1H), 9.02 (s, 1H), 8.97 (s, 1H), 8.57 – 8.55 (m, 1H), 8.00 – 7.96 (m, 3H), 7.94 (d, *J* = 9.2 Hz, 1H), 7.82 (dd, *J* = 7.9, 1.4 Hz, 1H),

7.76 – 7.71 (m, 1H), 7.61 (d, *J* = 8.8 Hz, 2H), 7.42 (d, *J* = 8.3 Hz, 2H), 7.38 (d, *J* = 8.3 Hz, 2H), 7.30 – 7.27 (m, 1H), 6.92 (d, *J* = 9.0 Hz, 2H), 5.13 (br, 1H), 4.52 (d, *J* = 9.2 Hz, 1H), 4.48 – 4.39 (m, 2H), 4.36 (br, 1H), 4.22 (dd, *J* = 15.9, 5.4 Hz, 1H), 4.10 – 4.03 (m, 1H), 3.93 (dd, *J* = 16.6, 5.3 Hz, 1H), 3.68 (d, *J* = 2.5 Hz, 2H), 3.60 (m, 2H), 3.56 (m, 2H) 3.46 – 3.40 (m, 1H), 3.08 – 3.03 (m, 4H), 2.44 (s, 3H), 2.24 – 2.21 (m, 2H), 2.14 – 2.11 (m, 2H), 2.06 – 2.01 (m, 1H), 1.91 (ddd, *J* = 12.9, 8.6, 4.6 Hz, 1H), 1.77 – 1.70 (m, 2H), 1.19 (d, *J* = 6.8 Hz, 6H), 0.94 (s, 9H); ^{13}C NMR (126 MHz, DMSO-*d*6) δ 172.1, 171.9, 171.9, 170.0, 167.3, 156.1, 151.4, 150.5, 147.7, 145.6, 139.5, 139.2, 138.3, 135.1, 133.9, 131.1, 130.9, 129.6, 128.6, 127.4, 122.3, 122.3, 122.1, 122.1, 120.5, 116.6, 1145.0, 68.9, 58.7, 56.6, 56.4, 55.2, 49.7, 49.4, 44.1, 41.6, 41.4, 40.4, 37.9, 35.0, 34.5, 34.1, 26.4, 22.0, 15.9, 14.9; ESI-HRMS m/z for $C_{53}H_{66}N_{13}O_8S_2$ $[M+H]^+$ calcd 1076.4599, found 1076.4626.

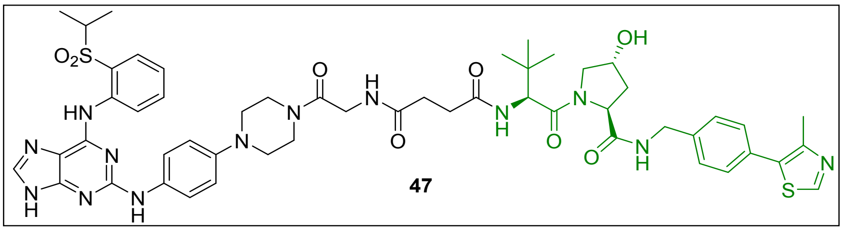

***N*1-((S)-1-((2*S*,4*R*)-4-hydroxy-2-((4-(4-methylthiazol-5-yl)benzyl)carbamoyl)pyrrolidin-1-yl)-3,3-dimethyl-1-oxobutan-2-yl)-*N*4-(2-(4-(4-((6-((2-(isopropylsulfonyl)phenyl)amino)-9*H*-purin-2-yl)amino)phenyl)piperazin-1-yl)-2-oxoethyl)succinamide, 47:** In a dried flask containing 4-(((*S*)-1-((2*S*,4*R*)-4-hydroxy-2-((4-(4-methylthiazol-5-yl)benzyl)carbamoyl)pyrrolidin-1-yl)-3,3-dimethyl-1-oxobutan-2-yl)amino)-4-oxobutanoic acid **46** (13 mg, 0.023 mmol) dry DMF (0.5 mL), 2-(4-(4-((6-((2-(isopropylsulfonyl)phenyl)amino)-9*H*-purin-2-yl)amino)phenyl)piperazin-1-yl)-2-oxoethan-1-aminium 2,2,2-trifluoroacetate **SF-1 (**17 mg, 0.026 mmol), HATU (11 mg, 0.028 mmol) and DIPEA (18 mg, 0.14 mmol) were added under Ar atmosphere. The reaction mixture was stirred overnight at room temperature. After the reaction's completion the solvent was evaporated and the crude mixture was purified via preparative HPLC to afford the desired product as a white solid (8 mg, yield 32%). **47:** ^{1}H NMR (500 MHz, DMSO-*d*6) δ 12.76 (s, 1H), 9.85 (s, 1H), 9.05 (m, 2H), 8.98 (s, 1H), 8.57 (m, 1H), 8.03 – 7.91 (m, 3H), 7.82 (d, *J* = 6.7 Hz, 1H), 7.73 (m, 1H), 7.61 (d, *J* = 8.6 Hz, 2H), 7.42 (d, *J* = 8.1 Hz, 2H), 7.38 (d, *J* = 8.1 Hz, 2H), 7.30 – 7.27 (m, 1H), 6.93 (d, *J* = 9.0 Hz, 2H), 5.14 (s, 1H), 4.53 (d, J = 9.3 Hz, 1H), 4.49 – 4.37 (m, 2H), 4.34 (s, 1H), 4.22 (dd, J = 15.9, 5.5 Hz, 1H), 3.99 (d, *J* = 5.3 Hz, 2H), 3.70 – 3.54 (m, 6H), 3.43 (m, 2H), 3.12 – 3.00 (m, 4H), 2.44 (s, 3H), 2.43 – 2.35 (m, 4H), 2.07 – 2.00 (m, 1H), 1.90 (m, 1H), 1.18 (d, J = 6.8 Hz, 6H), 0.93 (s, 9H); ^{13}C NMR (126 MHz, DMSO-*d*6) δ 172.4, 172.1, 171.7, 170.0, 167.5, 156.6, 151.9, 151.0, 148.2, 146.1, 140.0, 139.7, 138.8, 135.6, 134.3, 131.6, 131.4, 130.1, 129.1, 127.9, 122.7, 122.6, 120.9, 117.1, 115.4, 110.0, 69.3, 59.2, 56.9, 56.8, 55.6, 50.2, 49.8, 44.5, 42.1, 41.8, 40.9, 38.4, 35.8, 31.2, 30.9, 26.8, 16.4, 15.4; ESI-HRMS m/z for $C_{52}H_{64}N_{13}O_8S_2$ $[M+H]^+$ calcd 1062.4442, found 1062.4470.

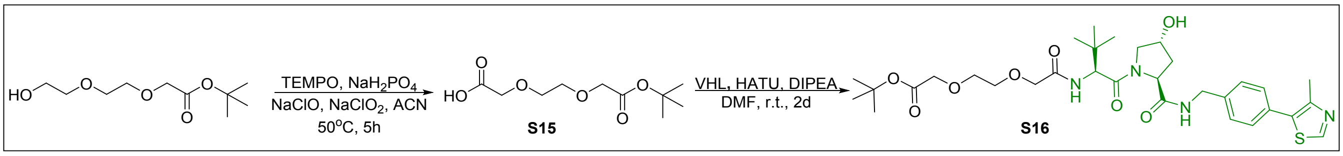

Scheme S9

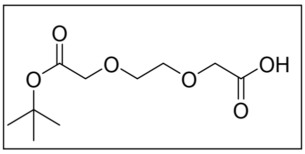

2-(2-(2-(*tert*-butoxy)-2-oxoethoxy)ethoxy)acetic acid, S15: In a flask containing *tert*-butyl 2-(2-(2-hydroxyethoxy)ethoxy)acetate (1.2 g, 5.45 mmol), acetonitrile (30 mL) was added followed by the addition of aqueous solutions of NaH_2PO_4 (25 mL) and $NaClO_2$ (3.9 g in 50 mL H_2O), TEMPO (370 mg, 2.39 mmol) and NaClO (2 mL) in room temperature and the reaction was allowed to stir at 50 °C for 5h. Then the reaction was quenched by the addition of saturated aqueous solution of Na_2SO_3 and acetonitrile was evaporated. Afterwards the crude was acidified to pH=1 with aq. solution of HCl 2M and extracted with ethyl acetate. The organic layer was dried over anhydrous Na_2SO_4, filtered, and concentrated under reduced pressure to afford the desired acid as a colorless oil (1.04 g, 82% yield). The spectral data were in accordance with those reported in the literature [13]**S15:** ^{1}H NMR (500 MHz, $CDCl_3$) δ 4.19 (s, 2H), 4.03 (s, 2H), 3.80 – 3.77 (m, 2H), 3.76 – 3.73 (m, 2H), 1.47 (s, 9H).

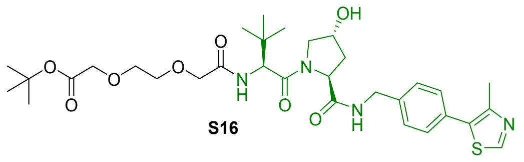

S16

***tert*-butyl 2-(2-(2-(((S)-1-((2S,4R)-4-hydroxy-2-((4-(4-methylthiazol-5-yl)benzyl)carbamoyl)pyrrolidin-1-yl)-3,3-dimethyl-1-oxobutan-2-yl)amino)-2-oxoethoxy)ethoxy)acetate, S16:** In a flask *tert*-butyl (*(S*)-1-((2*S*,4*R*)-4-hydroxy-2-((4-(4-methylthiazol-5-yl)benzyl)carbamoyl)pyrrolidin-1-yl)-3,3-dimethyl-1-oxobutan-2-yl)carbamate (127 mg, 0.235 mmol) was dissolved in a mixture of dichloromethane/TFA 3/1 (1 mL) and the solution was stirred for 1h at ambient temperature. Afterwards the solvents were removed in rotary to afford the desired salt which was used in the next step without further purification. To a stirred solution of 2-(((*S*)-1-((2*S*,4*R*)-4-hydroxy-2-((4-(4-methylthiazol-5-yl)benzyl) carbamoyl)pyrrolidin-1-yl)-3,3-dimethyl-1-oxobutan-2-yl)amino)-2-oxoethan-1-aminium2,2,2-trifluoroacetate (128 mg, 0.235 mmol) as a solution in dry DMF (0.7 mL), DIPEA (186 μL, 1.07 mmol) and HATU (122 mg, 0.32 mmol) were added followed by the addition of 2-(2-(2-(tert-butoxy)-2-oxoethoxy)ethoxy)acetic acid (50 mg, 0.213 mmol) and the reaction stirred at room temperature overnight. The reaction was diluted in ethyl acetate, washed with 5% aq. solution of citric acid and saturated $NaHCO_3$ and the organic layer was dried over anhydrous Na_2SO_4, filtered, and concentrated under reduced pressure. The crude product was purified by silica gel chromatography (5% MeOH in EtOAc to 15% MeOH in EtOAc) to afford the desired product as a yellow-white solid (45 mg, 43% yield). **S16:** ^{1}H NMR (500 MHz, $CDCl_3$) δ 8.67 (s, 1H), 7.56 – 7.51 (m, 1H), 7.42 (d, *J* = 7.8 Hz, 1H), 7.35 (d, *J* = 8.4 Hz, 2H), 7.32 (d, *J* = 8.4 Hz, 2H), 4.73 (t, *J* = 8.0 Hz, 1H), 4.54 – 4.50 (m, 2H), 4.46 (d, *J* = 8.3 Hz, 1H), 4.34 (dd, *J* = 15.0, 5.4 Hz, 1H), 4.12 – 4.07 (m, 1H), 4.05 – 3.97 (m, 3H), 3.73 – 3.67 (m, 4H), 3.59 (dd, *J* = 11.3, 3.3 Hz, 1H), 2.51 (s, 3H), 2.49 – 2.46 (m, 1H), 2.13 (dd, *J* = 13.5, 8.1 Hz, 1H), 1.45 (s, 9H), 0.95 (s, 9H); ^{13}C NMR (126 MHz, $CDCl_3$) δ 171.6, 171.0, 170.9, 169.9, 150.4, 148.6, 138.3, 131.8, 131.0, 129.6, 128.2, 82.3, 71.0, 70.4, 70.3, 70.2, 68.9, 58.6, 57.7, 56.9, 43.3, 36.1, 34.9, 28.2, 26.5, 16.2; ESI-MS m/z for $C_{32}H_{47}N_4O_8S$ $[M+H]^+$ calculated 647.31; found 646.95.

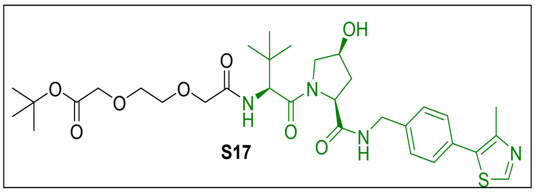

S17

***tert*-butyl 2-(2-(2-(((*S*)-1-((2*S*,4*S*)-4-hydroxy-2-((4-(4-methylthiazol-5-yl)benzyl)carbamoyl)pyrrolidin-1-yl)-3,3-dimethyl-1-oxobutan-2-yl)amino)-2-oxoethoxy)ethoxy)acetate, S17:** In a flask *tert*-butyl ((*S*)-1-((2*S*,4*R*)-4-hydroxy-2-((4-(4-methylthiazol-5-yl)benzyl)carbamoyl)pyrrolidin-1-yl)-3,3-dimethyl-1-oxobutan-2-yl)carbamate (127 mg, 0.235 mmol) was dissolved in a mixture of dichloromethane/TFA 3/1 (1 mL) and the solution was stirred for 1h at ambient temperature. Afterwards the solvents were removed in rotary to afford the desired salt which was used in the next step without further purification. To a stirred solution of 2-(((*S*)-1-

((2*S*,4*R*)-4-hydroxy-2-((4-(4-methylthiazol-5-yl)benzyl) carbamoyl)pyrrolidin-1-yl)-3,3-dimethyl-1-oxobutan-2-yl)amino)-2-oxoethan-1-aminium2,2,2-trifluoroacetate (128 mg, 0.235 mmol) as a solution in dry DMF (0.7 mL), DIPEA (186 µL, 1.07 mmol) and HATU (122 mg, 0.32 mmol) were added followed by the addition of 2-(2-(2-(tert-butoxy)-2-oxoethoxy)ethoxy)acetic acid (50 mg, 0.213 mmol) and the reaction stirred at room temperature overnight. The reaction was diluted in ethyl acetate, washed with 5% aq. solution of citric acid and saturated $NaHCO_3$ and the organic layer was concentrated under reduced pressure. The crude product was purified by silica gel chromatography (5% MeOH in EtOAc to 15% MeOH in EtOAc) to afford the desired product as a yellow-white solid (45 mg, yield= 43%). **S17:** ^{1}H NMR (300 MHz, $CDCl_3$) δ 8.69 (s, 1H), 7.67 – 7.59 (m, 1H), 7.41 – 7.26 (m, 6H), 5.58 (d, *J* = 9.8 Hz, 1H), 4.75 (d, *J* = 8.8 Hz, 1H), 4.64 (dd, J = 14.8, 6.9 Hz, 1H), 4.53 – 4.46 (m, 3H), 4.31 (dd, *J* = 14.9, 5.0 Hz, 1H), 4.05 – 3.99 (m, 3H), 3.96 – 3.91 (m, 2H), 3.81 (d, *J* = 10.9 Hz, 1H), 3.73 (m, 4H), 2.52 (s, 3H), 2.36 (d, *J* = 14.2 Hz, 1H), 2.25 – 2.12 (m, 1H), 1.46 (s, 9H), 0.94 (s, 9H); ^{13}C NMR (126 MHz, $CDCl_3$) δ 172.7, 172.0, 170.0, 169.5, 150.5, 148.6, 137.5, 131.6, 131.3, 129.7, 128.3, 81.9, 71.3, 71.2, 70.6, 70.5, 69.0, 60.0, 58.8, 56.7, 43.6, 35.1, 28.2, 26.4, 16.2; ESI-HRMS m/z for $C_{32}H_{47}N_4O_8S$ [M+H]+ calcd 647.3115, found 647.3098.

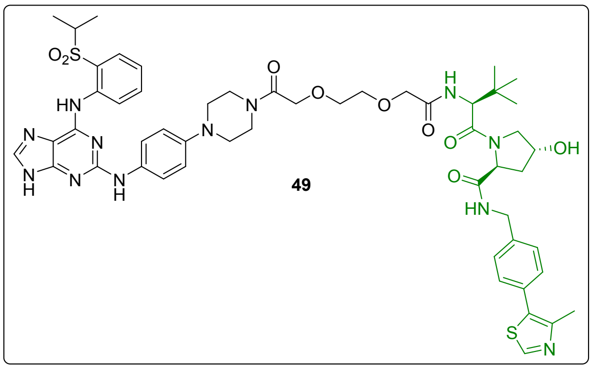

(2*S*,4*R*)-4-hydroxy-1-((S)-2-(2-(2-(2-(4-(4-((6-((2-(isopropylsulfonyl)phenyl)amino)-9*H*-purin-2-yl)amino)phenyl)piperazin-1-yl)-2-oxoethoxy)ethoxy)acetamido)-3,3-dimethylbutanoyl)-*N*-(4-(4-methylthiazol-5-yl)benzyl)pyrrolidine-2-carboxamide, 49: In a flask *tert*-butyl 2-(2-(2-(((*S*)-1-((2*S*,4*R*)-4-hydroxy-2-((4-(4-methylthiazol-5-yl)benzyl)carbamoyl)pyrrolidin-1-yl)-3,3-dimethyl-1-oxobutan-2-yl)amino)-2-oxoethoxy)ethoxy)acetate **S16** (33 mg, 0.057 mmol) was dissolved in a mixture of dichloromethane/TFA 3/1 (0.35 mL) and the solution was stirred for 2h at ambient temperature. Afterwards the solvents were removed in rotary to afford the desired salt which was used in the next step without further purification. In a dried flask containing 2-(2-(2-(((*S*)-1-((2*S*,4*R*)-4-hydroxy-2-((4-(4-methylthiazol-5-yl)benzyl)carbamoyl)pyrrolidin-1-yl)-3,3-dimethyl-1-oxobutan-2-yl)amino)-2-oxoethoxy)ethoxy)acetic acid **48** (34 mg, 0.057 mmol) dry DMF (1.3 mL), N6-(2-(isopropylsulfonyl)phenyl)-*N*2-(4-(piperazin-1-yl)phenyl)-9*H*-purine-2,6-diamine (31 mg, 0.063 mmol), HOBt (12 mg, 0.086 mmol), EDCI (16 mg, 0.086 mmol) and triethylamine (17 mg, 0.17 mmol) were added under Ar atmosphere. The reaction mixture was stirred overnight at room temperature. After the reaction's completion the solvent was evaporated and the crude mixture was purified by flash chromatography (eluent: 3% MeOH in dichloromethane) to afford the desired product as an orange solid (15 mg, yield 25%). **49:** ^{1}H NMR (500 MHz, DMSO-*d*6) δ 12.75 (s, 1H), 9.85 (s, 1H), 9.06 (d, *J* = 8.1 Hz, 1H), 9.03 (s, 1H), 8.96 (s, 1H), 8.60 – 8.58 (m, 1H), 7.97 (s, 1H), 7.81 (dd, *J* = 8.0, 1.3 Hz, 1H), 7.74 – 7.71 (m, 1H), 7.60 (d, *J* = 8.8 Hz, 2H), 7.48 (d, *J* = 9.6 Hz, 1H), 7.43 – 7.36 (m, 4H), 7.30 – 7.27 (m, 1H), 6.91 (d, *J* = 8.9 Hz, 2H), 5.17 (br, 1H), 4.58 (d, *J* = 9.6 Hz, 1H), 4.45 (m, 1H), 4.36 (m, 2H), 4.29 – 4.22 (m, 4H), 4.00 (s, 2H), 3.68 – 3.62 (m, 4H), 3.59 – 3.53 (m, 4H), 3.43 (m, 1H), 3.05 – 3.02 (m, 4H), 2.42 (s, 3H), 2.19 – 2.16 (m, 1H), 2.10 – 2.04 (m, 1H), 2.01 – 1.95 (m, 1H), 1.93 – 1.87 (m, 1H), 1.18 (d, *J* = 6.8 Hz, 6H), 0.95 (s, 9H); ^{13}C NMR (126 MHz, DMSO-*d*6) δ 171.8, 171.3, 169.2, 168.6, 167.2, 156.1, 152.3, 151.4, 150.5, 147.7, 145.7, 139.4, 139.3, 138.4, 135.1, 131.1, 130.9, 129.7, 128.7, 127.4, 122.3, 122.1, 120.5, 116.6, 115.0, 109.5, 70.3, 69.6, 69.6, 69.4, 68.9, 58.8, 56.6,

55.7, 55.2, 49.8, 49.4, 44.2, 42.7, 41.7, 41.1, 37.9, 35.8, 26.2, 15.9, 14.9; ESI-HRMS m/z for $C_{52}H_{65}N_{12}O_9S_2$ $[M+H]^+$ calcd 1065.4433, found 1065.4473.

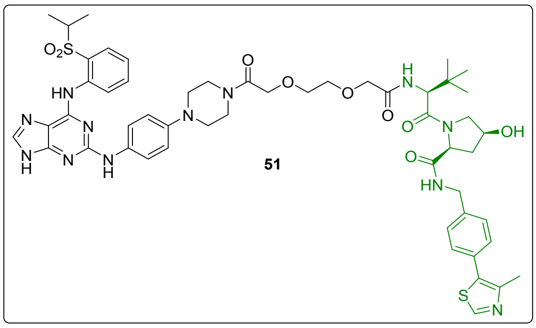

(2*S*,4*S*)-4-hydroxy-1-((*S*)-2-(2-(2-(2-(4-(4-((6-((2-(isopropylsulfonyl)phenyl)amino)-9*H*-purin-2-yl)amino)phenyl)piperazin-1-yl)-2-oxoethoxy)ethoxy)acetamido)-3,3-dimethylbutanoyl)-*N*-(4-(4-methylthiazol-5-yl)benzyl)pyrrolidine-2-carboxamide, 51: In a flask *tert*-butyl 2-(2-(2-(((*S*)-1-((2*S*,4*S*)-4-hydroxy-2-((4-(4-methylthiazol-5-yl)benzyl)carbamoyl)pyrrolidin-1-yl)-3,3-dimethyl-1-oxobutan-2-yl)amino)-2-oxoethoxy)ethoxy)acetate **S17** (24 mg, 0.042 mmol) was dissolved in a mixture of dichloromethane/TFA 3/1 (0.3 mL) and the solution was stirred for 2h at ambient temperature. Afterwards the solvents were removed in rotary to afford the desired salt which was used in the next step without further purification. In a dried flask containing 2-(2-(2-(((*S*)-1-((2*S*,4*S*)-4-hydroxy-2-((4-(4-methylthiazol-5-yl)benzyl)carbamoyl)pyrrolidin-1-yl)-3,3-dimethyl-1-oxobutan-2-yl)amino)-2-oxoethoxy)ethoxy)acetic acid **50** (25 mg, 0.042 mmol) dry DMF (1.0 mL), *N6*-(2-(isopropylsulfonyl)phenyl)-*N*2-(4-(piperazin-1-yl)phenyl)-9*H*-purine-2,6-diamine (25 mg, 0.05 mmol), HOBt (11 mg, 0.085 mmol), EDCI (16 mg, 0.085 mmol) and triethylamine (13 mg, 0.13 mmol) were added under Ar atmosphere. The reaction mixture was stirred overnight at room temperature. After the reaction's completion the solvent was evaporated and the crude mixture was purified by flash chromatography (eluent: 3% MeOH in dichloromethane) to afford the desired product as an orange solid (10 mg, yield 22%). **51:** ^{1}H NMR (500 MHz, acetone- *d6*) δ 11.87 (br, 1H), 9.98 (s, 1H), 9.08 (d, *J* = 8.2 Hz, 1H), 8.83 (s, 1H), 8.36 (s, 1H), 8.25 – 8.19 (m, 1H), 7.91 (s, 1H), 7.87 (d, *J* = 7.9 Hz, 1H), 7.66 (m, 3H), 7.44 (d, *J* = 8.1 Hz, 2H), 7.39 (d, *J* = 8.1 Hz, 2H), 7.26 (t, *J* = 7.5 Hz, 1H), 6.94 (d, *J* = 8.8 Hz, 2H), 5.56 (d, *J* = 7.2 Hz, 1H), 4.70 – 4.58 (m, 3H), 4.37 – 4.29 (m, 4H), 4.03 (m, 3H), 3.84 (m, 2H), 3.73 (m, 7H), 3.42 (dt, *J* = 13.5, 6.8 Hz, 1H), 3.17 – 3.03 (m, 5H), 2.45 (s, 3H), 2.33 (m, 1H), 2.00 (d, *J* = 13.8 Hz, 1H), 1.27 (d, *J* = 6.8 Hz, 6H), 1.02 (s, 9H); ^{13}C NMR (126 MHz, acetone-*d6*) δ 174.4, 171.5, 170.0, 168.2, 157.6, 153.4, 152.1, 151.4, 149.1, 147.3, 140.6, 139.7, 138.6, 135.6, 135.2, 132.2, 131.9, 131.4, 129.9, 128.6, 124.2, 123.5, 122.8, 121.5, 118.0, 110.9, 71.6, 71.6, 71.2, 70.9, 70.8, 60.7, 58.5, 57.0, 56.3, 51.2, 51.0, 45.7, 43.4, 42.4, 37.2, 36.3, 26.8, 16.4, 15.6; ESI-HRMS m/z for $C_{52}H_{65}N_{12}O_9S_2$ $[M+H]^+$ calcd 1065.4439, found 1065.4421.

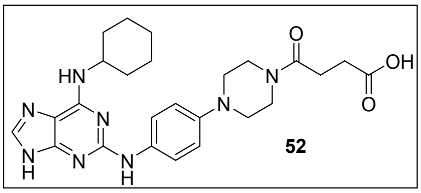

4-(4-(4-((6-(cyclohexylamino)-9H-purin-2-yl)amino)phenyl)piperazin-1-yl)-4-oxobutanoic acid, 52: In a flask containing azareversine (50 mg, 0.127 mmol) as a solution in dry pyridine (300 µL) and under Ar atmosphere, succinic anhydride (26 mg, 0.259 mmol) was added and the reaction stirred overnight at room temperature. Pyridine was removed under reduced pressure and the crude mixture was purified via silica gel chromatography (eluent; 2-10% MeOH in ethyl acetate) to give the desired product as a white solid (35 mg,

yield 56%). **52:** ^{1}H NMR (500 MHz, DMSO-*d6*) δ 12.30 (s, 1H), 12.04 (s, 1H), 8.54 (s, 1H), 7.73 (s, 1H), 7.67 (d, *J* = 8.9 Hz, 2H), 7.07 (s, 1H), 6.86 (d, *J* = 8.9 Hz, 2H), 3.59 (br, 2H), 3.39 (br, 2H), 3.02 (t, *J* = 5.0 Hz, 2H), 2.95 (t, *J* = 5.0 Hz, 2H) 2.60 – 2.56 (m, 2H), 2.43 (m, 3H), 1.92 (d, *J* = 9.1 Hz, 2H), 1.77 (d, *J* = 10.9 Hz, 2H), 1.64 (d, *J* = 12.4 Hz, 1H), 1.47 – 1.27 (m, 5H); ^{13}C NMR (126 MHz, DMSO-*d6*) δ 174.1, 173.7, 169.6, 158.5, 158.2, 158.0, 145.2, 119.6, 118.5, 116.8, 116.1, 50.0, 49.7, 44.7, 41.2, 30.6, 29.0, 27.5, 25.2, 19.2 ;ESI-MS m/z for $C_{25}H_{33}N_8O_3$ [M+H]$^+$ calcd 493.26, found 493.15.

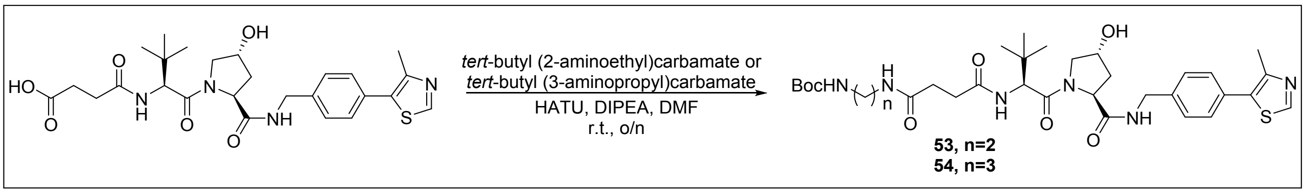

Scheme 10

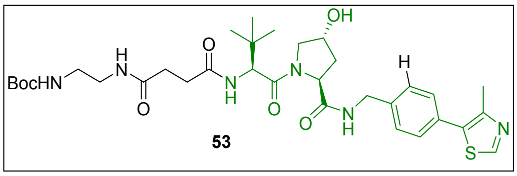

***tert*-butyl (2-(4-(((*S*)-1-((2*S*,4*R*)-4-hydroxy-2-((4-(4-methylthiazol-5-yl)benzyl)carbamoyl)pyrrolidin-1-yl)-3,3-dimethyl-1-oxobutan-2-yl)amino)-4-oxobutanamido)ethyl)carbamate, 53:** In a dried flask 4-(((S)-1-((2S,4R)-4-hydroxy-2-((4-(4-methylthiazol-5-yl)benzyl)carbamoyl)pyrrolidin-1-yl)-3,3-dimethyl-1-oxobutan-2-yl)amino)-4-oxobutanoic acid (32 mg, 0.06 mmol) was dissolved in dry DMF (1.3 mL) and *tert*-butyl (2-aminoethyl)carbamate (11 mg, 0.066 mmol), HATU (27 mg, 0.072 mmol) and DIPEA (46 mg, 0.362 mmol) were added under Ar atmosphere and the reaction mixture was stirred at room temperature for 16h. The solvent was removed under reduced pressure and the crude product was purified by flash chromatography (eluent; 5% MeOH in ethyl acetate) to afford the desired product as a colorless oil (29 mg, 71%). **53:** ^{1}H NMR (500 MHz, acetone-*d6*) δ 8.85 (s, 1H), 7.90 (br, 1H), 7.47 – 7.46 (m, 3H), 7.41 (d, *J* = 7.9 Hz, 2H), 7.34 (br, 1H), 6.16 (br, 1H), 4.66 – 4.62 (m, 2H), 4.57 (dd, *J* = 15.6, 6.7 Hz, 1H), 4.52 (br, 1H), 4.42 (br, 1H), 4.36 (dd, *J* = 15.4, 5.3 Hz, 1H), 3.90 (d, *J* = 10.5 Hz, 1H), 3.74 (dd, *J* = 10.7, 3.6 Hz, 1H), 3.28 – 3.22 (m, 2H), 3.18 – 3.14 (m, 2H), 2.58 – 2.50 (m, 2H), 2.47 (s, 3H), 2.45 – 2.41 (m, 2H), 2.16 (dd, *J* = 8.0, 3.3 Hz, 2H), 1.39 (s, 9H), 1.00 (s, 9H); ^{13}C NMR (126 MHz, $CDCl_3$) δ 173.4, 173.0, 171.8, 171.3, 156.7, 150.2, 148.3, 138.3, 131.6, 130.7, 129.3, 127.9, 79.7, 70.1, 58.8, 58.5, 57.0, 43.0, 40.0, 39.7, 34.9, 31.2, 30.9, 28.3, 26.5, 16.0; ESI-MS m/z for $C_{33}H_{47}N_6O_7S$ [M-H]$^-$ calcd 671.32, found 671.05.

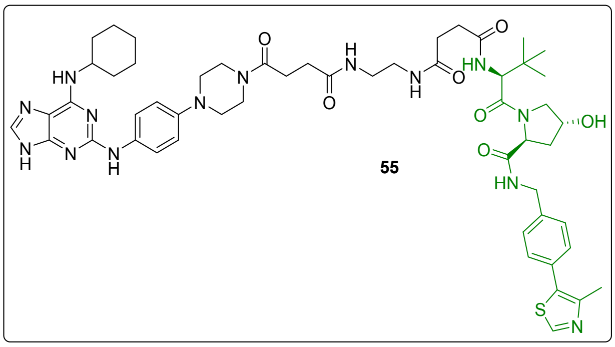

***N*1-(2-(4-(4-(4-((6-(cyclohexylamino)-9*H*-purin-2-yl)amino)phenyl)piperazin-1-yl)-4-oxobutanamido)ethyl)-*N*4-((*R*)-1-((2*S*,4*R*)-4-hydroxy-2-((4-(4-methylthiazol-5-yl)benzyl)carbamoyl)pyrrolidin-1-yl)-3,3-dimethyl-1-**

oxobutan-2-yl)succinimide, 55: In a flask *tert*-butyl (2-(4-(((*S*)-1-((2*S*,4*R*)-4-hydroxy-2-((4-(4-methylthiazol-5-yl)benzyl)carbamoyl)pyrrolidin-1-yl)-3,3-dimethyl-1-oxobutan-2-yl)amino)-4-oxobutanamido)ethyl)carbamate **53** (30 mg, 0.044 mmol) was dissolved in a mixture of dichloromethane/TFA 3/1 (0.35 mL) and the solution was stirred for 2h at ambient temperature. Afterwards the solvents were removed in rotary to afford the desired salt which was used in the next step without further purification. In a dried flask containing 4-(4-(4-((6-(cyclohexylamino)-9*H*-purin-2-yl)amino)phenyl)piperazin-1-yl)-4-oxobutanoic acid (20 mg, 0.04 mmol) dry DMF (0.9 mL), 2-(4-(((*R*)-1-((2*S*,4*R*)-4-hydroxy-2-((4-(4-methylthiazol-5-yl)benzyl)carbamoyl)pyrrolidin-1-yl)-3,3-dimethyl-1-oxobutan-2-yl)amino)-4-oxobutanamido)ethan-1-aminium 2,2,2-trifluoroacetate (30 mg, 0.044 mmol), HATU (18 mg, 0.048 mmol) and DIPEA (31 mg, 0.24 mmol) were added under Ar atmosphere. The reaction mixture was stirred overnight at room temperature. After the reaction's completion the solvent was evaporated and the crude mixture was purified via preparative HPLC to afford the desired product as a white solid (10 mg, yield 24%). **55:** ^{1}H NMR (500 MHz, DMSO-*d6*) δ 12.30 (br, 1H), 8.98 (s, 1H), 8.55 (t, *J* = 6.0 Hz, 1H), 8.3 (br, 1H), 7.91 (d, *J* = 9.3 Hz, 1H), 7.82 (br, 1H), 7.79 (br, 1H) 7.74 (s, 1H), 7.67 (d, *J* = 8.8 Hz, 2H), 7.40 (d, *J* = 8.3 Hz, 2H), 7.36 (d, *J* = 8.3 Hz, 2H), 7.04 (br, 1H), 6.85 (d, *J* = 8.9 Hz, 2H), 5.12 (br, 1H), 4.52 (d, *J* = 9.3 Hz, 1H), 4.42 (dd, *J* = 15.2, 7.0 Hz, 2H), 4.34 (br, 1H), 4.22 (dd, *J* = 15.9, 5.4 Hz, 1H), 3.66 (m, 2H), 3.58 (br, 2H), 3.35 (2H, overlapping with DMSO-*d6*), 3.05 (m, 4H), 3.01 (br, 2H), 3.03 (br, 2H), 2.58 (m, 4H), 2.44 (s, 3H), 2.38 (m, 4H), 2.06 – 2.00 (m, 2H), 1.96 – 1.86 (m, 3H), 1.80 – 1.73 (m, 1H), 1.64 (d, *J* = 12.5 Hz, 1H), 1.43 – 1.29 (m, 3H), 1.16 (m, 2H), 0.93 (s, 9H); ^{13}C NMR (126 MHz, DMSO-*d6*) δ 171.9, 171.6, 171.6, 171.3, 169.9, 169.6, 156.5, 151.47, 147.7, 144.9, 139.5, 135.9, 135.1, 131.2, 129.6, 128.6, 127.4, 119.2, 116.7, 109.6, 68.9, 58.7, 56.4, 56.3, 50.1, 49.7, 44.8, 41.7, 41.2, 38.4, 38.3, 37.9, 35.3, 31.0, 30.5, 27.9, 26.4, 25.4, 25.2, 15.9; ESI-HRMS m/z for $C_{53}H_{71}N_{14}O_7S$ $[M+H]^+$ calcd 1047.5345, found 1047.5365.

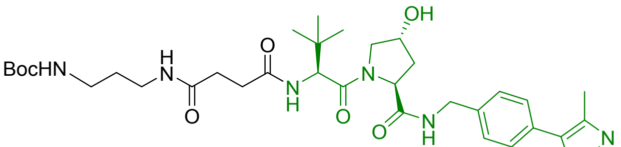

54

***tert*-butyl (3-(4-(((*S*)-1-((2*S*,4*R*)-4-hydroxy-2-((4-(4-methylthiazol-5-yl)benzyl)carbamoyl)pyrrolidin-1-yl)-3,3-dimethyl-1-oxobutan-2-yl)amino)-4-oxobutanamido)propyl)carbamate, 54:** In a dried flask 4-(((*S*)-1-((2*S*,4*R*)-4-hydroxy-2-((4-(4-methylthiazol-5-yl)benzyl)carbamoyl)pyrrolidin-1-yl)-3,3-dimethyl-1-oxobutan-2-yl)amino)-4-oxobutanoic acid (24 mg, 0.045 mmol) was dissolved in dry DMF (0.9 mL) and *tert*-butyl (3-aminopropyl)carbamate (9 mg, 0.049 mmol), HATU (21 mg, 0.054 mmol) and DIPEA (35 mg, 0.27 mmol) were added under Ar atmosphere and the reaction mixture was stirred at room temperature for 16h. The solvent was removed under reduced pressure and the crude product was purified by flash chromatography (eluent; 5% MeOH in ethyl acetate) to afford the desired product as a colorless oil (18 mg, 55%). **54:** ^{1}H NMR (500 MHz, acetone-*d6*) δ 8.84 (s, 1H), 7.94 (s, 1H), 7.47 (d, *J* = 8.1 Hz, 2H), 7.44 (s, 1H), 7.41 (d, *J* = 8.0 Hz, 2H), 7.29 (s, 1H), 6.08 (s, 1H), 4.68 – 4.62 (m, 2H), 4.56 (dd, *J* = 15.5, 6.6 Hz, 1H), 4.52 (br, 1H), 4.36 (dd, *J* = 15.5, 5.3 Hz, 1H), 3.88 (d, *J* = 10.7 Hz, 1H), 3.73 (dd, *J* = 10.8, 3.7 Hz, 1H), 3.25 – 3.17 (m, 2H), 3.08 (m, 2H), 2.87 (br, 1H), 2.63 – 2.55 (m, 1H), 2.53 – 2.42 (m, 2H), 2.47 (s, 3H), 2.30 (m, 1H), 2.17 – 2.14 (m, 2H), 1.60 (dt, *J* = 12.5, 6.1 Hz, 2H), 1.39 (s, 9H), 1.00 (s, 9H); ^{13}C NMR (126 MHz, $CDCl_3$) δ 173.1, 172.6, 171.6, 151.2, 149.1, 140.5, 132.3, 131.3, 130.3, 129.9, 128.7, 79.2, 70.7, 60.0, 58.0, 57.4, 43.1, 38.5, 38.3, 37.1, 36.2, 32.1, 31.8, 30.9, 28.7, 27.0, 16.3; ESI-HRMS m/z for $C_{34}H_{51}N_6O_7S$ $[M+H]^+$ calcd 687.3540, found 687.3531.

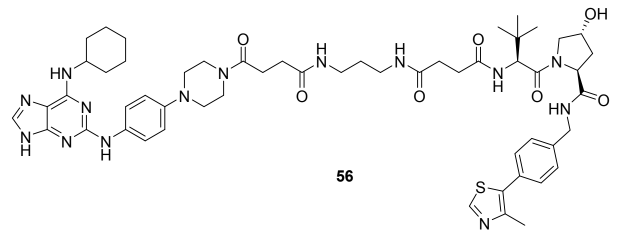

56

***N*1-(3-(4-(4-(4-((6-(cyclohexylamino)-9*H*-purin-2-yl)amino)phenyl)piperazin-1-yl)-4-oxobutanamido) propyl)-*N*4-((R)-1-((2*S*,4*R*)-4-hydroxy-2-((4-(4-methylthiazol-5-yl)benzyl)carbamoyl)pyrrolidin-1-yl)-3,3-dimethyl-1-oxobutan-2-yl)succinimide, 56:** In a flask tert-butyl (3-(4-(((*S*)-1-((2*S*,4*R*)-4-hydroxy-2-((4-(4-methylthiazol-5-yl)benzyl)carbamoyl)pyrrolidin-1-yl)-3,3-dimethyl-1-oxobutan-2-yl)amino)-4-oxobutanamido)propyl)carbamate **54** (15 mg, 0.022 mmol) was dissolved in a mixture of dichloromethane/TFA 3/1 (0.35 mL) and the solution was stirred for 2h at ambient temperature. Afterwards the solvents were removed in rotary to afford the desired salt which was used in the next step without further purification. In a dried flask containing 4-(4-(4-((6-(cyclohexylamino)-9*H*-purin-2-yl)amino)phenyl)piperazin-1-yl)-4-oxobutanoic acid (10 mg, 0.02 mmol) dry DMF (0.3 mL), 3-(4-(((*R*)-1-((2*S*,4*R*)-4-hydroxy-2-((4-(4-methylthiazol-5-yl)benzyl)carbamoyl)pyrrolidin-1-yl)-3,3-dimethyl-1-oxobutan-2-yl)amino)-4-oxobutanamido)propan-1-aminium 2,2,2-trifluoroacetate (16 mg, 0.022 mmol), HATU (15 mg, 0.041 mmol) and DIPEA (16 mg, 0.12 mmol) were added under Ar atmosphere. The reaction mixture was stirred overnight at room temperature. After the reaction's completion the solvent was evaporated and the crude mixture was purified via preparative HPLC to afford the desired product as a white solid (4 mg, yield 19%). **56:** ^{1}H NMR (500 MHz, DMSO-*d*6)) δ 12.29 (br, 1H), 8.98 (s, 1H), 8.55 (m, 2H), 7.90 (d, *J* = 9.3 Hz, 1H), 7.79 (m, 2H), 7.74 (br, 1H), 7.67 (d, *J* = 8.6 Hz, 2H), 7.40 (d, *J* = 8.1 Hz, 2H), 7.34 (d, *J* = 8.1 Hz, 2H), 7.05 (br, 1H), 6.85 (d, *J* = 8.6 Hz, 2H), 5.13 (br, 1H), 4.52 (d, *J* = 9.3 Hz, 1H), 4.42 (dd, *J* = 15.1, 6.8 Hz, 2H), 4.35 (br, 1H), 4.22 (dd, *J* = 15.7, 5.1 Hz, 1H), 3.67 – 3.58 (m, 6H), 3.03 (d, *J* = 5.5 Hz, 6H), 2.96 (br, 2H), 2.63 – 2.56 (m, 3H), 2.44 (s, 3H), 2.33 (m, 5H), 2.07 – 2.00 (m, 1H), 1.96 – 1.86 (m, 3H), 1.77 (m, 2H), 1.64 (m, 1H), 1.49 (m, 2H), 1.34 (m, 5H), 1.16 (m, 1H), 0.93 (s, 9H); ^{13}C NMR (75 MHz, DMSO-*d*6)) δ 172.0, 171.4, 170.0, 169.9, 169.6, 156.5, 155.5, 151.5, 147.8, 147.7, 144.9, 139.5, 135.2, 131.2, 129.7, 128.7, 127.5, 122.4, 119.3, 118.0, 116.8, 68.9, 58.7, 56.4, 56.3, 50.1, 49.7, 44.8, 41.7, 41.2, 37.9, 36.4, 35.3, 32.7, 31.0, 30.6, 30.4, 27.9, 26.4, 25.3, 25.2, 16.0; ESI-HRMS m/z for $C_{54}H_{73}N_{14}O_7S$ $[M+H]^+$ calcd 1061.5507, found 1061.5502.

Plasma stability

First, 250 µg of conjugate **20** were diluted in 5 µL DMSO and the mixture was added to 0.5 mL of human plasma. The mixture was incubated at 37 °C. 50 µL of aliquot was removed at predetermined time points and quenched with 150 µL ice-cold acetonitrile (+0.1% formic acid). Then, the mixture was centrifuged at 10,000 rpm for 10 min. Then, 50 µL of supernatant was added to 50 µL of ultrapure water (+0.1% formic acid) and analyzed by LC-MS. Results are presented as the mean ± SD of three independent experiments.

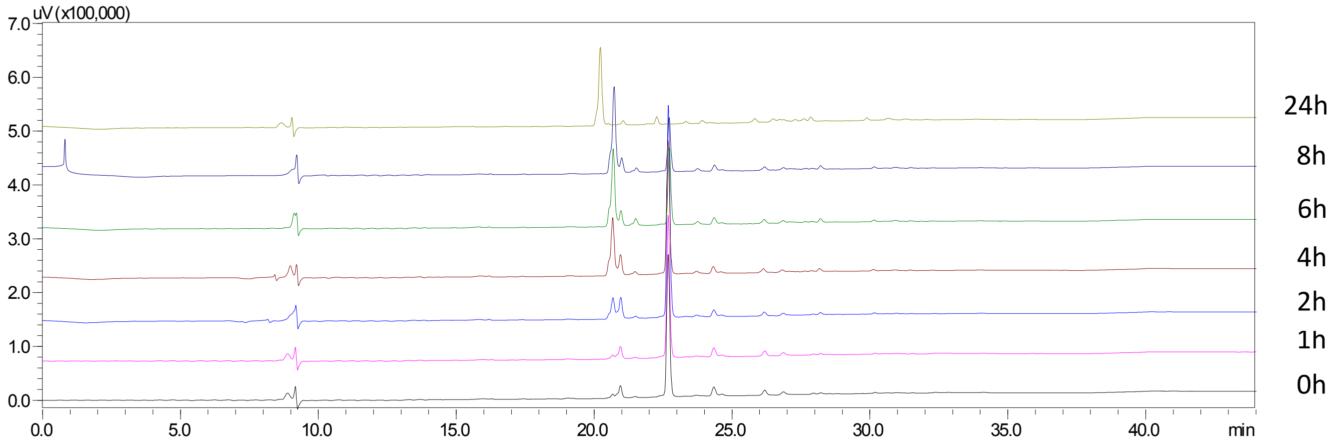

Figure S8

Stability in Buffer Solutions

The stability of the conjugate was examined by performing chemostability experiments at two different buffer solutions (pH = 5.2 and 7.4). Conjugate **20** was dissolved in 5 µL DMSO and transferred to 0.5 mL of the relevant buffer solution (acetate or phosphate aquatic buffer solutions). The mixture was then incubated at 37 °C, samples were collected at predetermined time points (0, 1, 2, 3, 4, 5, 24 and 48 h), and analyzed by LC-MS. Results are presented as the mean ± of SD after repeating the experiment three times.

pH=5.2

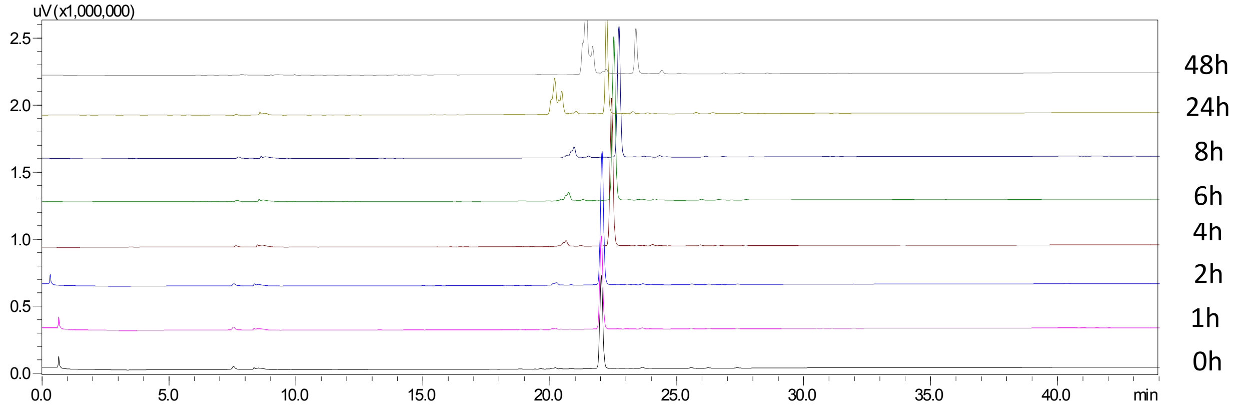

Figure S9: ESI-LCMS charts detected at 254 nm of conjugate **20** at pH5.2 and selected time intervals

pH=7.4

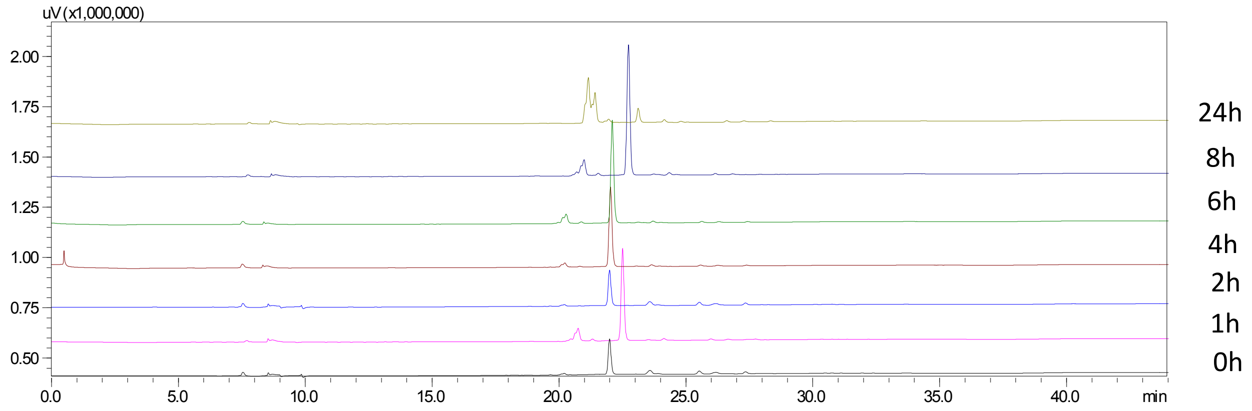

Figure S10: ESI-LCMS charts detected at 254 nm of conjugate **20** at pH7.4 and selected time intervals

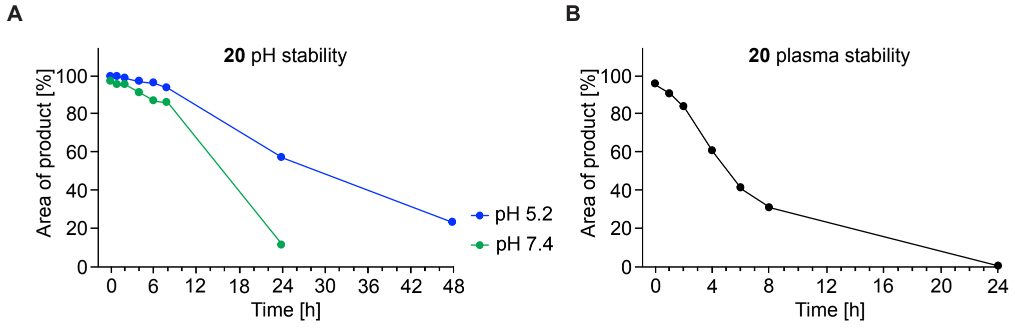

Figure S11: Chemostability of degrader 20. (A-B) LC-ESI-MS based measurement of **20** under various conditions. Stability of **20** was analyzed at pH 5.2 and 7.4 (A) or in human plasma (B) using LC-ESI-MS at indicated time points.

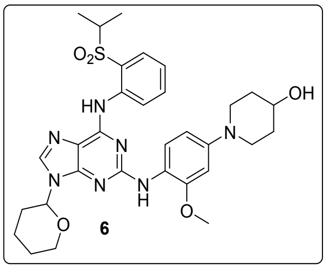

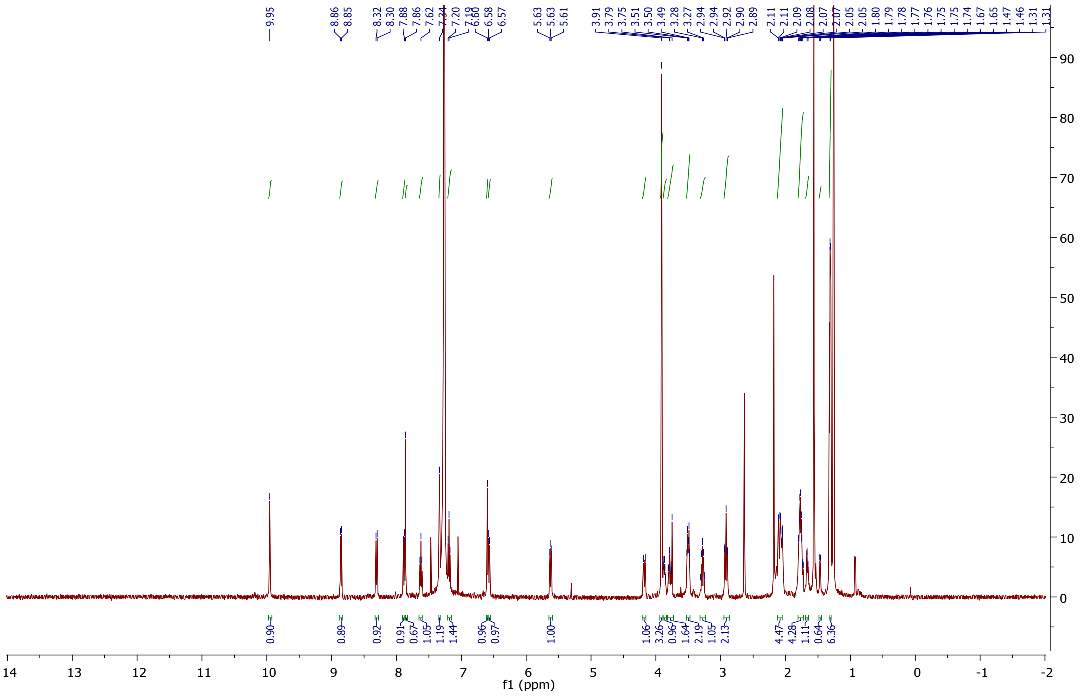

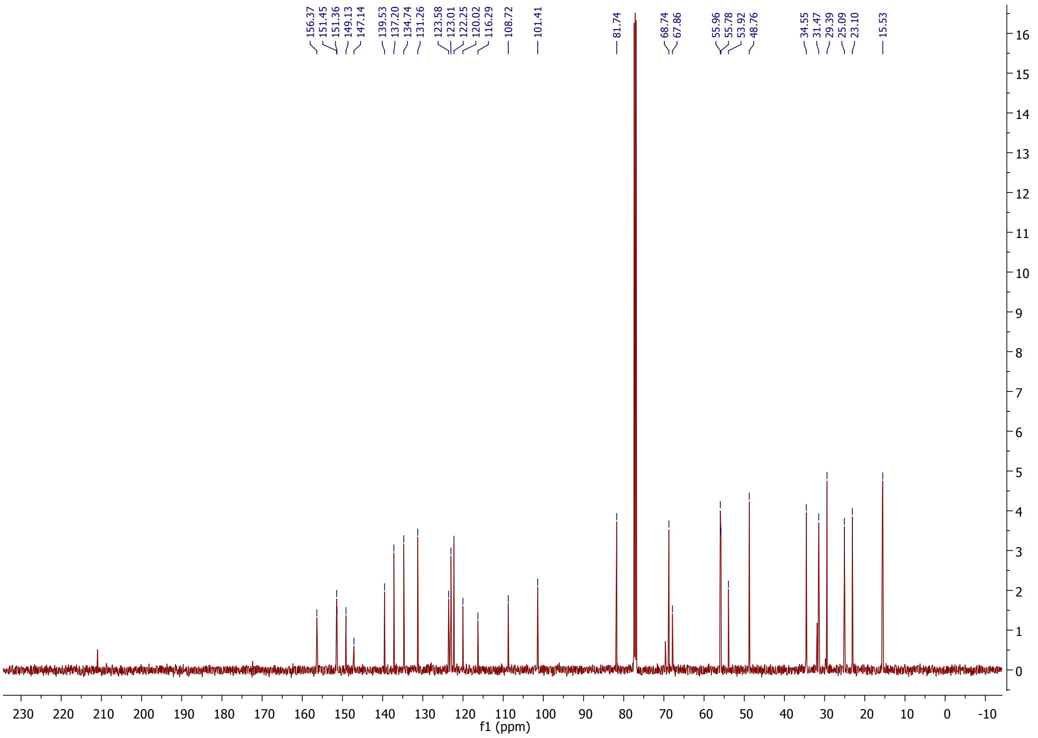

Figure S12

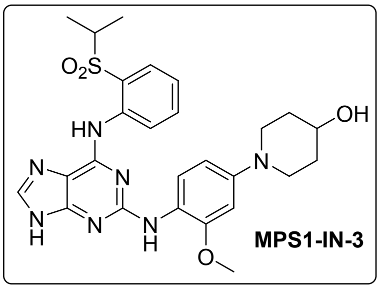

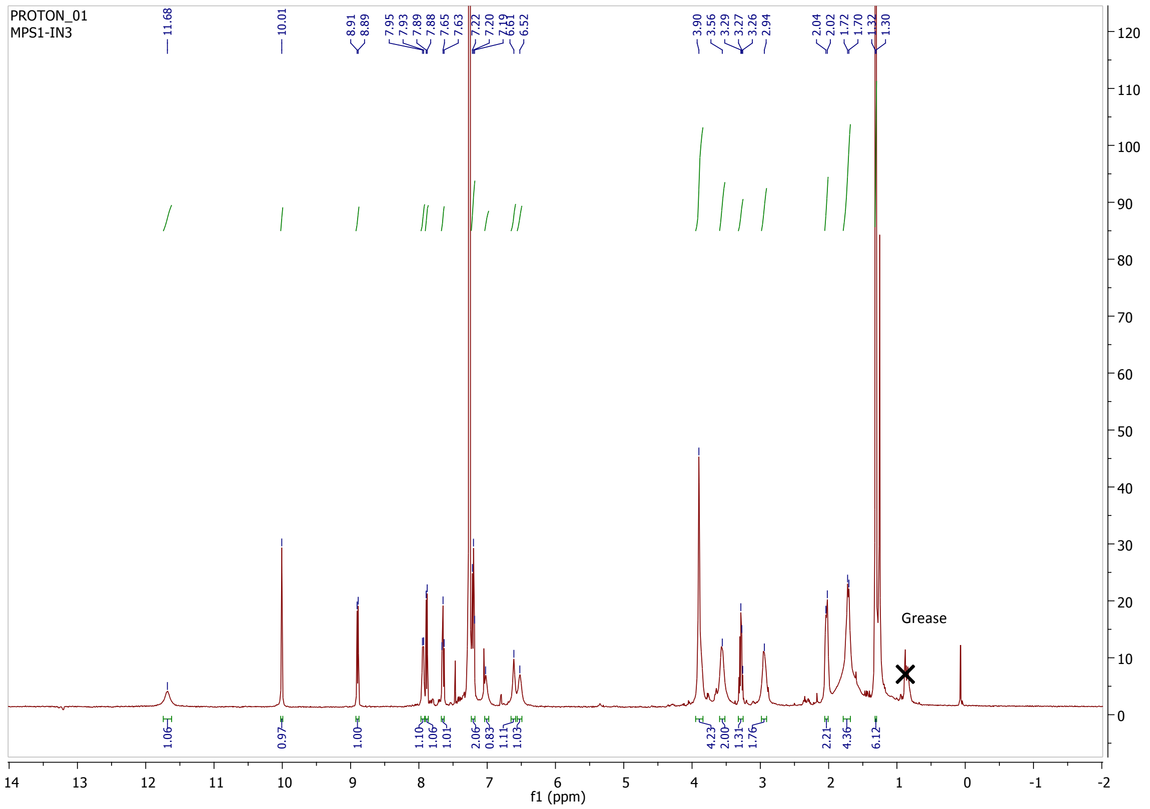

Figure S13

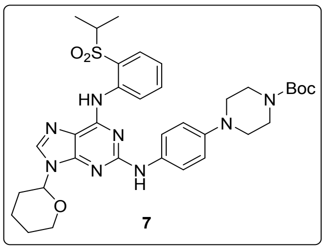

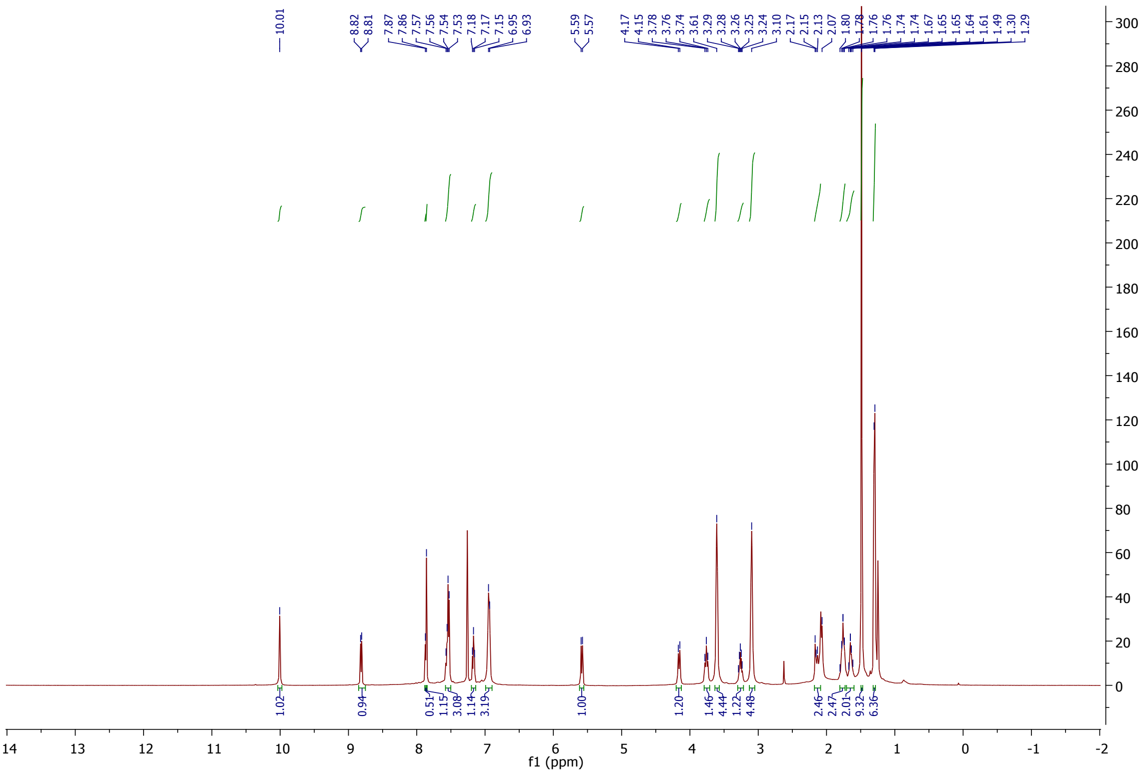

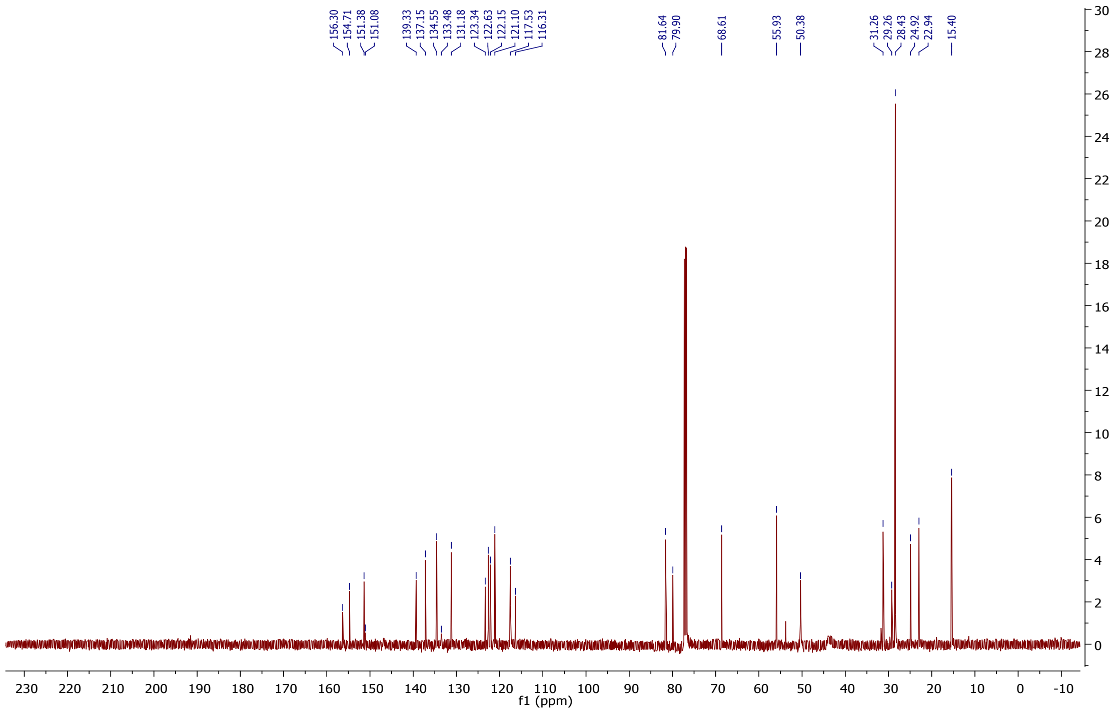

Figure S14

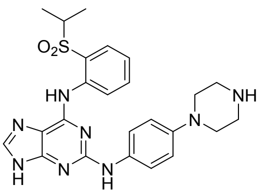

SF1

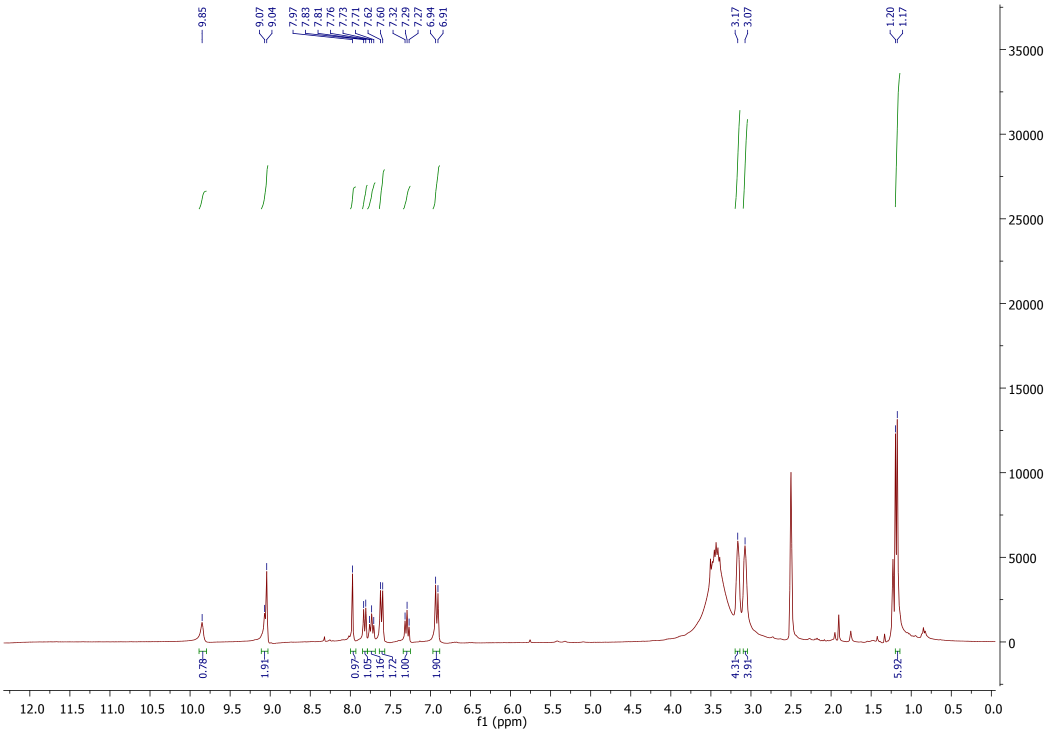

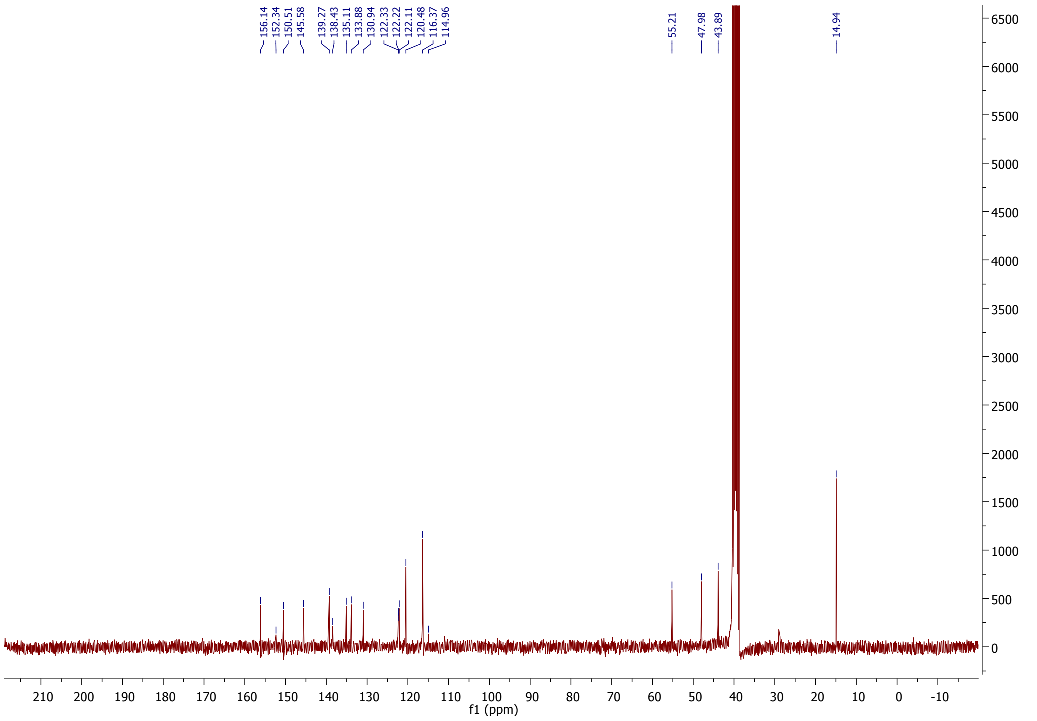

Figure S15

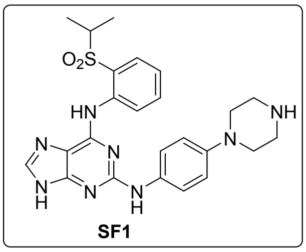

SF1

Analytical method 2

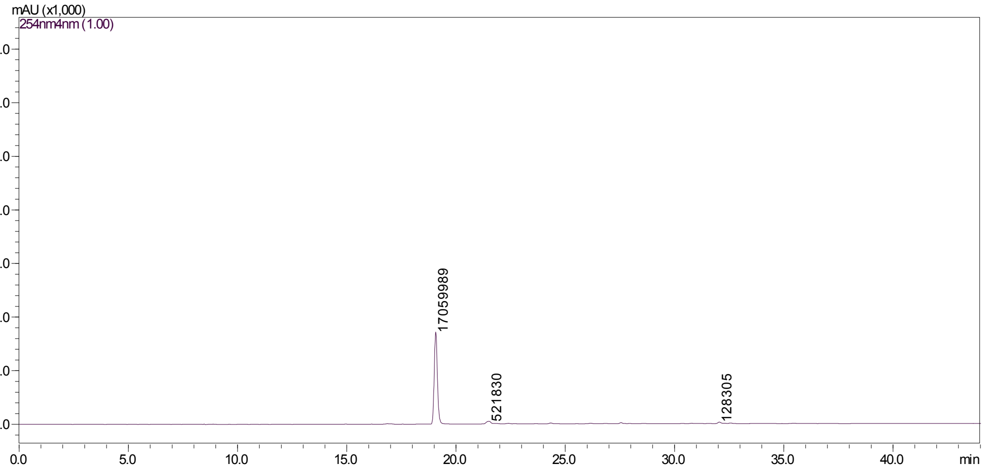

Area= 17059989

Total Area= 17710124

(%)=96.3%

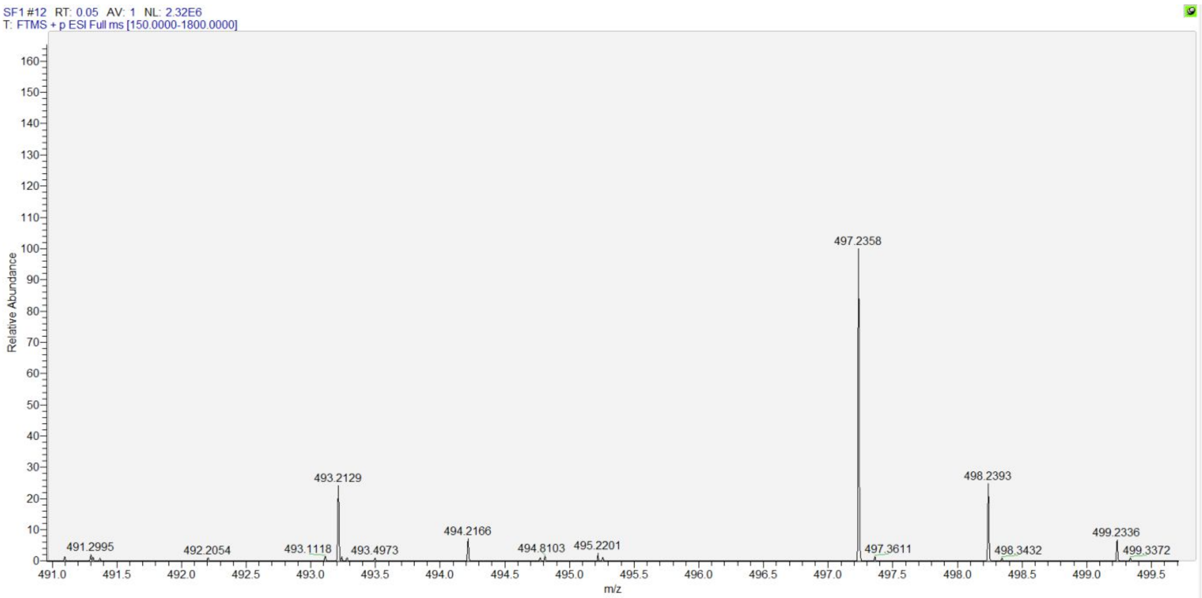

ESI-HRMS m/z for $C_{24}H_{29}N_8O_2S$ $[M+H]^+$ calcd 493.2134, found 493.2129.

Figure S16

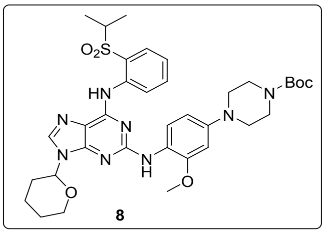
O2S
HN
N
N
N
N
N
H
O
O
N
N
Boc

8

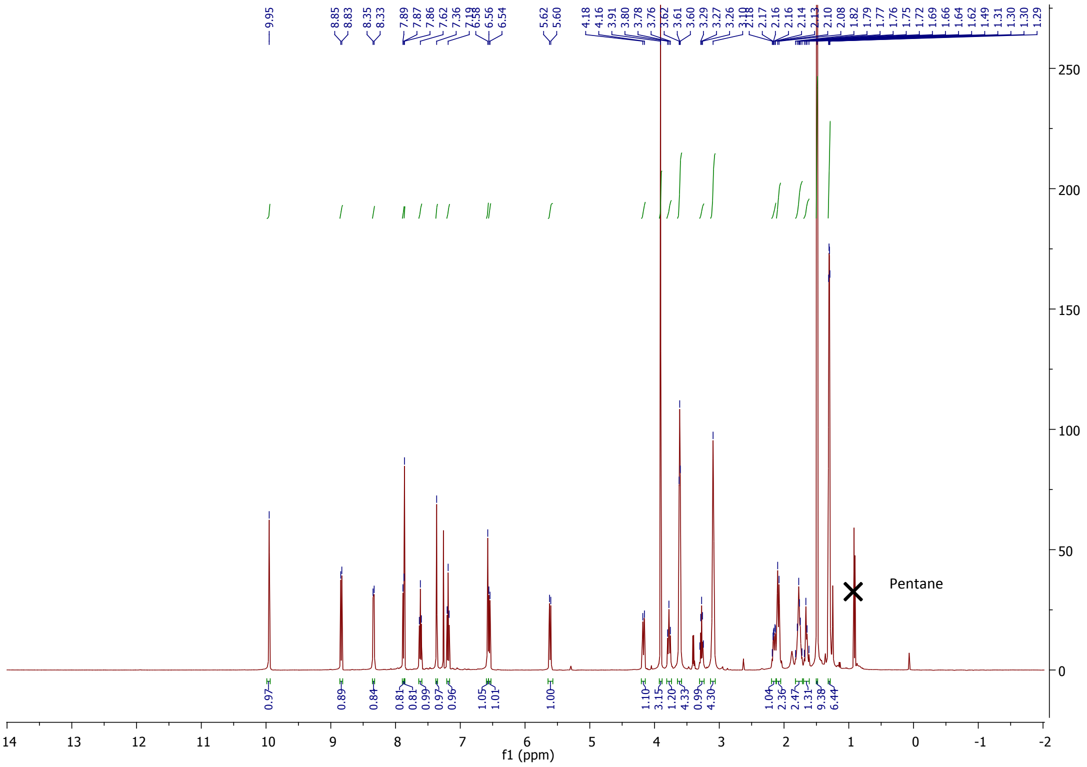
9.95
8.85
8.83
8.35
8.33
7.89
7.87
7.86
7.62
7.36
7.19
6.58
6.56
6.54
5.62
5.60
4.18
4.16
3.91
3.80
3.78
3.76
3.62
3.61
3.60
3.29
3.27
3.26
3.10
2.18
2.17
2.16
2.16
2.14
2.13
2.10
2.08
1.82
1.79
1.77
1.76
1.75
1.72
1.69
1.66
1.64
1.62
1.49
1.31
1.30
1.29
Pentane
0.97
0.89
0.84
0.81
0.81
0.99
0.97
0.96
1.05
1.01
1.00
1.10
3.15
1.20
4.33
0.99
4.30
1.04
2.36
2.47
1.31
9.38
6.44
f1 (ppm)

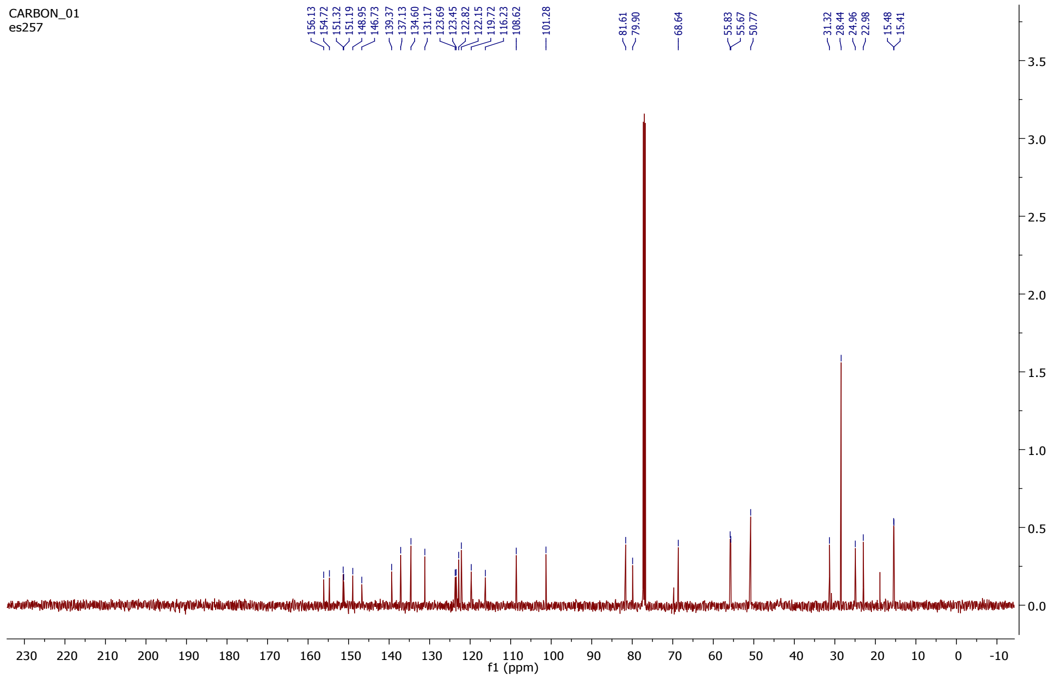
CARBON_01
es257
156.13
154.72
151.32
151.19
148.95
146.73
139.37
137.13
134.60
131.17
123.69
123.45
122.82
122.15
119.72
116.23
108.62
101.28
81.61
79.90
68.64
55.83
55.67
50.77
31.32
28.44
24.96
22.98
15.48
15.41
f1 (ppm)

Figure S17

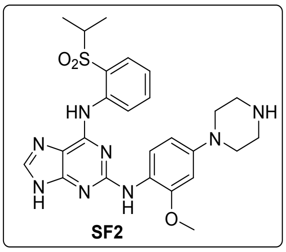
O2S
HN
N
N
NH
N
N
H
N
H
O
SF2

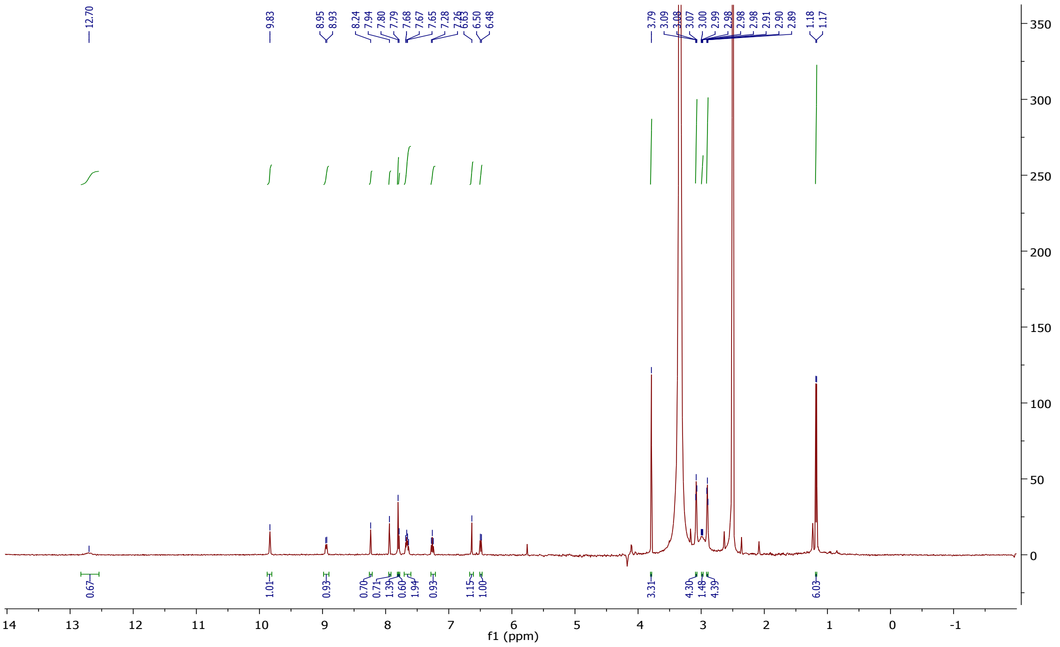
12.70
9.83
8.95
8.93
8.24
7.94
7.80
7.79
7.68
7.67
7.65
7.28
7.26
6.63
6.50
6.48
3.79
3.09
3.08
3.07
3.00
2.99
2.98
2.98
2.98
2.91
2.90
2.89
1.18
1.17
0.67
1.01
0.93
0.70
0.71
1.39
0.60
1.94
0.93
1.15
1.00
3.31
4.30
1.48
4.39
6.03
f1 (ppm)

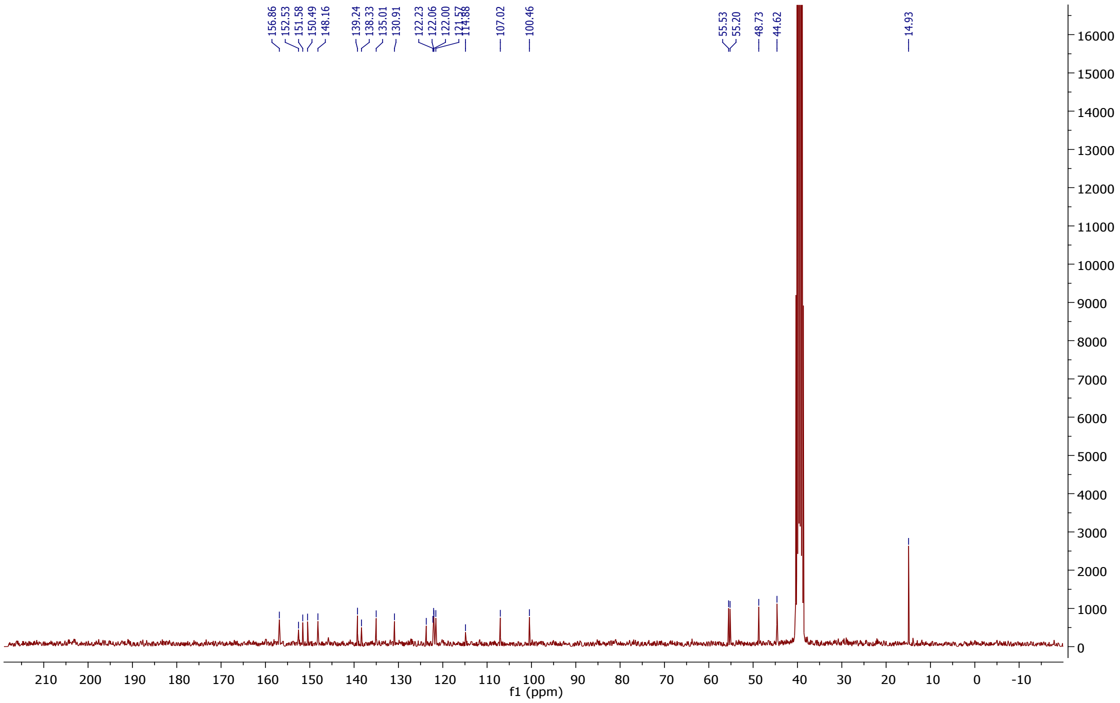
156.86
152.53
151.58
150.49
148.16
139.24
138.33
135.01
130.91
122.23
122.06
122.00
121.57
114.88
107.02
100.46
55.53
55.20
48.73
44.62
14.93
f1 (ppm)

Figure S18

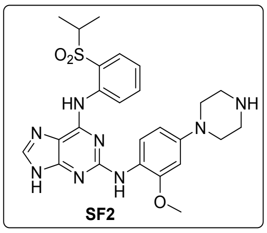

Analytical method 1

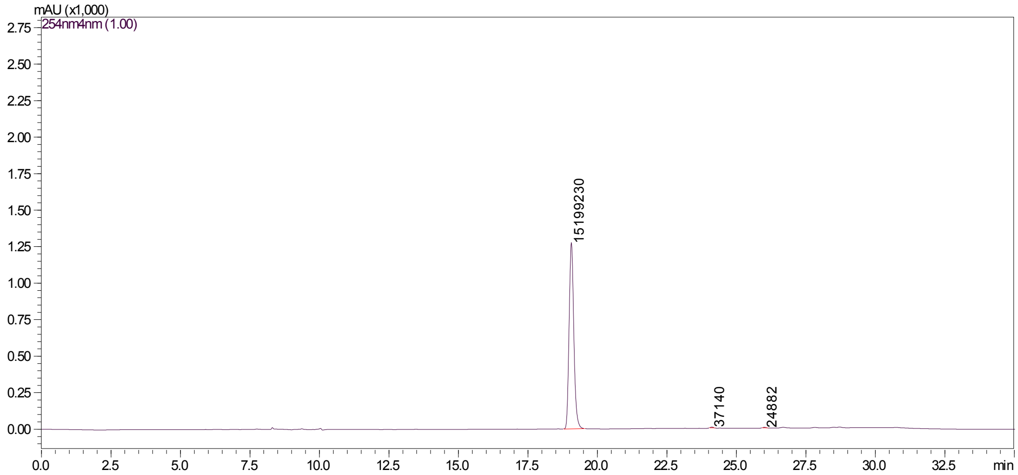

total area: 15199230

peak area: 15261252

Purity (%)=99.6%

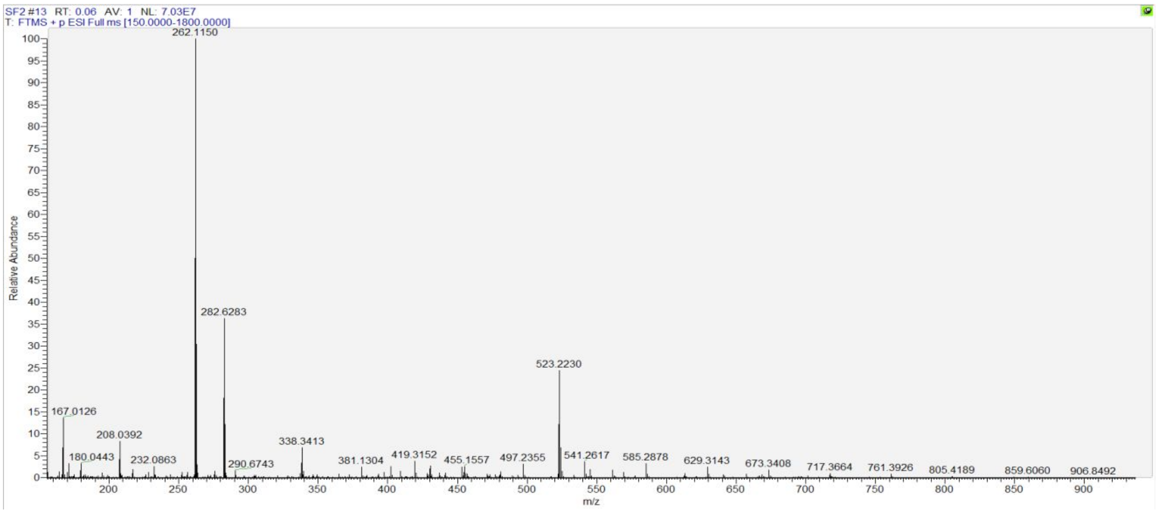

ESI-HRMS m/z for $C_{25}H_{32}N_8O_3S$ [M+H]+ calcd 523.2240, found 523.2230.

Figure S19

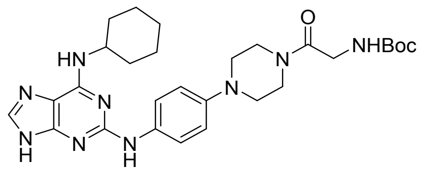
O
NHBoc
HN
N
N
N
N
H
N
H
N
N

9

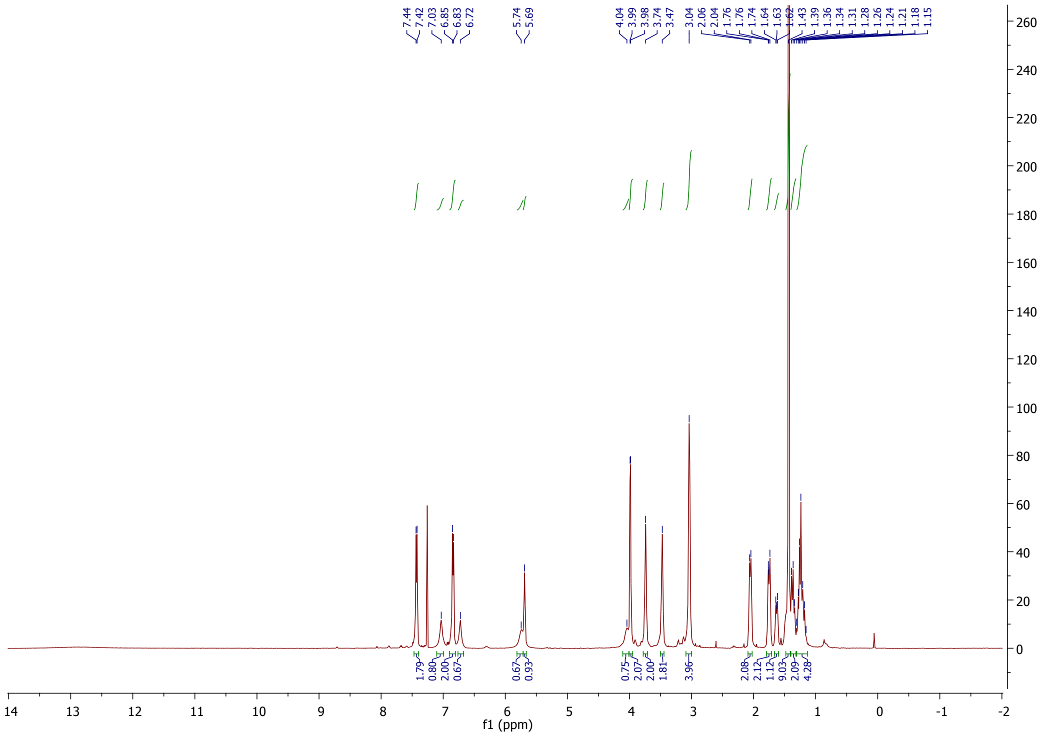
7.44
7.42
7.03
6.85
6.83
6.72
5.74
5.69
4.04
3.99
3.98
3.74
3.47
3.04
2.06
2.04
1.76
1.76
1.74
1.64
1.63
1.62
1.43
1.39
1.36
1.34
1.31
1.28
1.26
1.24
1.21
1.18
1.15
1.79
0.80
2.00
0.67
0.67
0.93
0.75
2.07
2.00
1.81
3.96
2.08
2.12
1.12
9.03
2.09
4.28
f1 (ppm)

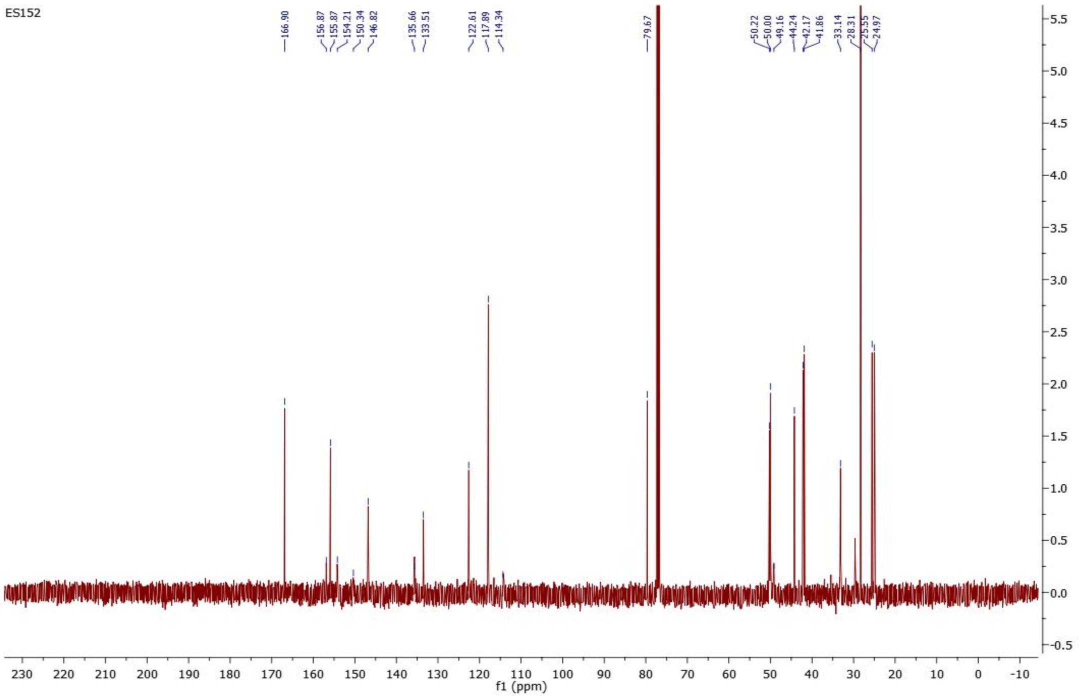
ES152
166.90
156.87
155.87
154.21
150.34
146.82
135.66
133.51
122.61
117.89
114.34
79.67
50.22
50.00
49.16
44.24
42.17
41.86
33.14
28.31
25.55
24.97
f1 (ppm)

Figure S20

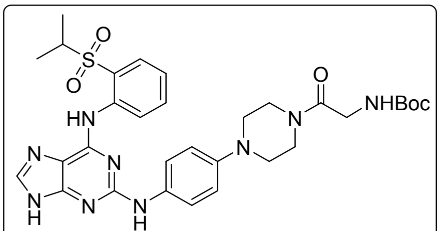

10

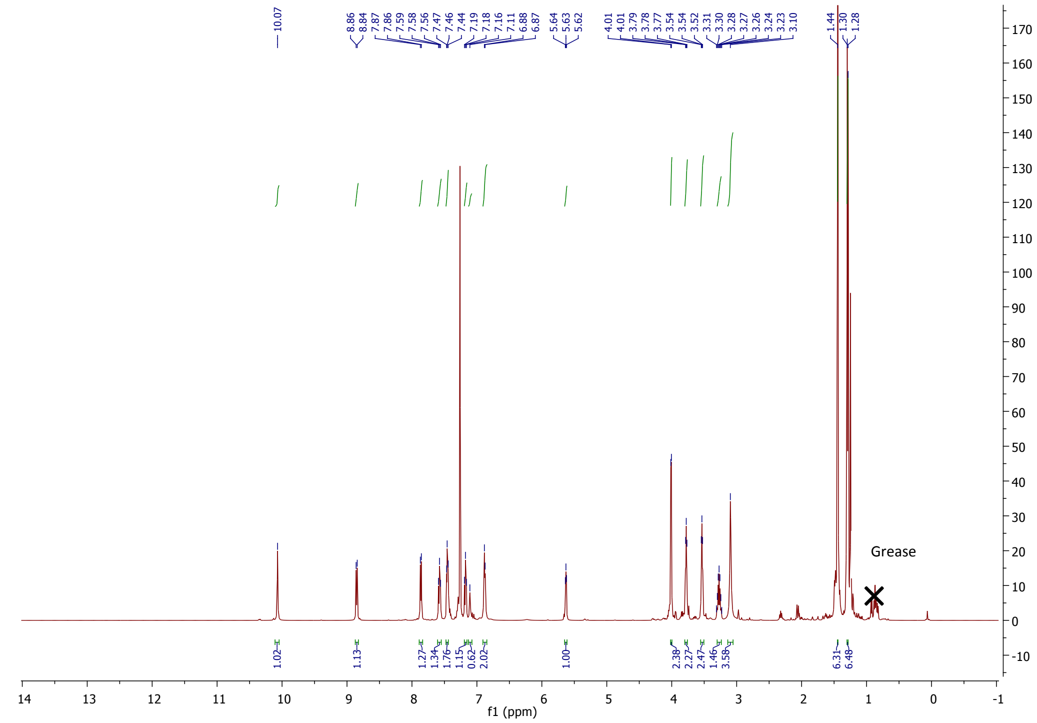

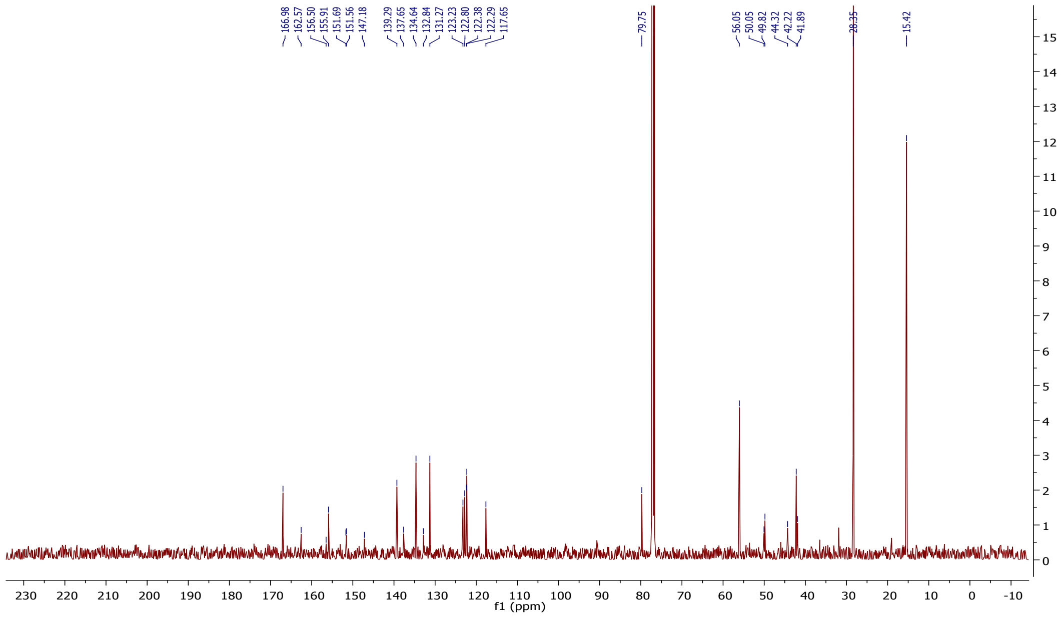

Figure S21

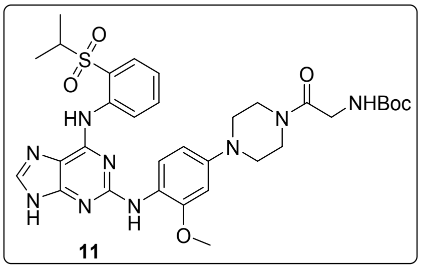

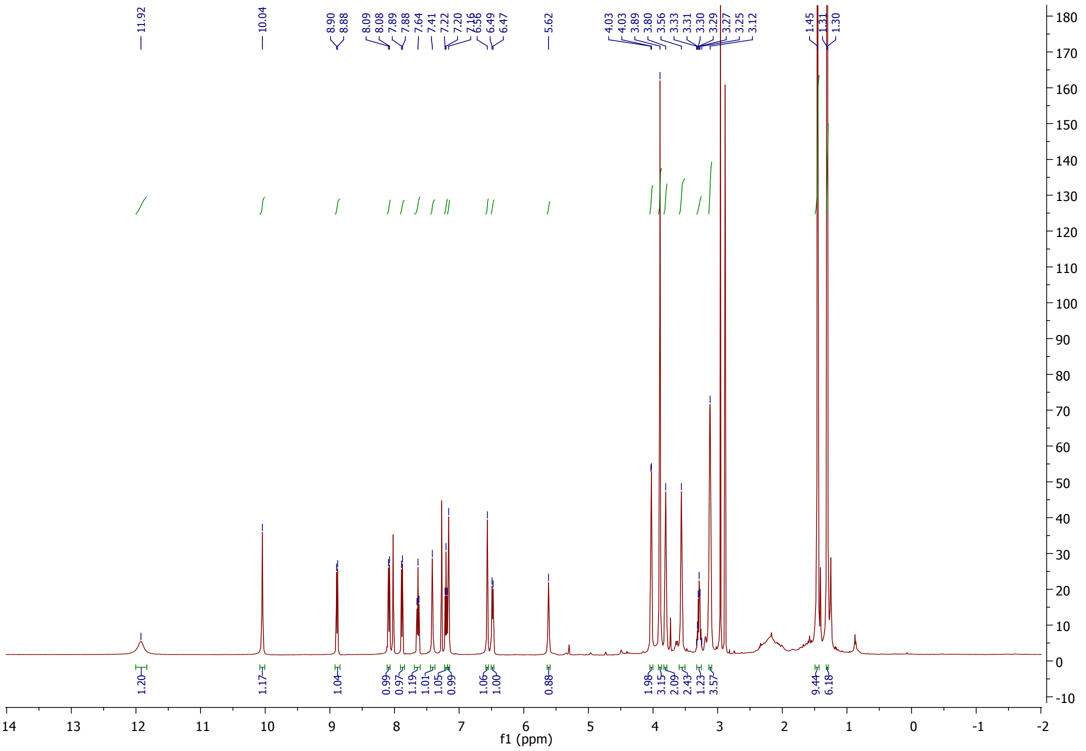

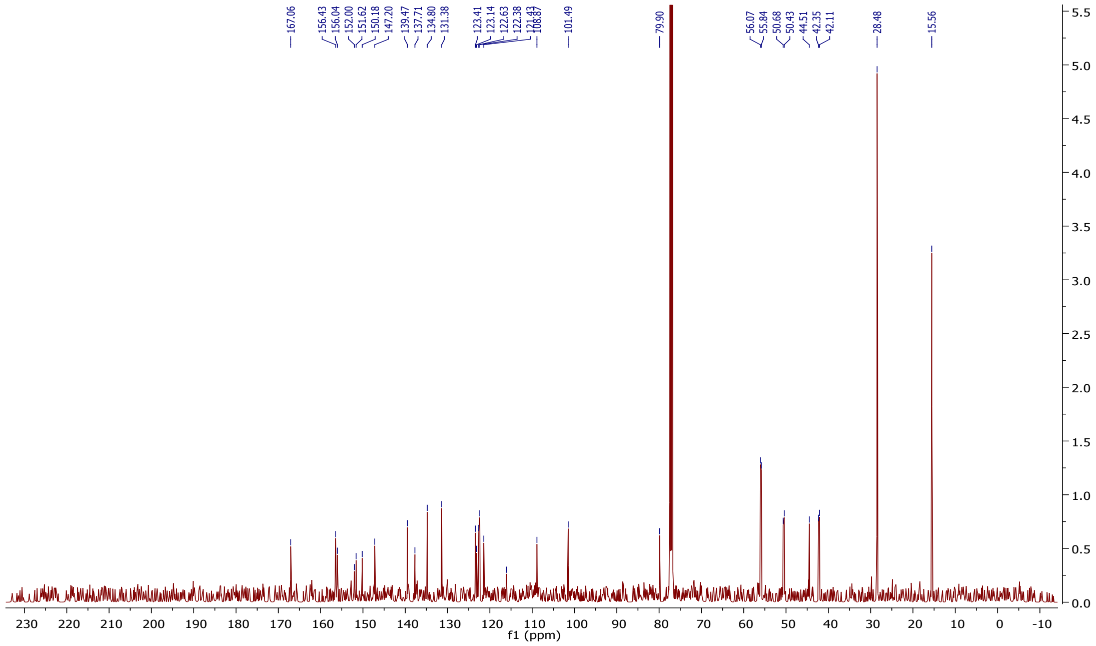

Figure S22

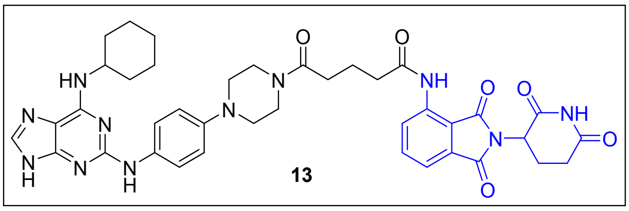

13

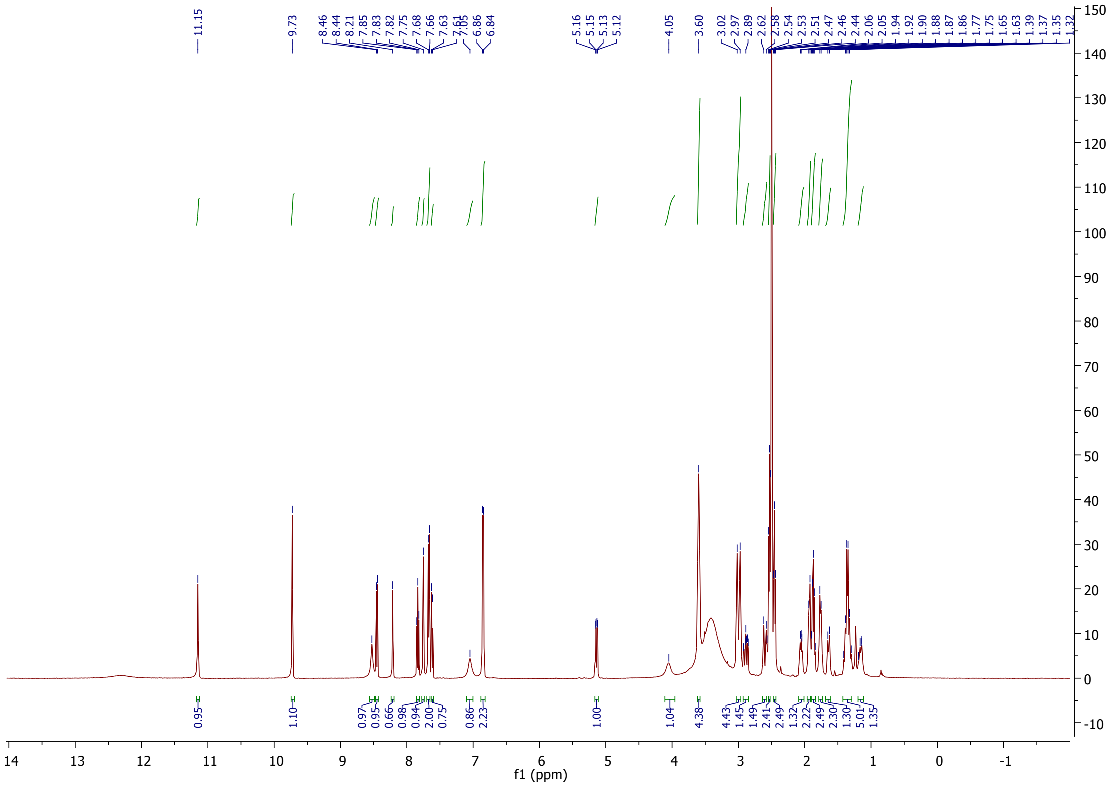

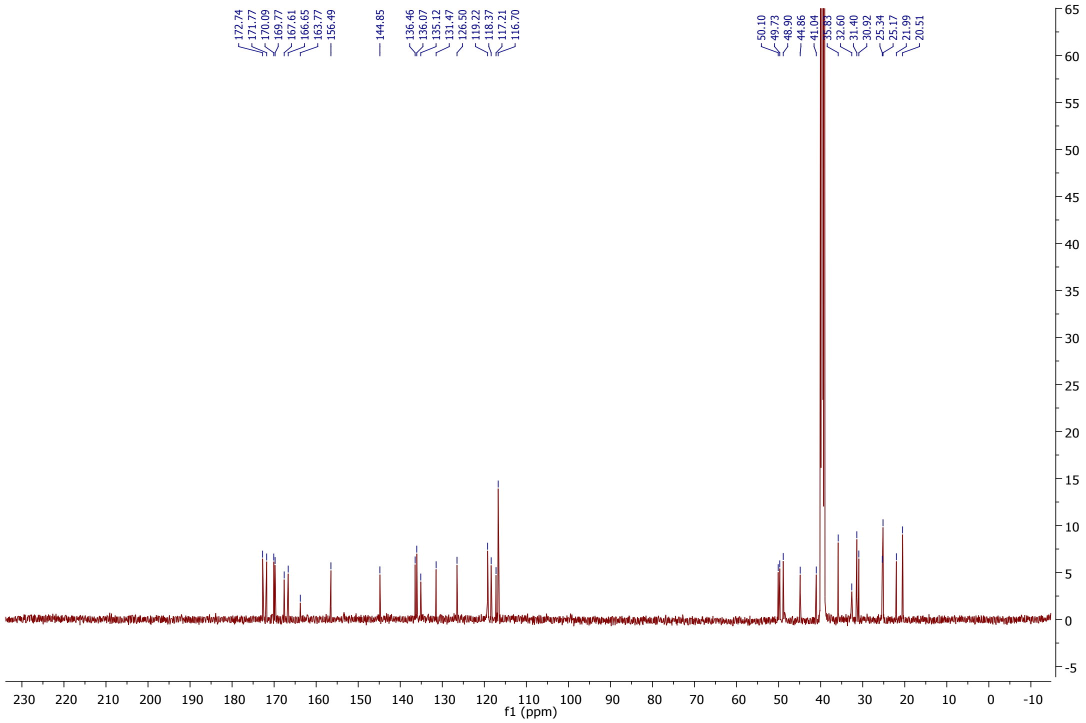

Figure S23

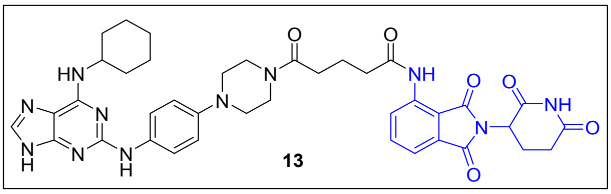

Analytical method 2

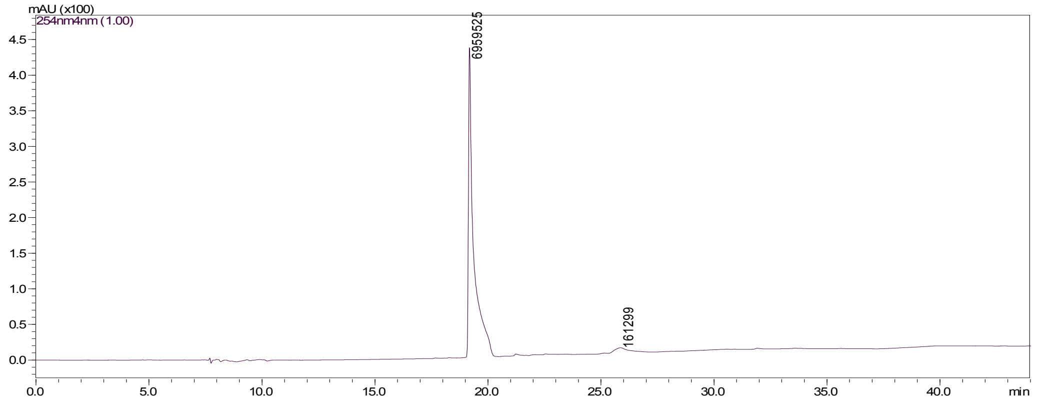

Area= 8959525

Total Area= 9120824

Purity (%)=98.2%

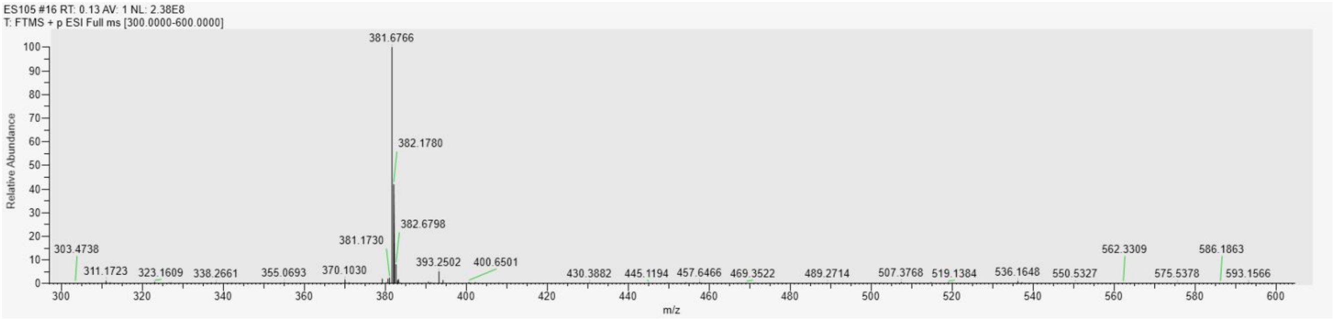

ESI-HRMS m/z for $C_{39}H_{44}N_{11}O_6$ [M/2+H]+ calcd 381.6766, found 381.6766.

Figure S24

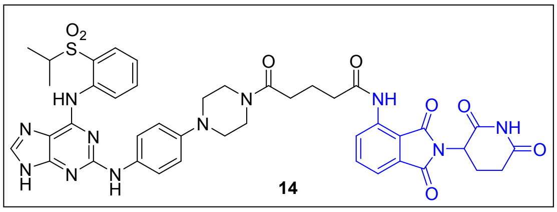

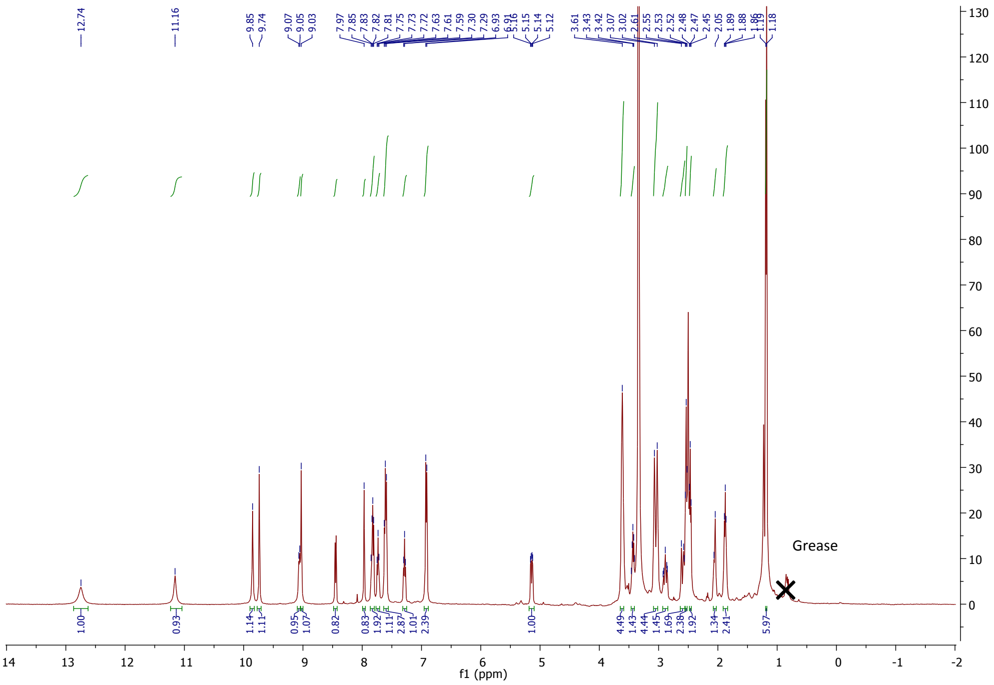

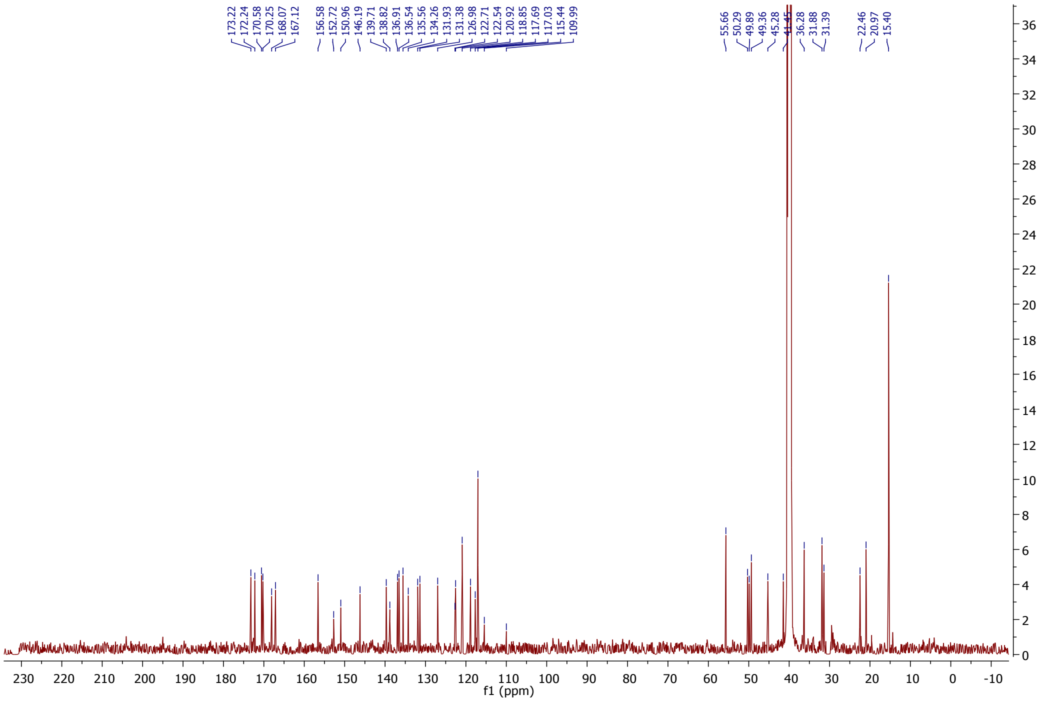

Figure S25

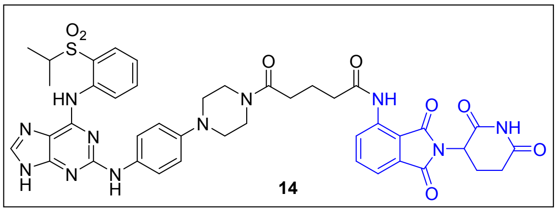

Analytical method 2

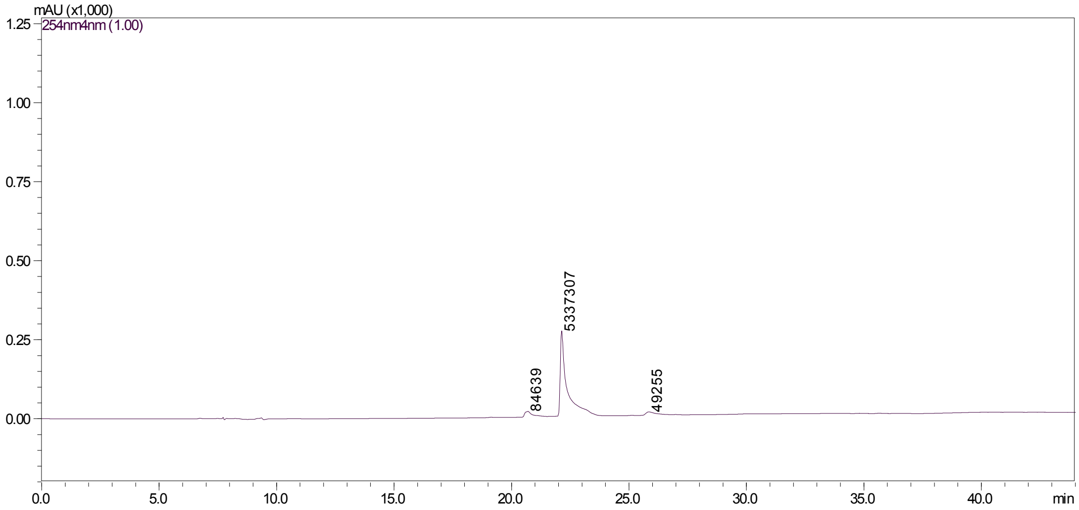

Area= 5337307

Total Area= 5471255

(%)=97.5%

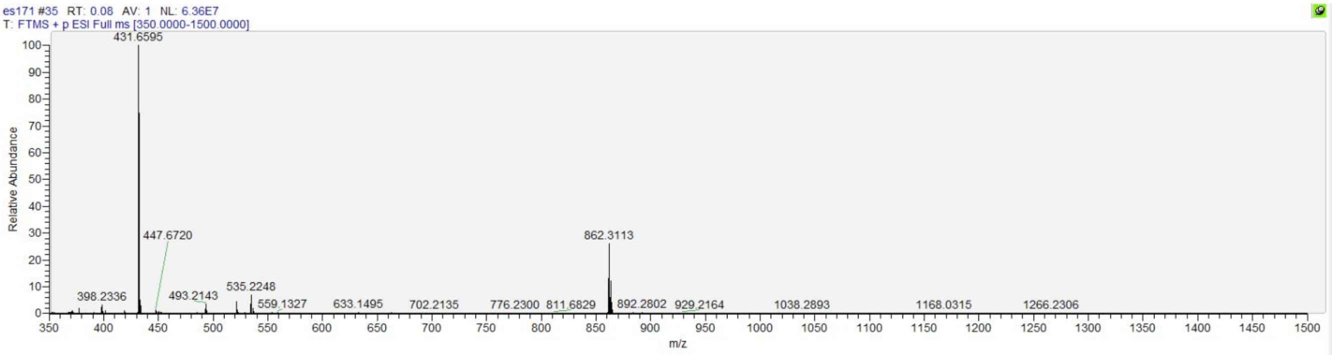

ESI-HRMS m/z for $C_{42}H_{44}N_{11}O_8S$ $[M+H]^+$ calcd 862.3090, found 862.3113.

Figure S26

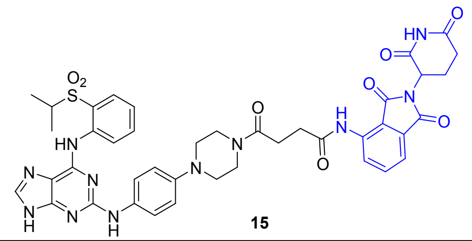

15

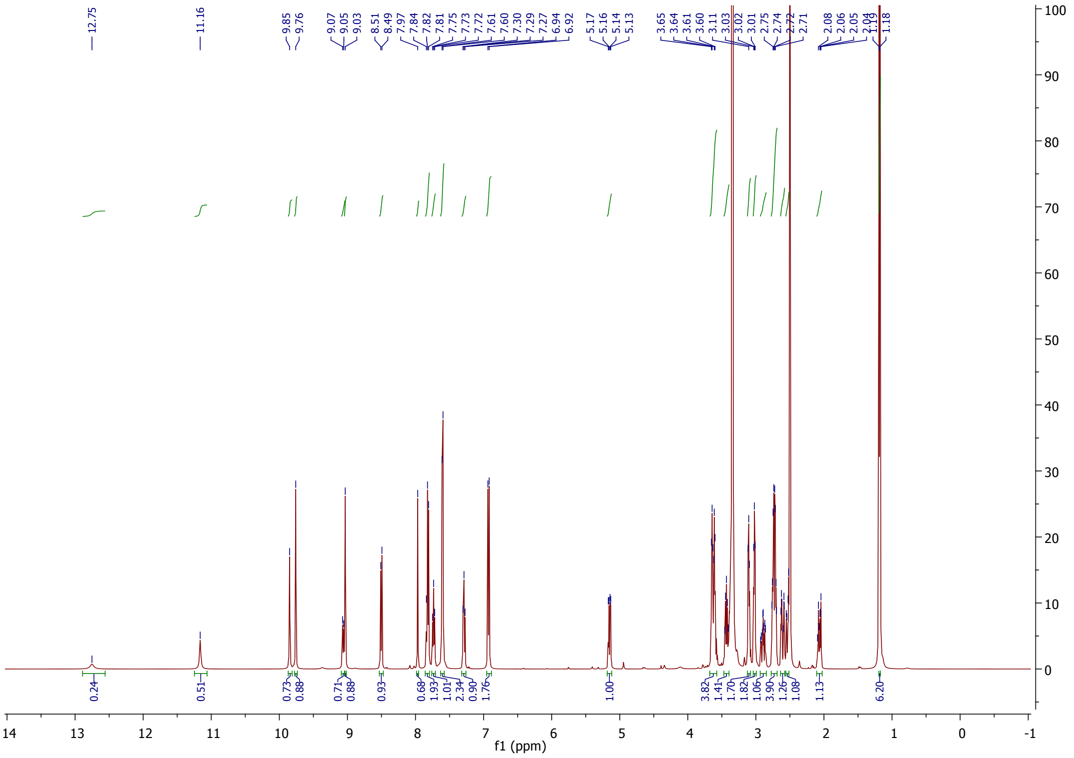

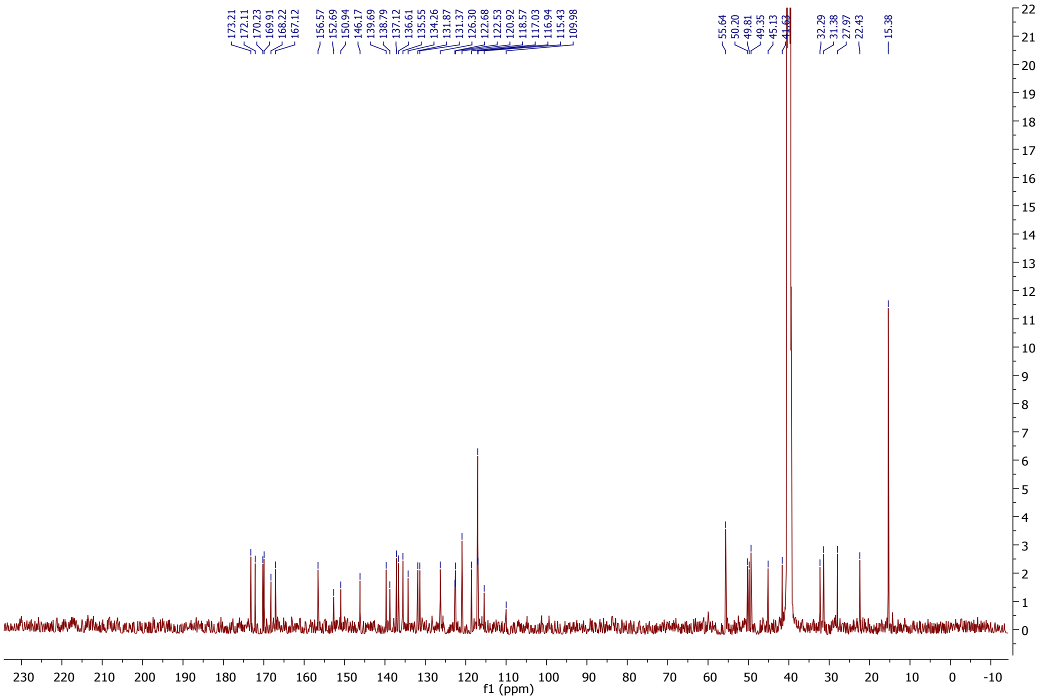

Figure S27

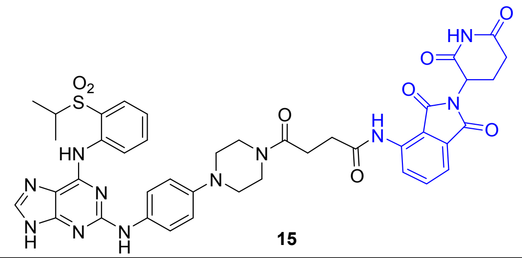

15

Analytical method 1

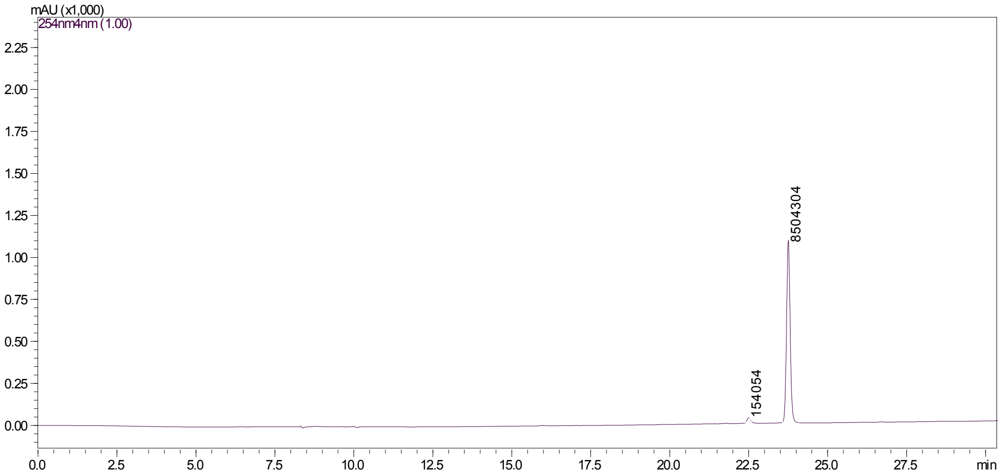

Area: 8287731

Total area: 8497746

Purity (%)=97.5%

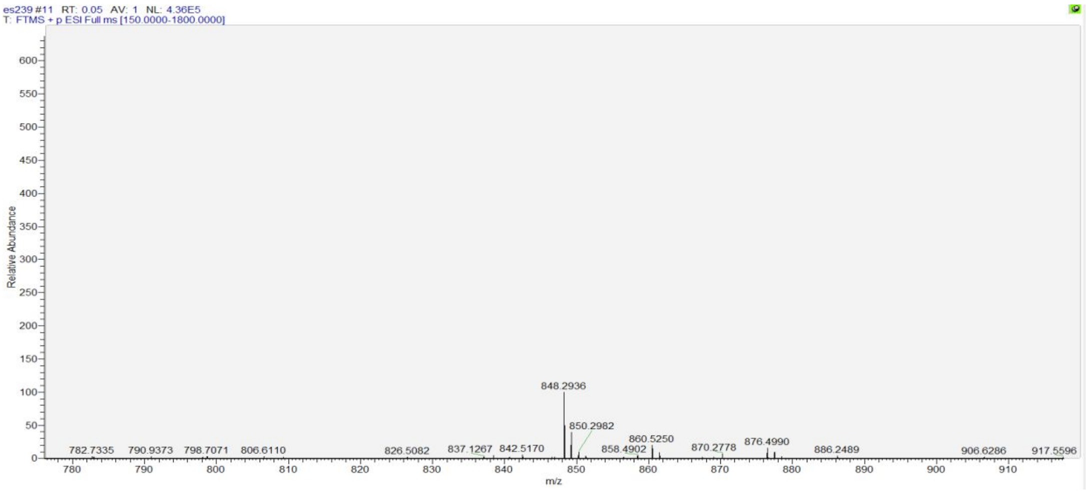

ESI-HRMS m/z for $C_{41}H_{42}N_{11}O_8S$ $[M+H]^+$ calcd 848.2939, found 848.2936.

Figure S28

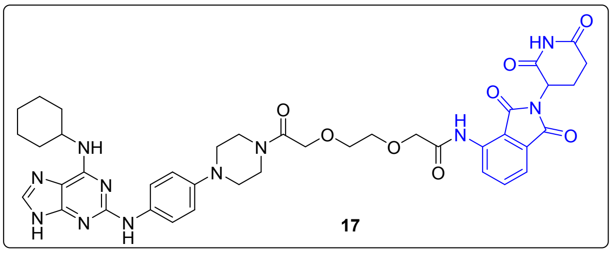

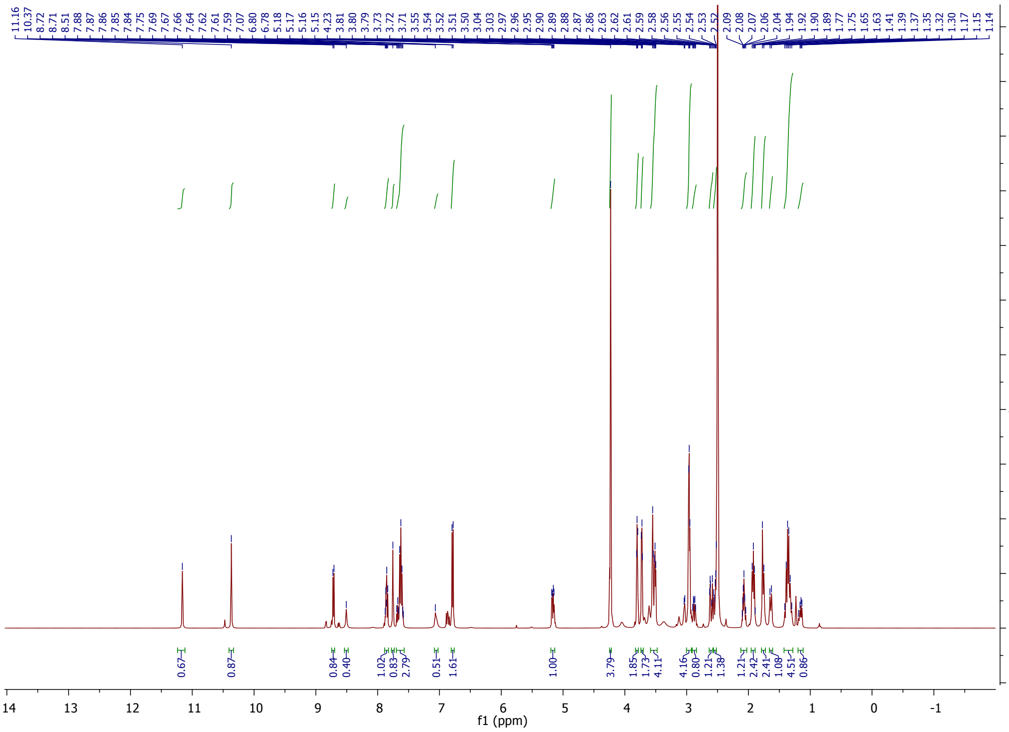

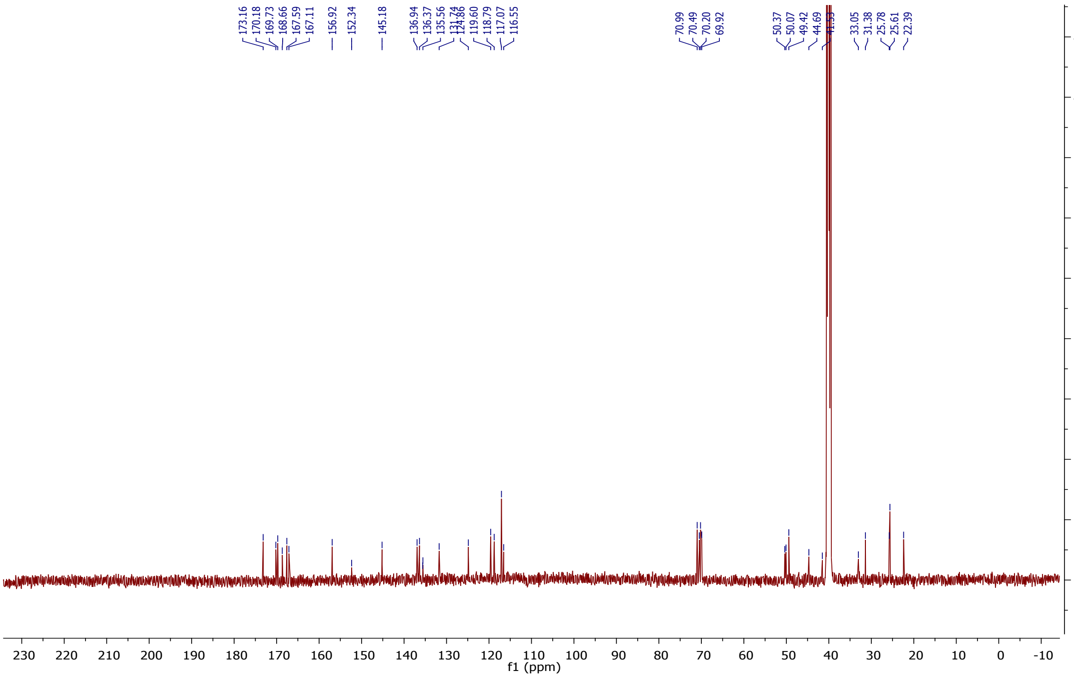

Figure S29

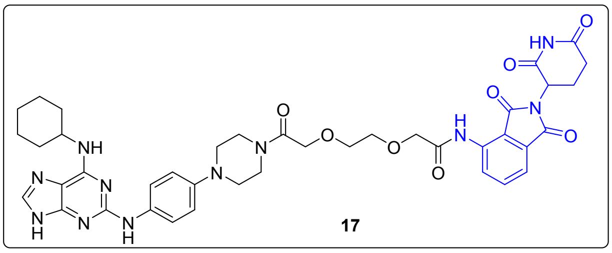

17

Analytical method 2

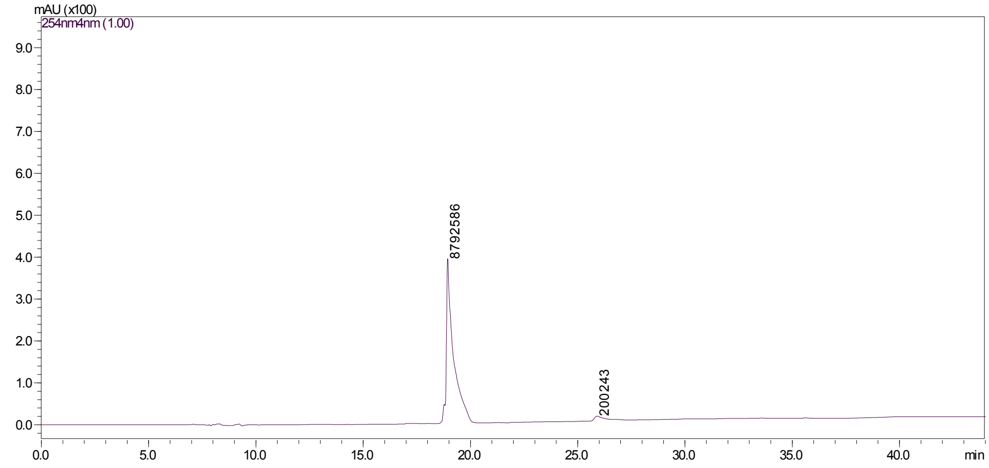

Area= 8792586

Total Area= 8992829

Purity (%)=97.7%

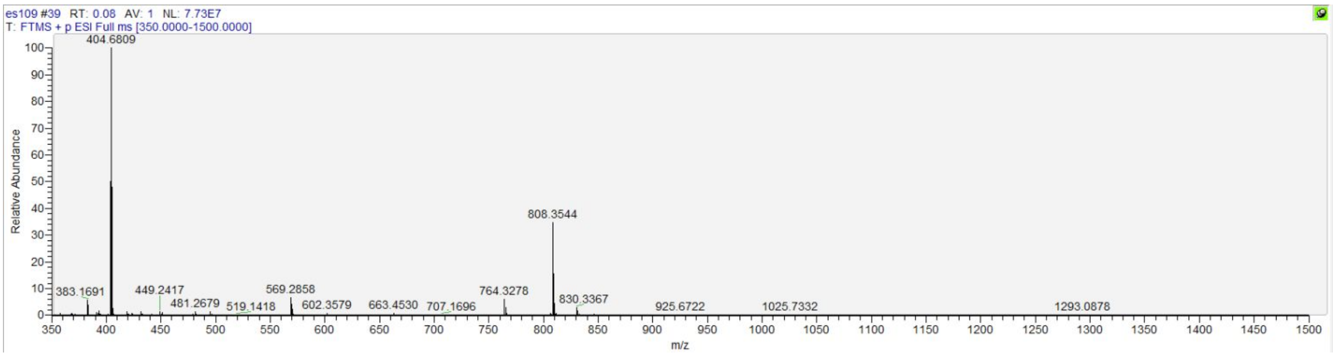

ESI-HRMS m/z for $C_{40}H_{46}N_{11}O_8$ $[M+H]^+$ calcd 808.3525, found 808.3544.

Figure S30

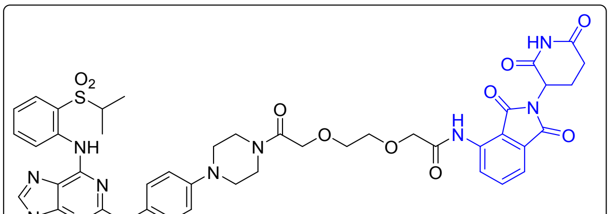

18

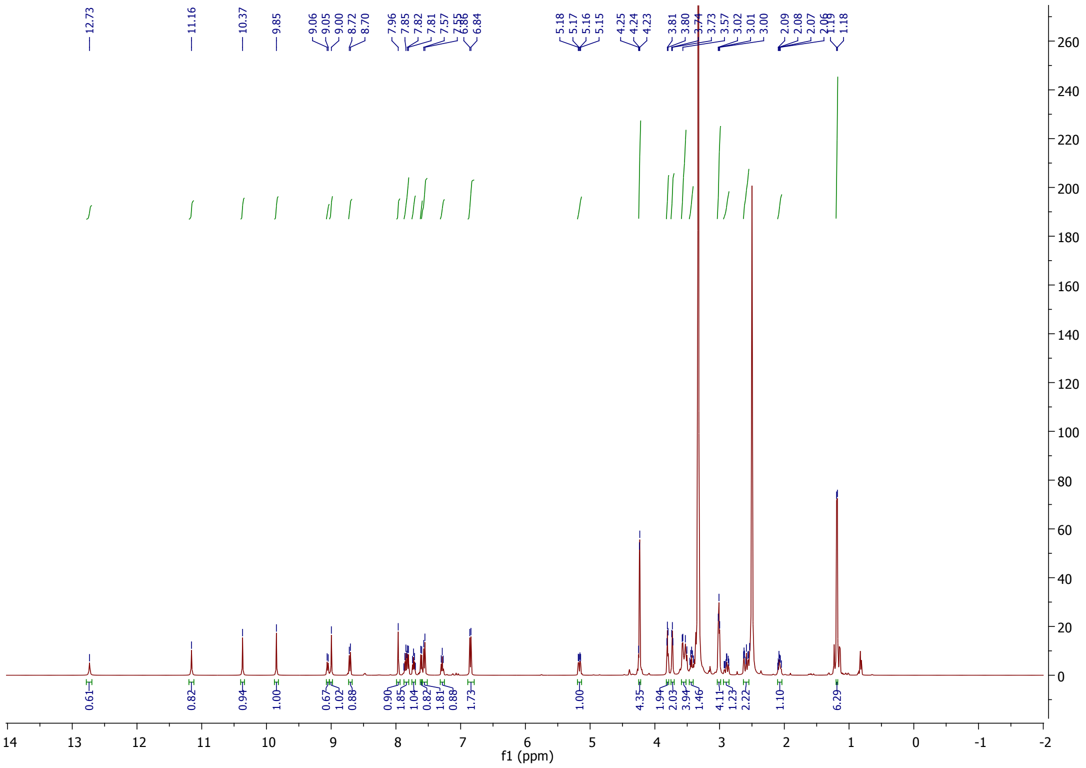

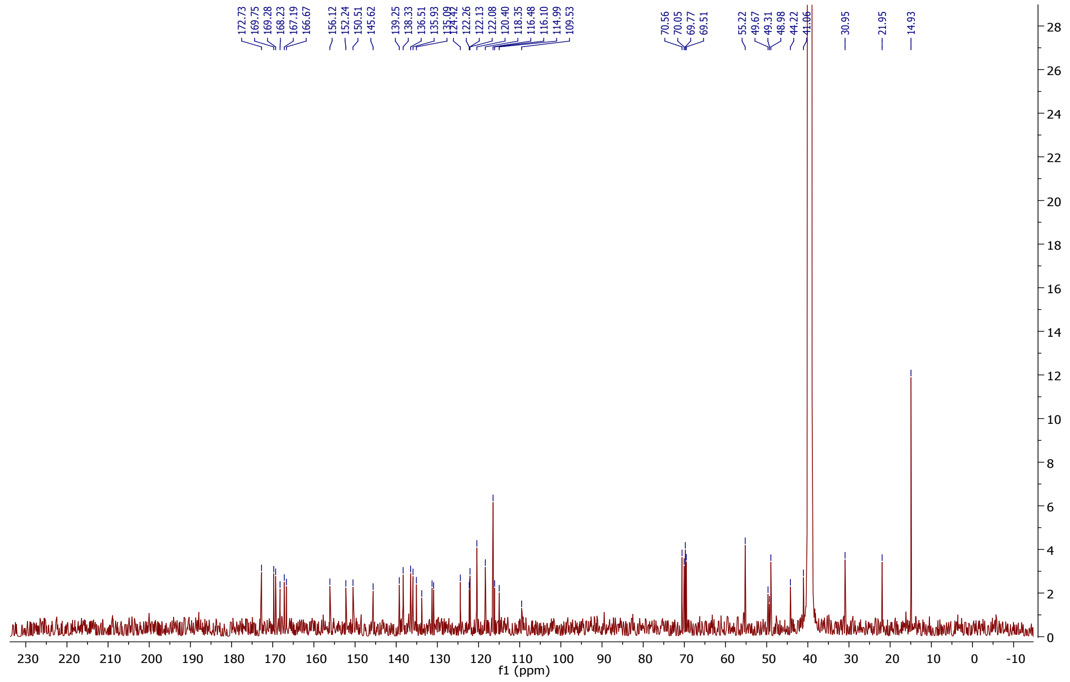

Figure S31

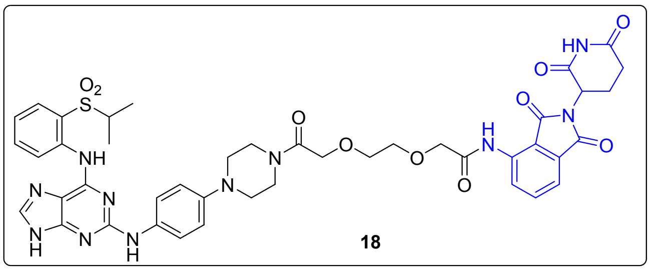

18

Analytical method 2

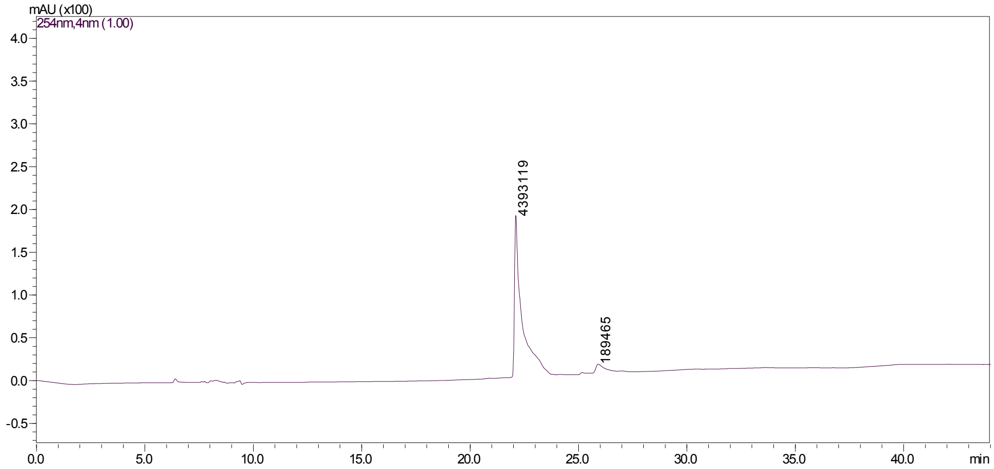

Area= 4393116

Total Area= 4582584

Purity (%)=95.9%

ESI-HRMS m/z for $C_{43}H_{46}N_{11}O_{10}S$ $[M+H]^+$ calcd 908.3144, found 908.3168.

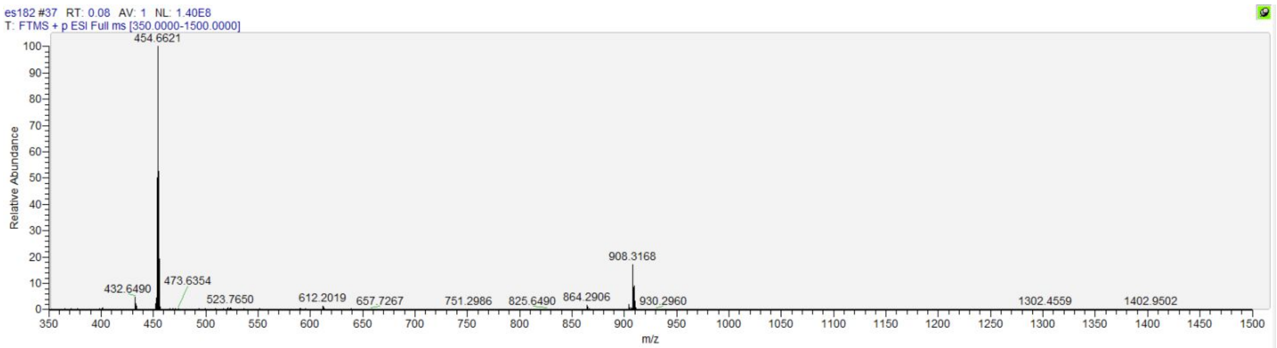

Figure S32

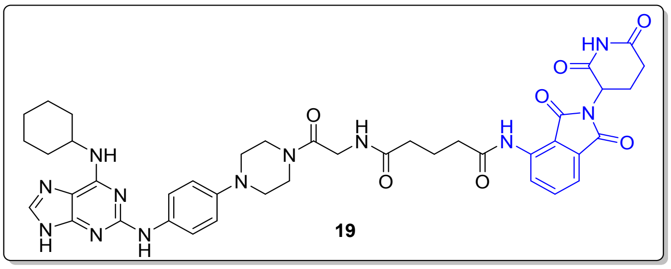

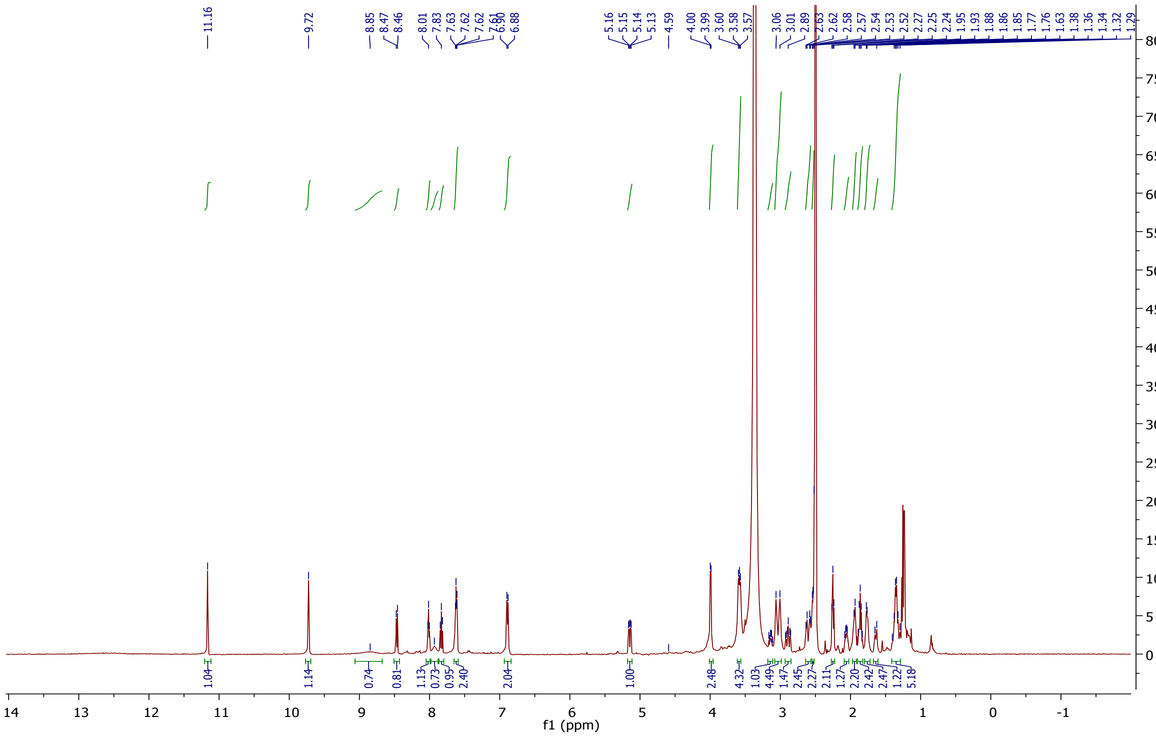

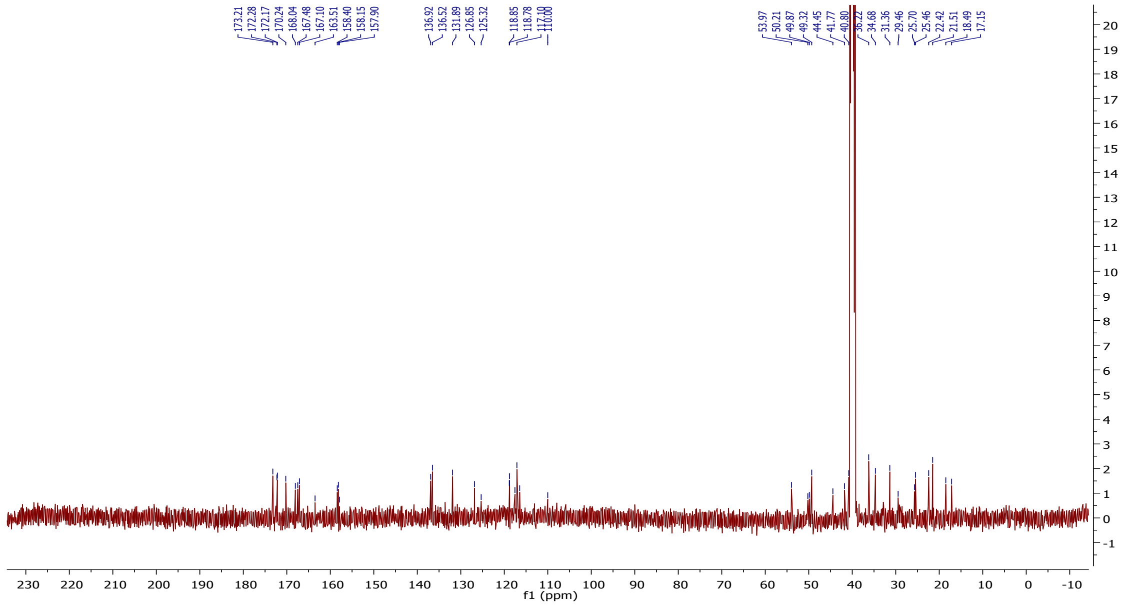

Figure S33

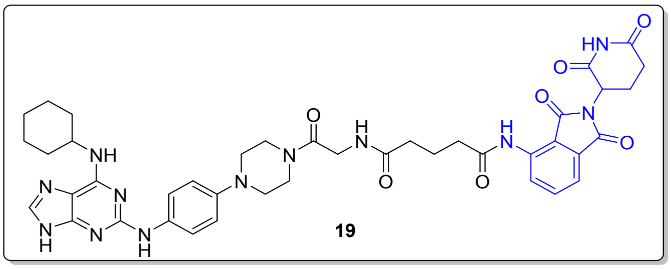

Analytical method 2

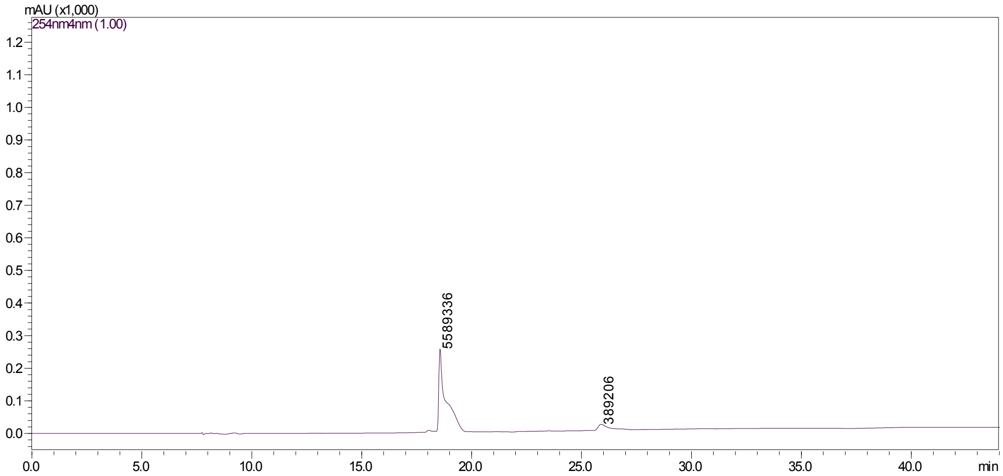

Area= 5589336

Total Area=5978542

Purity (%)=95%

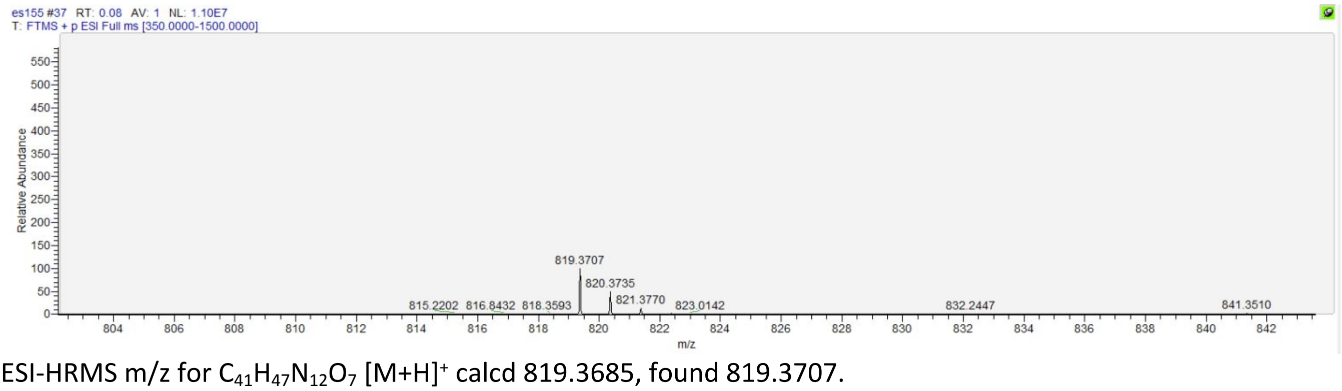

ESI-HRMS m/z for $C_{41}H_{47}N_{12}O_7$ $[M+H]^+$ calcd 819.3685, found 819.3707.

Figure S34

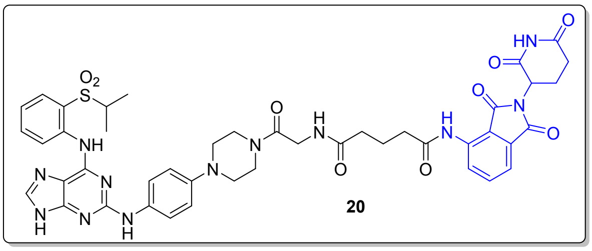
20

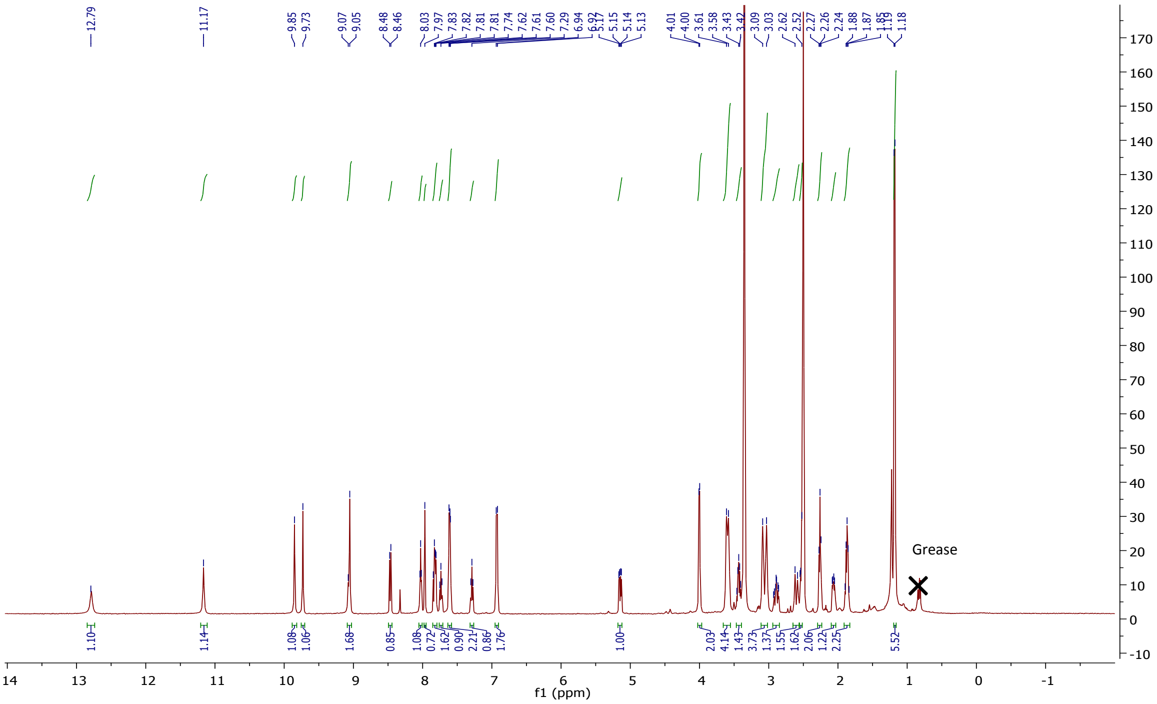
12.79
11.17
9.85
9.73
9.07
9.05
8.48
8.46
8.03
7.97
7.83
7.82
7.81
7.81
7.74
7.62
7.61
7.60
7.29
6.94
6.92
5.17
5.15
5.14
5.13
4.01
4.00
3.61
3.58
3.43
3.42
3.09
3.03
2.62
2.52
2.27
2.26
2.24
1.88
1.87
1.85
1.19
1.18
Grease
1.10
1.14
1.08
1.06
1.68
0.85
1.08
0.72
1.62
0.90
2.21
0.86
1.76
1.00
2.03
4.14
1.43
3.73
1.37
1.55
1.62
2.06
1.22
2.25
5.52
f1 (ppm)

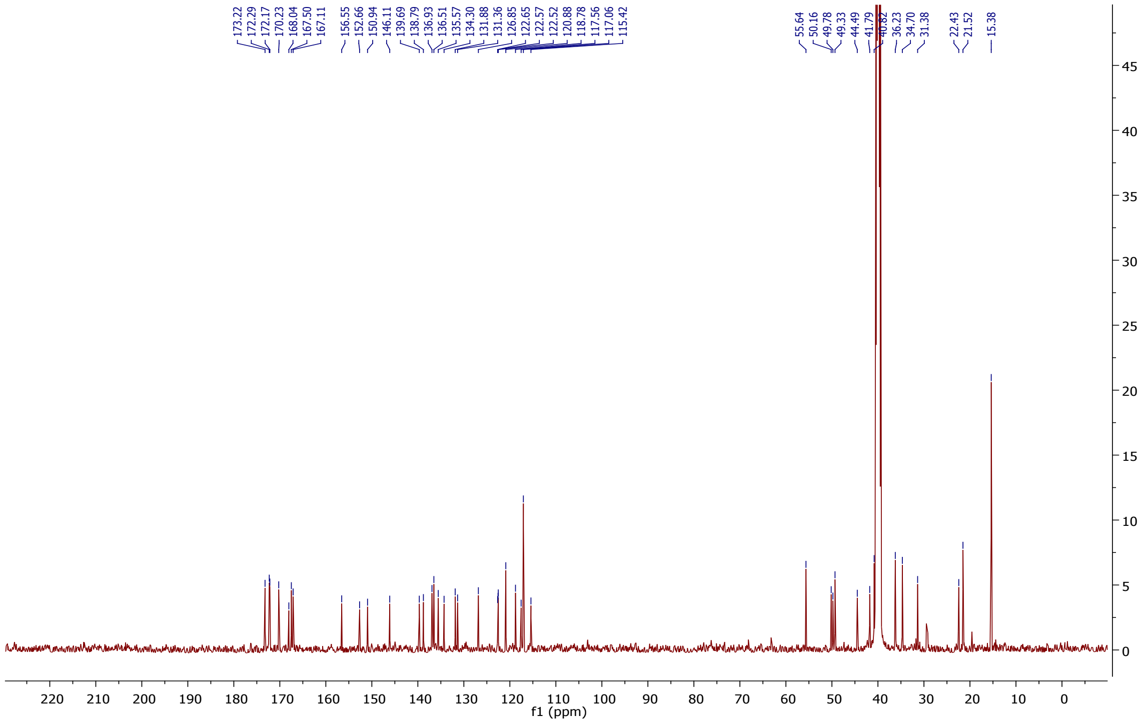
173.22
172.29
172.17
170.23
168.04
167.50
167.11
156.55
152.66
150.94
146.11
139.69
138.79
136.93
136.51
135.57
134.30
131.88
131.36
126.85
122.65
122.57
122.52
120.88
118.78
117.56
117.06
115.42
55.64
50.16
49.78
49.33
44.49
41.79
40.82
36.23
34.70
31.38
22.43
21.52
15.38
f1 (ppm)

Figure S35

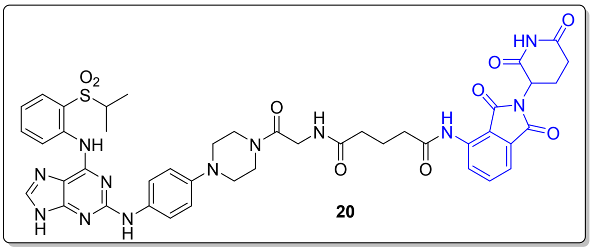

Analytical method 2

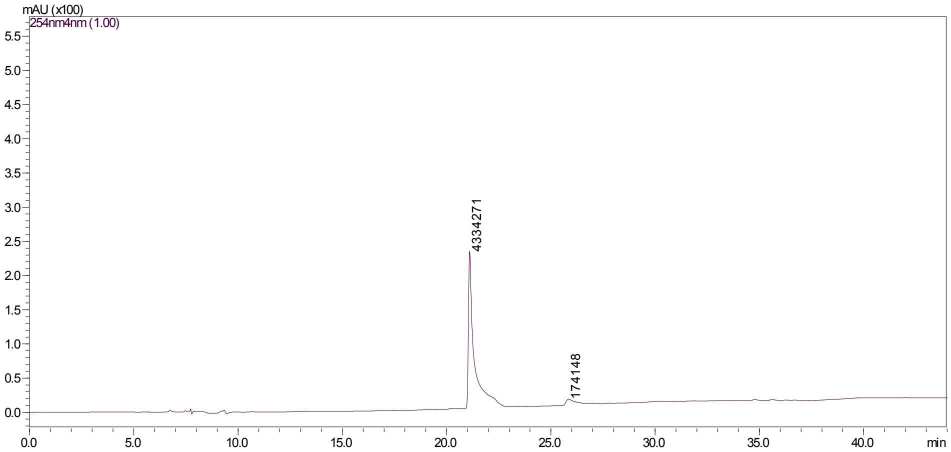

Area= 4334271

Total Area= 4508419

(%)=96.1%

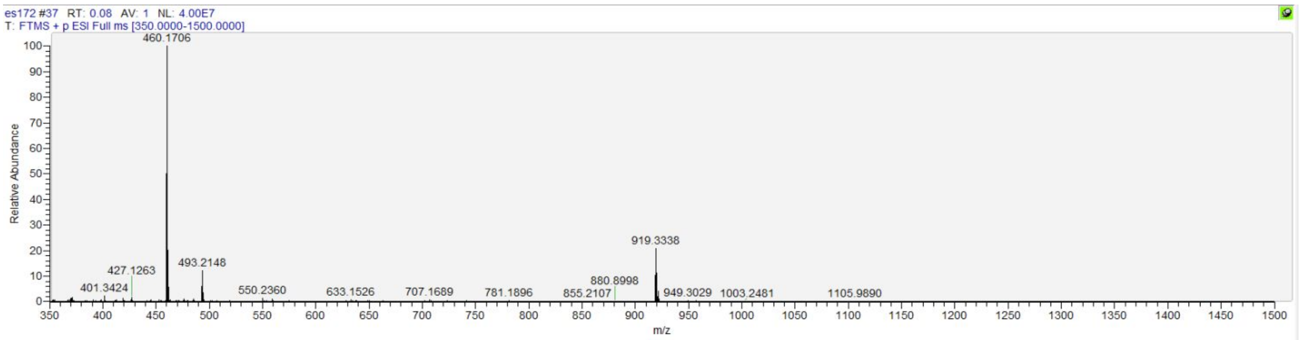

ESI-HRMS m/z for $C_{44}H_{47}N_{12}O_9S$ $[M+H]^+$ calcd 919.3304, found 919.3338.

Figure S36

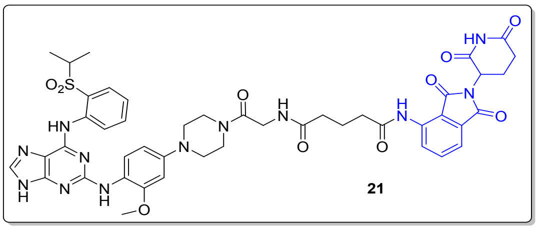

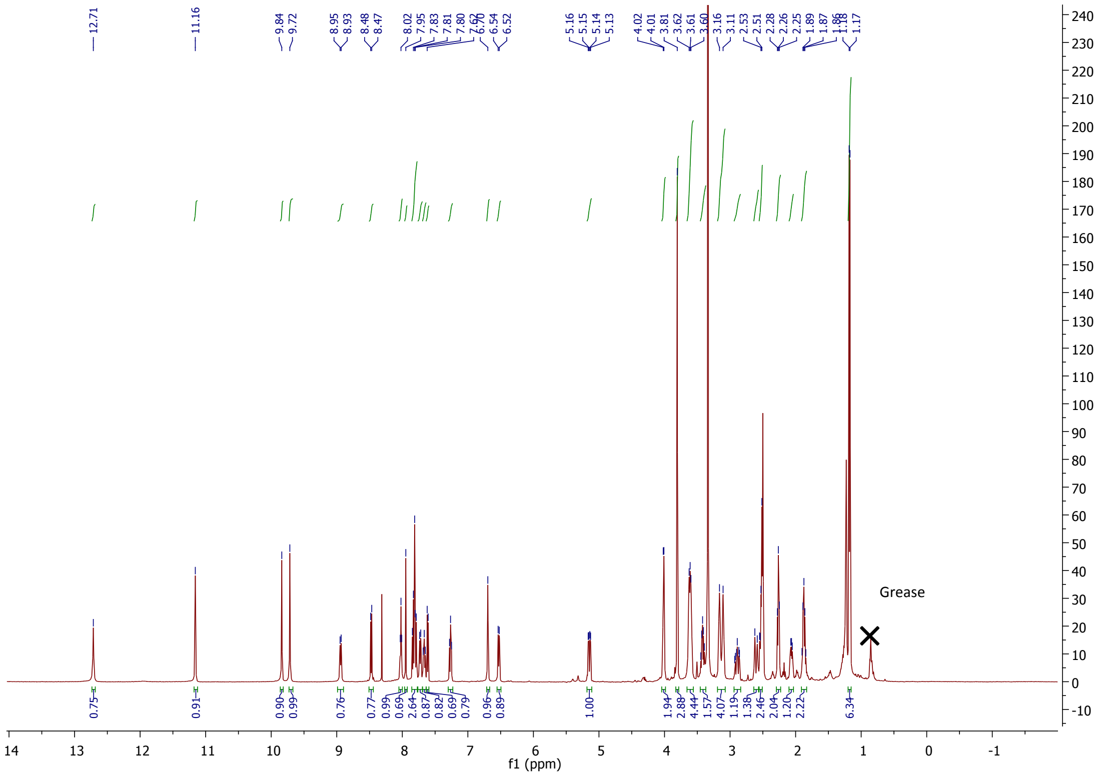

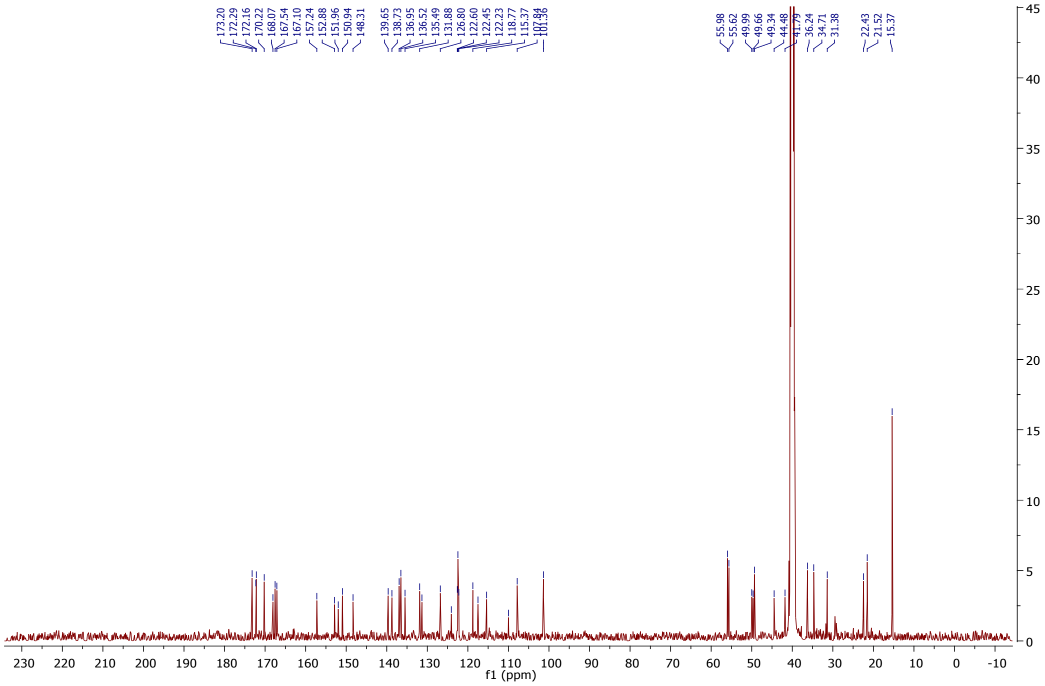

Figure S37

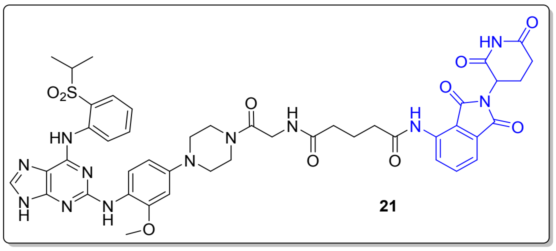

21

Analytical method 1

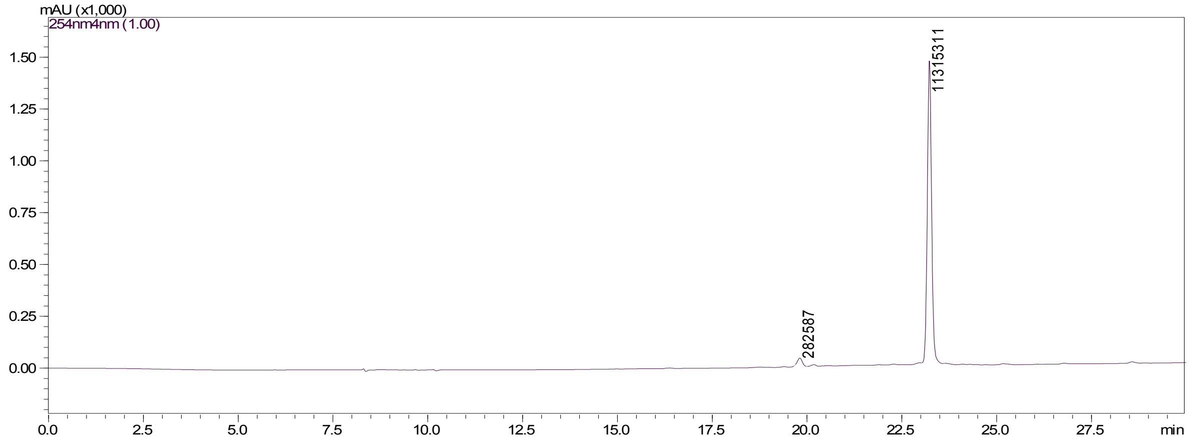

Total area: 11597898

Area: 11315311

Purity (%)=97.6%

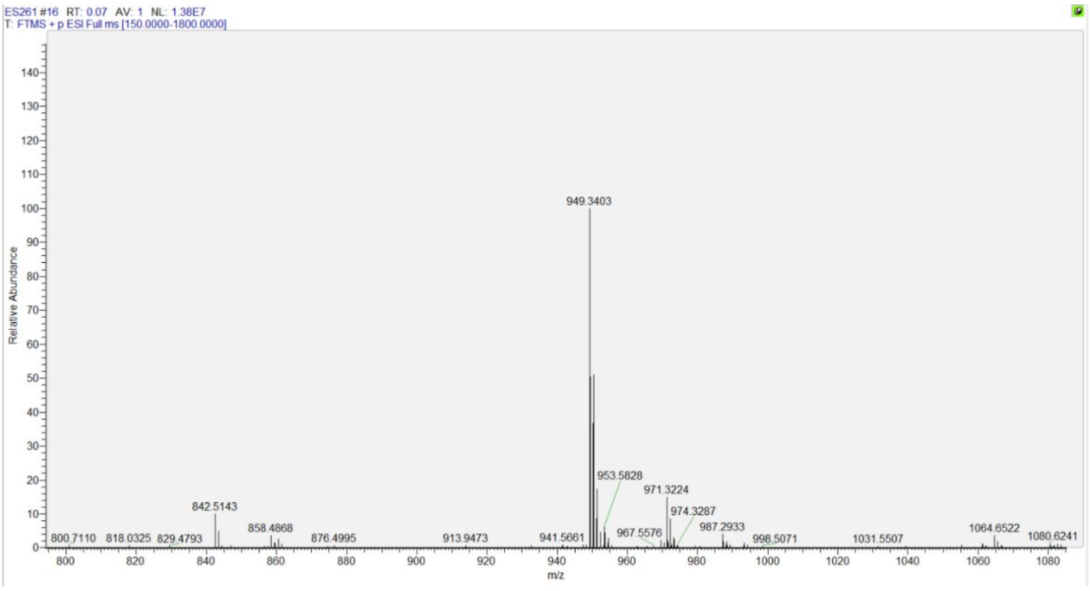

ESI-HRMS m/z for $C_{45}H_{49}N_{12}O_{10}S$ $[M+H]^+$ calcd 949.3415, found 949.3403.

Figure S38

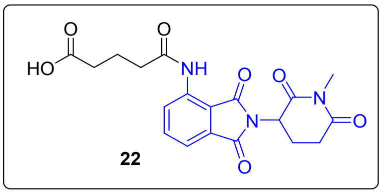
HO
O
O
NH
O
O
N
N
O
O
22

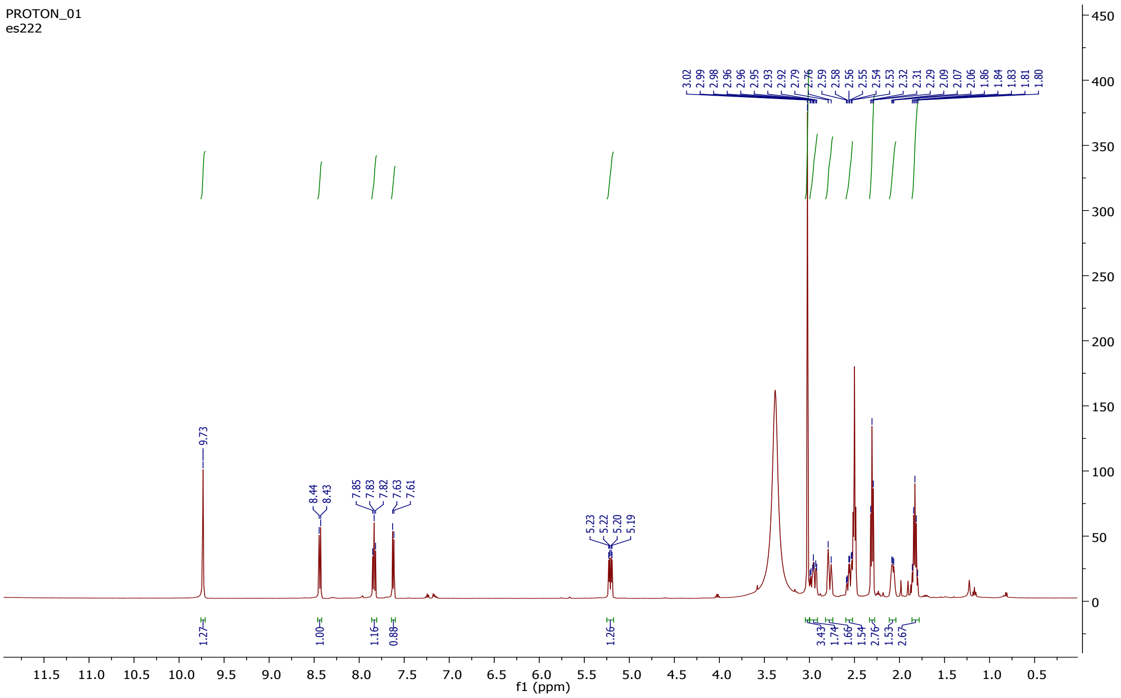
PROTON_01
es222
9.73
8.44
8.43
7.85
7.83
7.82
7.63
7.61
5.23
5.22
5.20
5.19
3.02
2.99
2.98
2.96
2.96
2.95
2.93
2.93
2.92
2.79
2.76
2.59
2.58
2.56
2.55
2.54
2.53
2.32
2.31
2.29
2.09
2.07
2.06
1.86
1.84
1.83
1.81
1.80
1.27
1.00
1.16
0.88
1.26
3.43
1.74
1.66
1.54
2.76
1.53
2.67
f1 (ppm)

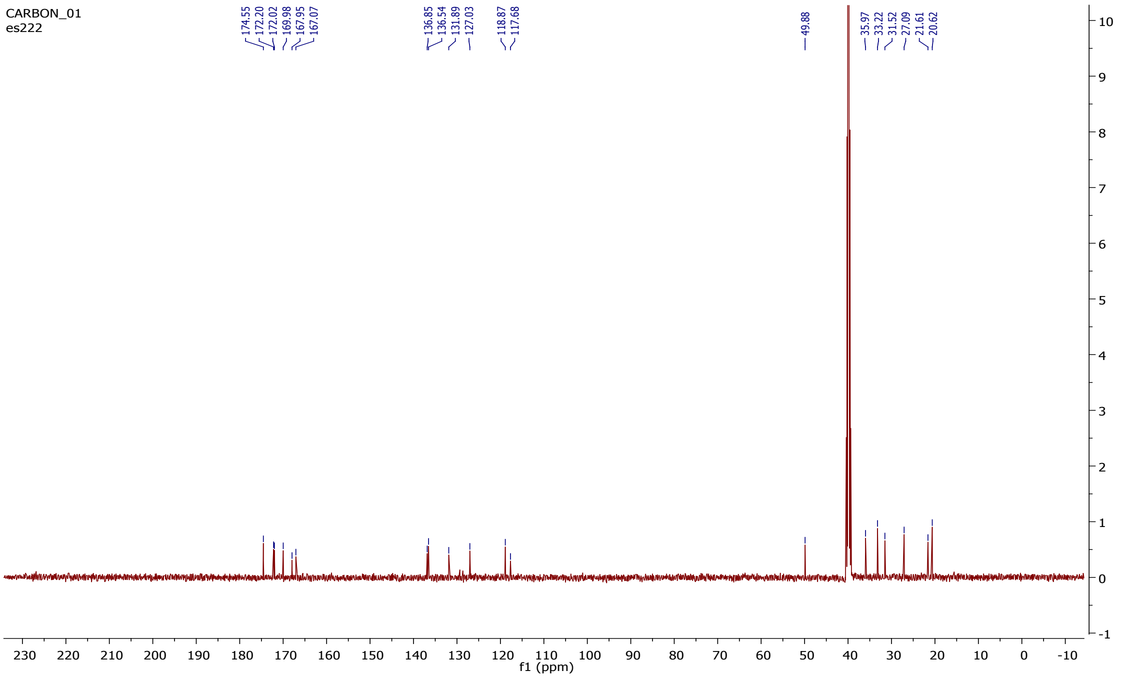
CARBON_01
es222
174.55
172.20
172.02
169.98
167.95
167.07
136.85
136.54
131.89
127.03
118.87
117.68
49.88
35.97
33.22
31.52
27.09
21.61
20.62
f1 (ppm)

Figure S39

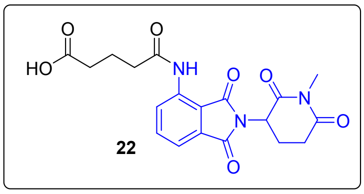

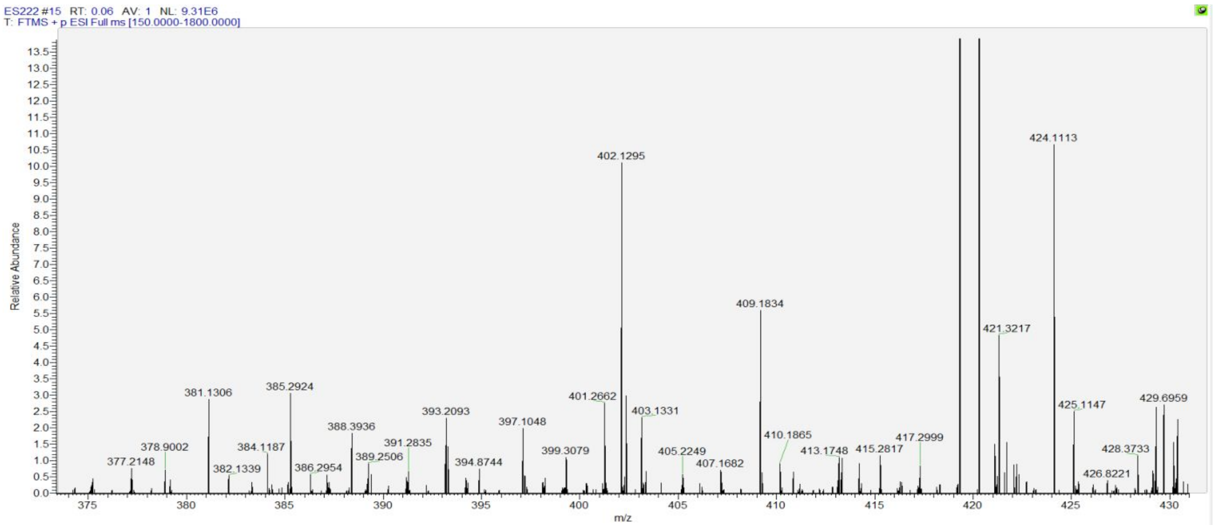

ESI-HRMS m/z for $C_{19}H_{20}N_3O_7$ $[M+H]^+$ calcd 402.1301, found 402.1295.

Figure S40

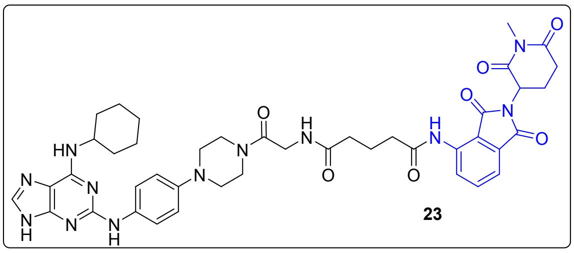

23

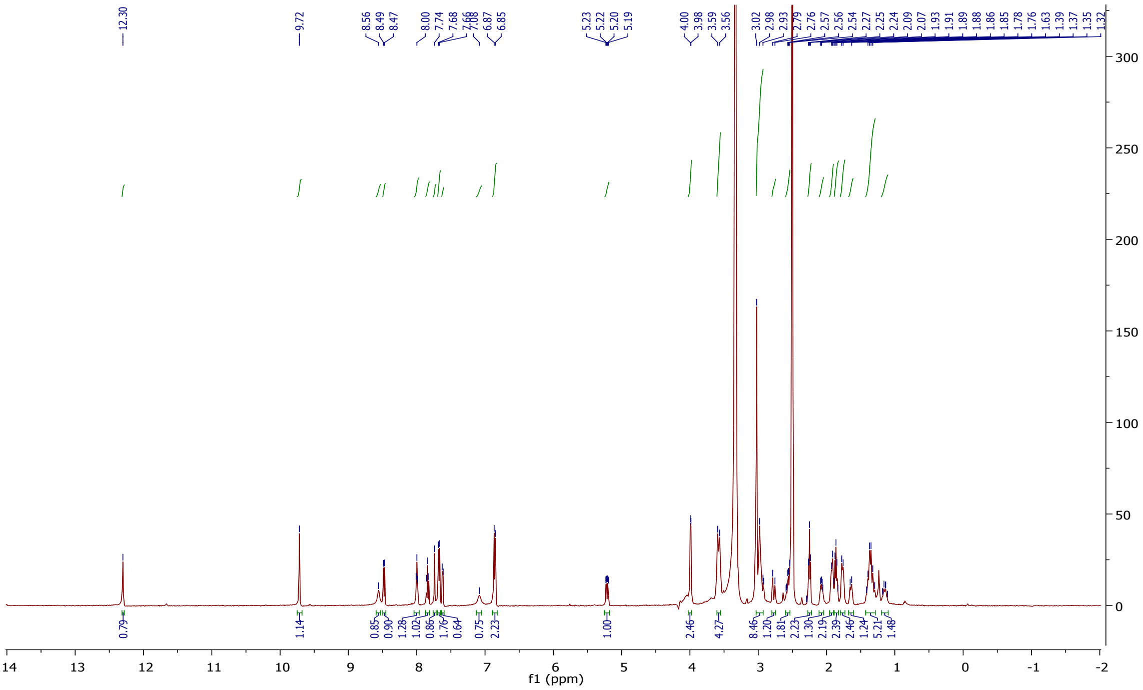

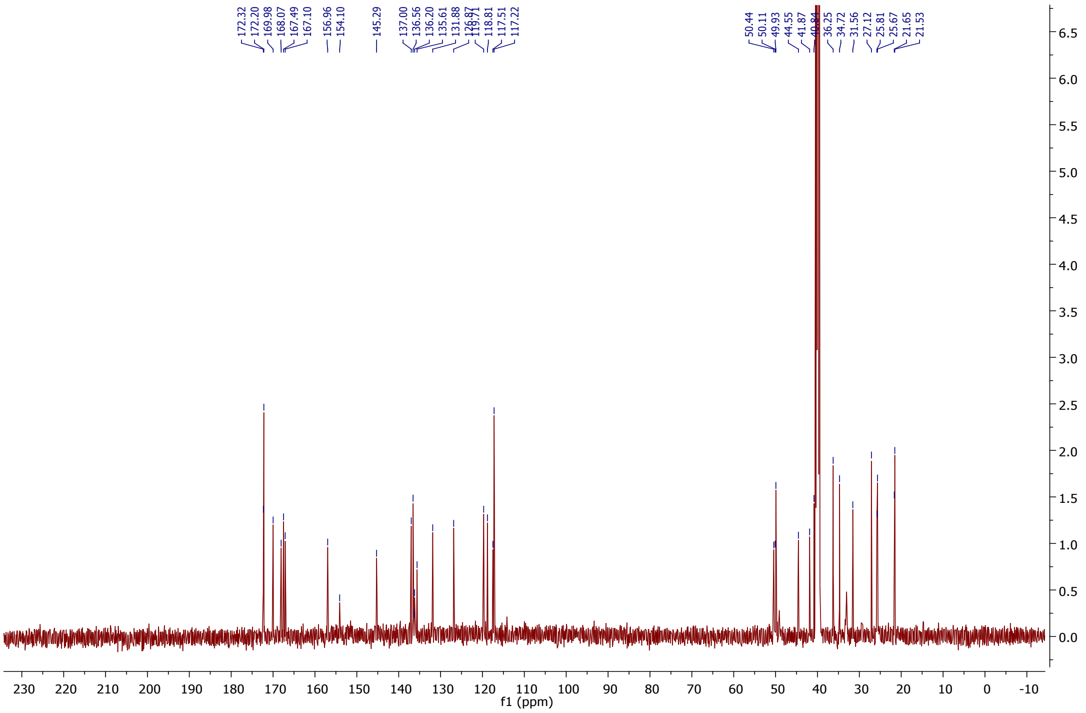

Figure S41

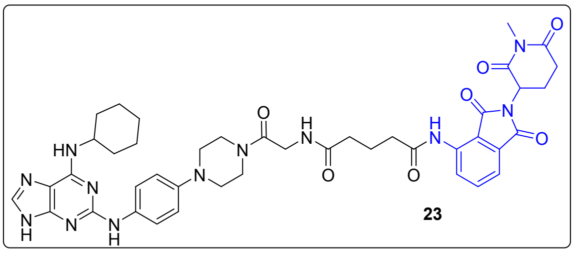

23

Analytical method 1

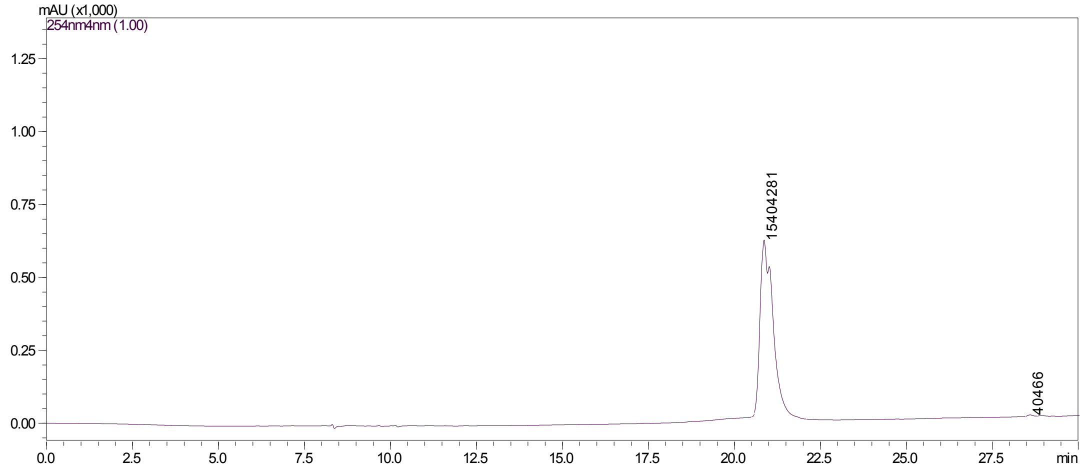

Area: 15404281

Total area: 15444747

Purity (%)=99.7%

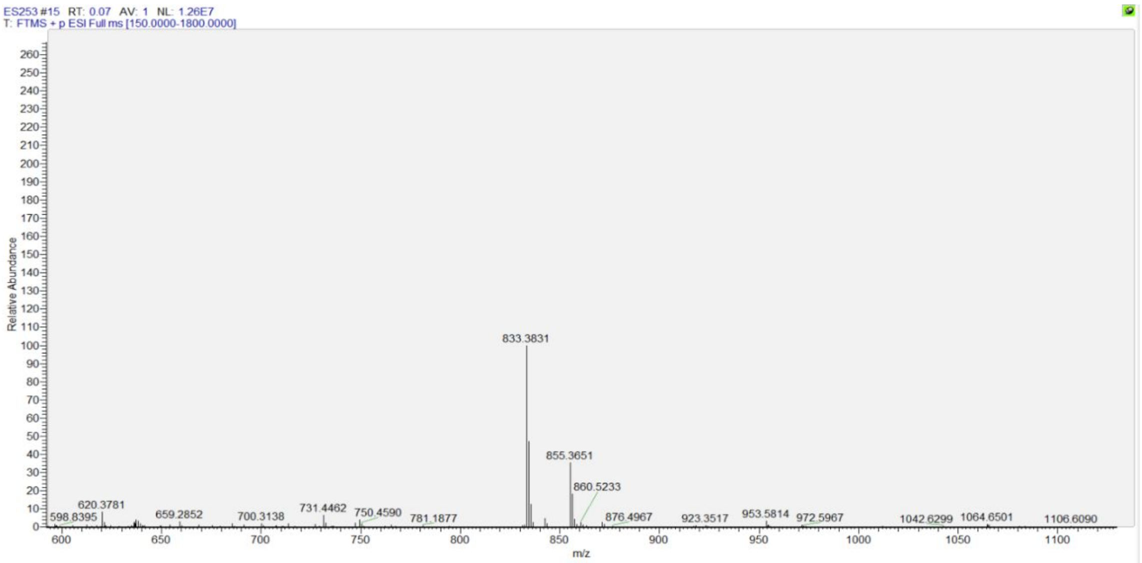

ESI-HRMS m/z for $C_{42}H_{49}N_{12}O_7$ $[M+H]^+$ calcd 833.3847, found 833.3831.

Figure S42

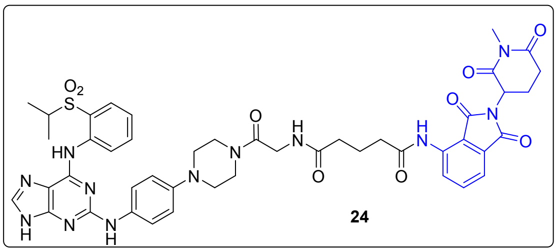

24

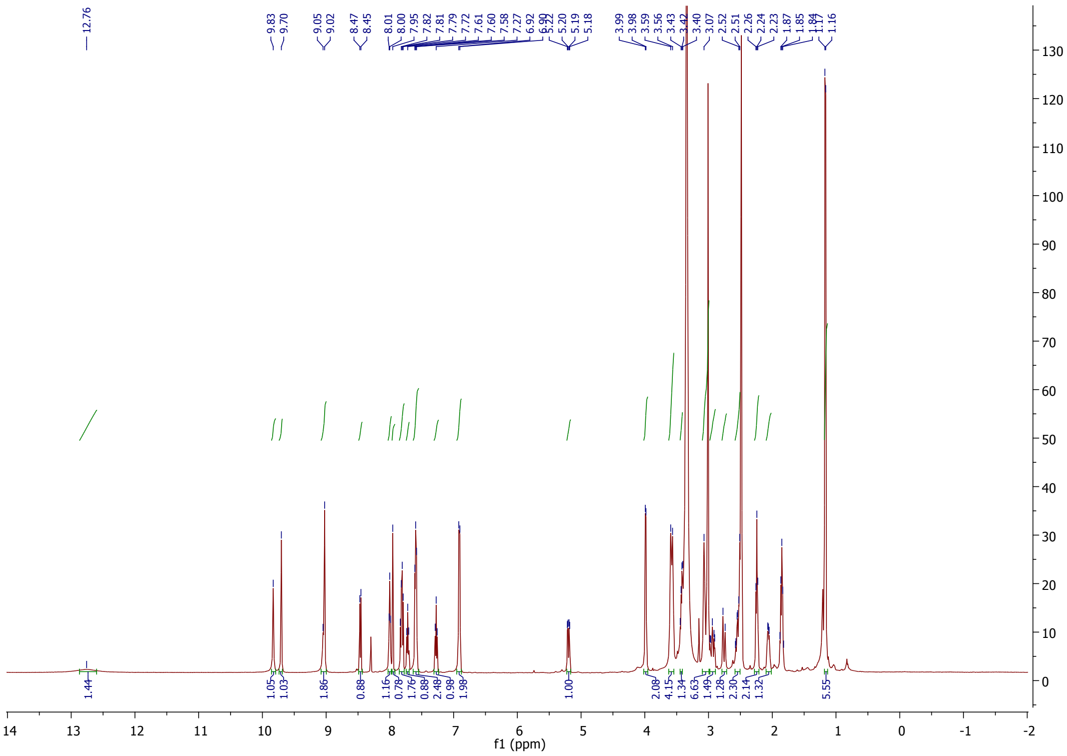

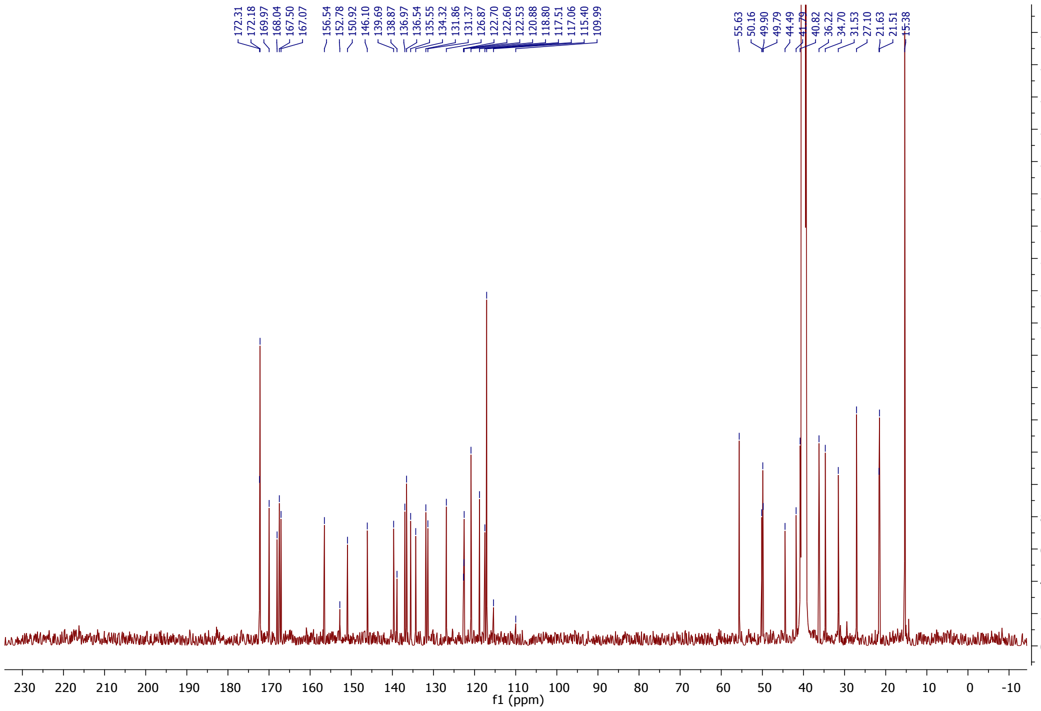

Figure S43

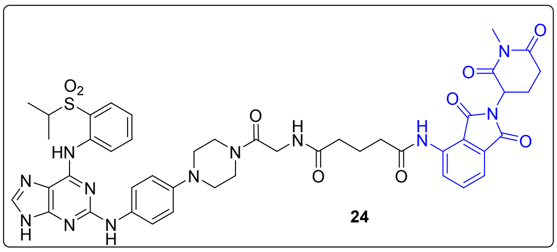

24

Analytical method 2

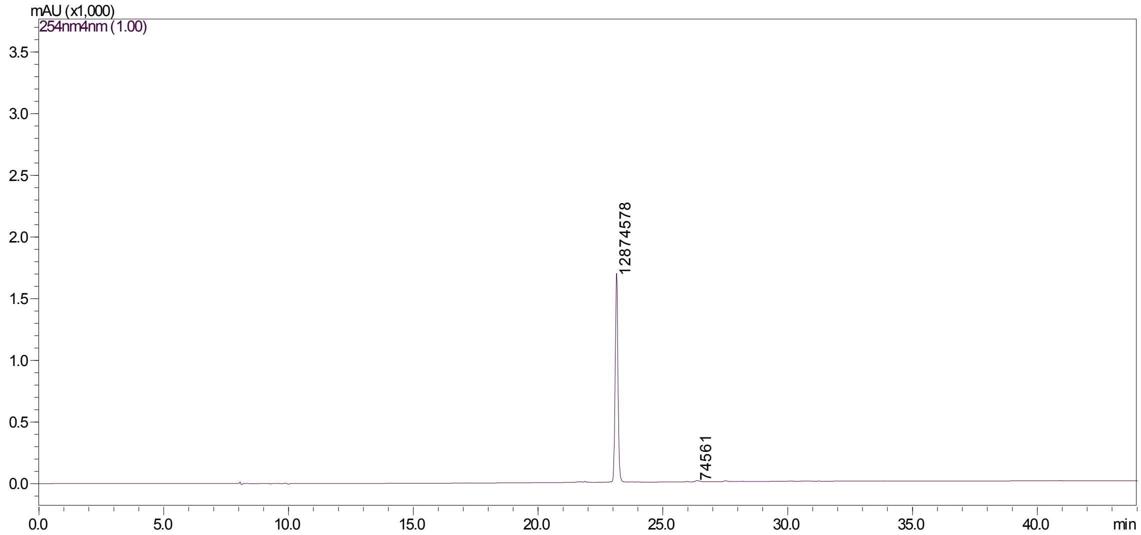

Area= 12893769

Total Area= 13001563

Purity (%)= 99.1%

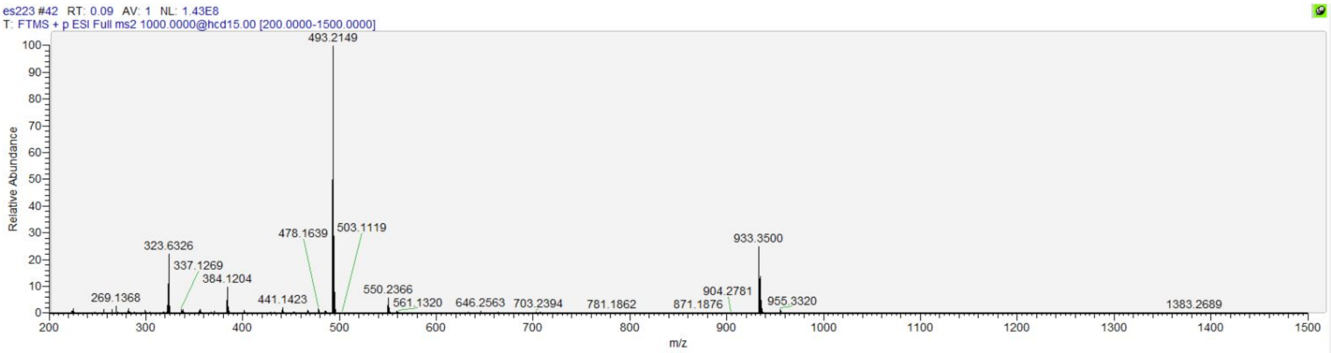

ESI-HRMS m/z for $C_{45}H_{49}N_{12}O_9S$ $[M+H]^+$ calcd 933.3461, found 933.3500.

Figure S44

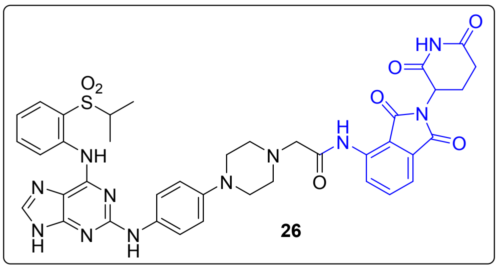
26

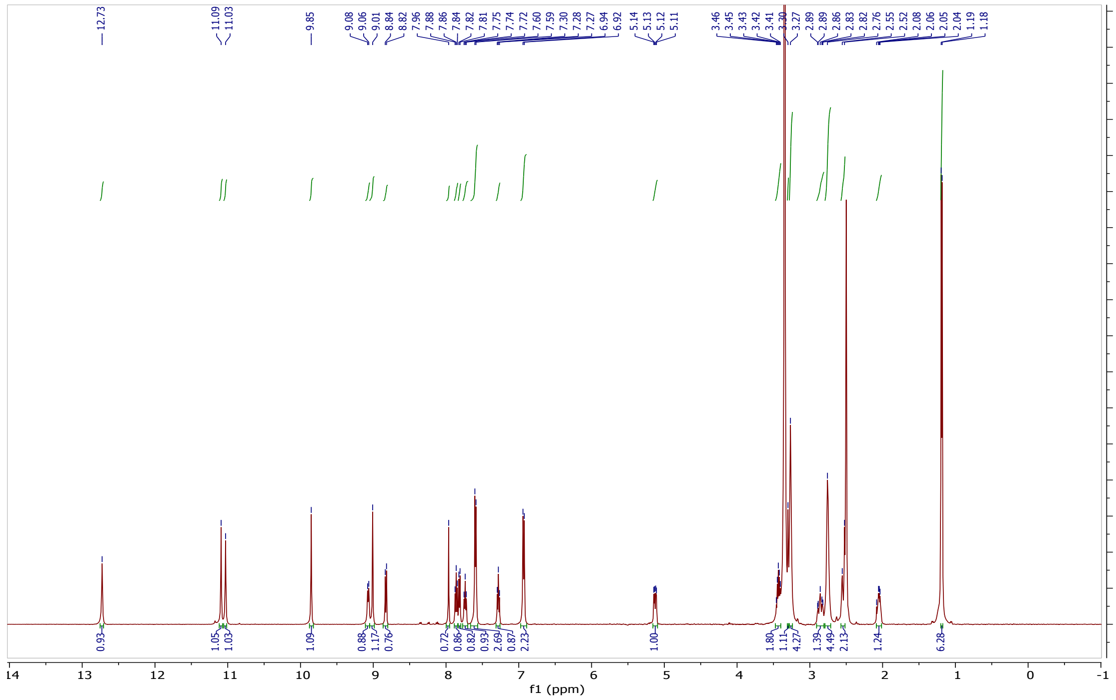
12.73
11.09
11.03
9.85
9.08
9.06
9.01
8.84
8.82
7.96
7.88
7.86
7.86
7.84
7.82
7.81
7.75
7.74
7.72
7.60
7.59
7.30
7.28
7.27
6.94
6.92
5.14
5.13
5.12
5.11
3.46
3.45
3.43
3.42
3.41
3.30
3.27
2.89
2.89
2.86
2.83
2.82
2.76
2.55
2.52
2.08
2.06
2.05
2.04
1.19
1.18
0.93
1.05
1.03
1.09
0.88
1.17
0.76
0.72
0.86
0.82
0.93
2.69
0.87
2.23
1.00
1.80
1.11
4.27
1.39
4.49
2.13
1.24
6.28
f1 (ppm)

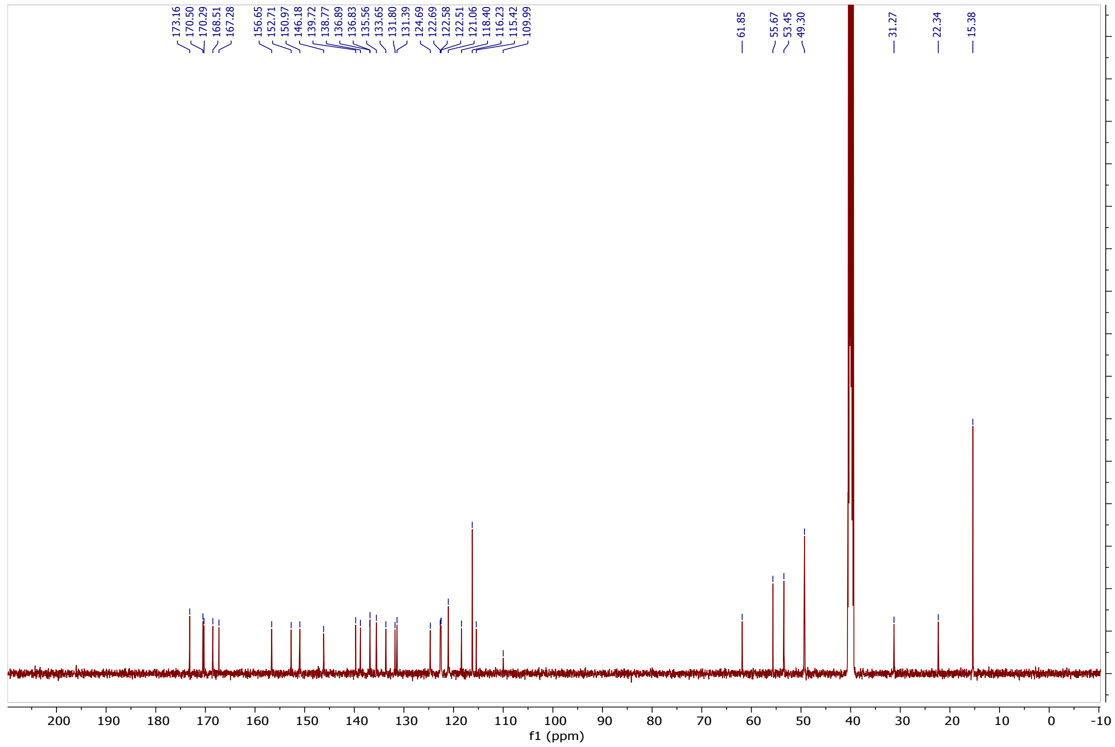
173.16
170.50
170.29
168.51
167.28
156.65
152.71
150.97
146.18
139.72
138.77
136.89
136.83
135.56
133.65
131.80
131.39
124.69
122.69
122.58
122.51
121.06
118.40
116.23
115.42
109.99
61.85
55.67
53.45
49.30
31.27
22.34
15.38
f1 (ppm)

Figure S45

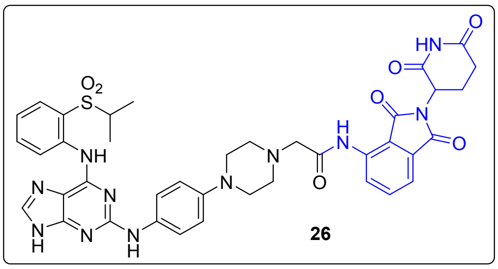

Analytical method 1

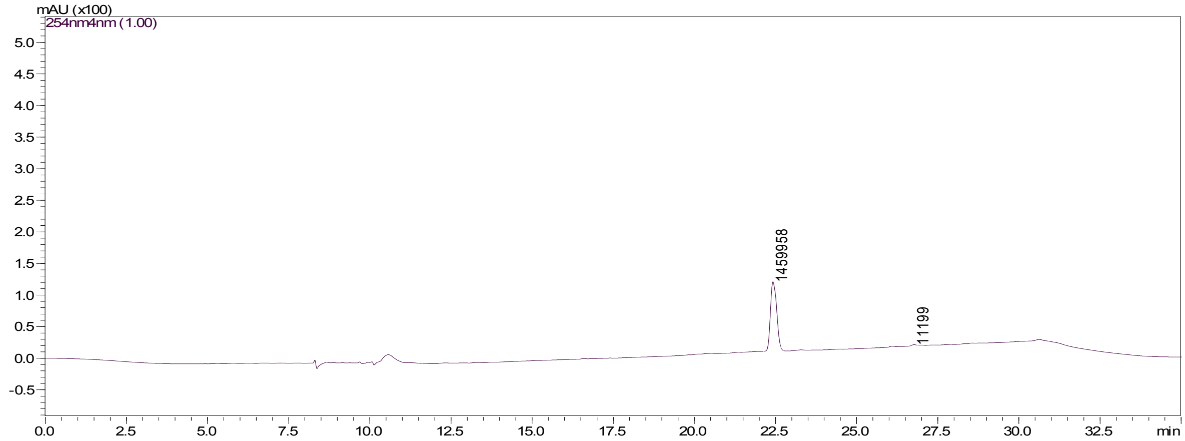

Area: 1459958

Total area: 1471157

Purity (%)=99.2%

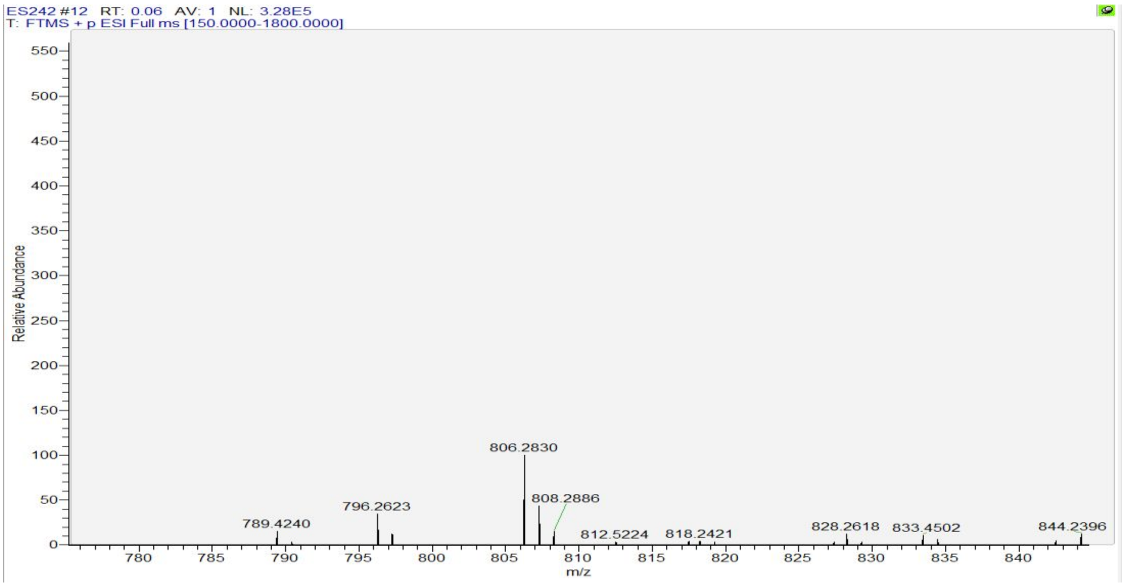

ESI-HRMS m/z for $C_{39}H_{40}N_{11}O_7S$ $[M+H]^+$ calcd 806.2833, found 806.2830.

Figure S46

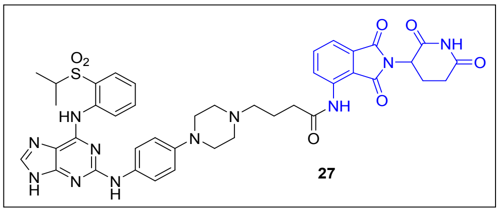

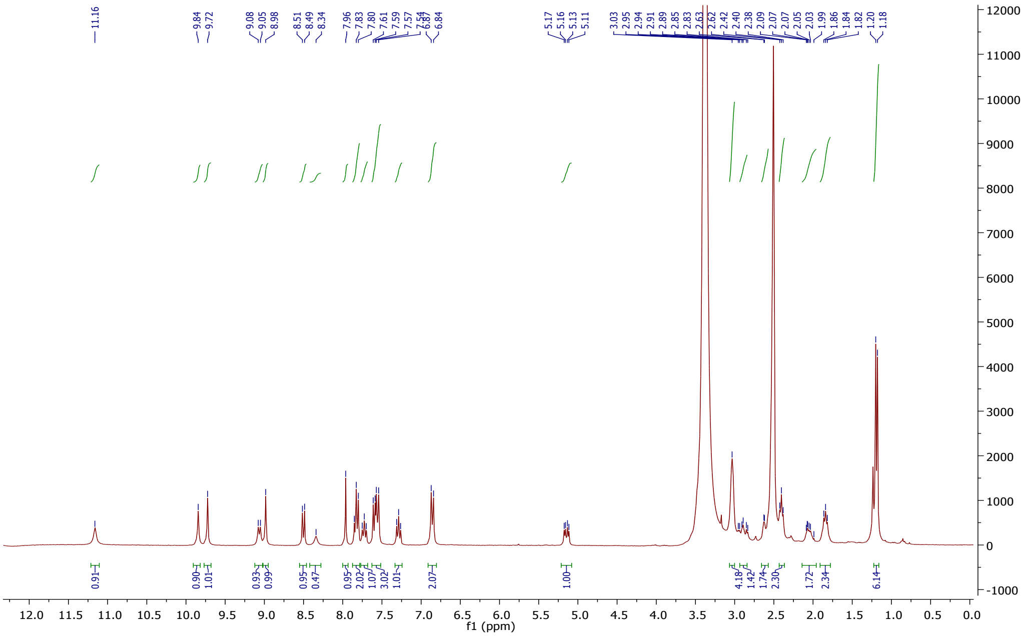

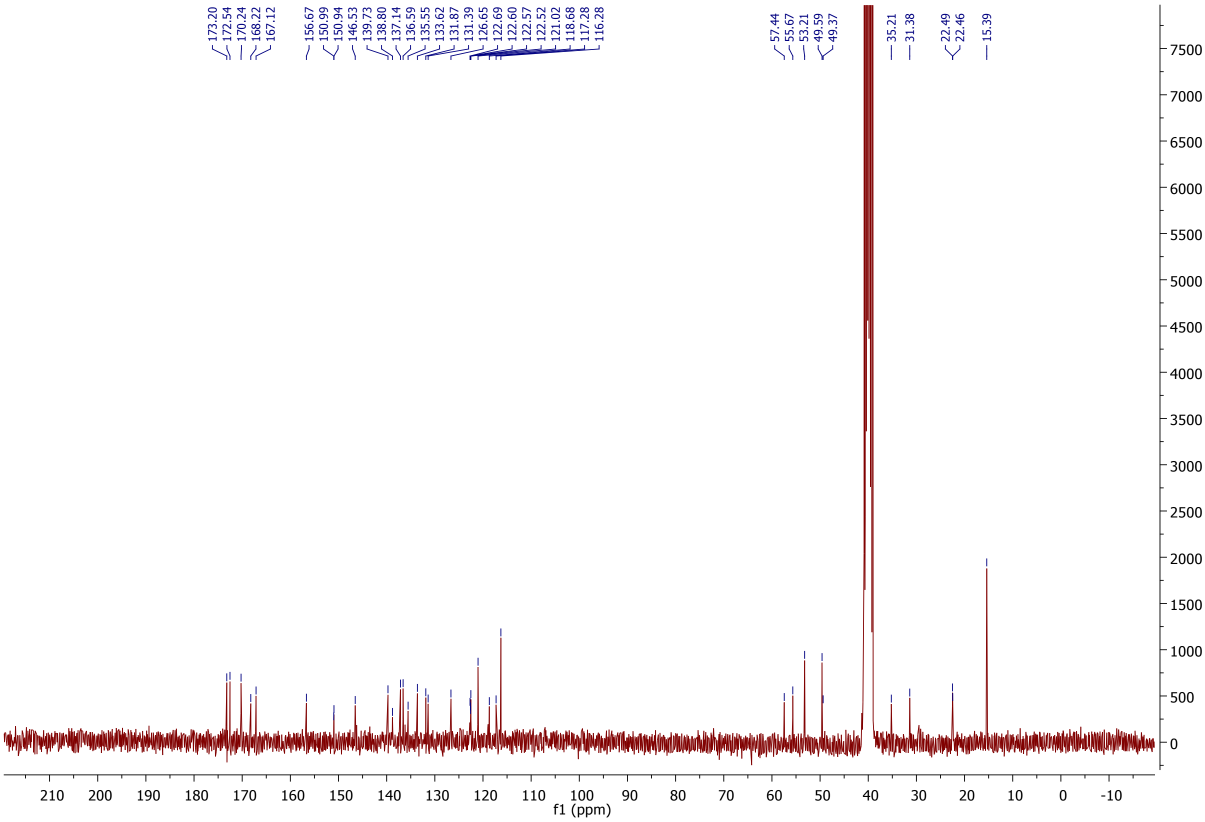

Figure S47

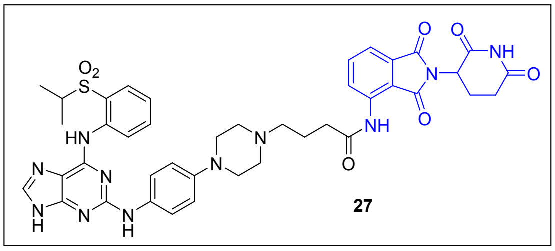

Analytical method 1

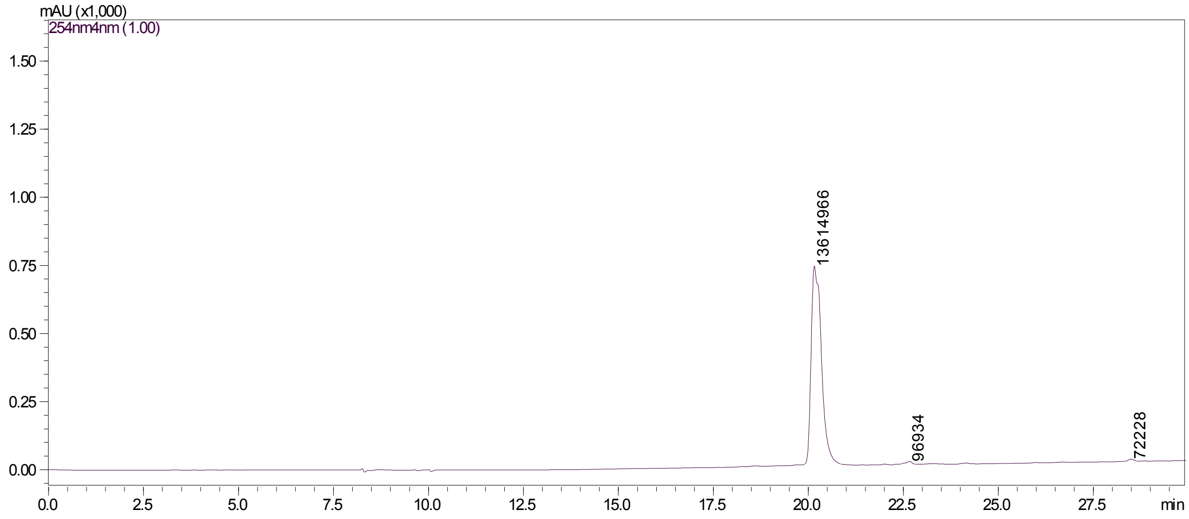

Area: 13614966

Total area: 13784128

Purity (%)=98.8%

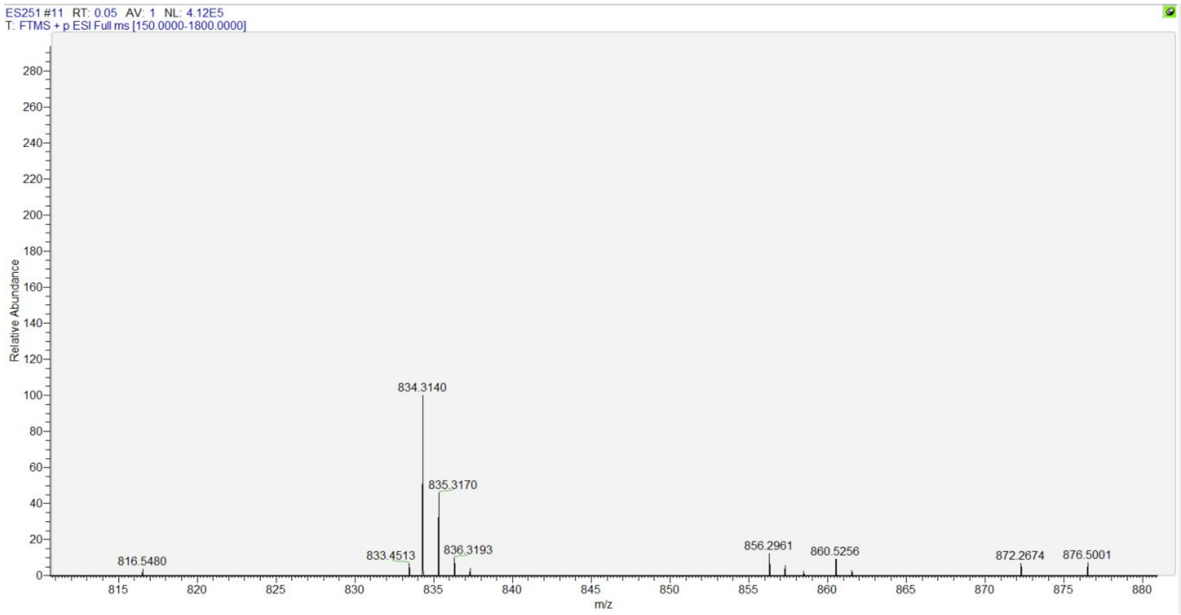

ESI-HRMS m/z for $C_{41}H_{44}N_{11}O_7S$ $[M+H]^+$ calcd 834.3146, found 834.3140.

Figure S48

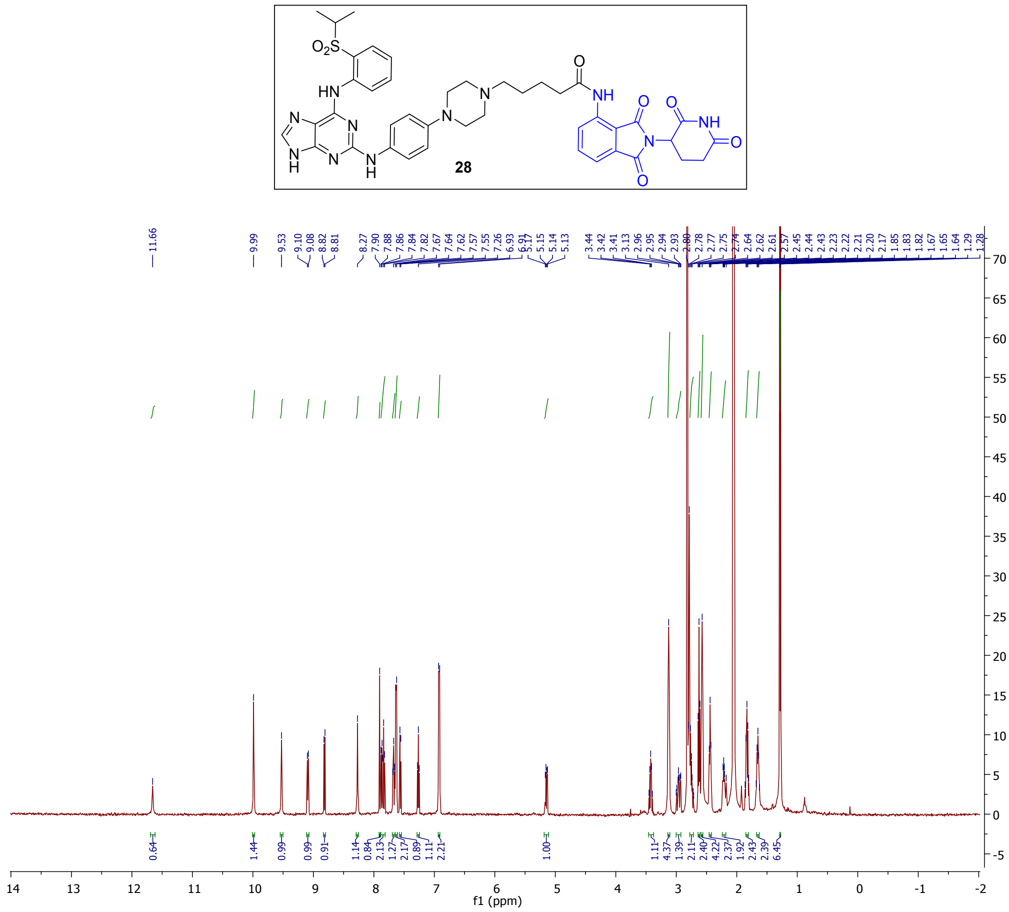

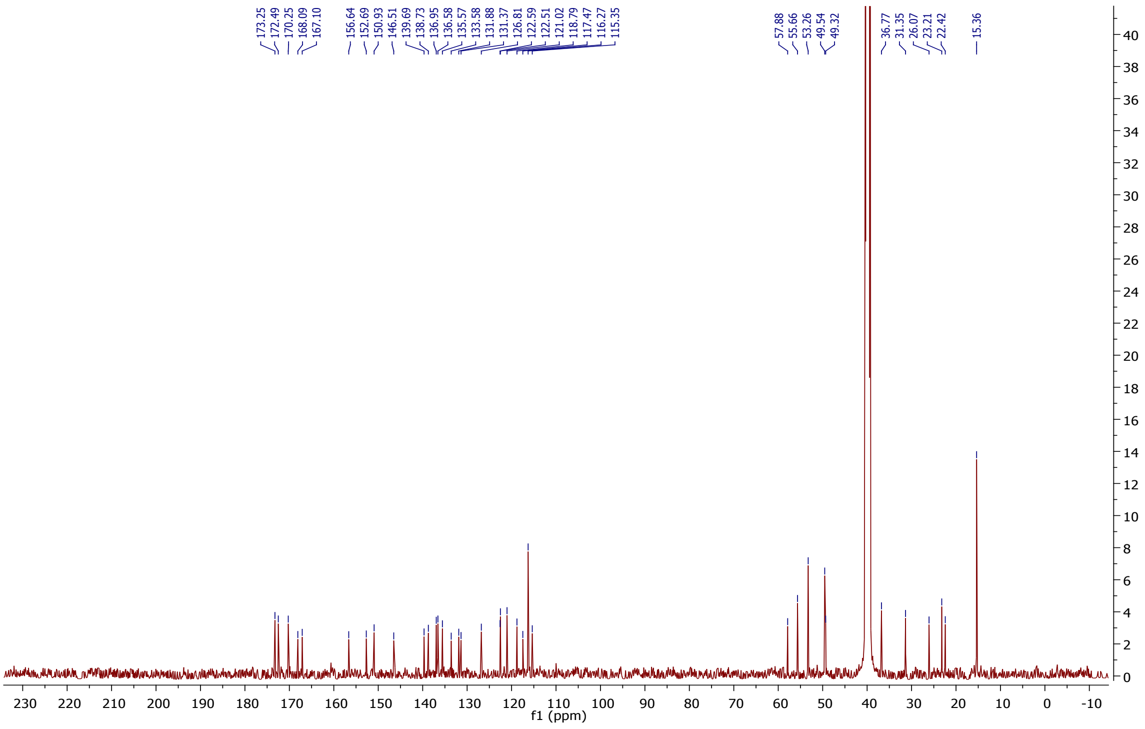

Figure S49

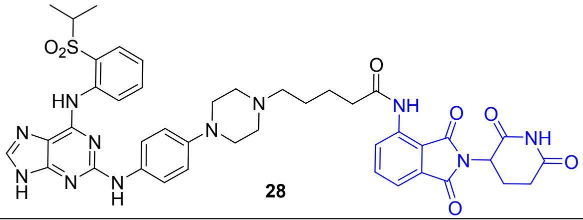

28

Analytical method 1

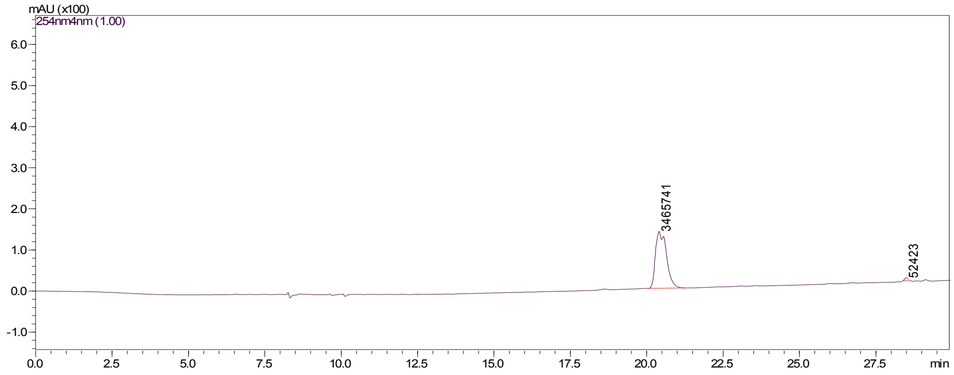

Area: 3465741

Total area: 3518164

Purity (%)=98.5%

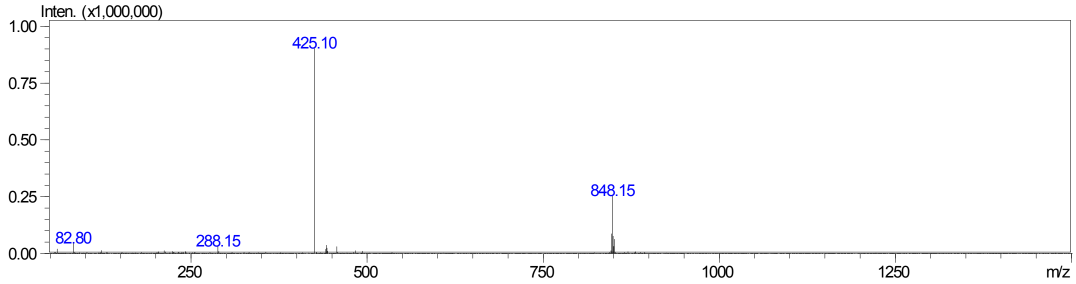

ESI-MS m/z for $C_{42}H_{46}N_{11}O_7S$ $[M+H]^+$ calcd 848.33, found 848.15.

Figure S50

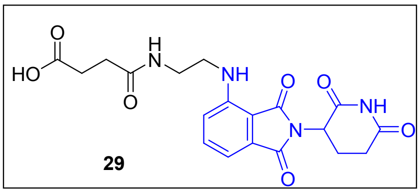

HO
O
O
H
N
NH
O
O
N
O
NH
O
O
29

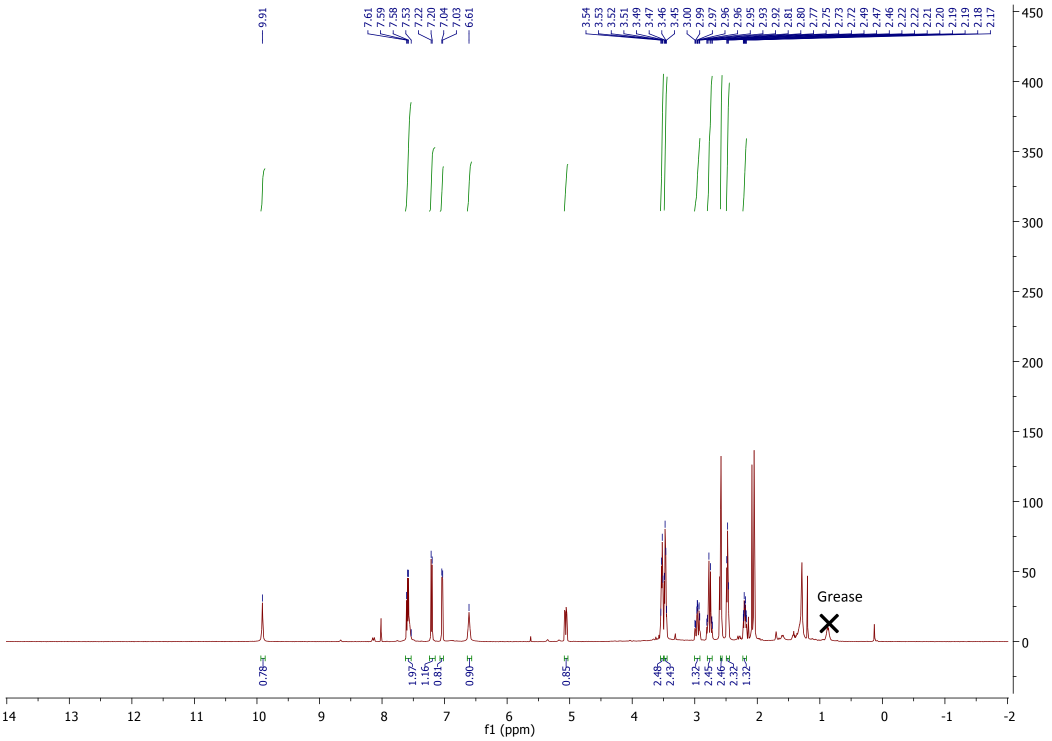

9.91
7.61
7.59
7.58
7.53
7.22
7.20
7.04
7.03
6.61
3.54
3.53
3.52
3.51
3.49
3.47
3.46
3.45
3.00
2.99
2.97
2.96
2.96
2.95
2.93
2.92
2.81
2.80
2.77
2.75
2.73
2.72
2.49
2.47
2.46
2.22
2.22
2.21
2.20
2.19
2.19
2.18
2.17
Grease
0.78
1.97
1.16
0.81
0.90
0.85
2.48
2.43
1.32
2.45
2.46
2.32
1.32
f1 (ppm)

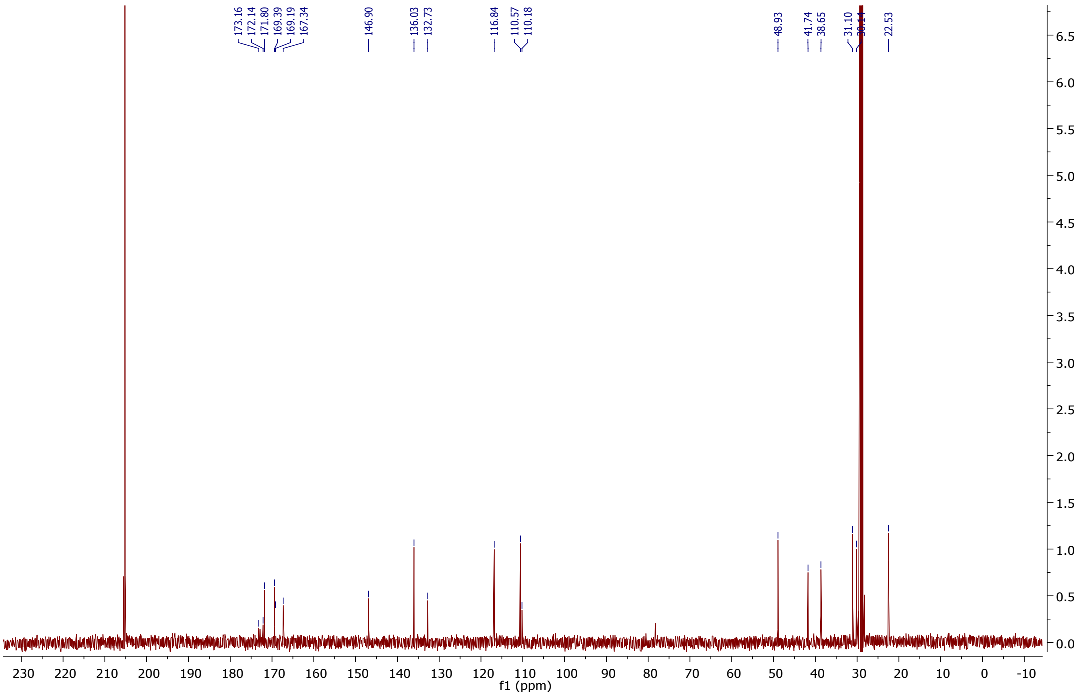

173.16
172.14
171.80
169.39
169.19
167.34
146.90
136.03
132.73
116.84
110.57
110.18
48.93
41.74
38.65
31.10
30.14
22.53
f1 (ppm)

Figure S51

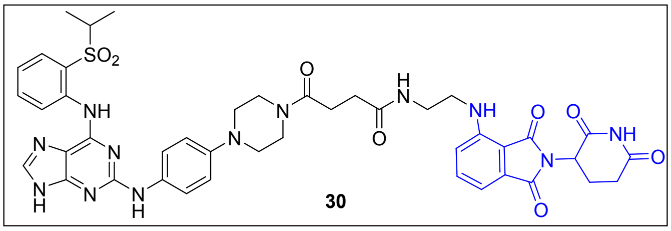

30

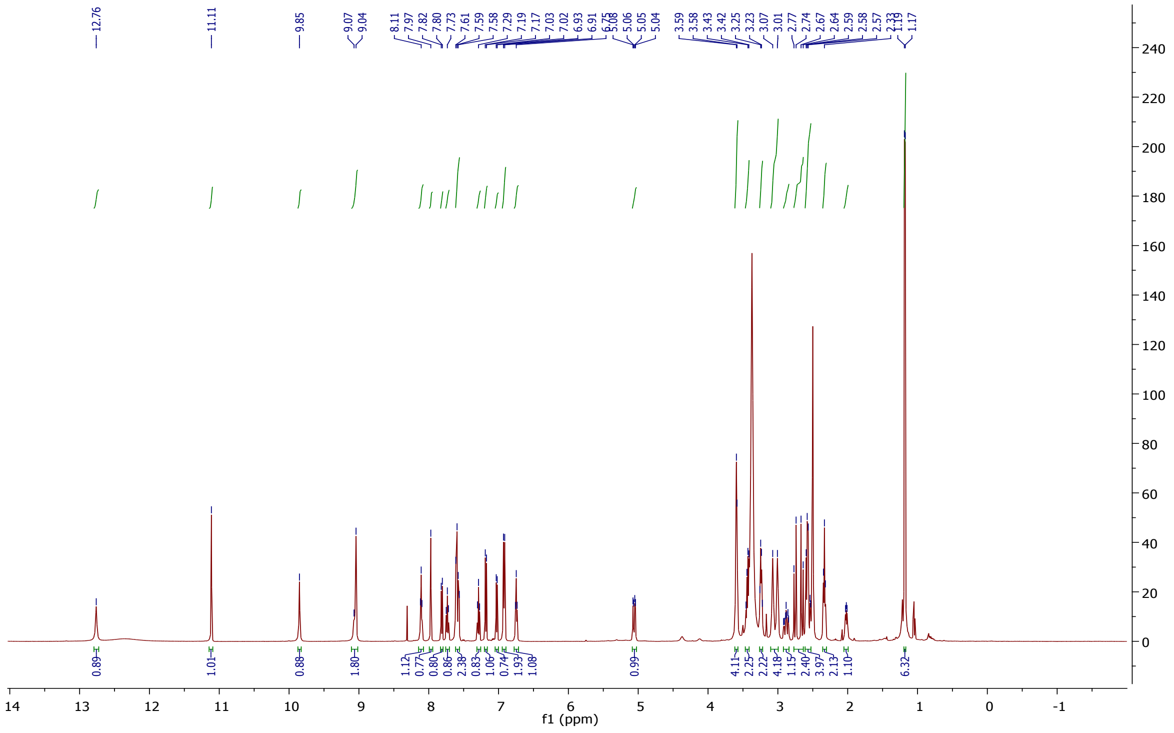

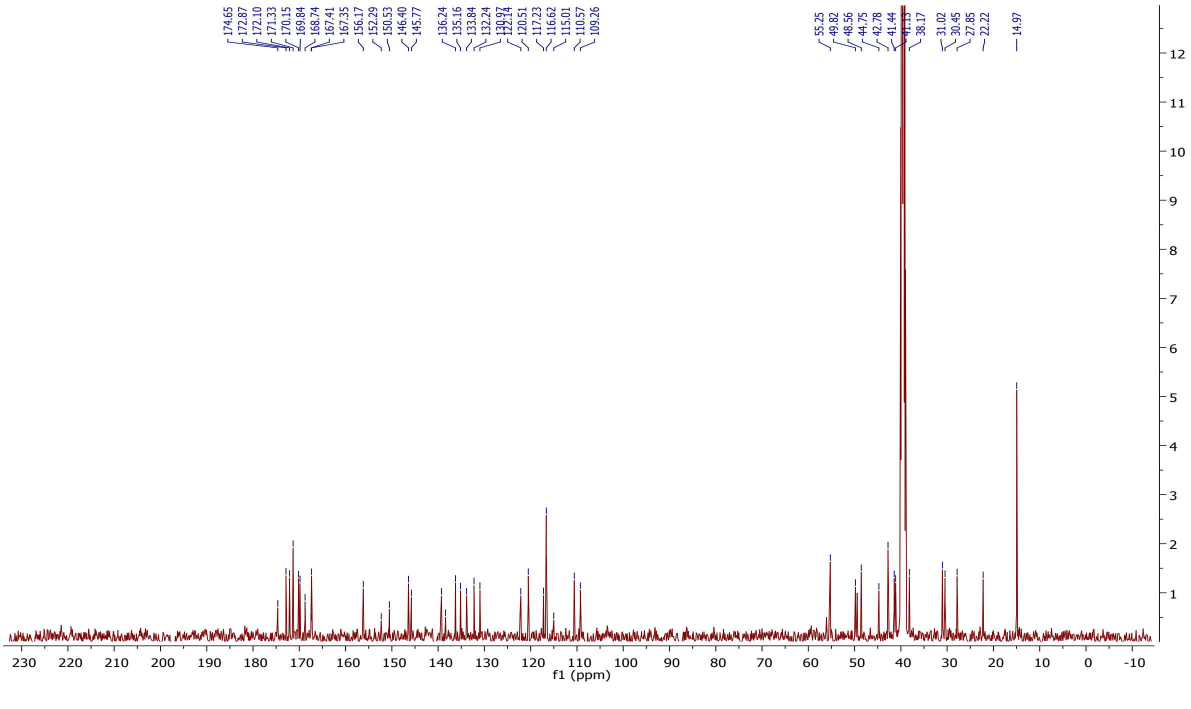

Figure S52

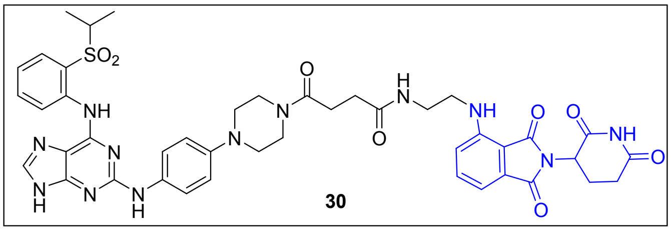

30

Analytical method 2

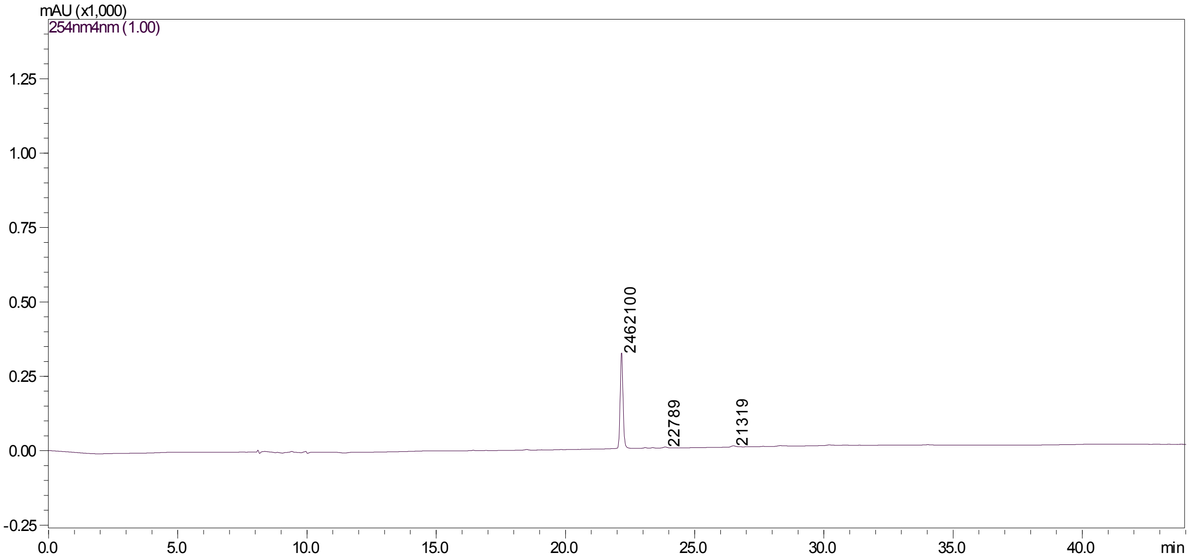

Area= 2462100

Total Area= 2506208

Purity (%)= 98,2%

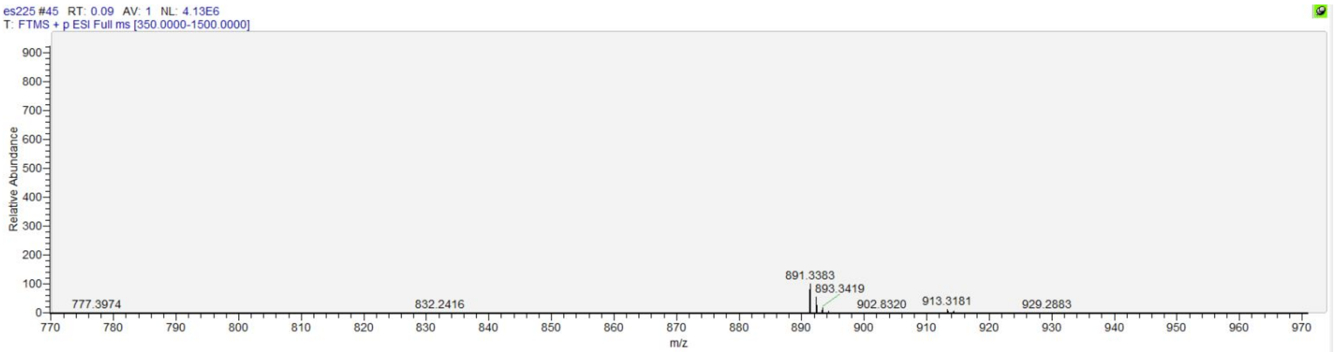

ESI-HRMS m/z for $C_{43}H_{47}N_{12}O_8S$ $[M+H]^+$ calcd 891.3355, found 891.3383.

Figure S53

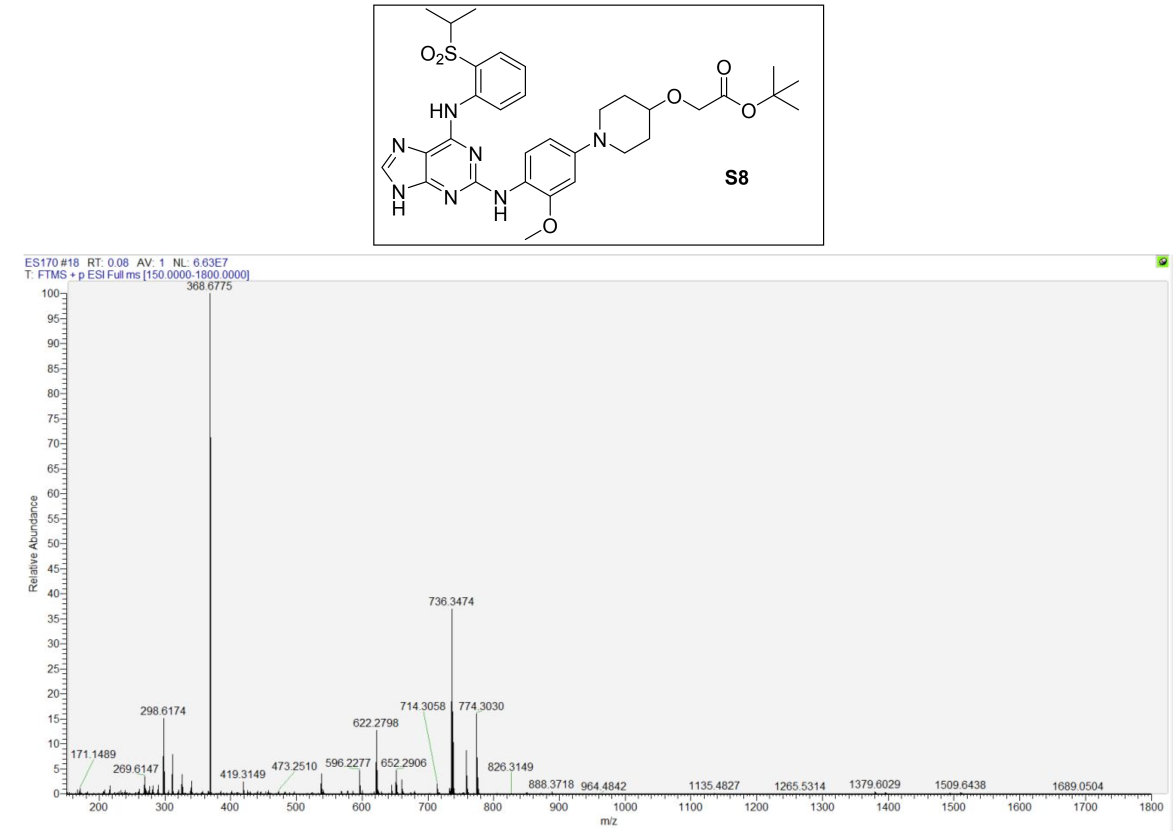

Figure S54. ESI-HRMS m/z for $C_{37}H_{50}N_7O_7S$ $[M+H]^+$ calcd 736.3492, found 736.3474.

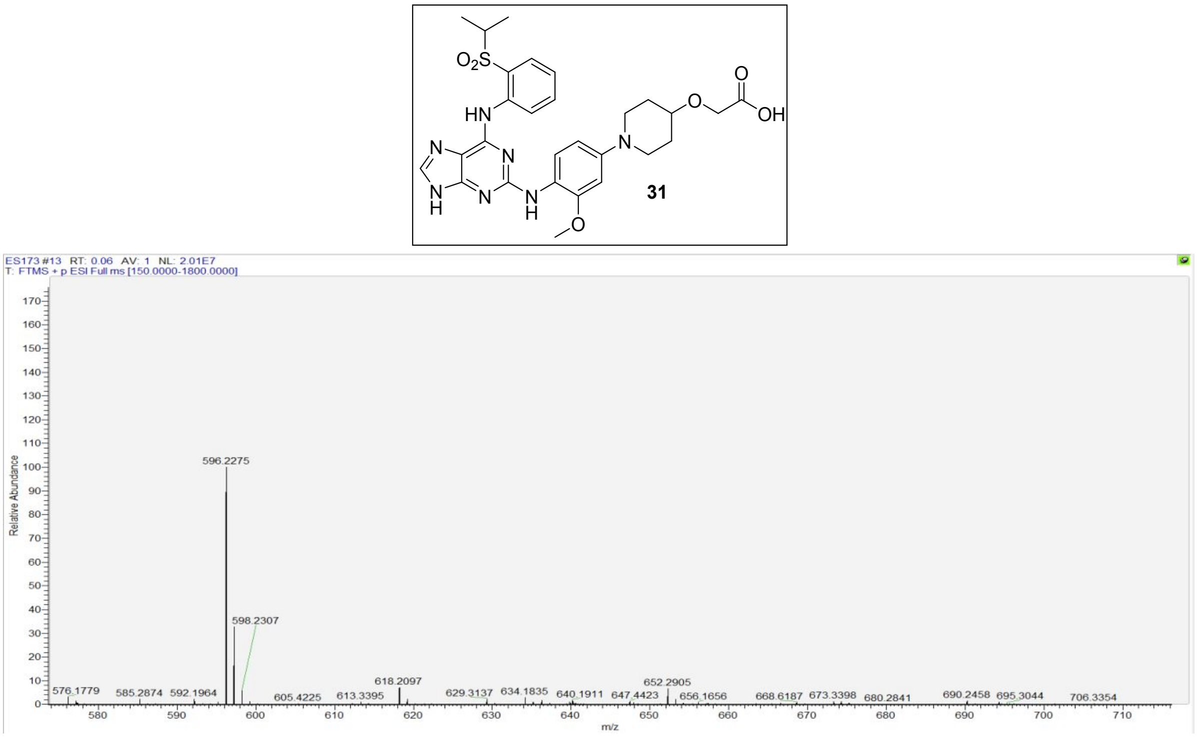

Figure S55. ESI-HRMS m/z for $C_{28}H_{34}N_7O_6S$ $[M+H]^+$ calcd 596.2291, found 596.2275.

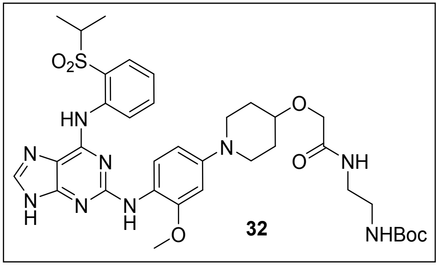

32

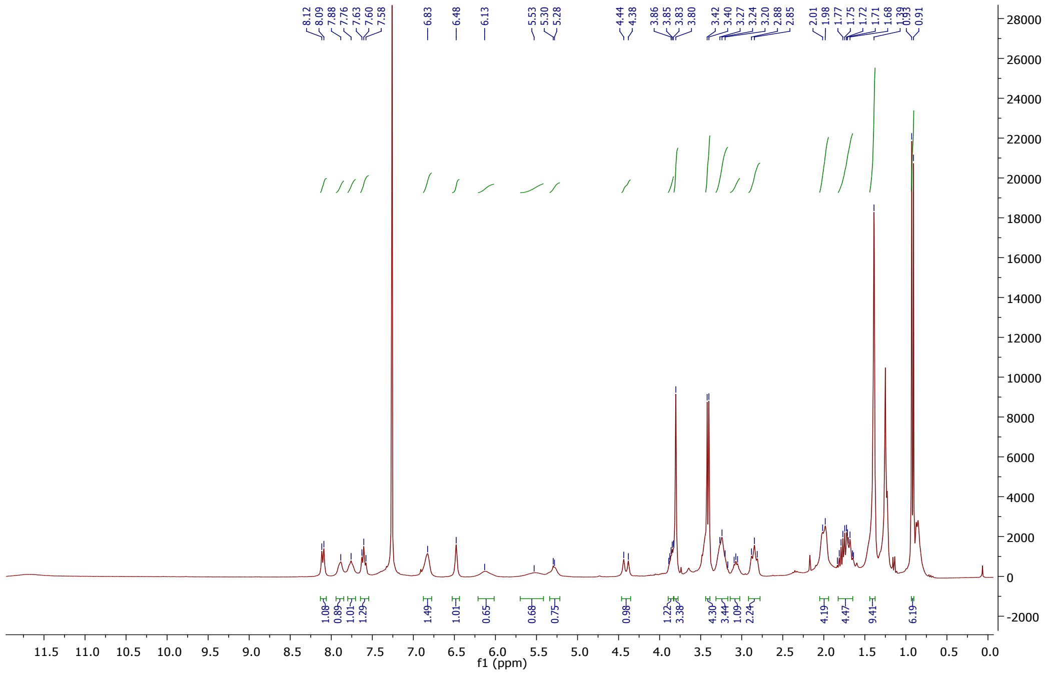

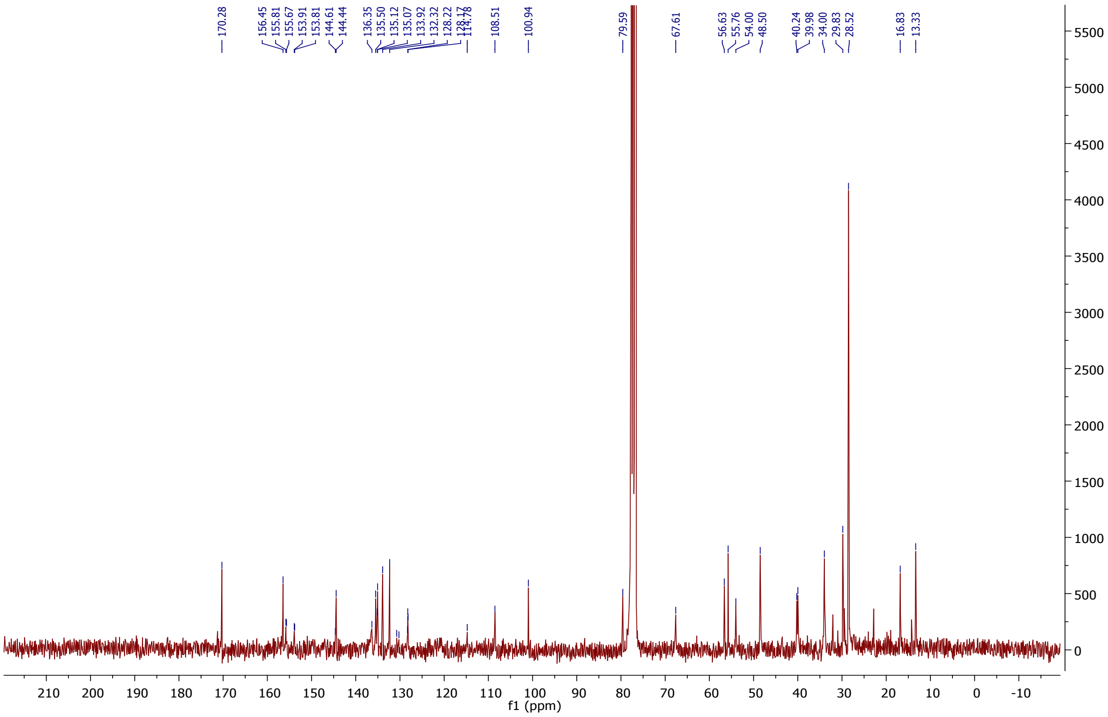

Figure S56

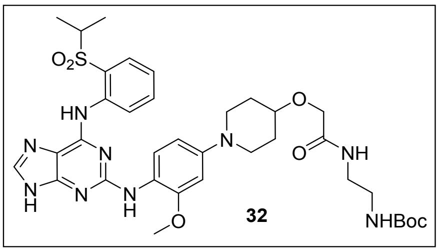

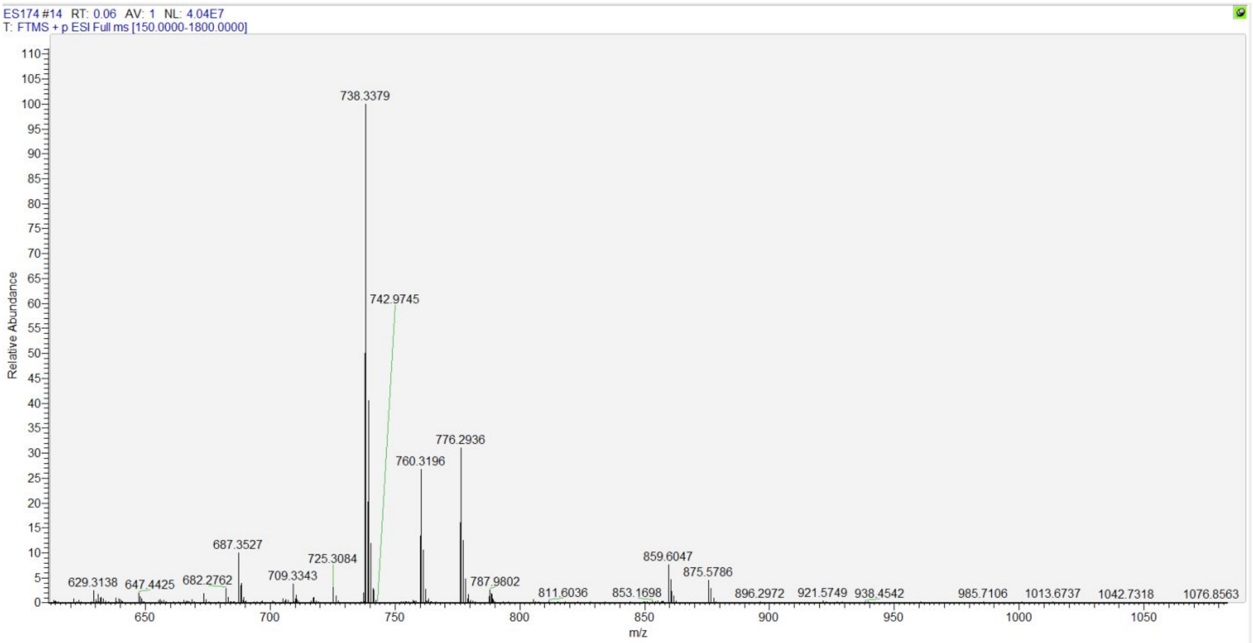

Figure S57. ESI-HRMS m/z for $C_{35}H_{48}N_9O_7S$ $[M+H]^+$ calcd 738.3397, found 738.3379.

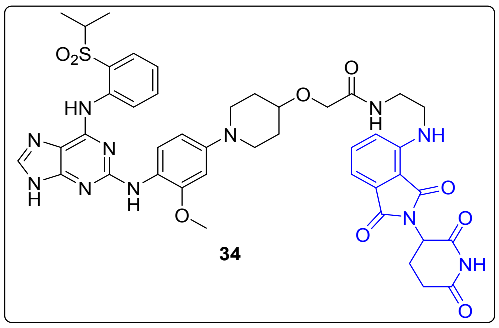

34

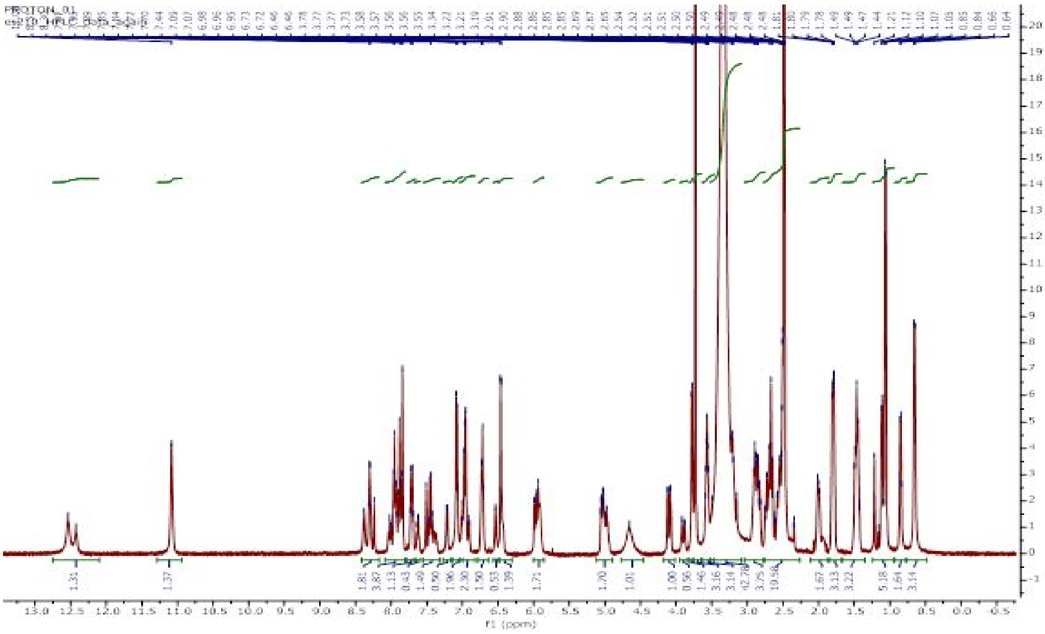

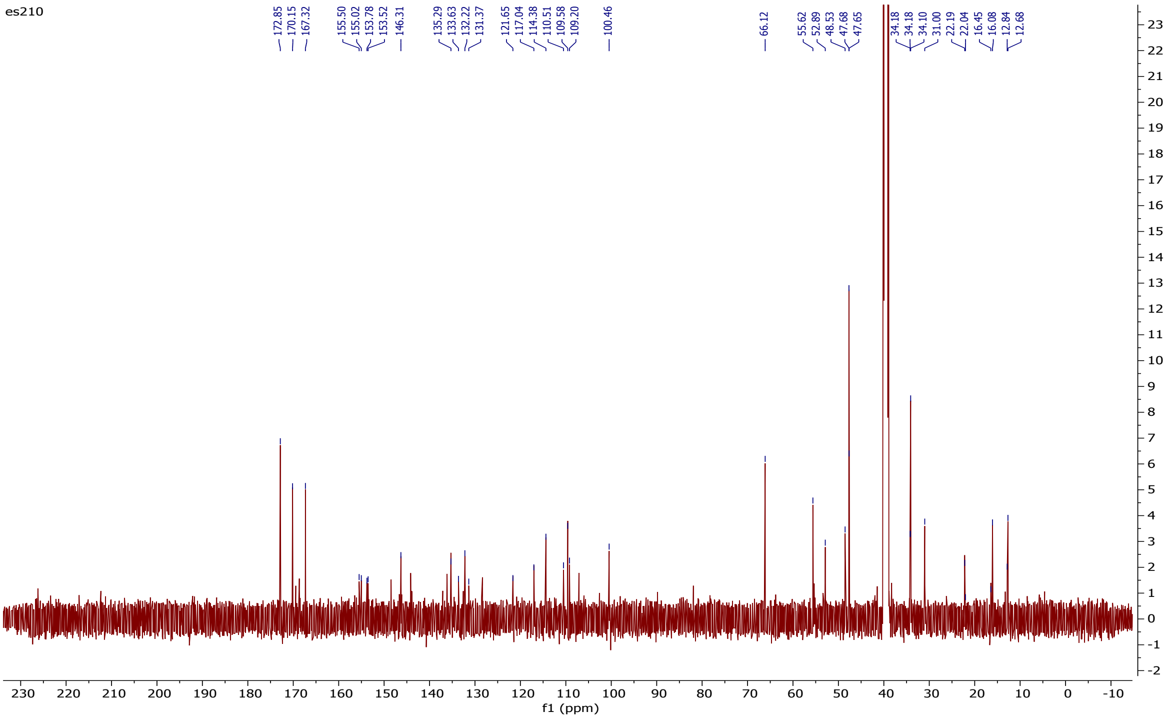

Figure S58

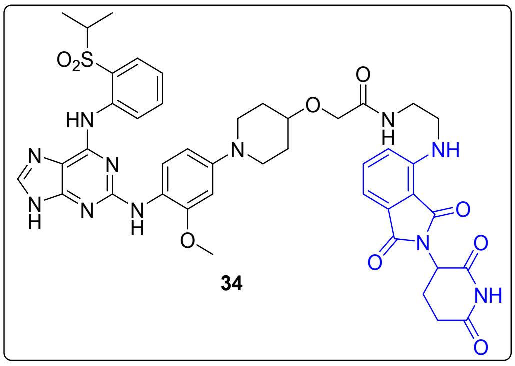

Analytical method 2

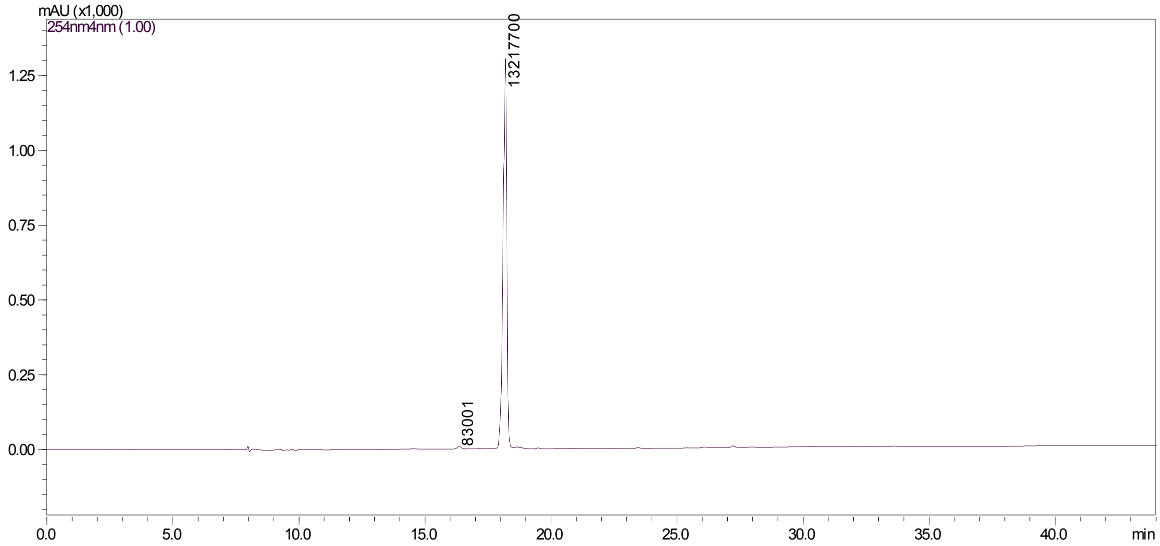

Area: 13217700

Total Area: 13300701

Purity (%)= 99.3%

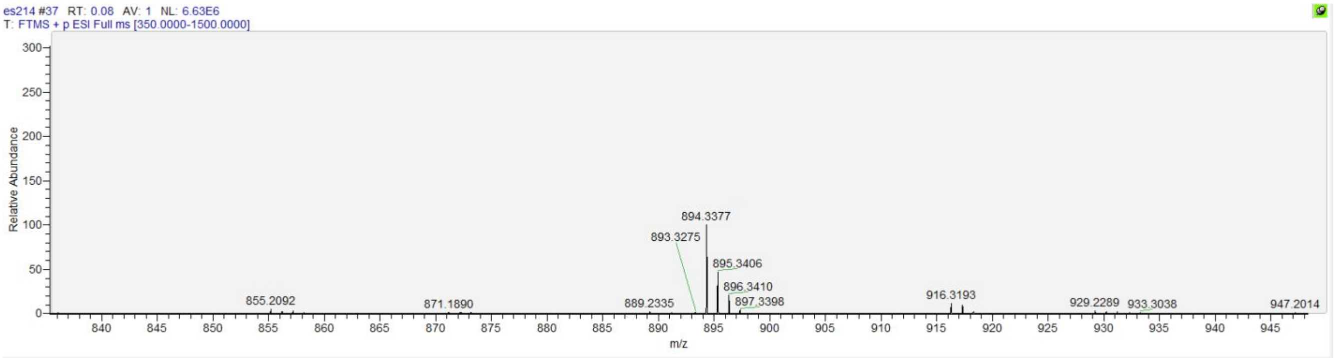

ESI-HRMS m/z for $C_{43}H_{48}N_{11}O_9S$ $[M+H]^+$ calcd 894.3352, found 894.3377.

Figure S59

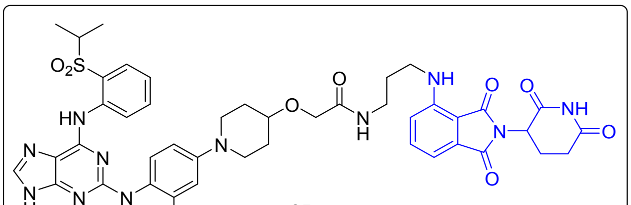

35

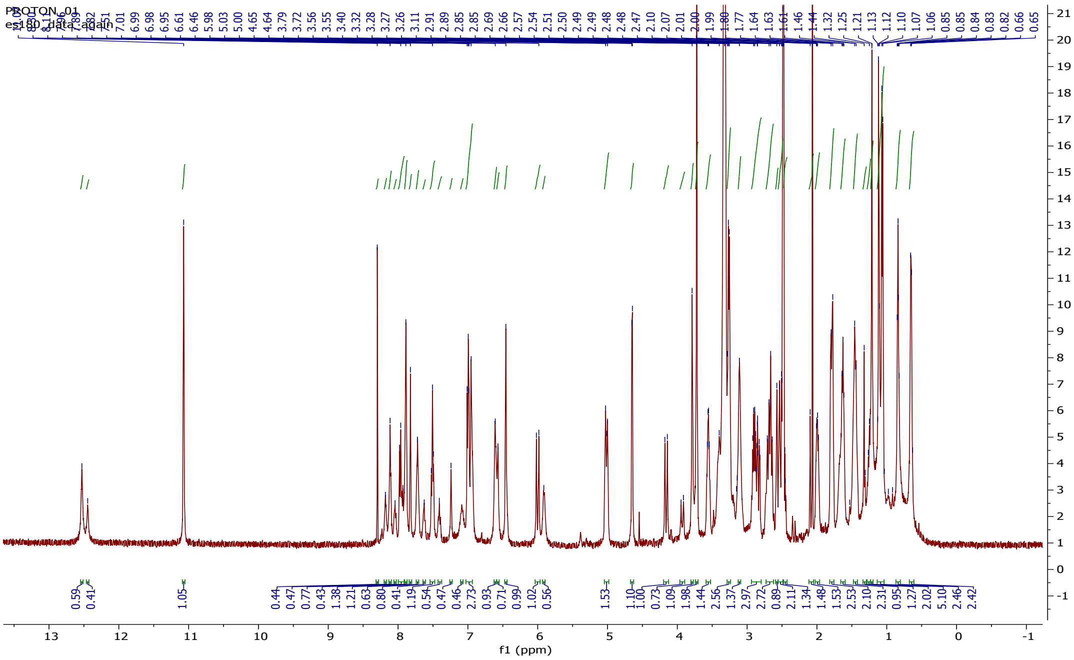

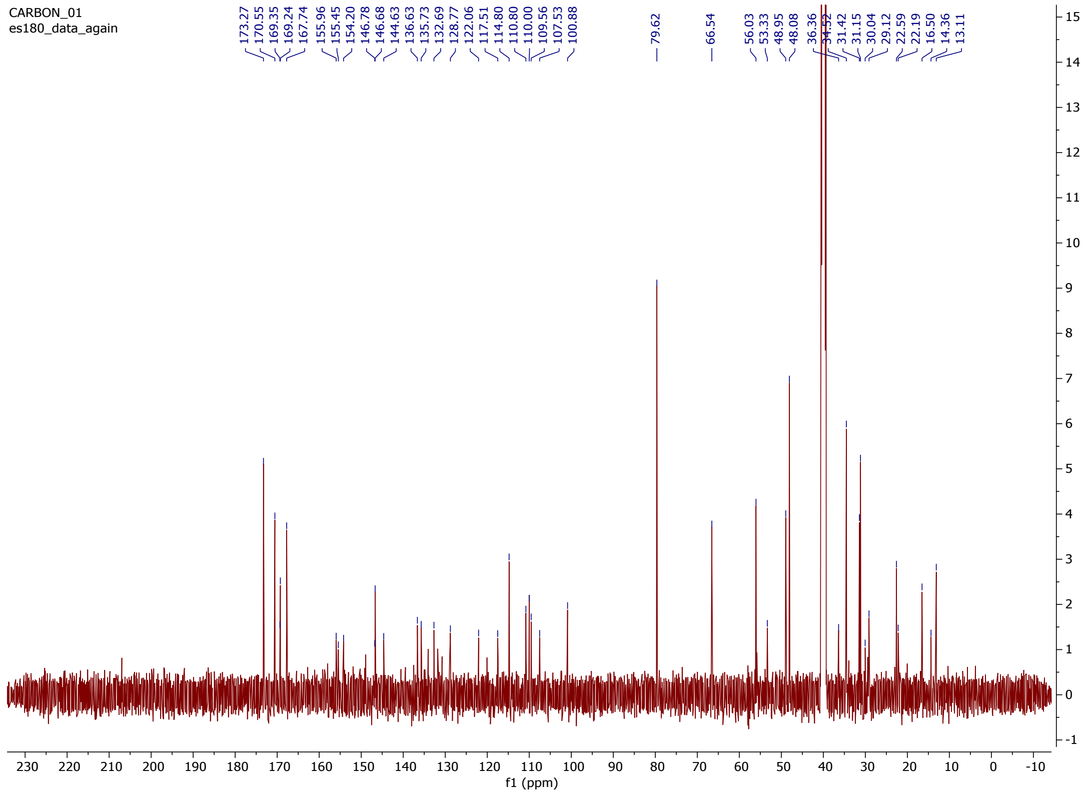

Figure S60

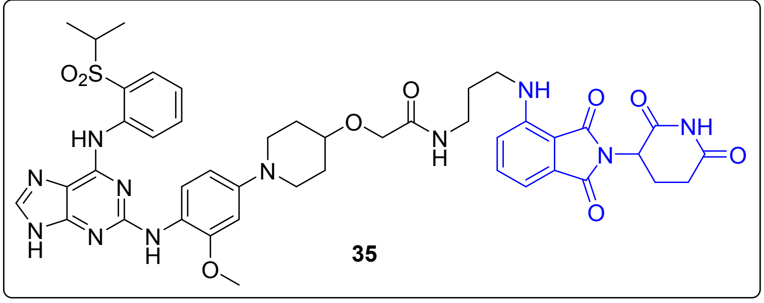

35

Analytical method 1

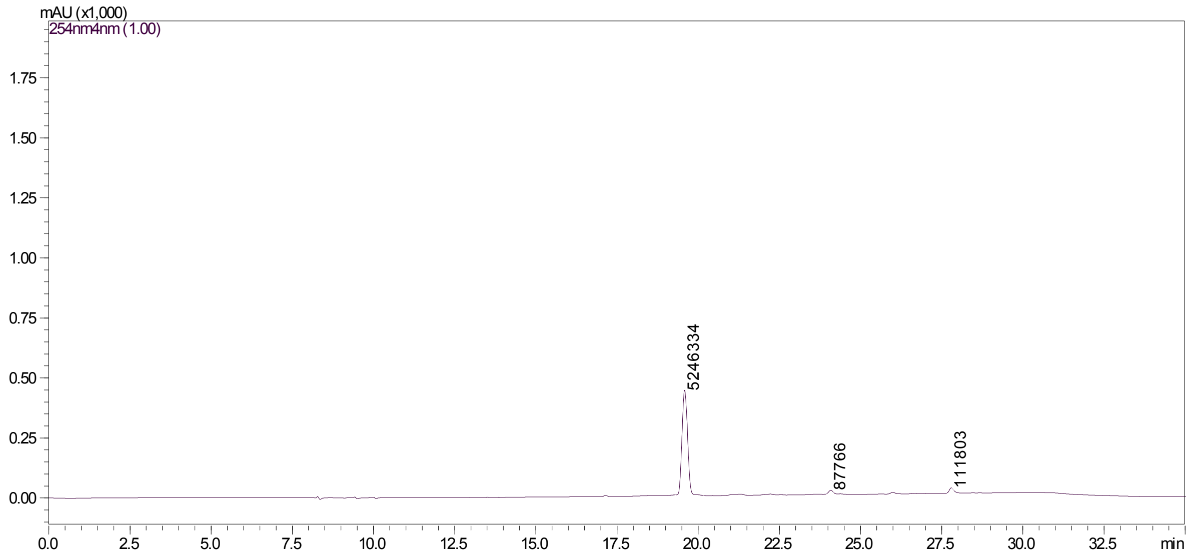

total area: 5445903

Area: 5246334

%=96.3%

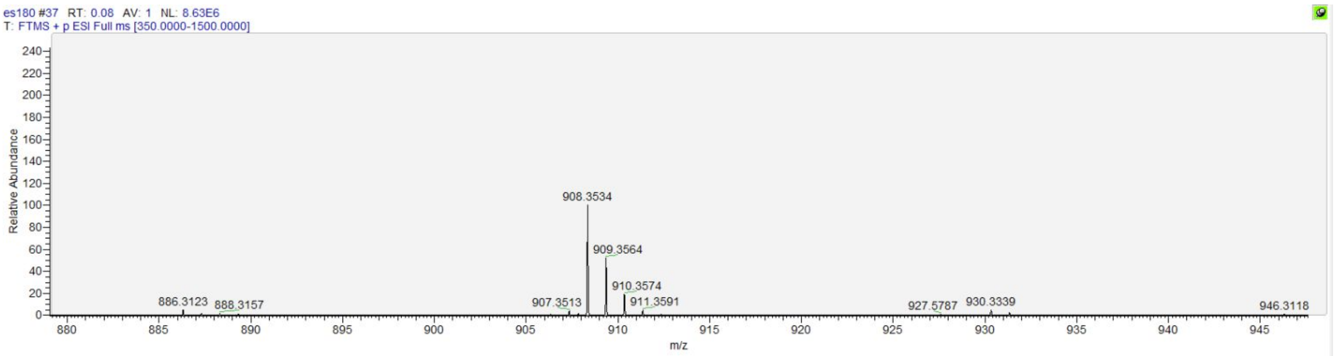

ESI-HRMS m/z for $C_{44}H_{50}N_{11}O_9S$ $[M+H]^+$ calcd 908.3508, found 908.3534.

Figure S61

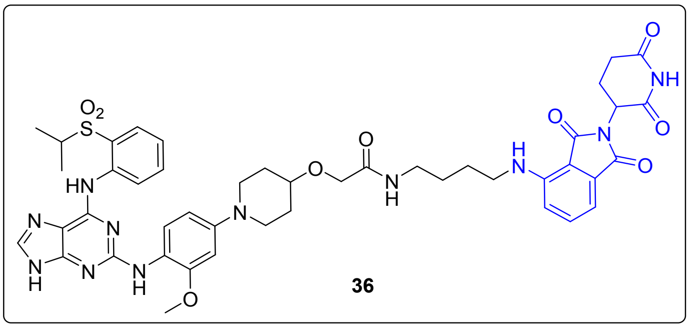

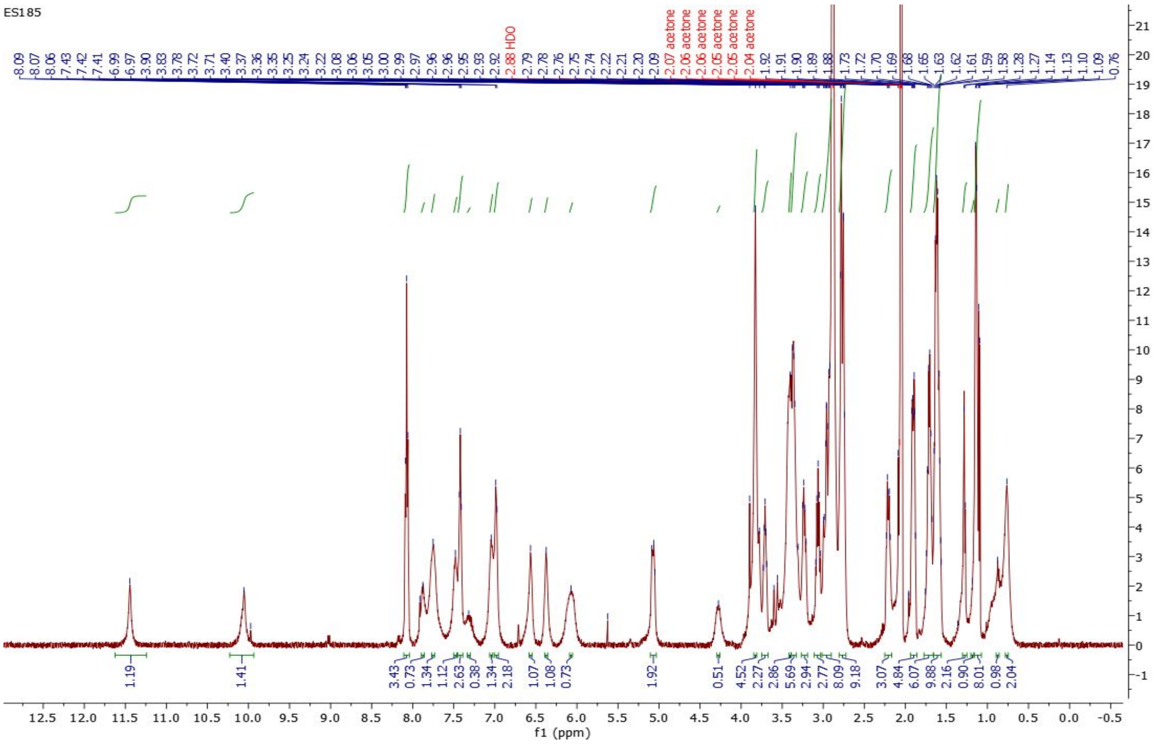

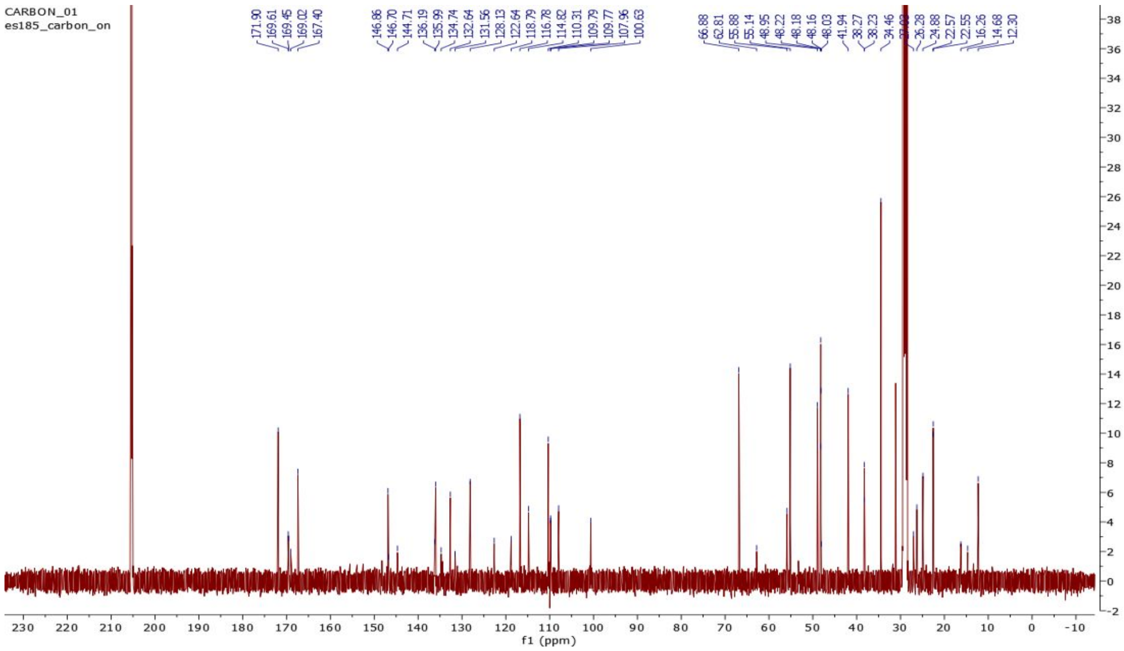

Figure S62

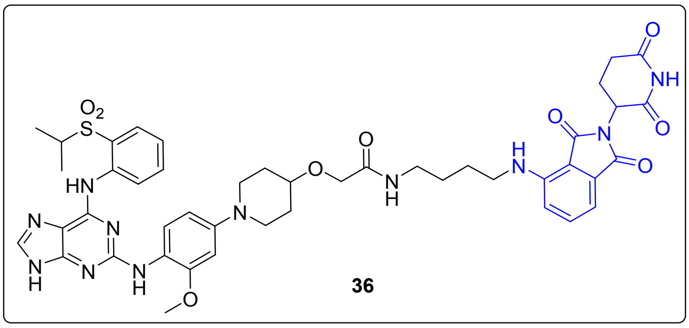

36

Analytical method 1

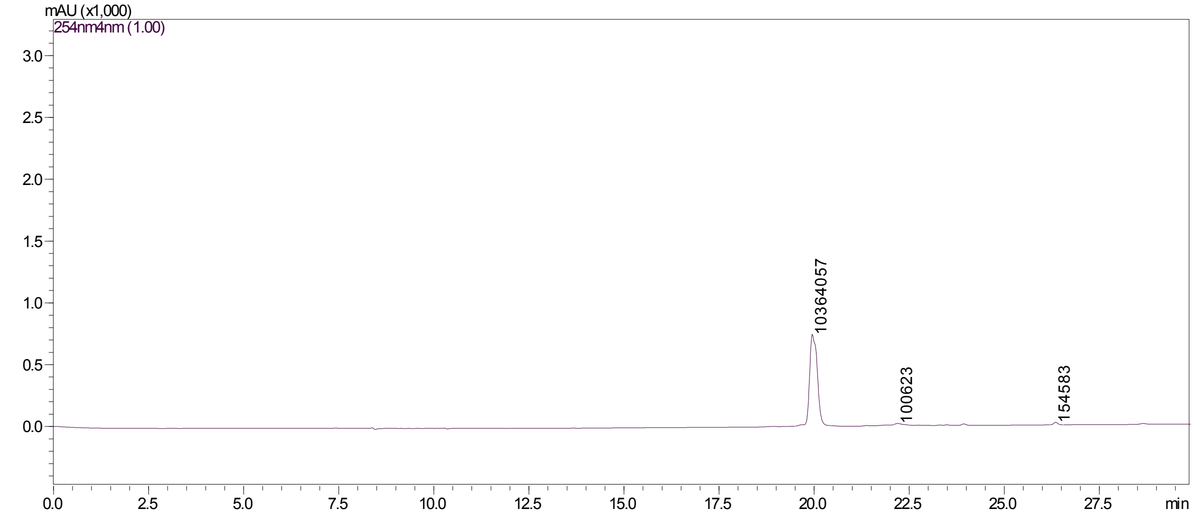

Area: 10364057

Total area: 10619263

Purity (%)=97.6%

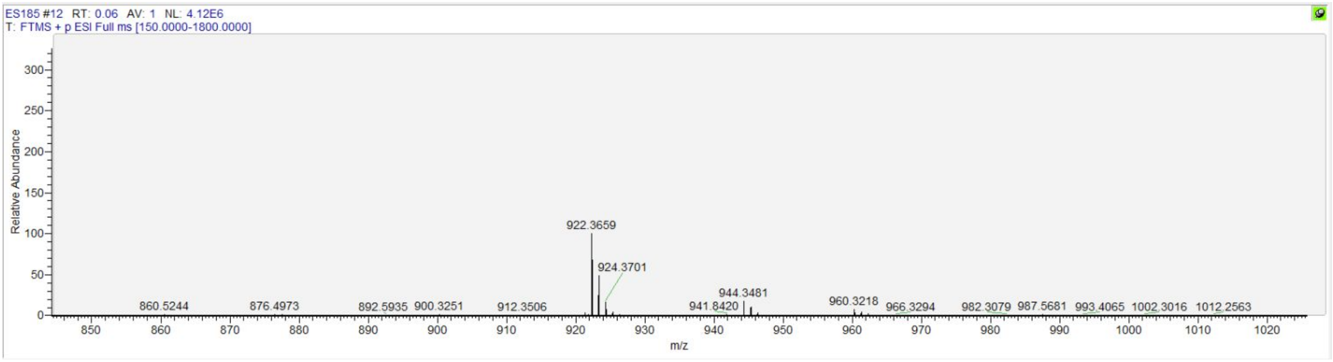

ESI-HRMS m/z for $C_{45}H_{52}N_{11}O_9S$ $[M+H]^+$ calcd 922.3670, found 922.3659.

Figure S63

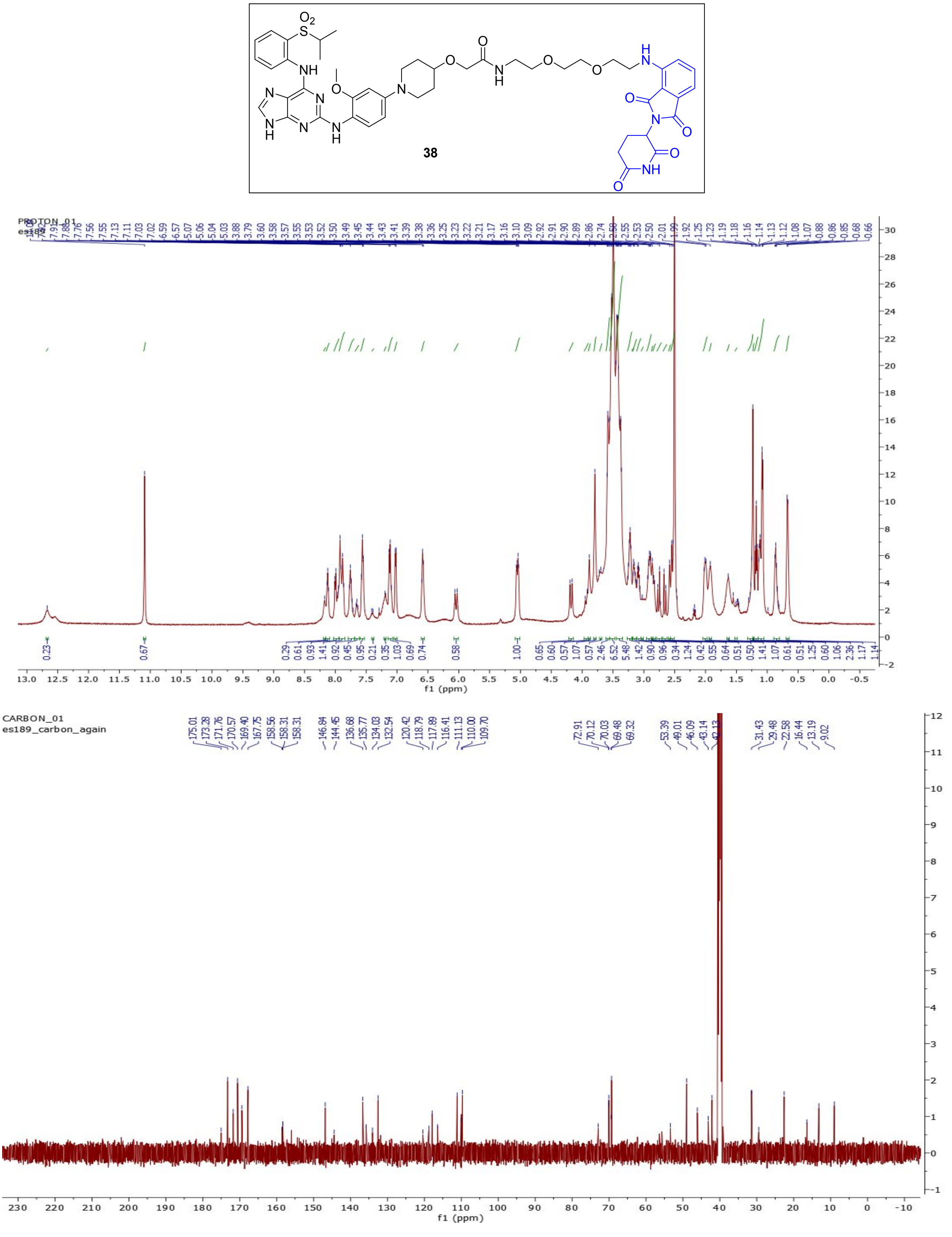

Figure S64

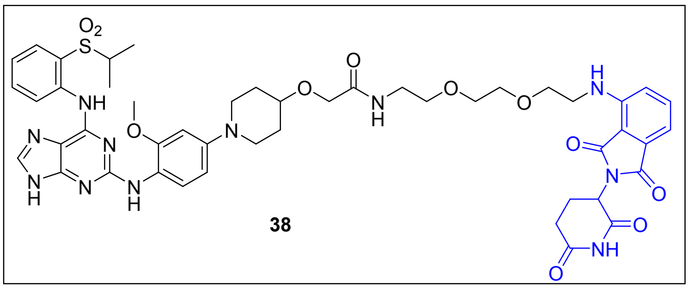

38

Analytical method 2

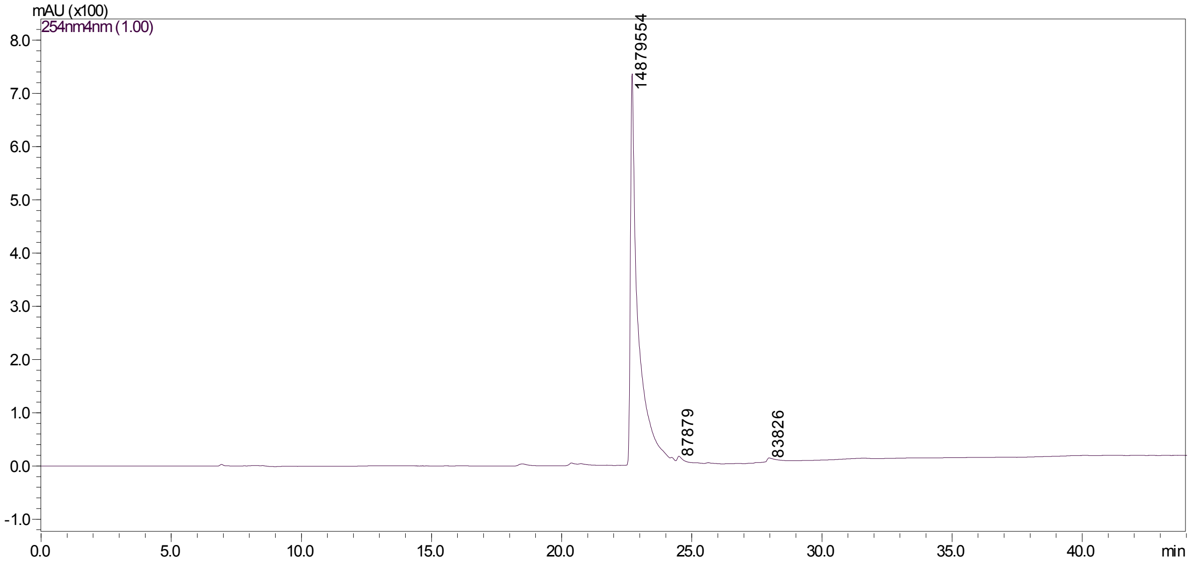

Area: 14879554

Total Area: 15051261

Purity (%) = 98.8%

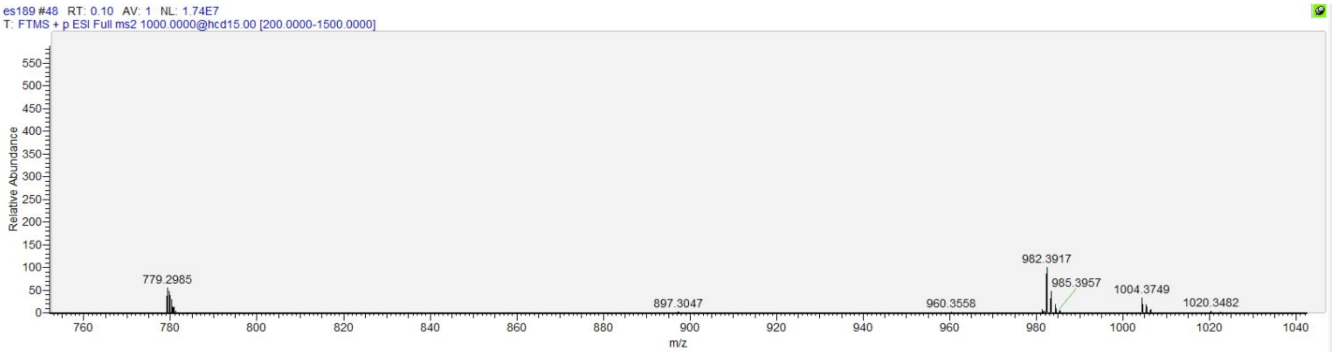

ESI-HRMS m/z for $C_{47}H_{56}N_{11}O_{11}S$ $[M+H]^+$ calcd 982.3876, found 982.3917.

Figure S65

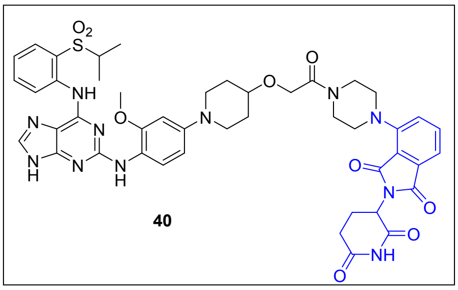

40

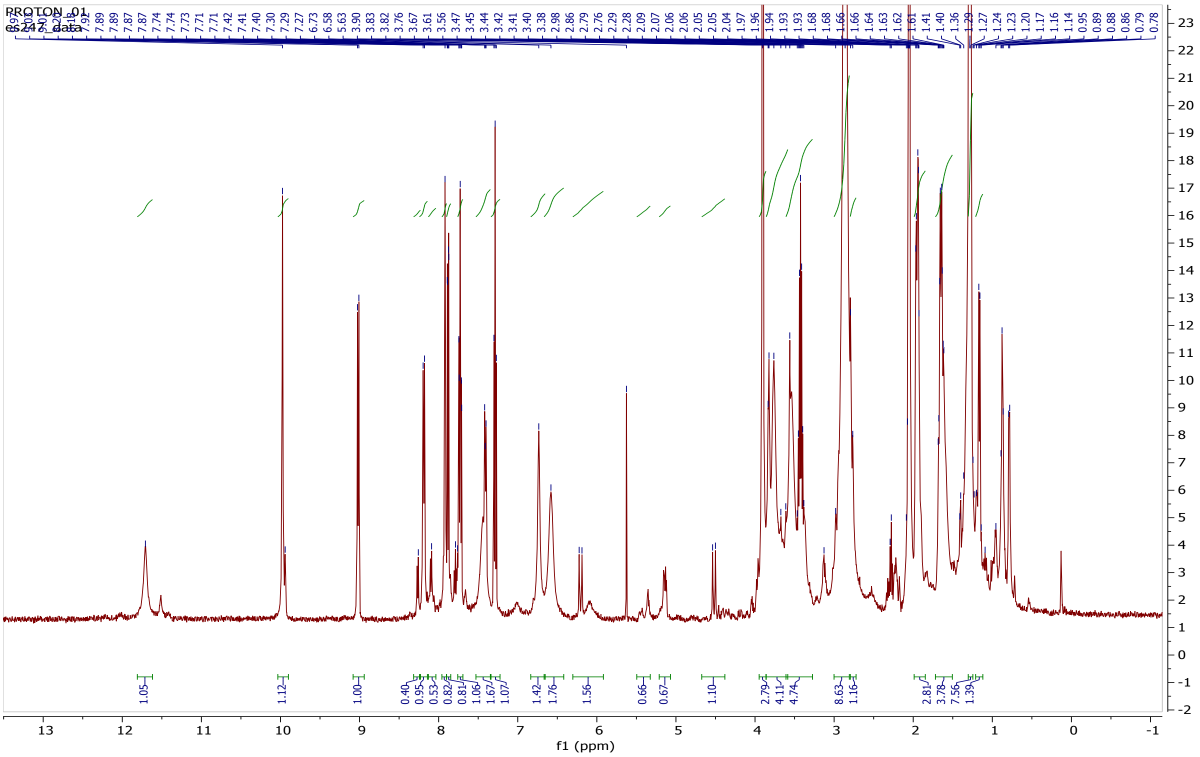

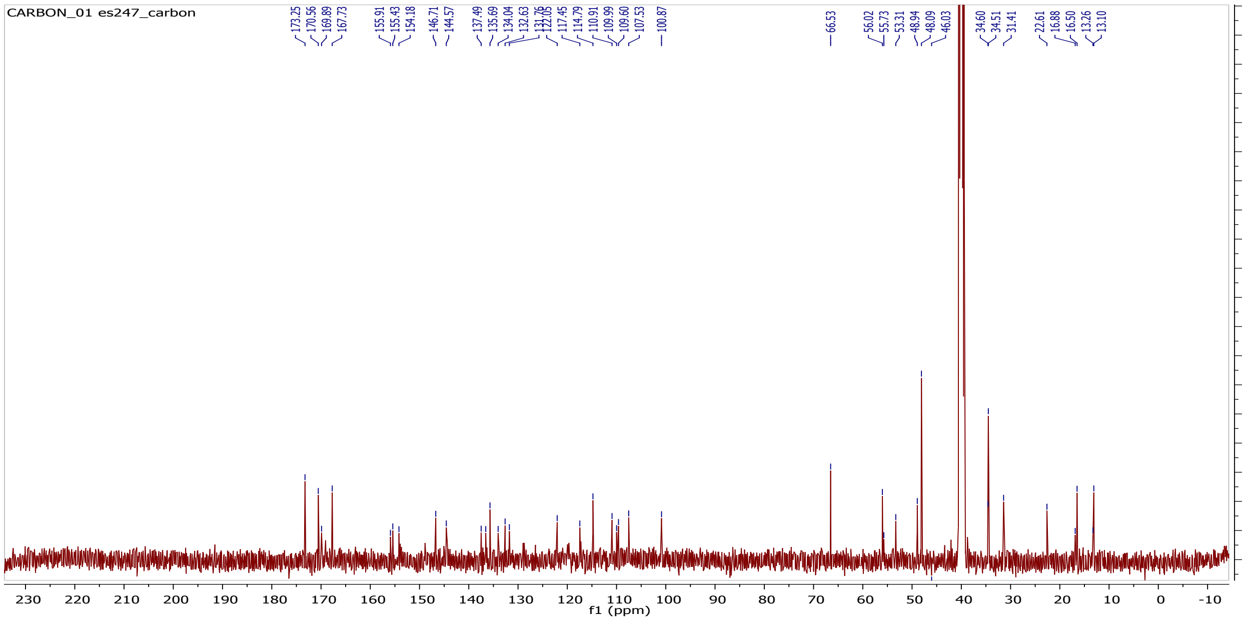

Figure S66

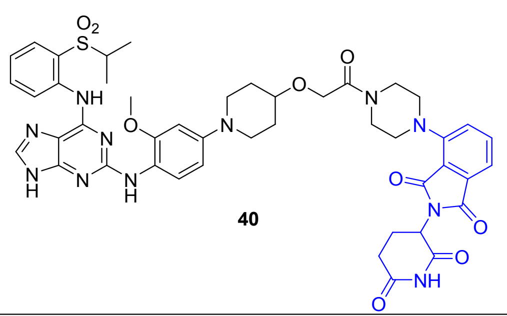

Analytical method 2

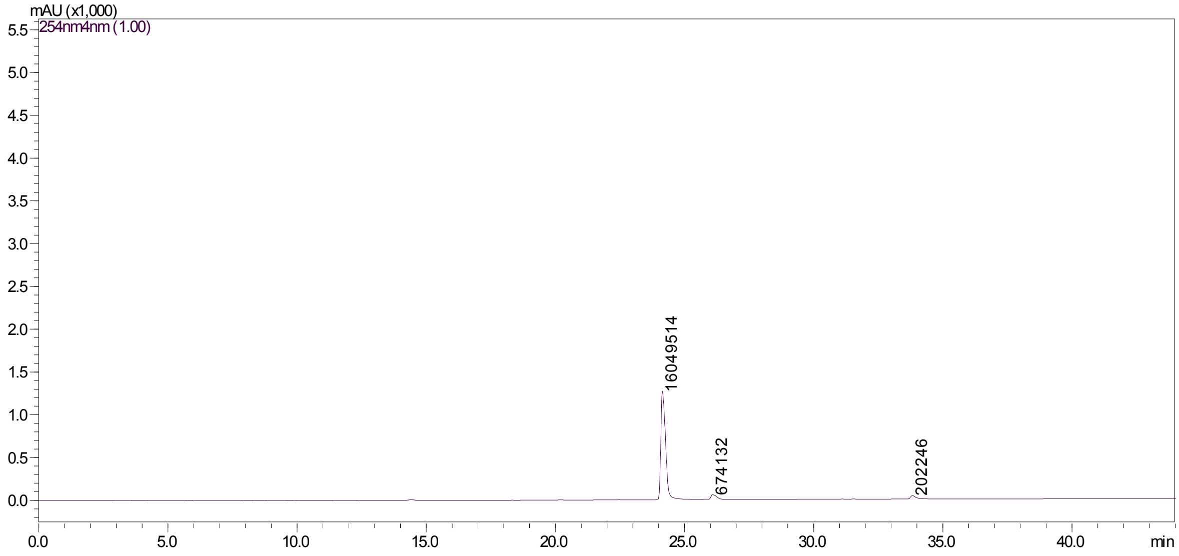

total area: 16925892

peak area: 16049514

%=94.9 %

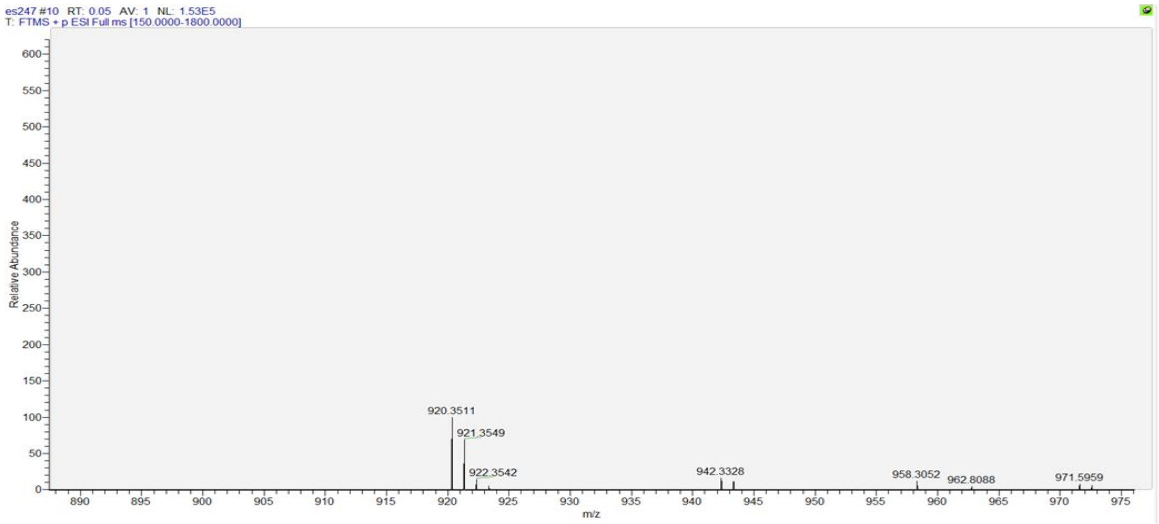

ESI-HRMS m/z for $C_{45}H_{50}N_{11}O_9S$ $[M+H]^+$ calcd 920.3514, found 920.3511.

Figure S67

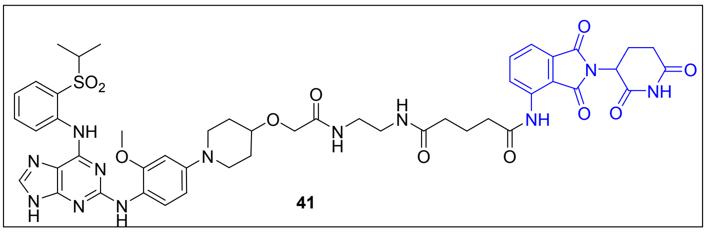

41

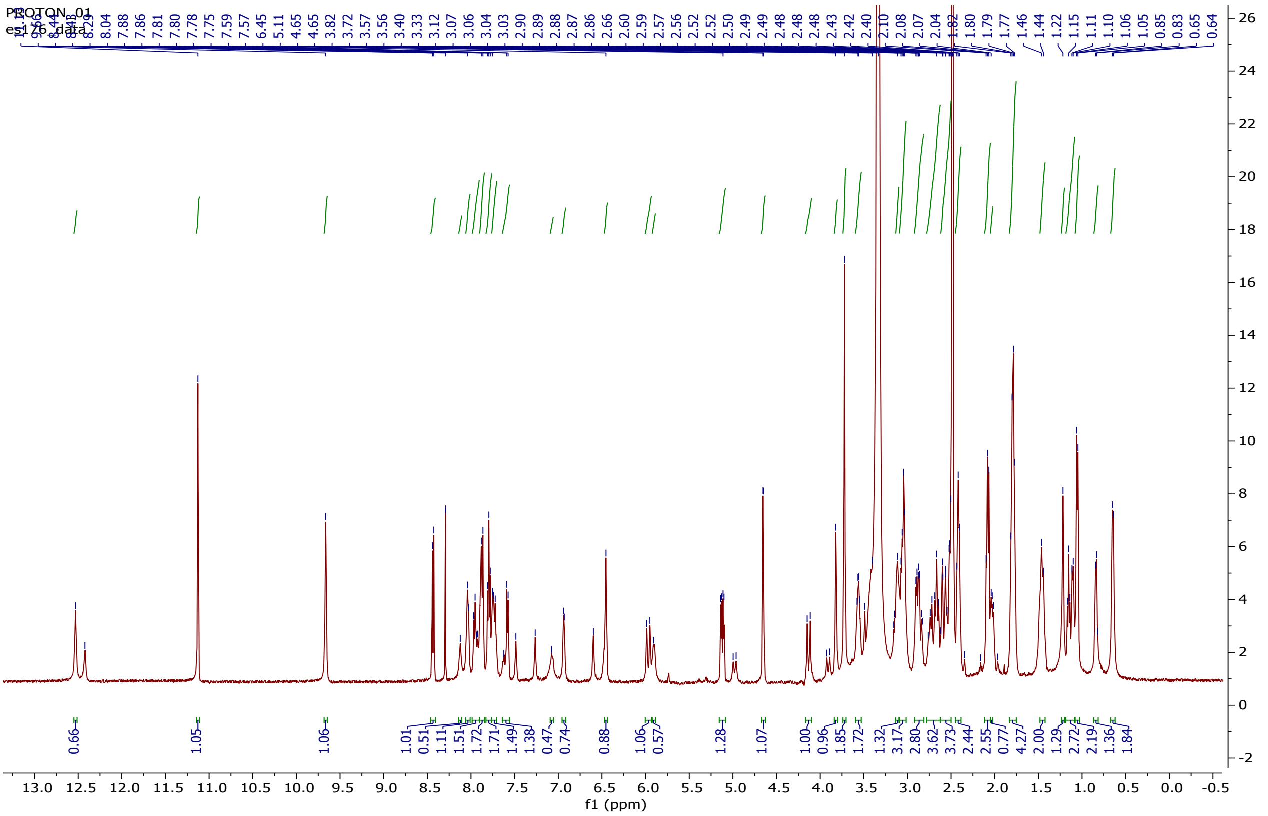

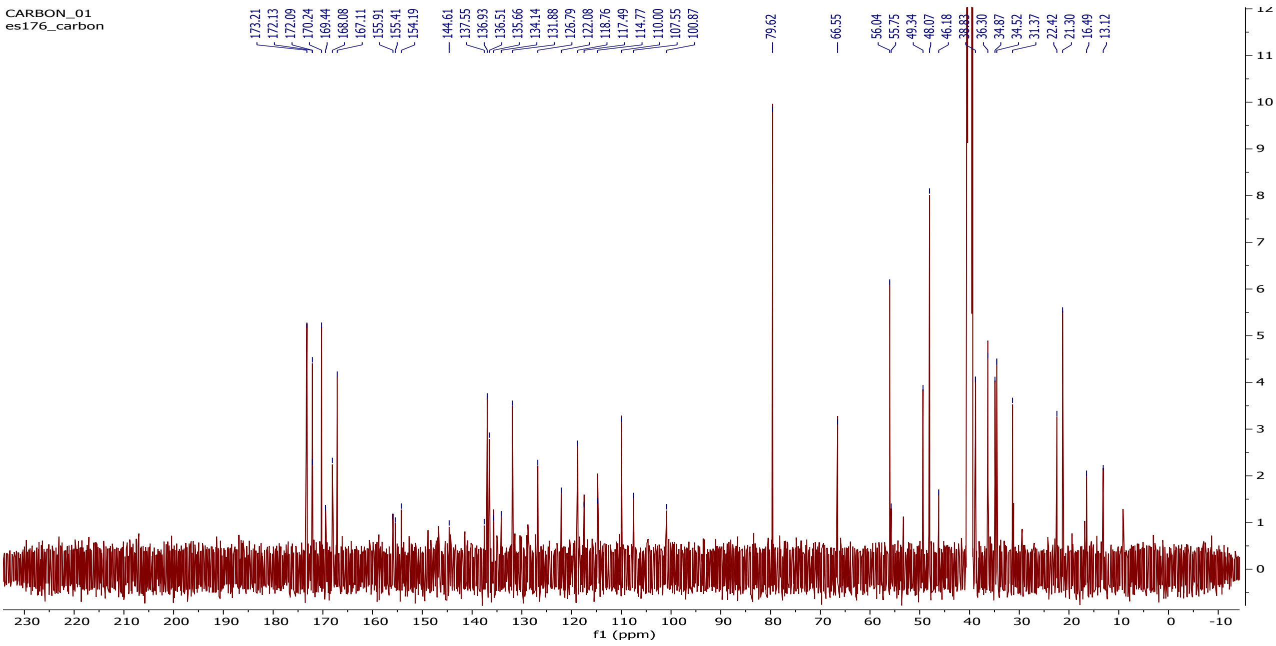

Figure S68

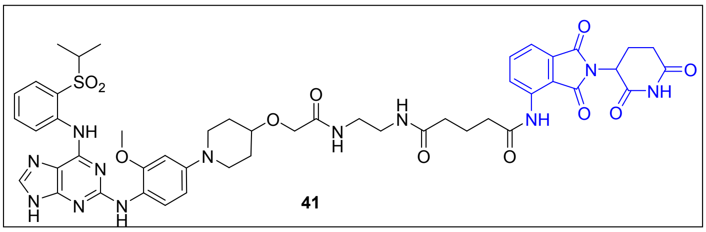

Analytical method 2

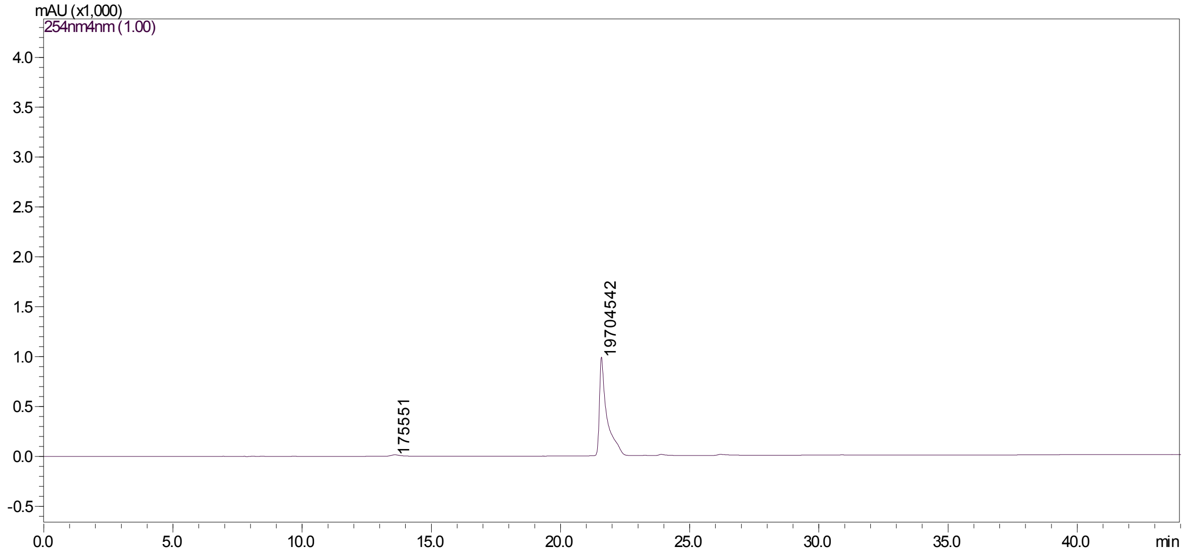

Area: 19704542

Total area: 19880093

Purity (%) = 99.1%

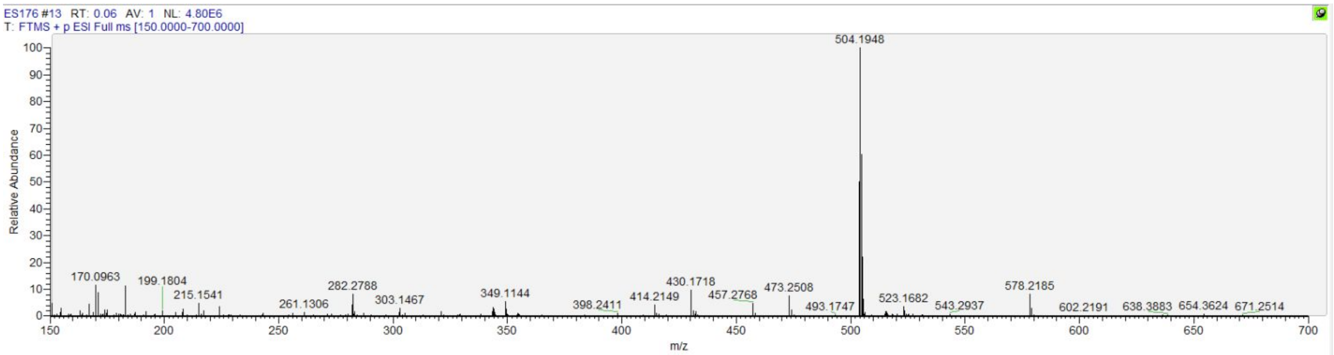

ESI-HRMS m/z for $C_{48}H_{55}N_{12}O_{11}S$ $[M/2+H]^+$ calcd 504.1956, found 504.1948.

Figure S69

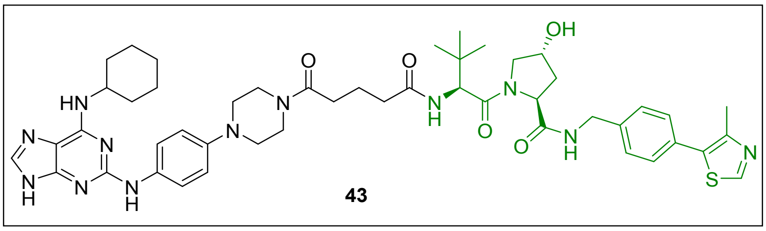

43

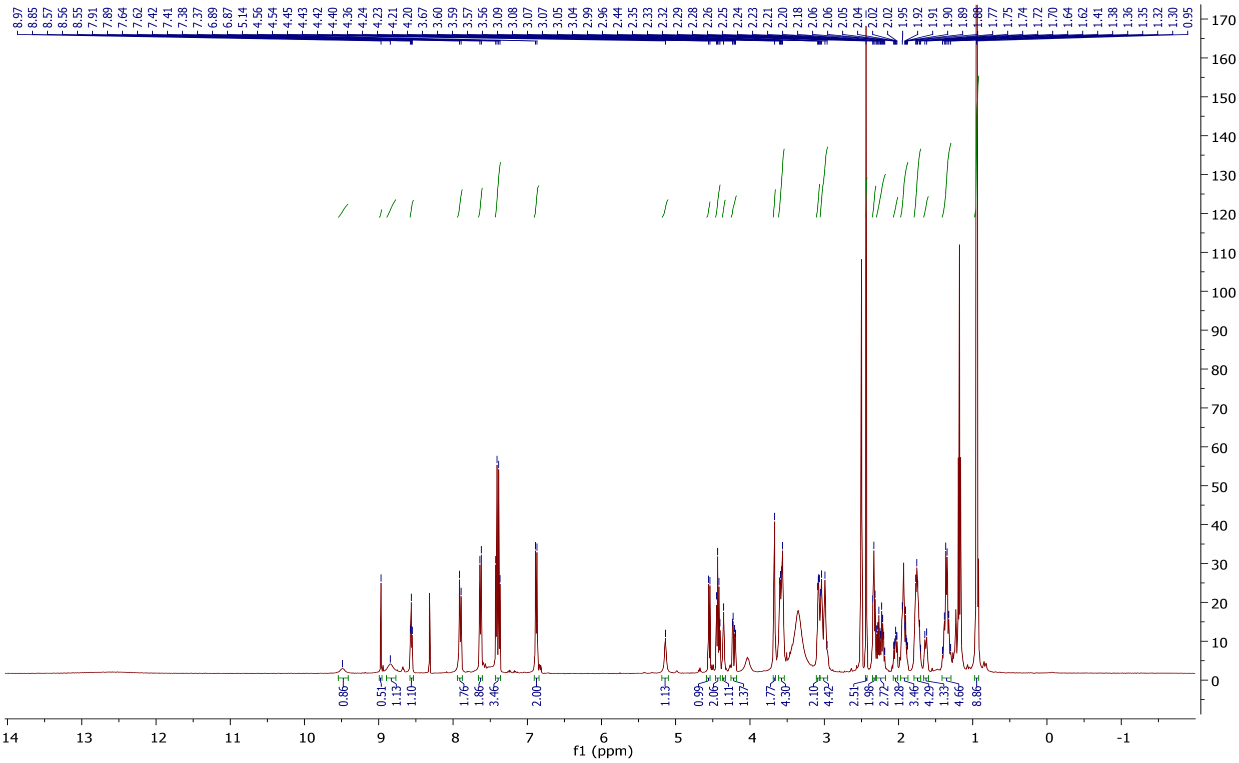

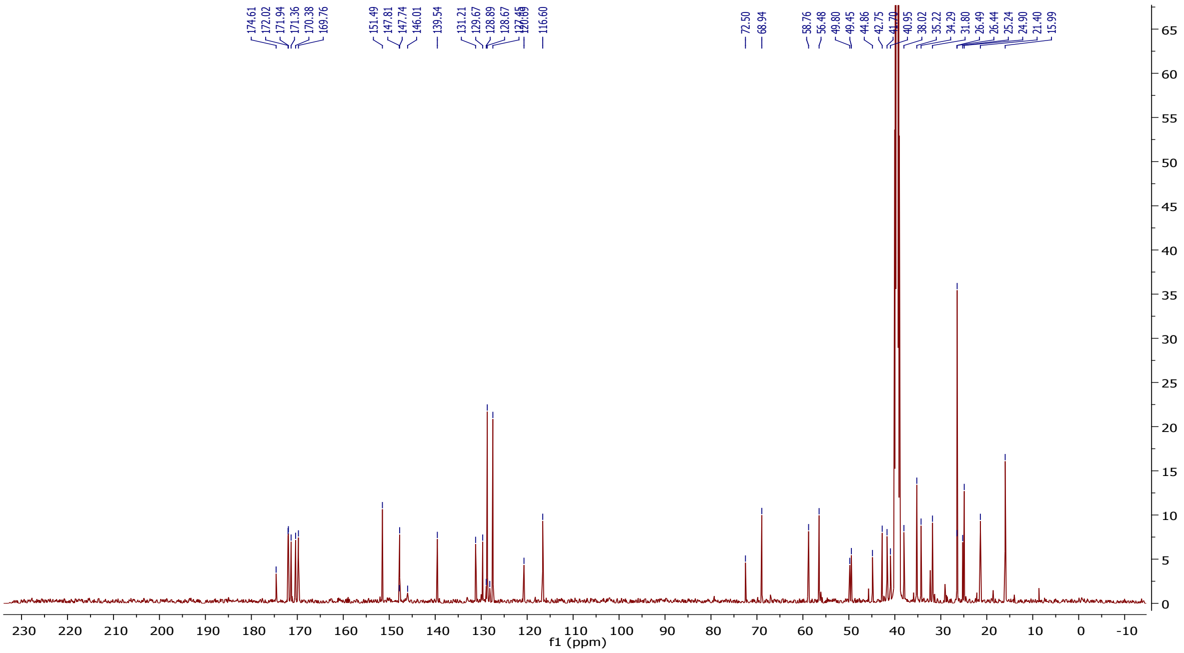

Figure S70

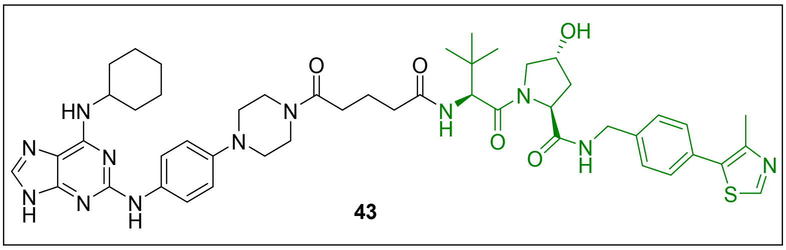

Analytical method 2

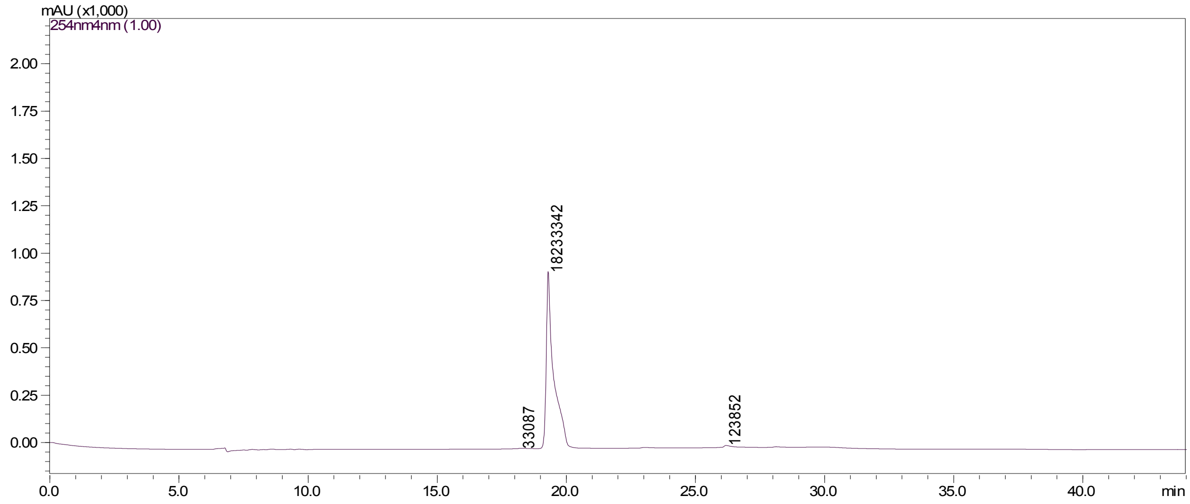

Area= 18233342

Total Area= 18390281

Purity (%)= 99.1%

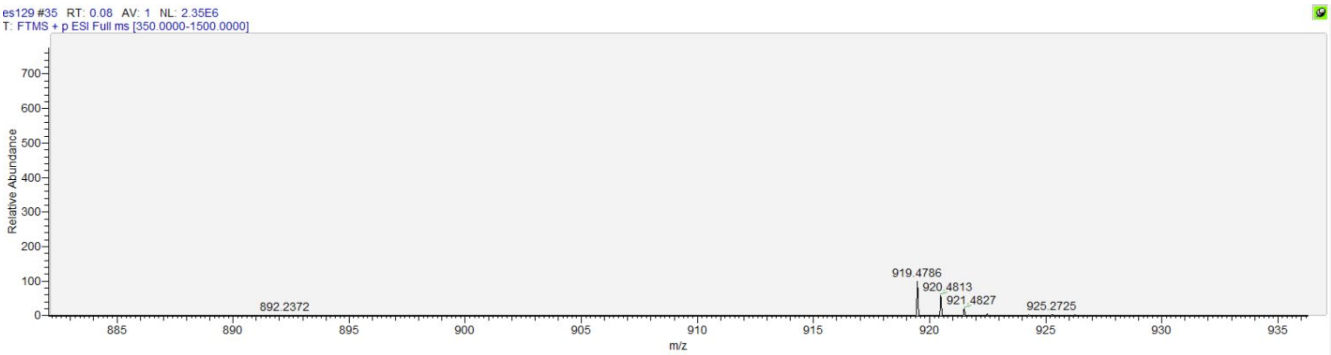

ESI-HRMS m/z for $C_{48}H_{63}N_{12}O_5S$ $[M+H]^+$ calcd 919.4760, found 919.4786.

Figure S71

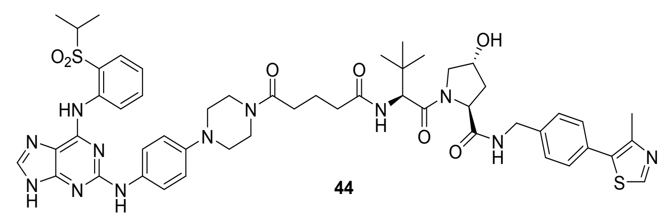

44

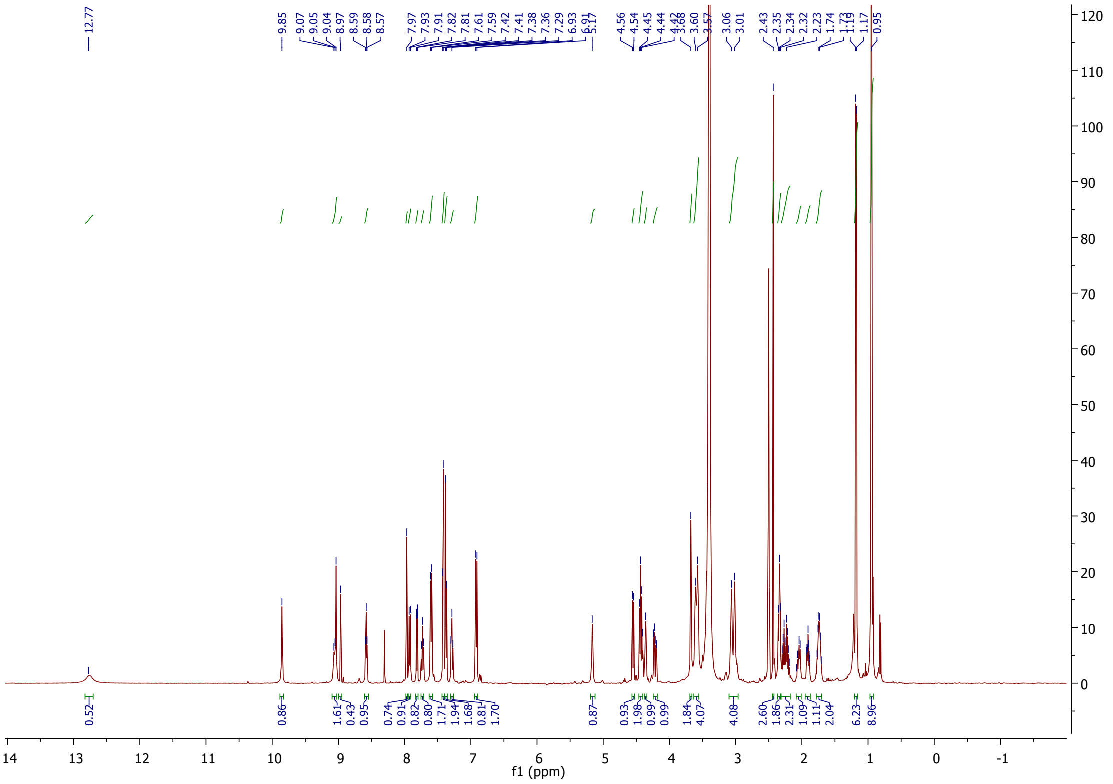

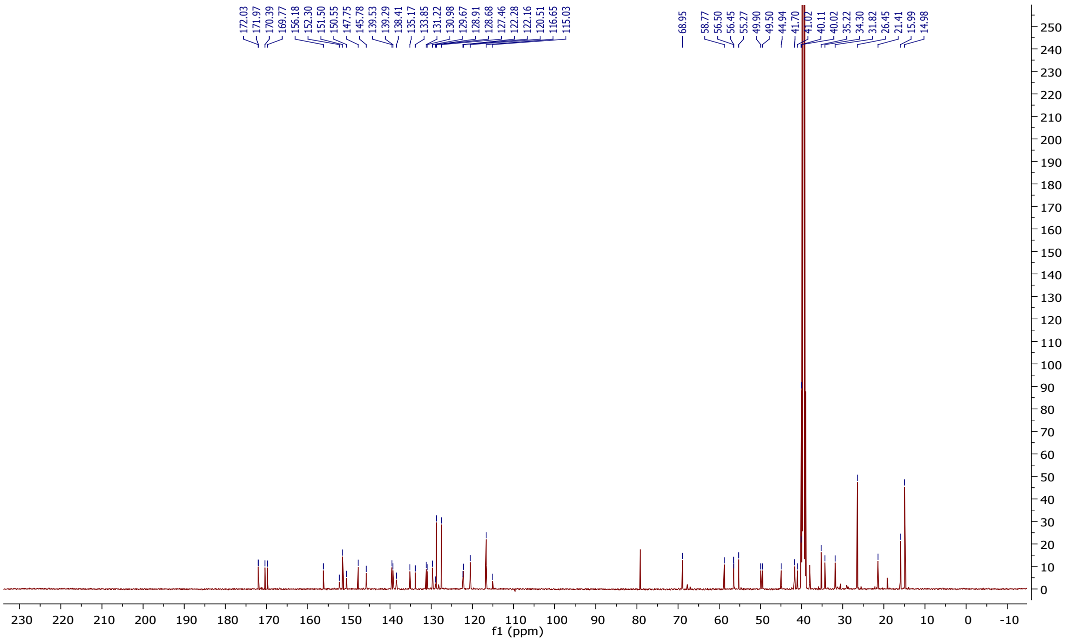

Figure S72

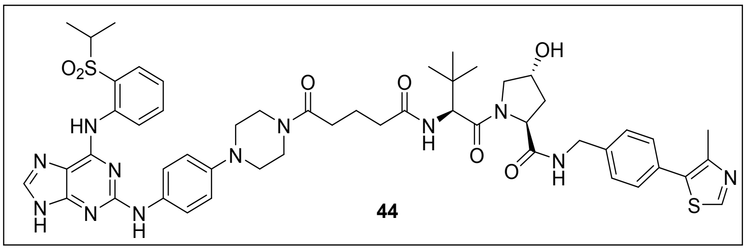

Analytical method 2

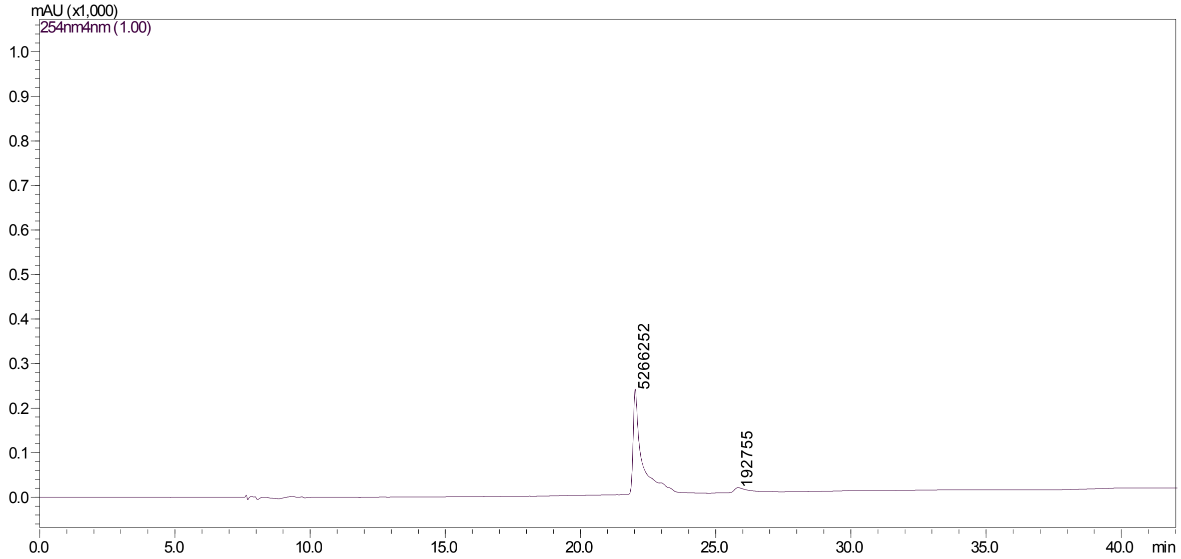

Area= 5266525

Total Area=5459007

(%)=96.5%

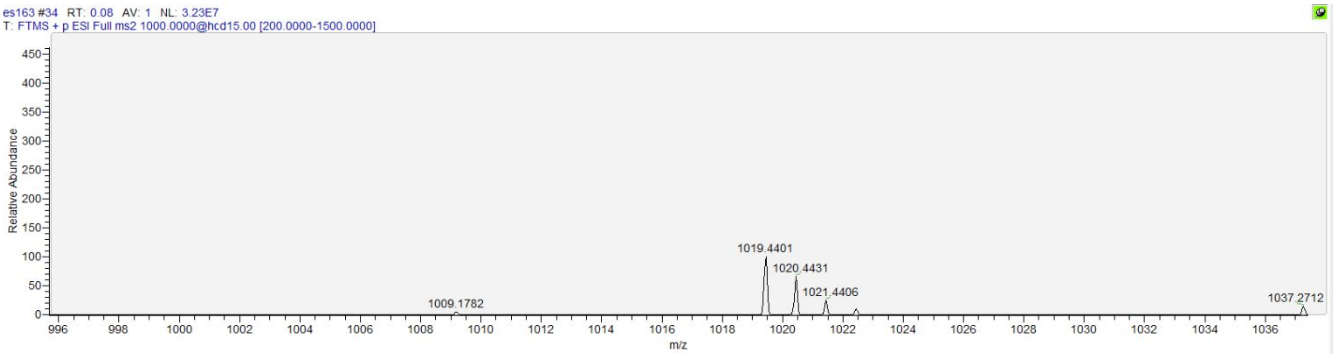

ESI-HRMS m/z for $C_{51}H_{63}N_{12}O_7S_2$ $[M+H]^+$ calcd 1019.4379, found 1019.4401.

Figure S73

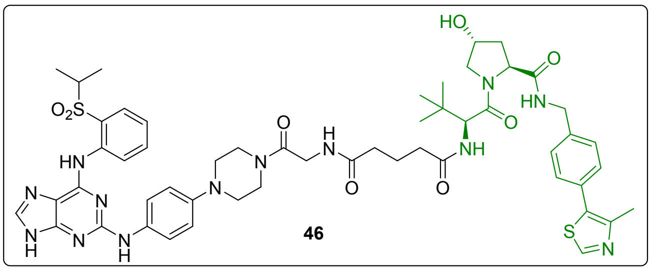

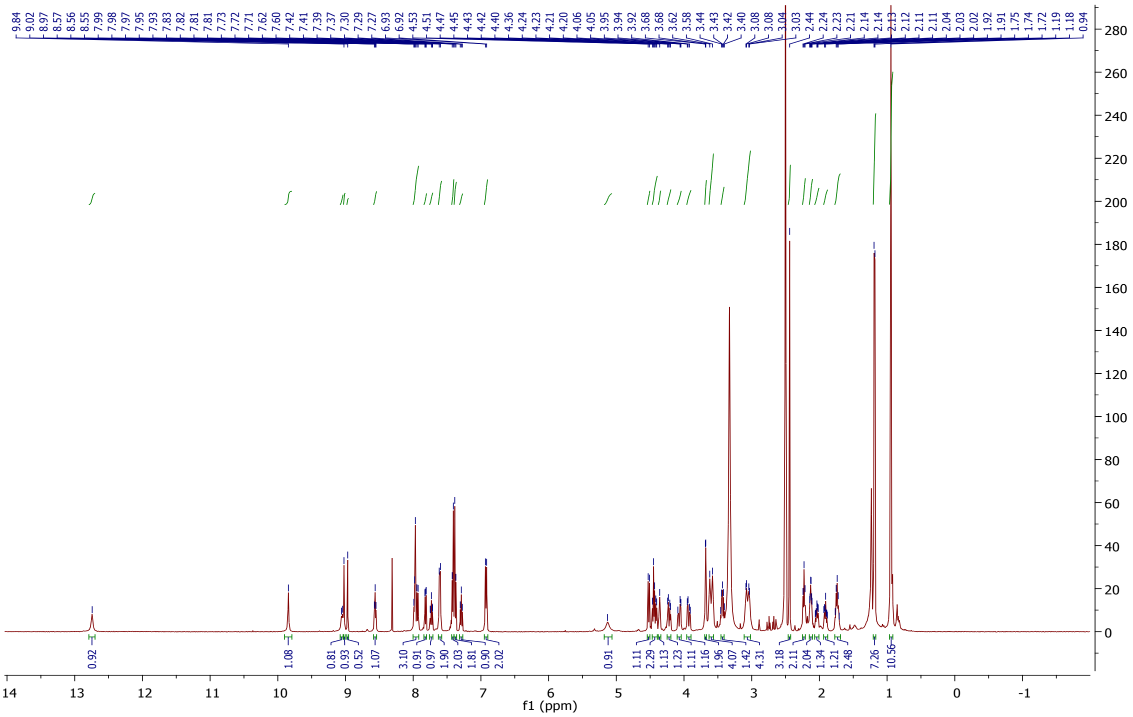

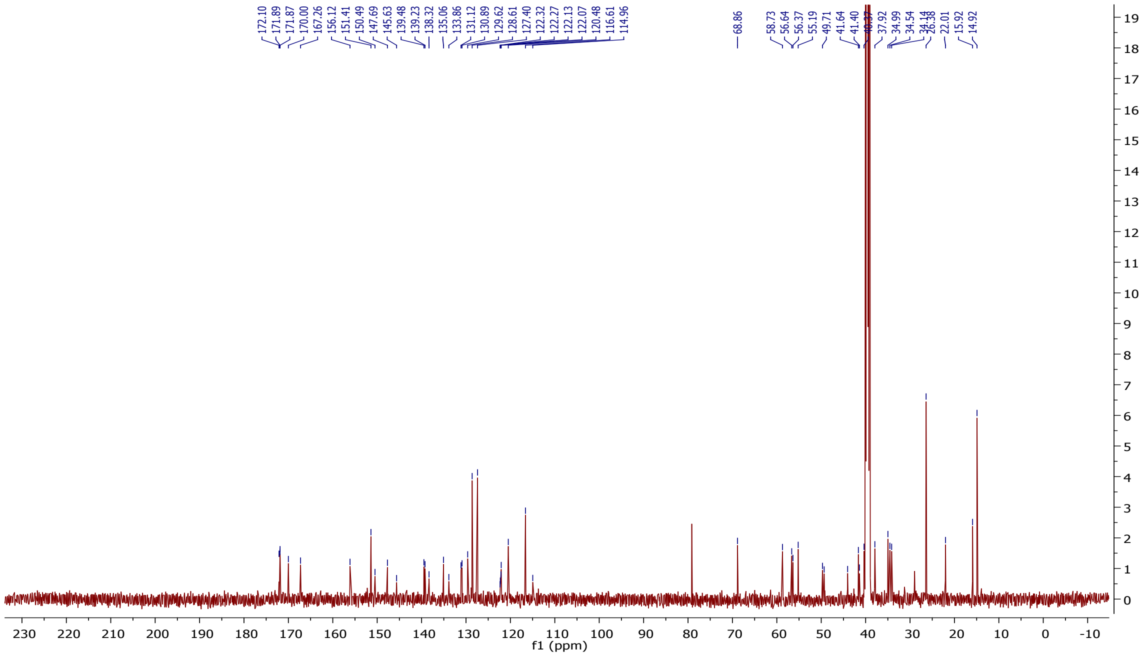

Figure S74

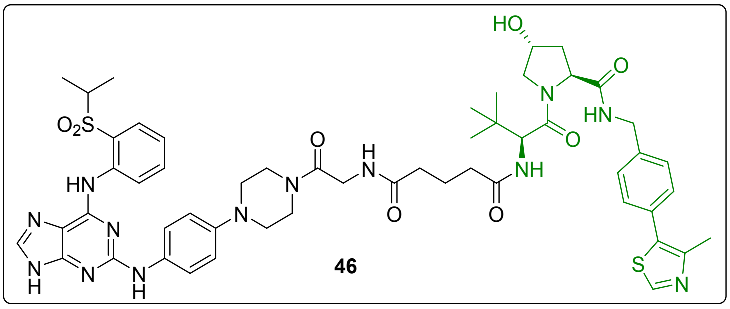

Analytical method 2

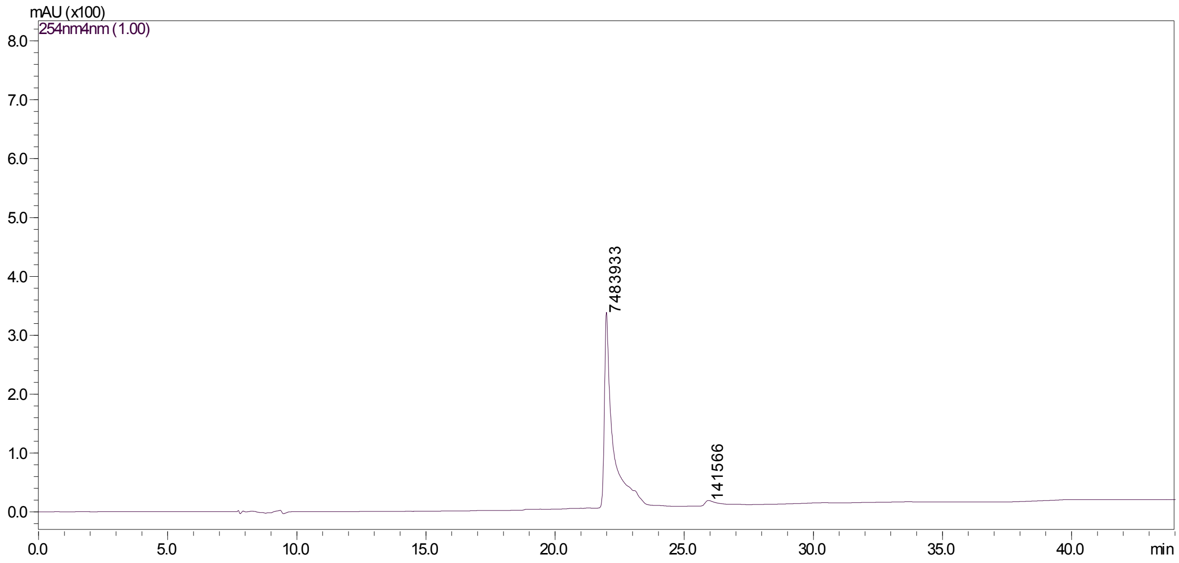

Area= 7483933

Total Area=7625499

Purity (%)=98.1%

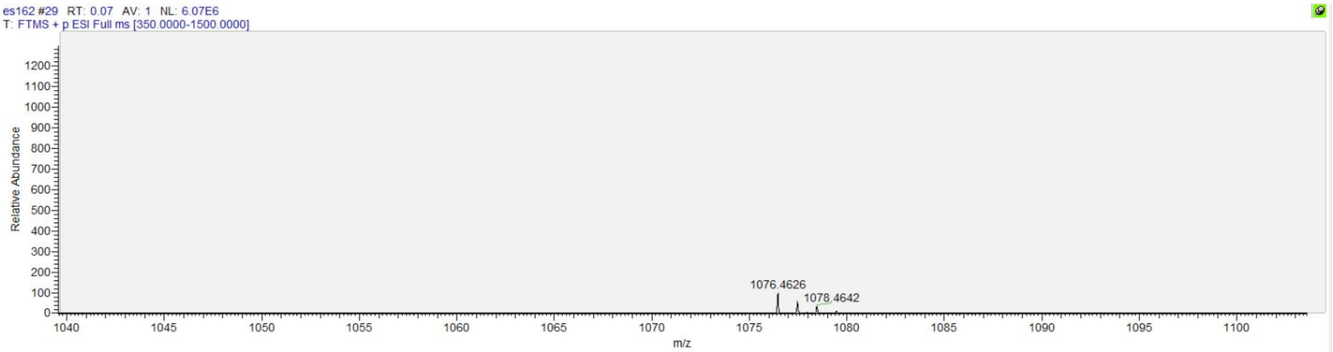

ESI-HRMS m/z for $C_{53}H_{66}N_{13}O_8S_2$ $[M+H]^+$ calcd 1076.4599, found 1076.4626.

Figure S75

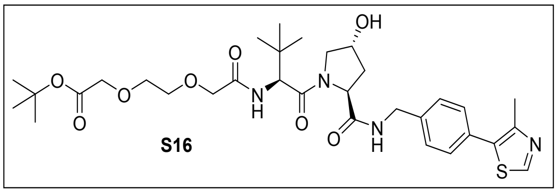
OH
O
N
H
O
O
O
O
O
N
H
O
N
S
S16

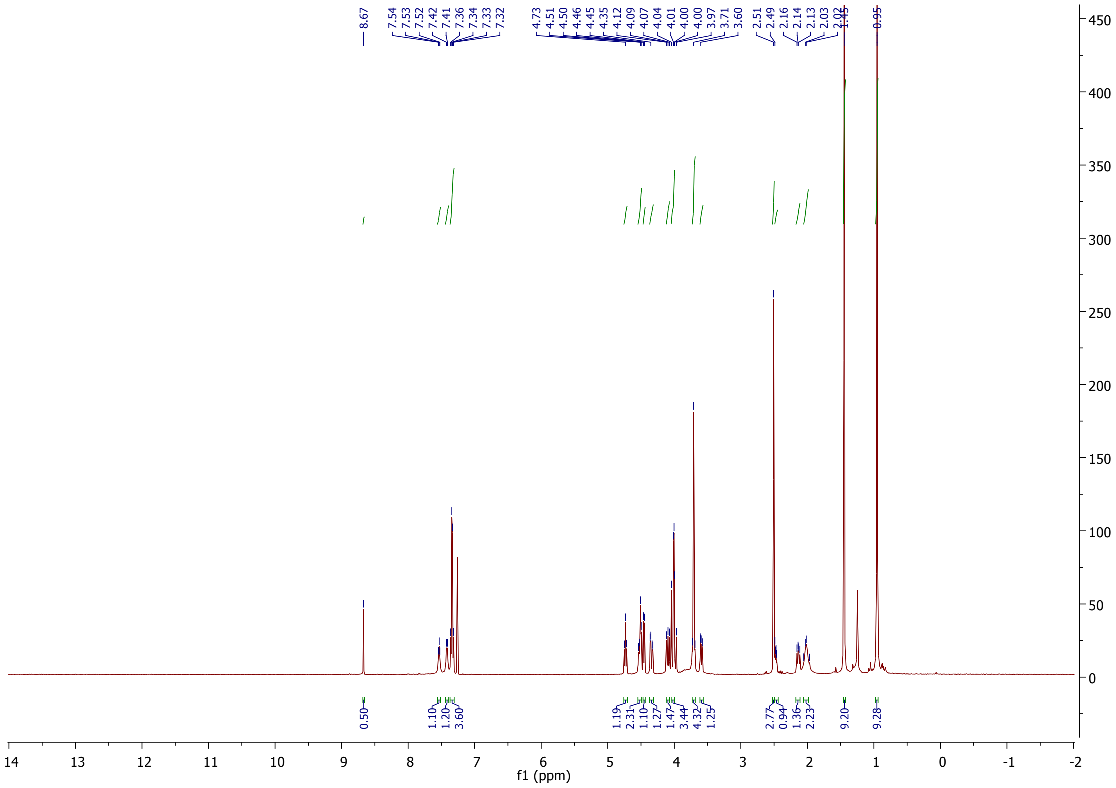
8.67
7.54
7.53
7.52
7.42
7.41
7.36
7.34
7.33
7.32
4.73
4.51
4.50
4.46
4.45
4.35
4.12
4.09
4.07
4.04
4.01
4.00
4.00
3.97
3.71
3.60
2.51
2.49
2.16
2.14
2.13
2.03
2.02
1.45
0.95
0.50
1.10
1.20
3.60
1.19
2.31
1.10
1.27
1.47
3.44
4.32
1.25
2.77
0.94
1.36
2.23
9.20
9.28
f1 (ppm)

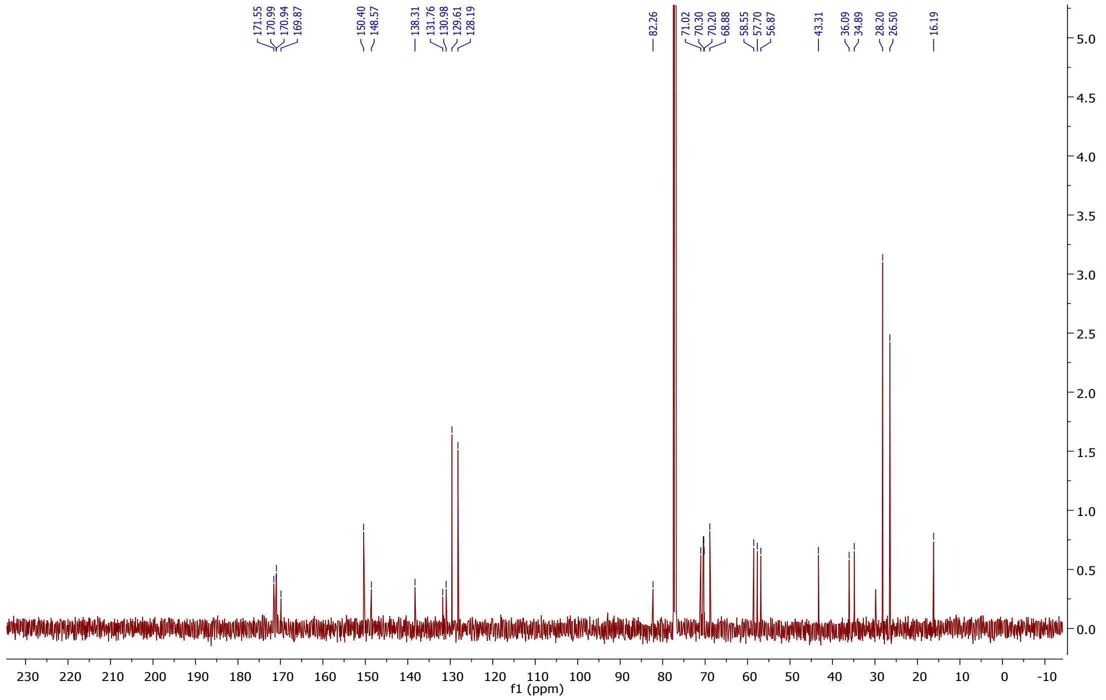
171.55
170.99
170.94
169.87
150.40
148.57
138.31
131.76
130.98
129.61
128.19
82.26
71.02
70.30
70.20
68.88
58.55
57.70
56.87
43.31
36.09
34.89
28.20
26.50
16.19
f1 (ppm)

Figure S76

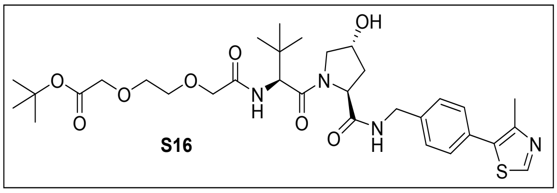

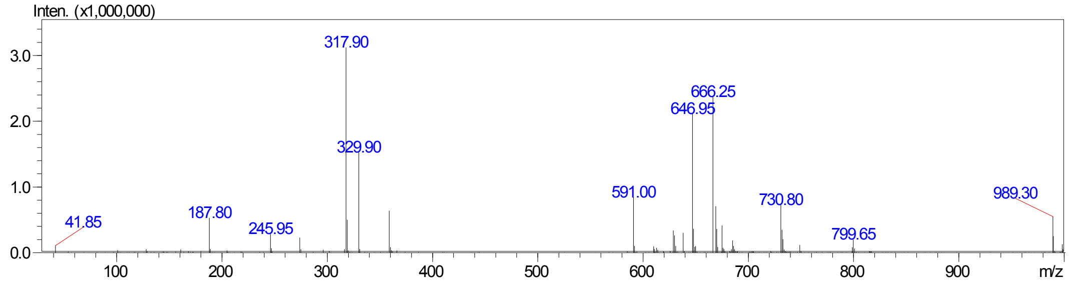

Figure S77. ESI-MS m/z for $C_{32}H_{47}N_4O_8S$ $[M+H]^+$ calculated 647.3, found 646.95.

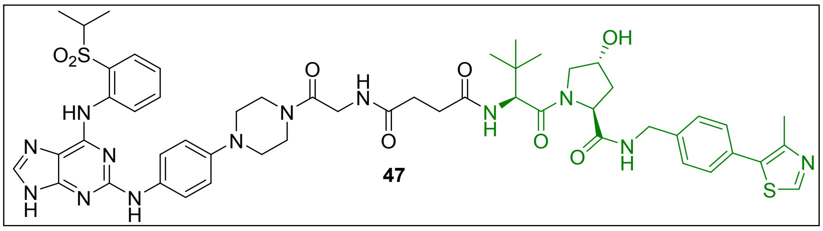

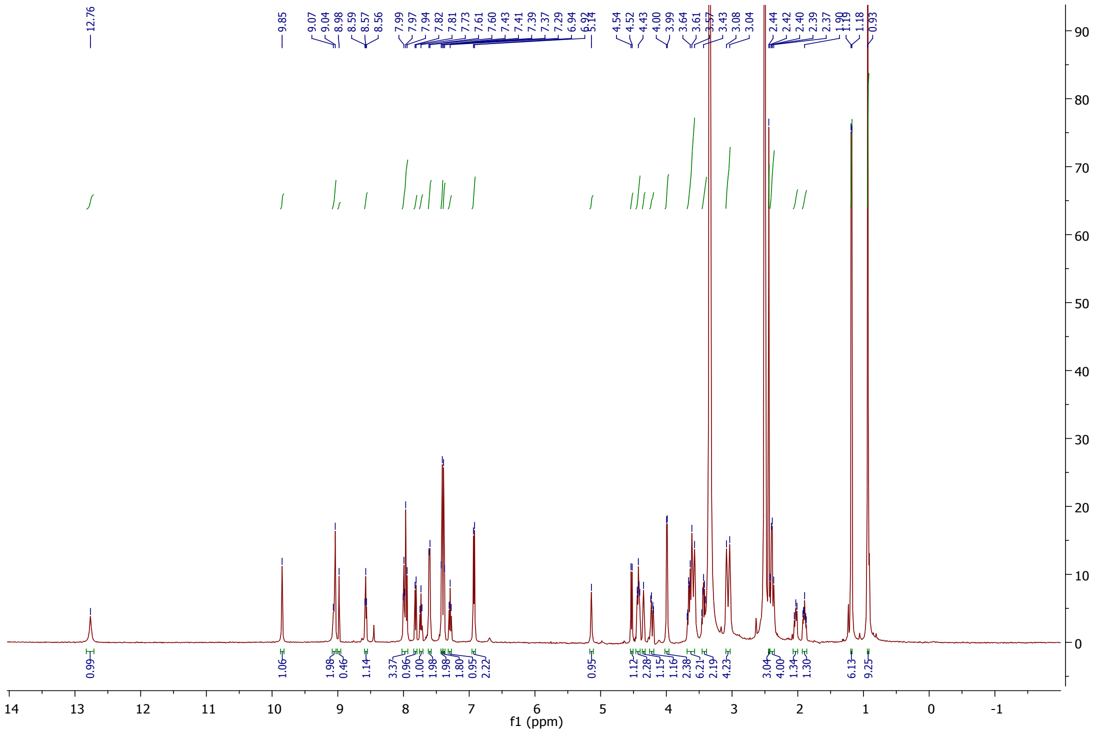

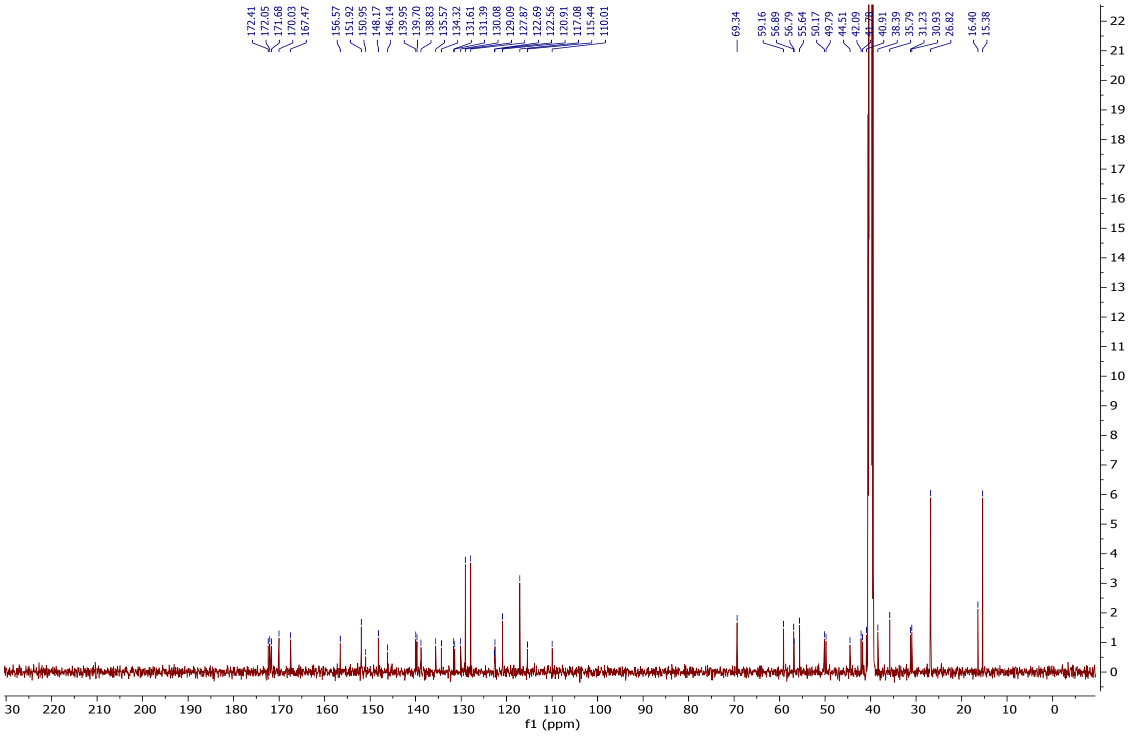

Figure S78

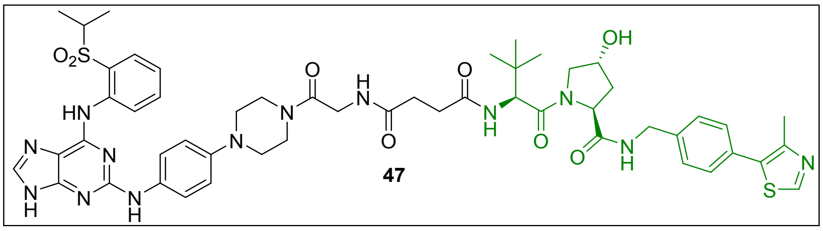

47

Analytical method 2

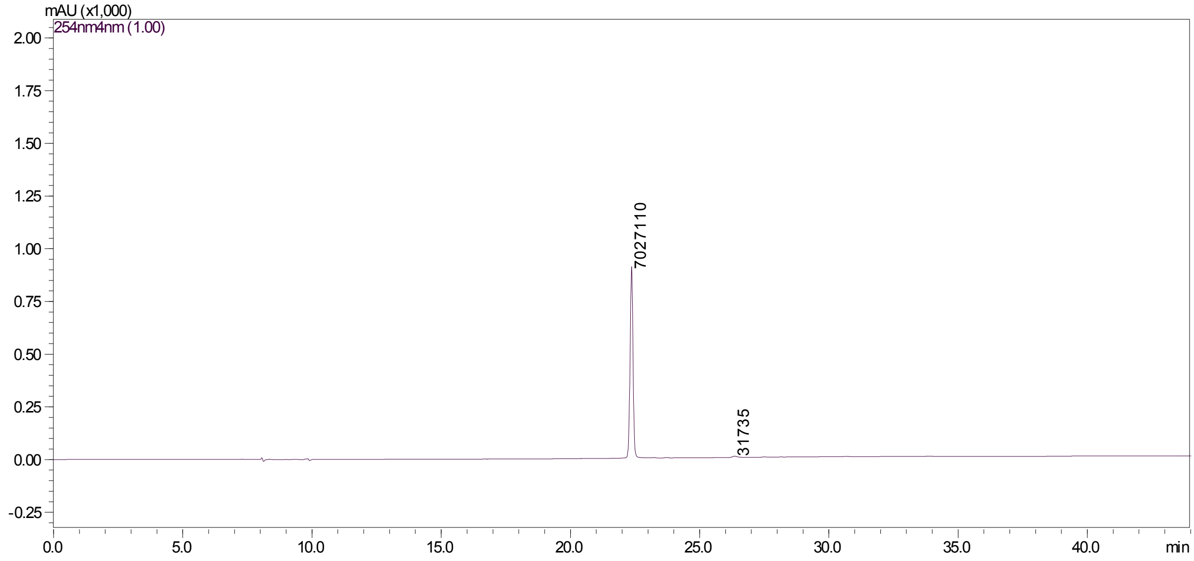

Total area=7058845

Area=7027110

Purity (%)=99.5%

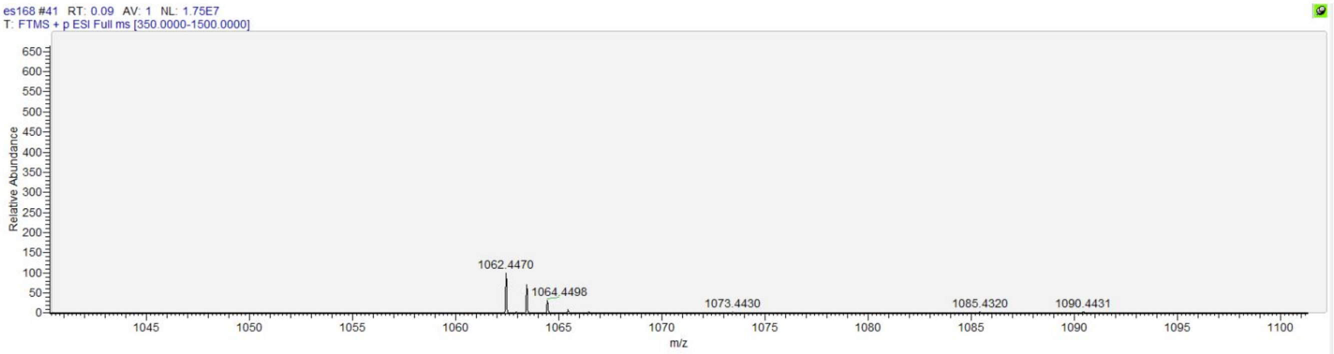

ESI-HRMS m/z for $C_{52}H_{64}N_{13}O_8S_2$ $[M+H]^+$ calcd 1062.4442, found 1062.4470.

Figure S79

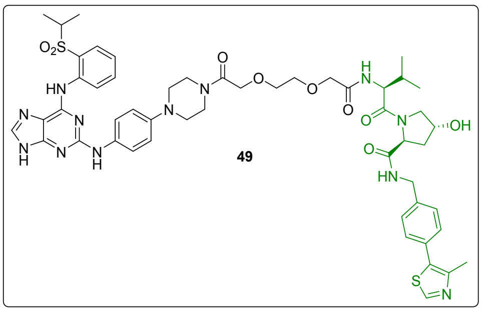
49

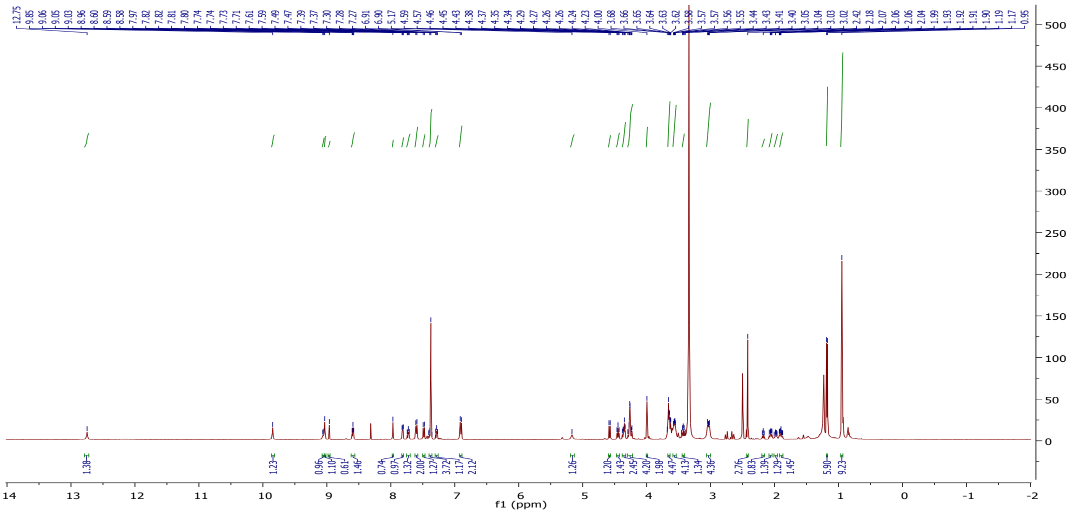
f1 (ppm)

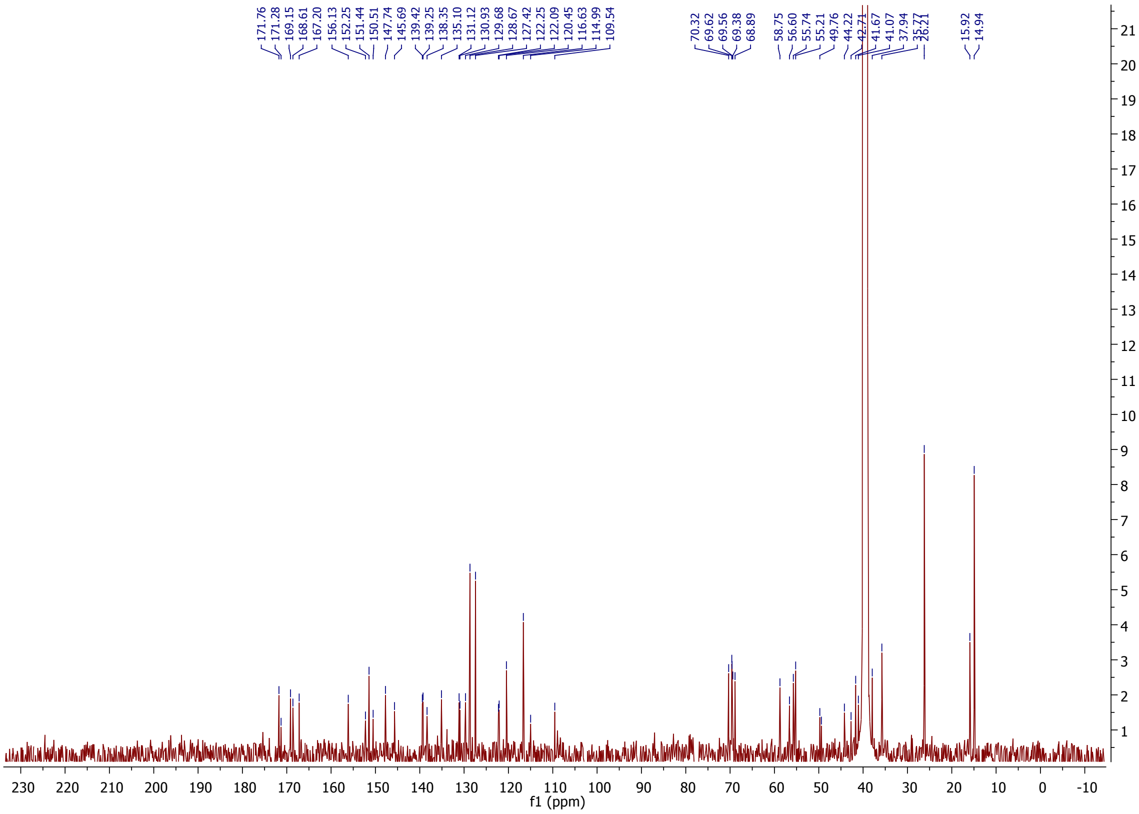
171.76
171.28
169.15
168.61
167.20
156.13
152.25
151.44
150.51
147.74
145.69
139.42
139.25
138.35
135.10
131.12
130.93
129.68
128.67
127.42
122.25
122.09
120.45
116.63
114.99
109.54
70.32
69.62
69.56
69.38
68.89
58.75
56.60
55.74
55.21
49.76
44.22
41.67
41.07
37.94
35.77
26.21
15.92
14.94
f1 (ppm)

Figure S80

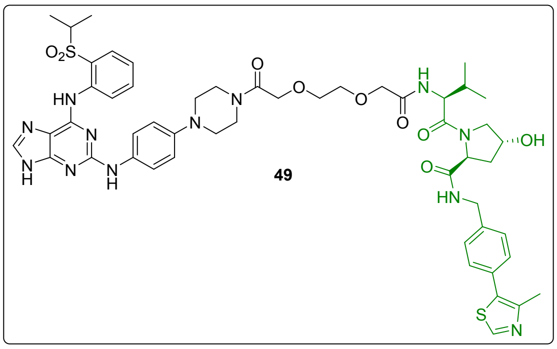

Analytical method 2

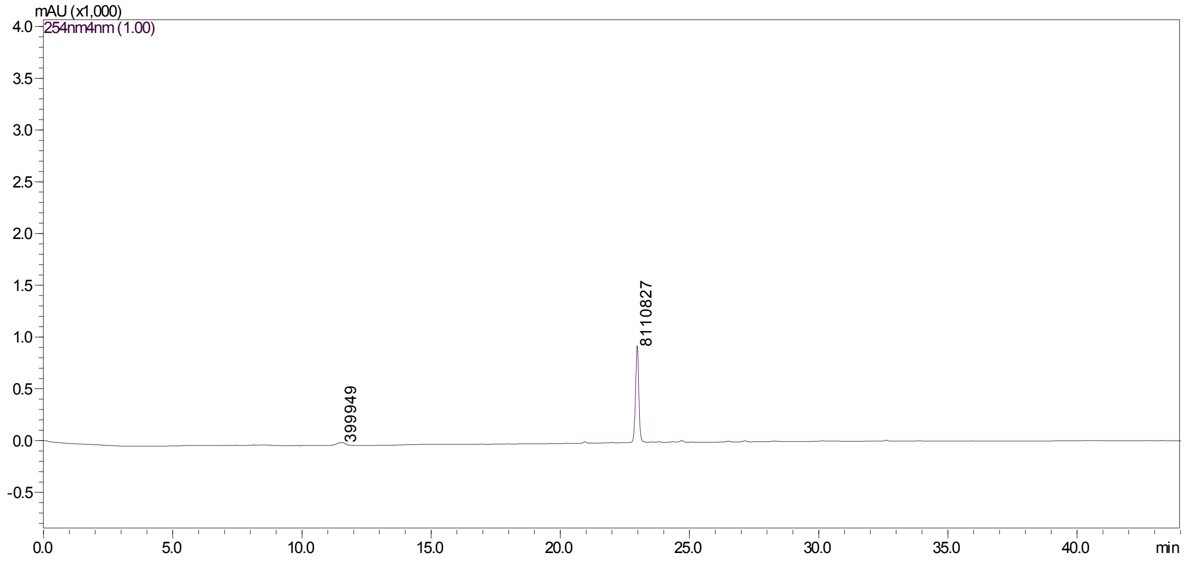

Area= 8110827

Total Area= 8510776

Purity (%)= 95,3%

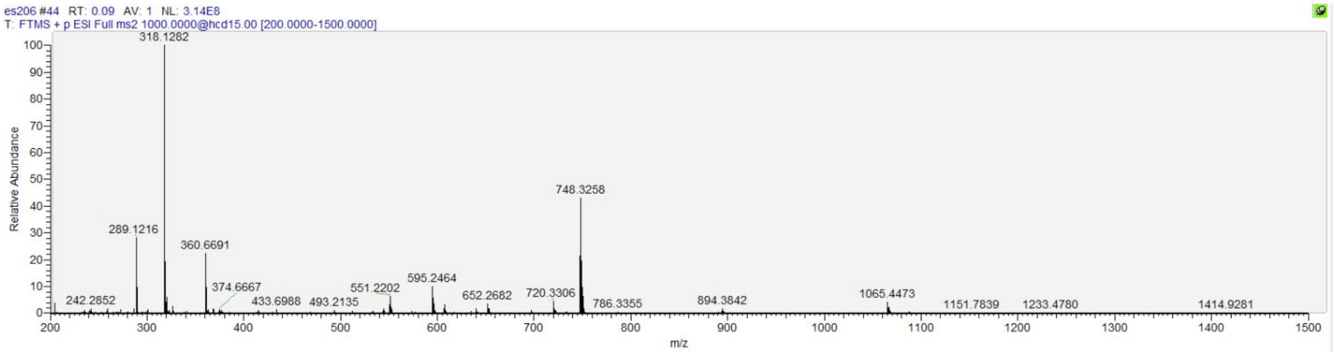

ESI-HRMS m/z for $C_{52}H_{65}N_{12}O_9S_2$ $[M+H]^+$ calcd 1065.4433, found 1065.4473.

Figure S81

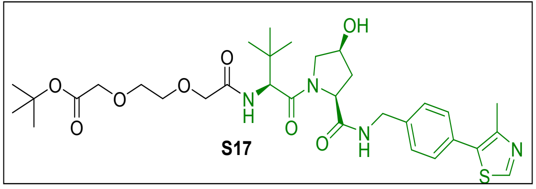

S17

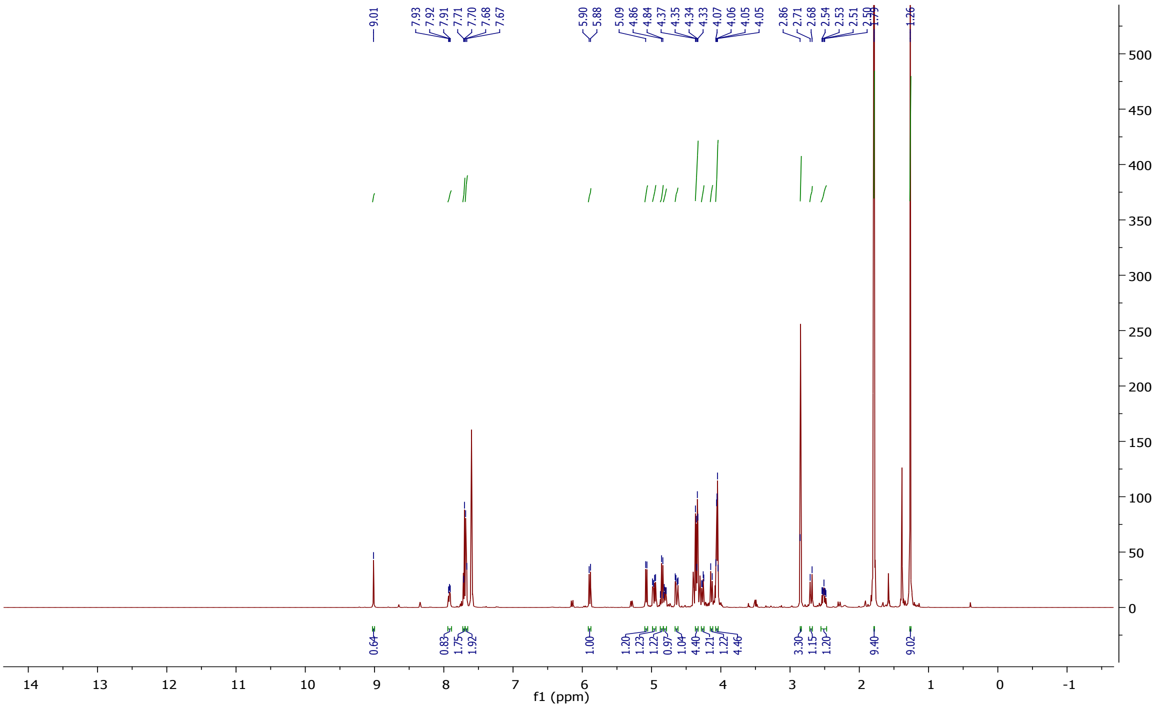

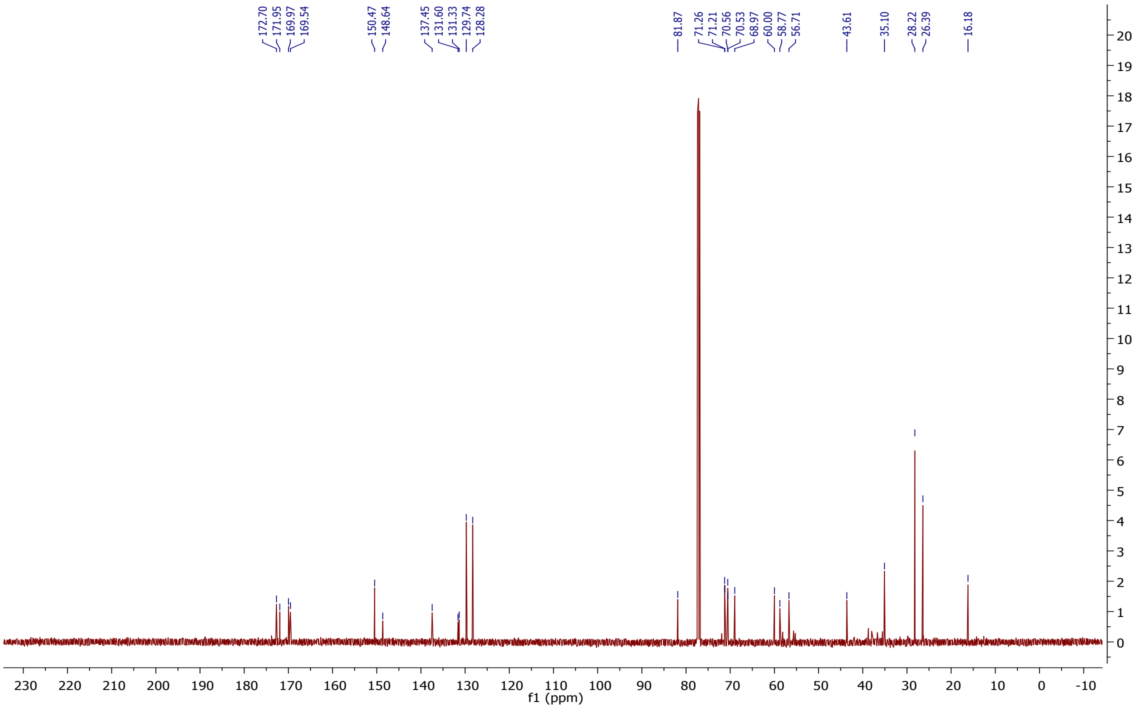

Figure S82

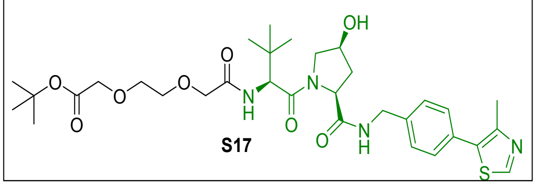

S17

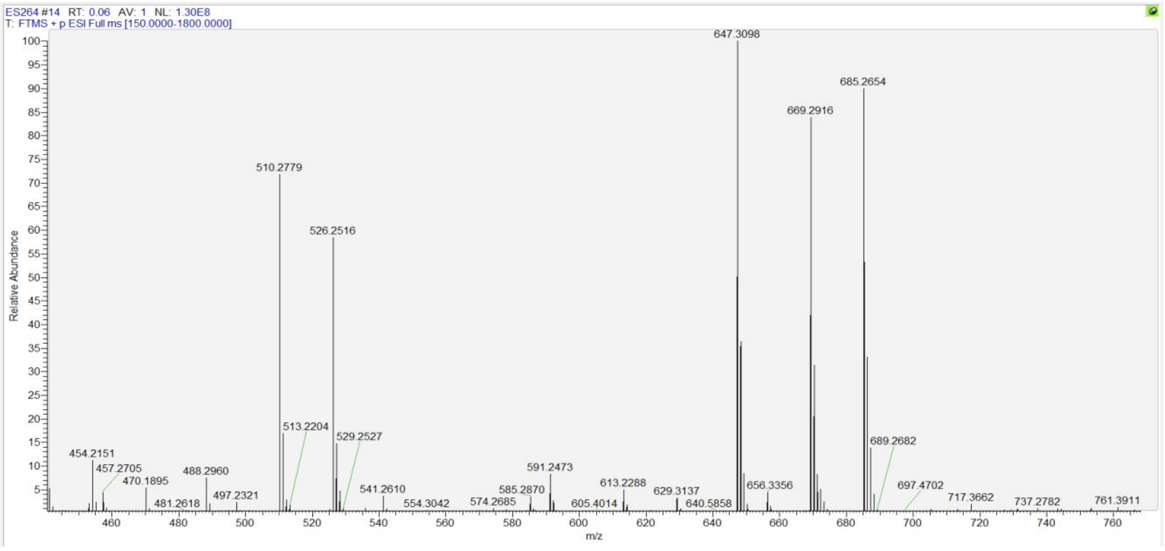

Figure S83. ESI-HRMS m/z for $C_{32}H_{47}N_4O_8S$ $[M+H]^+$ calcd 647.3115, found 647.3098.

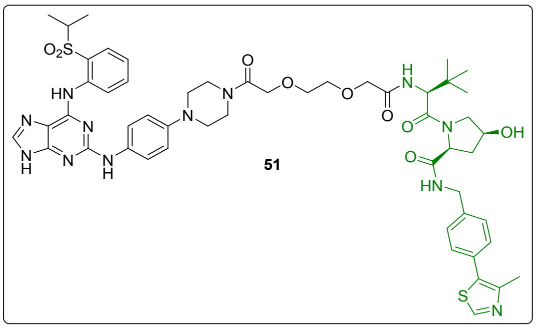

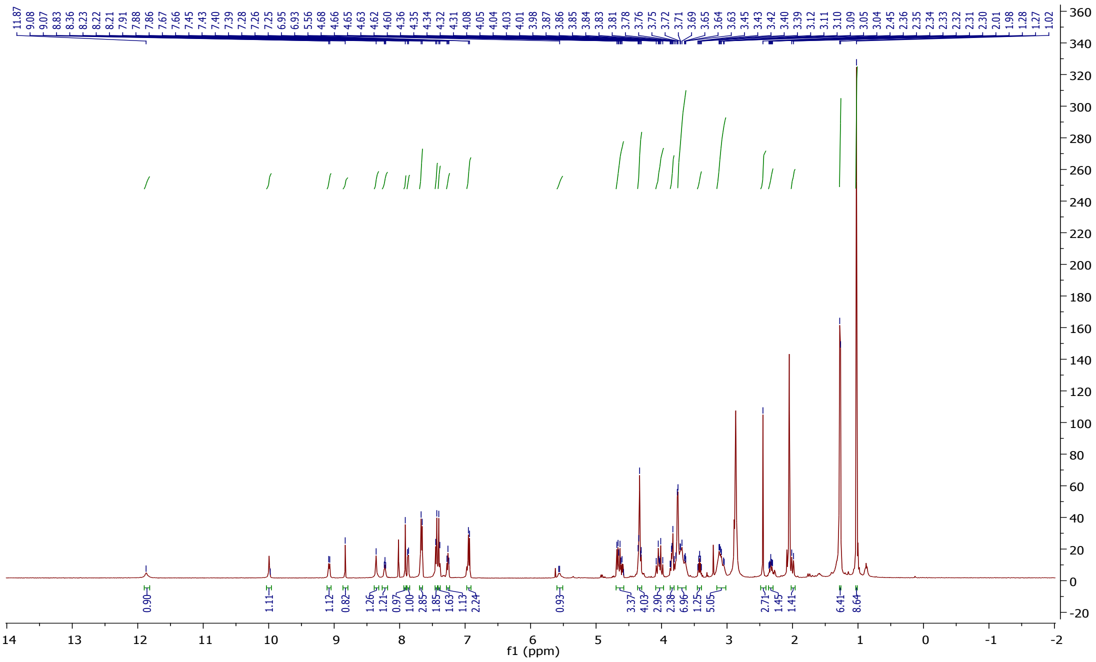

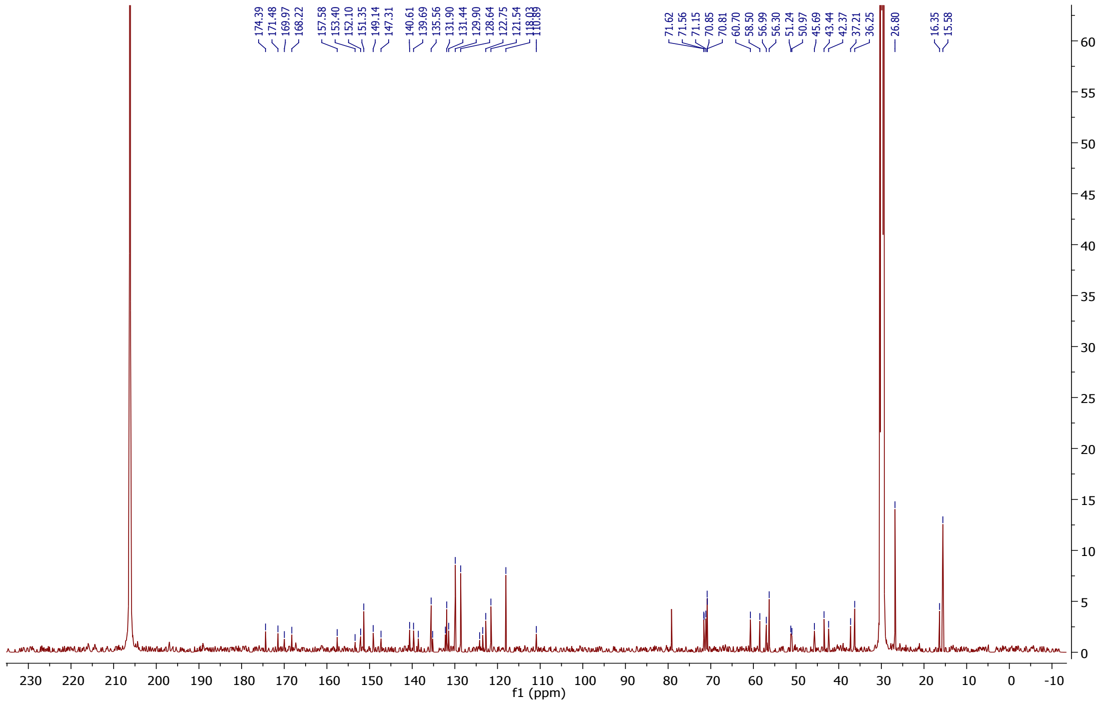

Figure S84

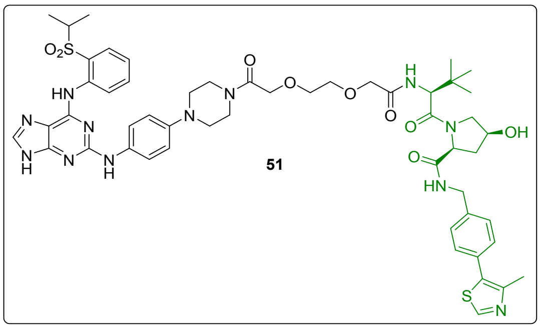

Analytical method 2

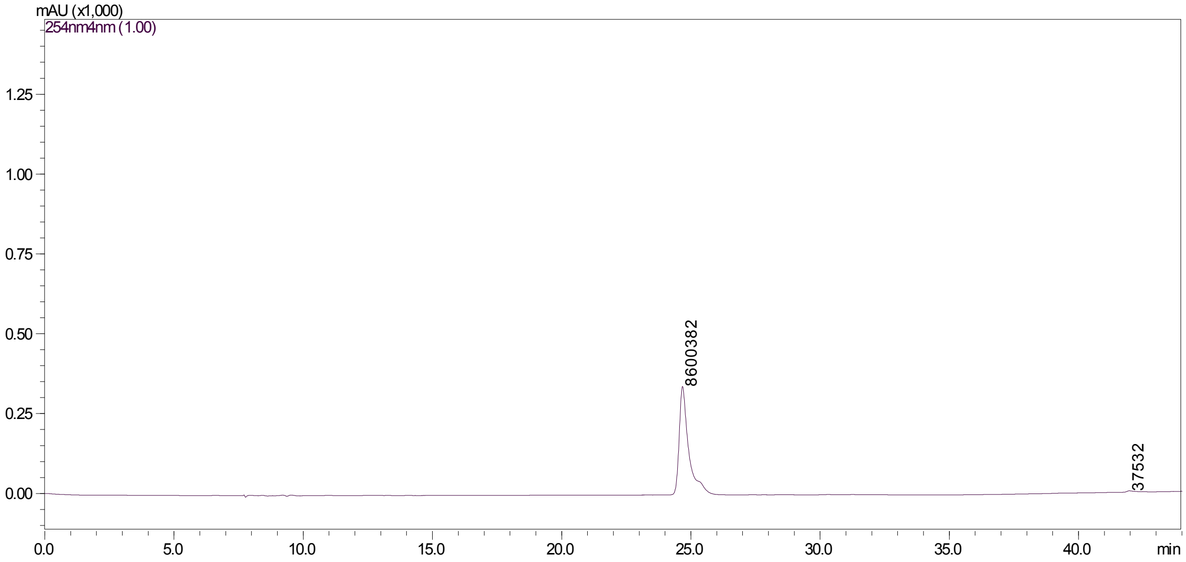

Area: 8600382

Total area: 8637914

Purity (%)= 99.5%

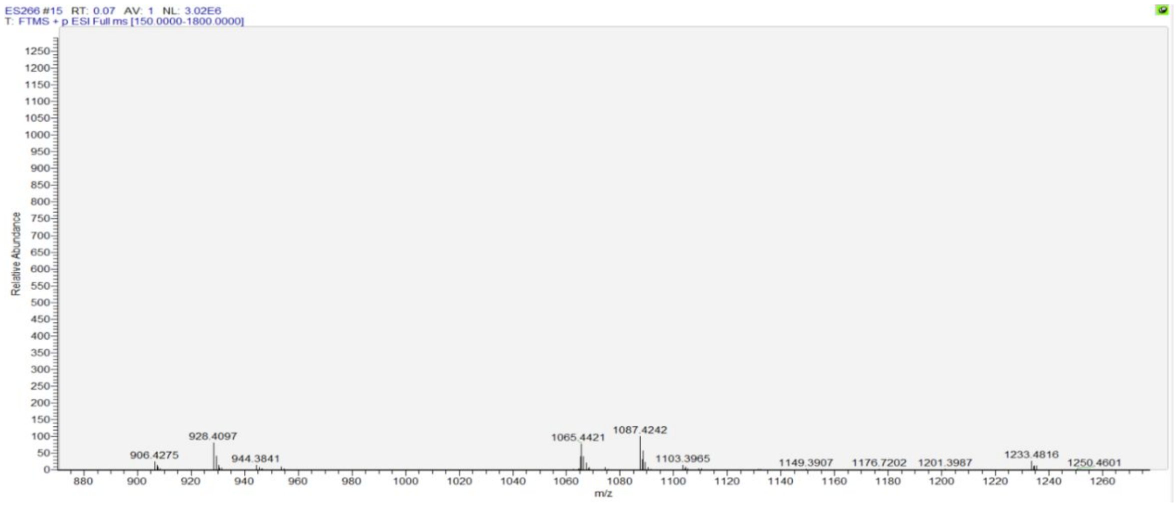

ESI-HRMS m/z for $C_{52}H_{65}N_{12}O_9S_2$ $[M+H]^+$ calcd 1065.4439, found 1065.4421.

Figure S85

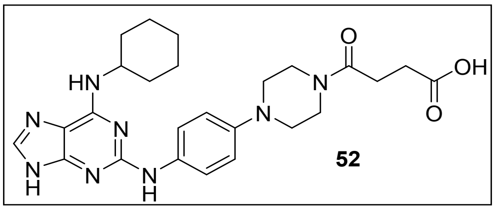
HN
N
N
N
N
H
N
H
N
N
O
OH
O
52

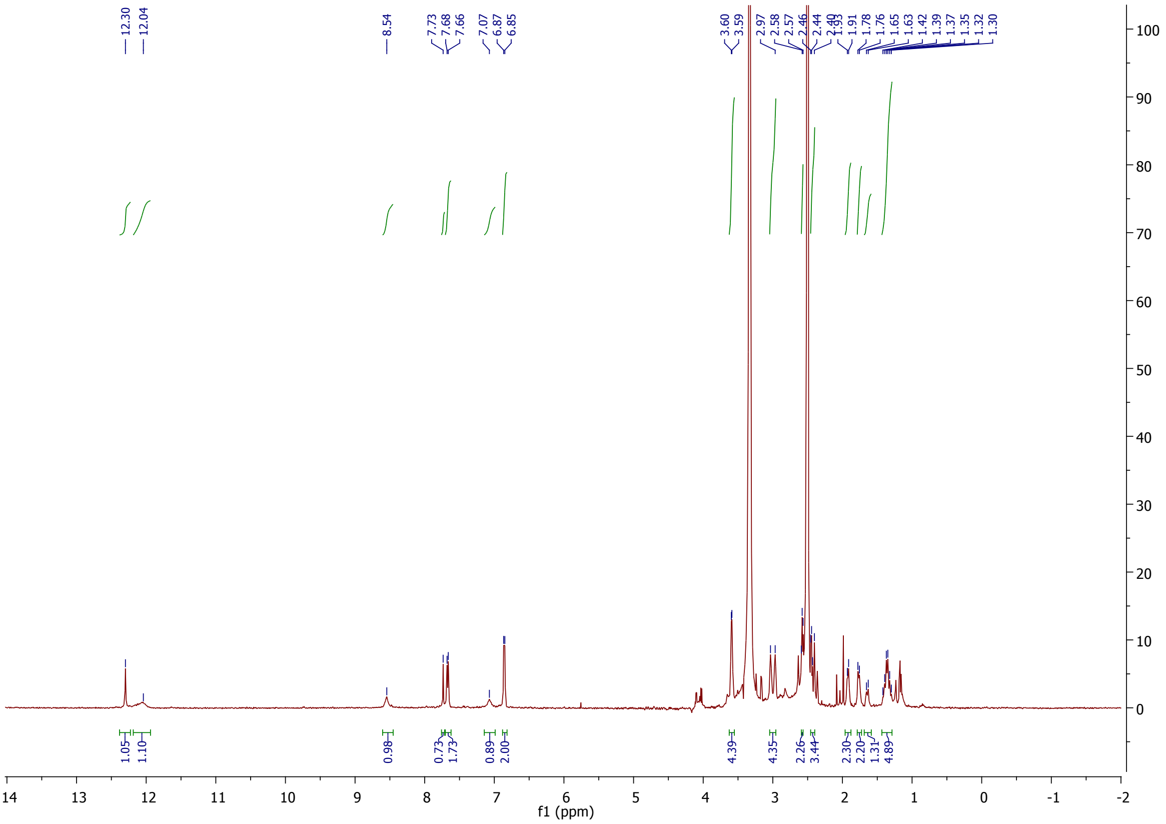
12.30
12.04
8.54
7.73
7.68
7.66
7.07
6.87
6.85
3.60
3.59
2.97
2.58
2.57
2.46
2.44
1.91
1.78
1.76
1.65
1.63
1.42
1.39
1.37
1.35
1.32
1.30
1.05
1.10
0.98
0.73
1.73
0.89
2.00
4.39
4.35
2.26
3.44
2.30
2.20
1.31
4.89
f1 (ppm)

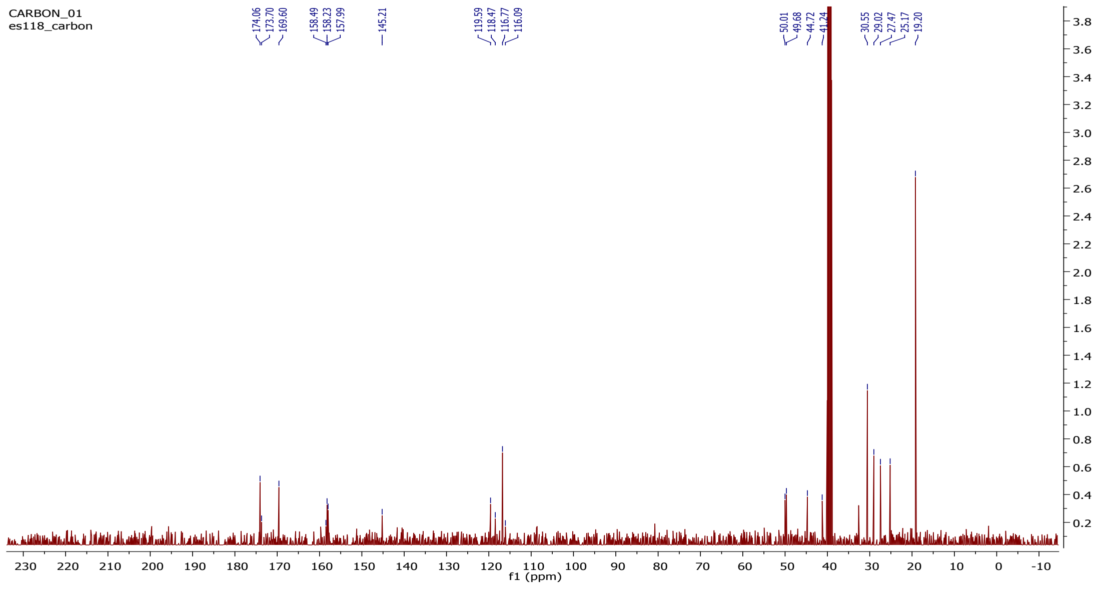
CARBON_01
es118_carbon
174.06
173.70
169.60
158.49
158.23
157.99
145.21
119.59
118.47
116.77
116.09
50.01
49.68
44.72
41.24
30.55
29.02
27.47
25.17
19.20
f1 (ppm)

Figure S86

Compound 52

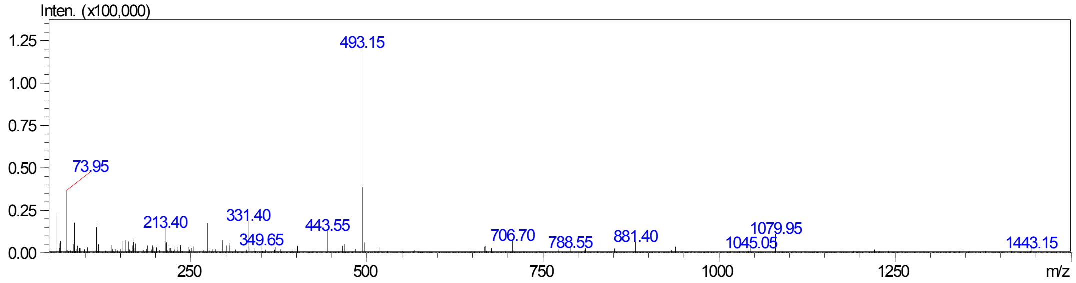

Figure S87.ESI-MS m/z for $C_{25}H_{33}N_8O_3$ $[M+H]^+$ calcd 493.26, found 493.15.

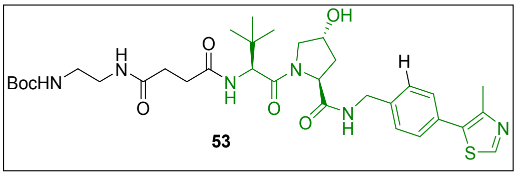

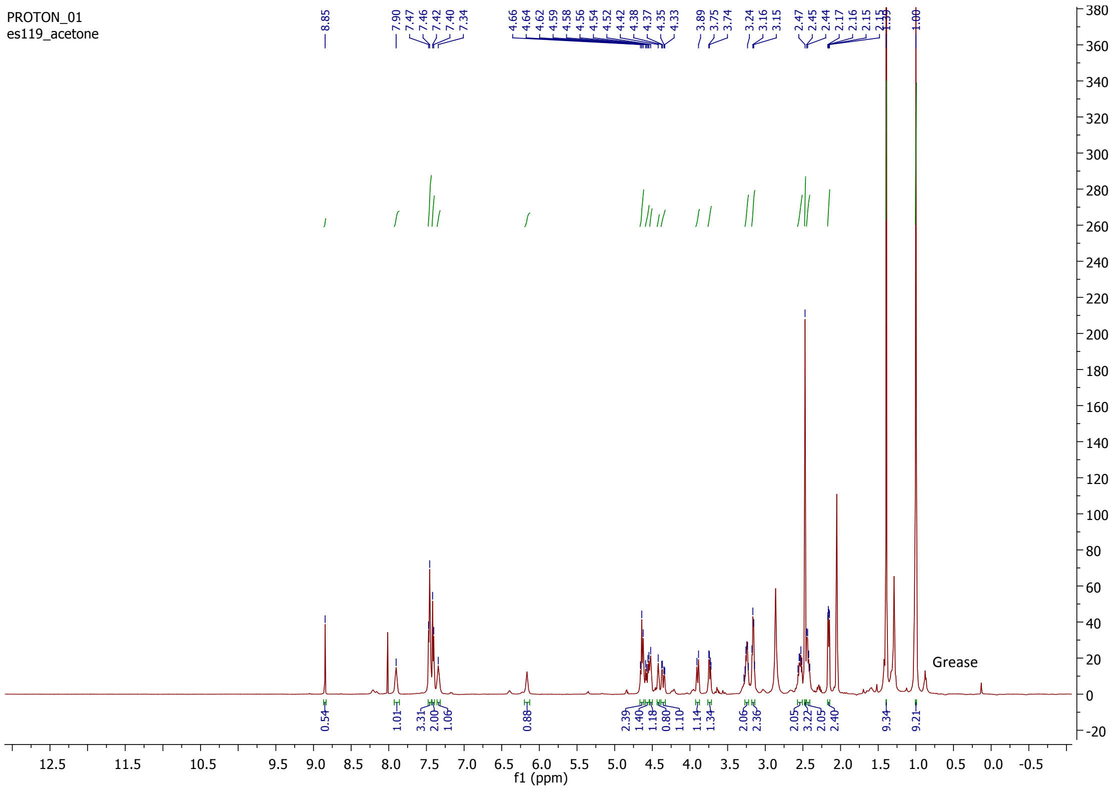

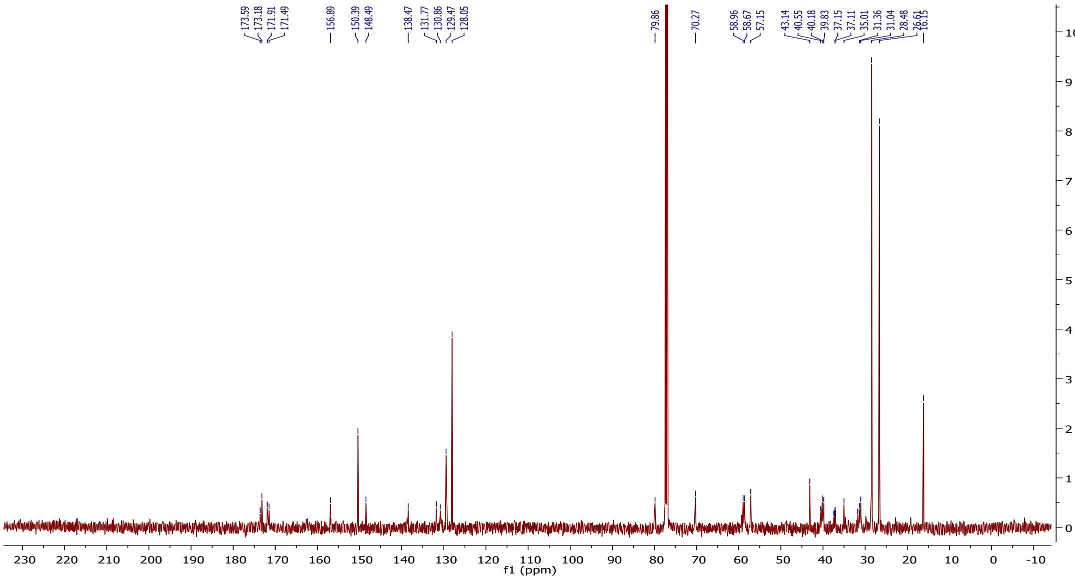

Figure S88

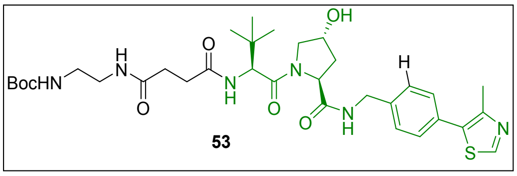

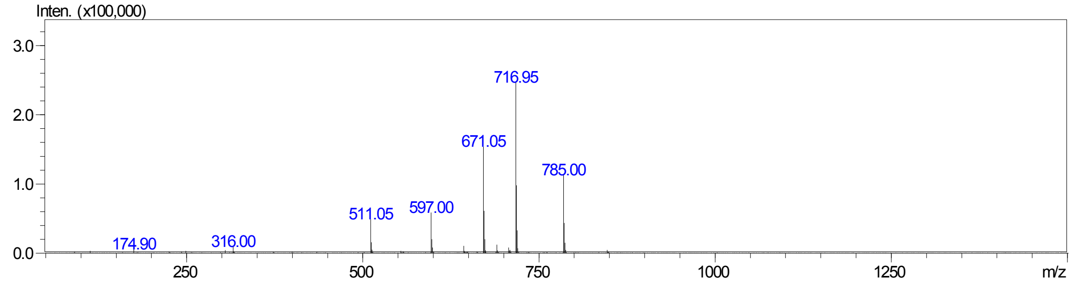

Figure S89. ESI-MS m/z for $C_{33}H_{47}N_6O_7S$ [M-H]$^-$ calcd 671.32, found 671.05.

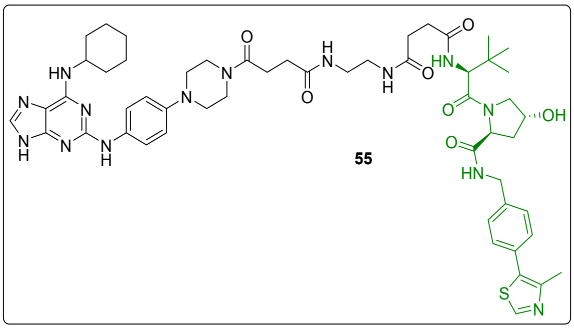

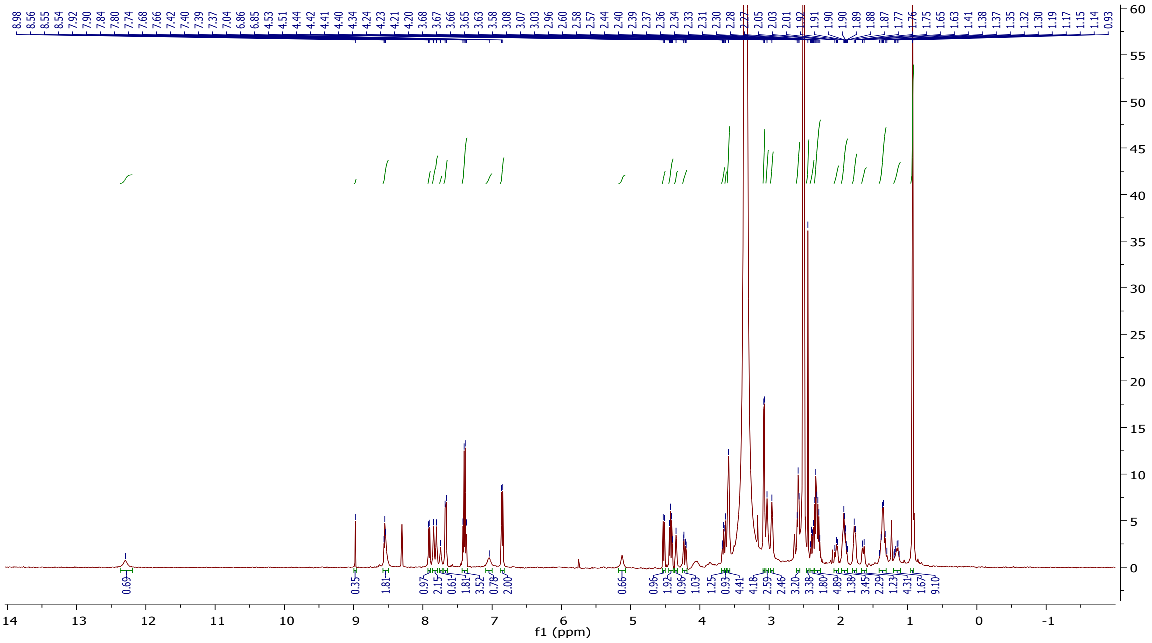

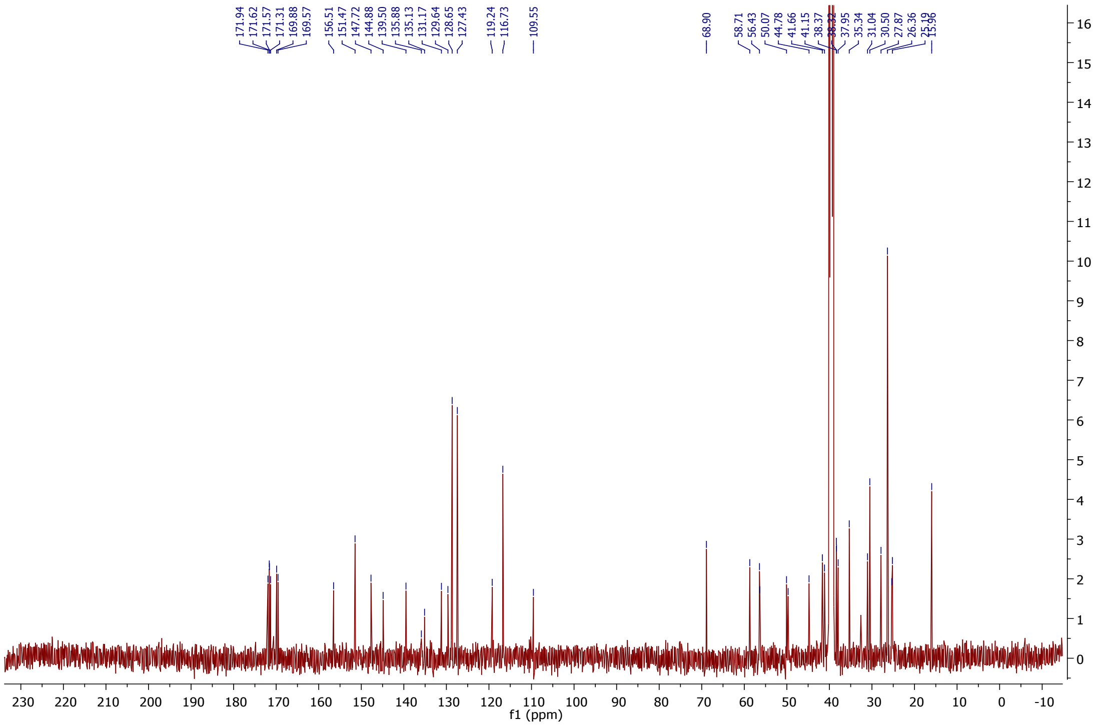

Figure S90

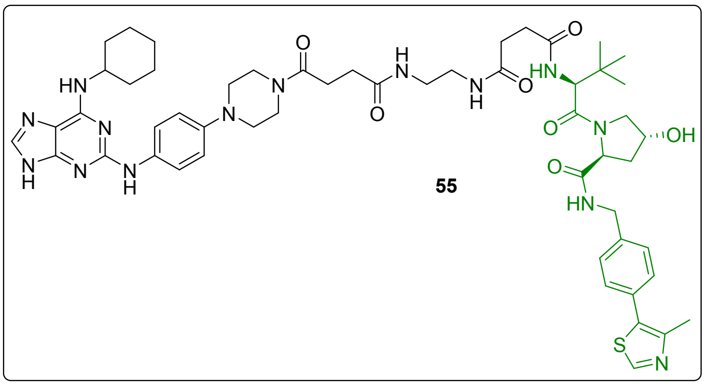

Analytical method 1

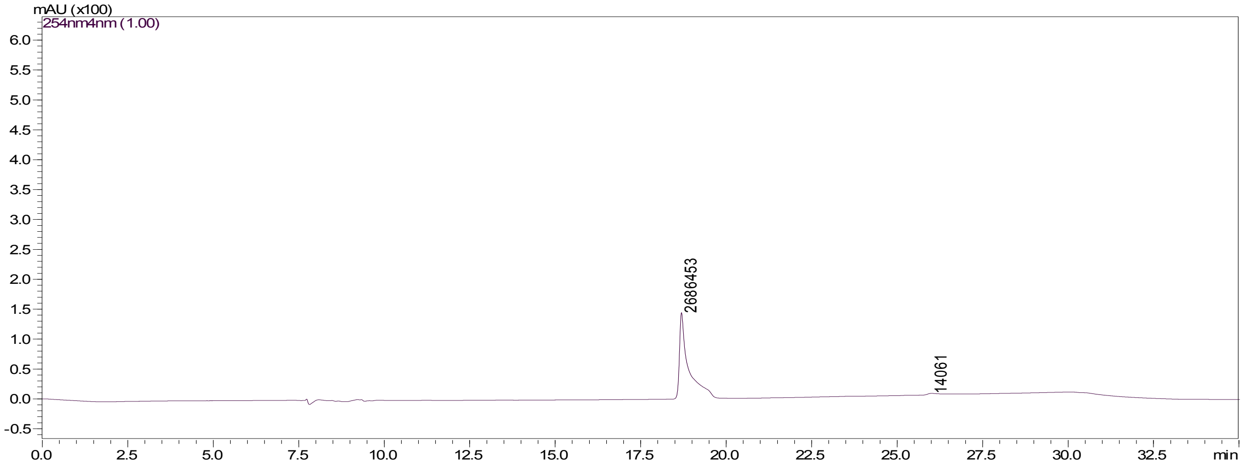

Area= 2686453

Total Area= 2700514

Purity (%)=99.5%

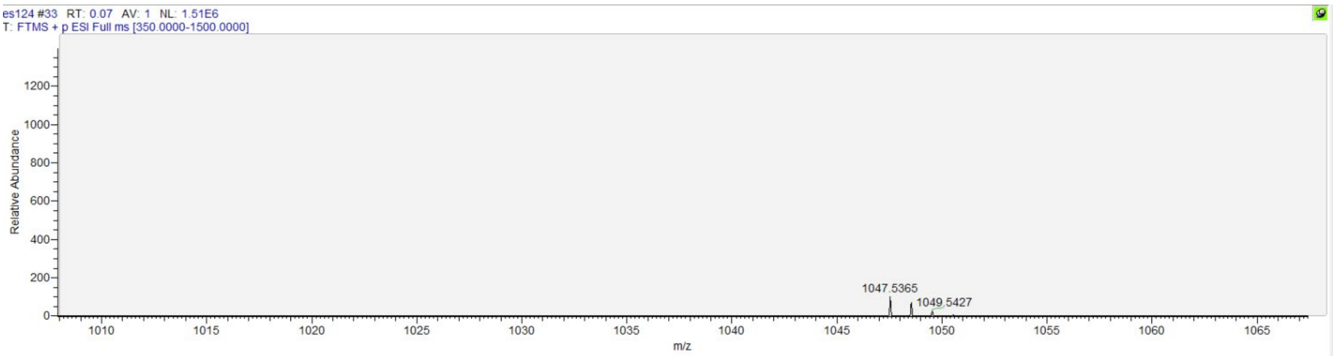

ESI-HRMS m/z for $C_{53}H_{71}N_{14}O_7S$ $[M+H]^+$ calcd 1047.5345, found 1047.5365.

Figure S91

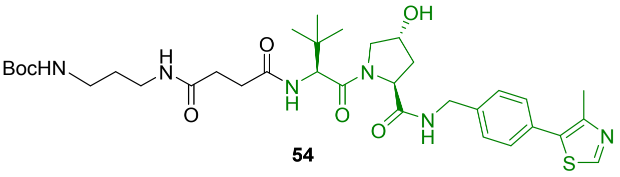

54

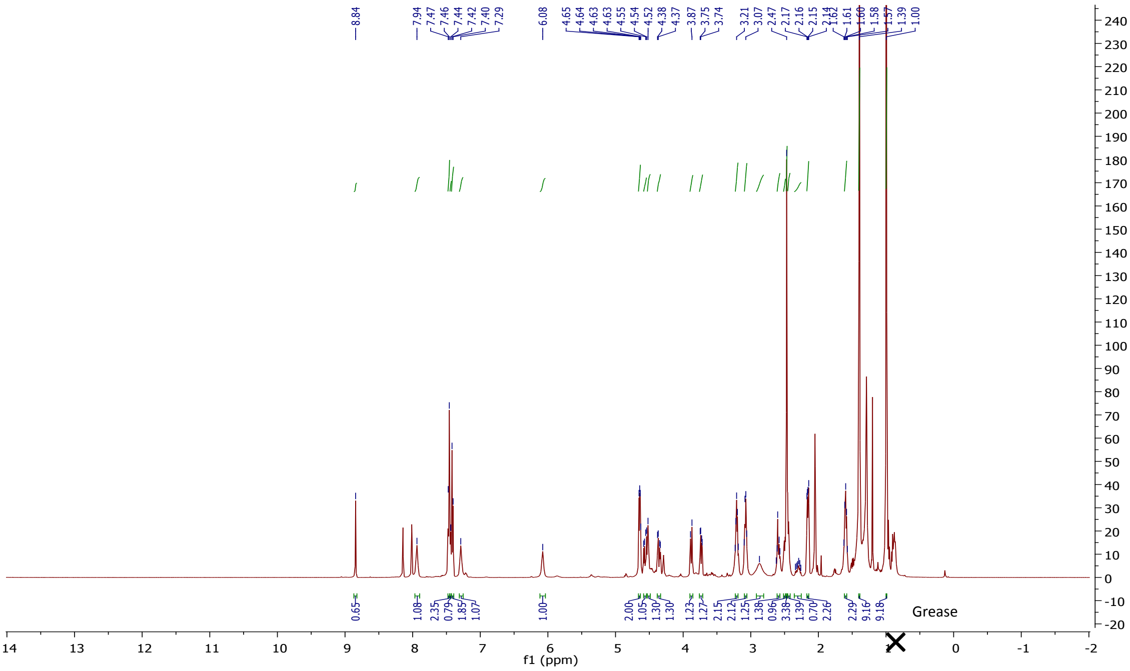

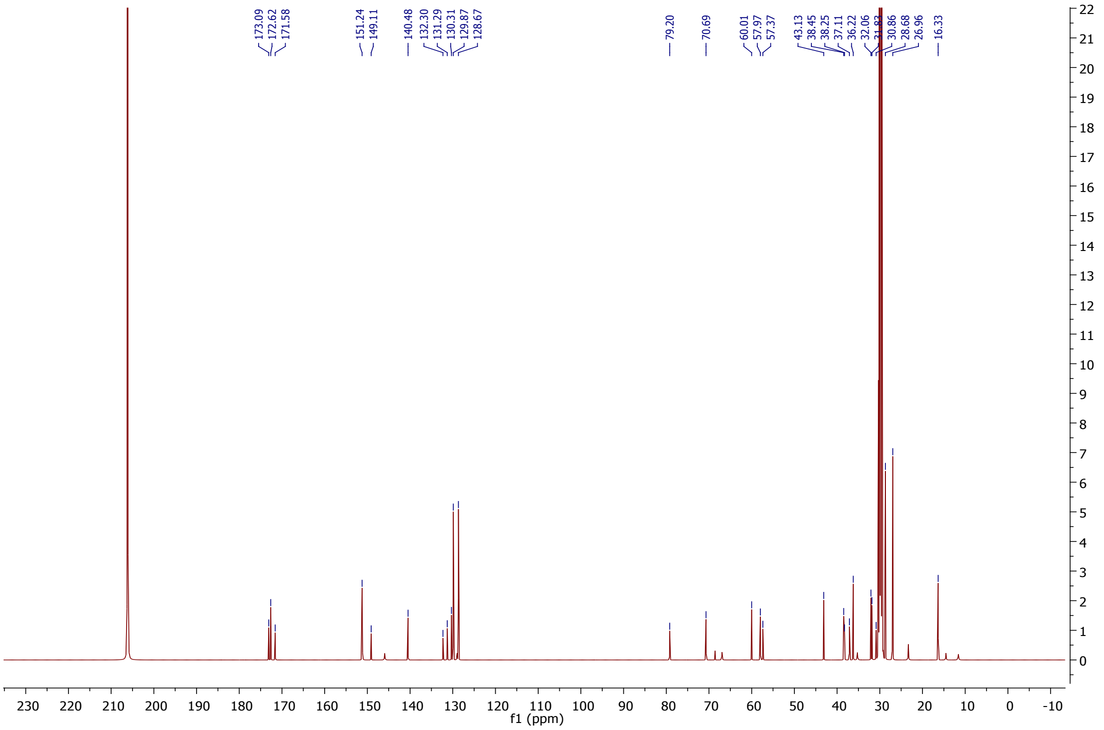

Figure S92

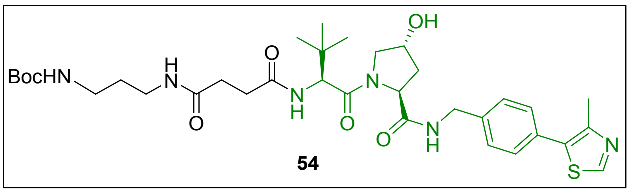

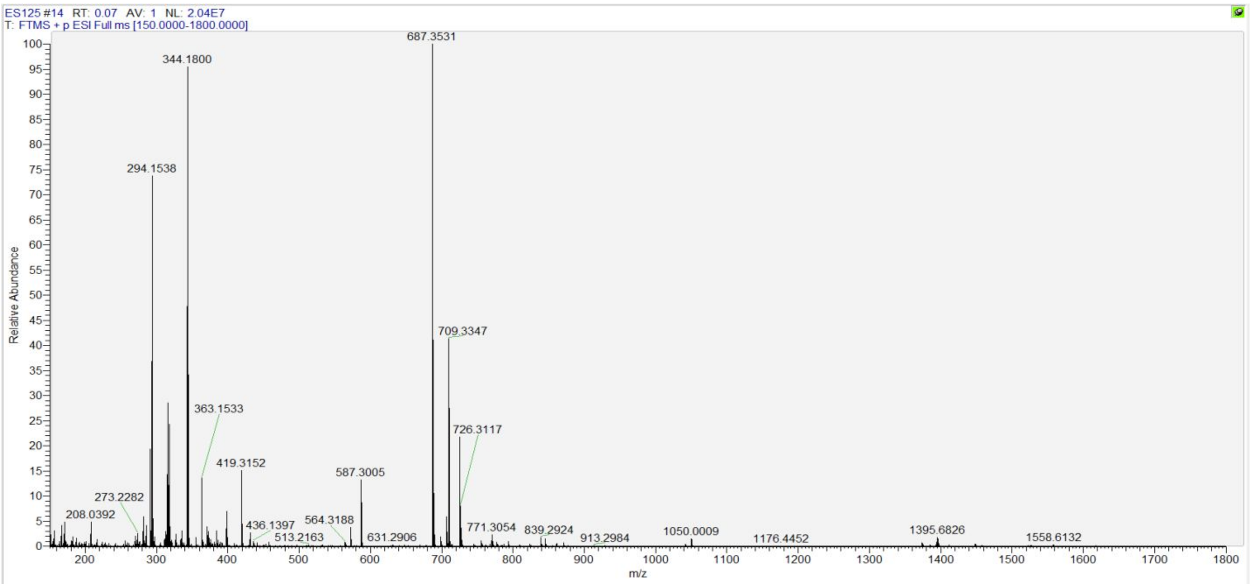

Figure S93. ESI-HRMS m/z for $C_{34}H_{51}N_6O_7S$ $[M+H]^+$ calcd 687.3540, found 687.3531.

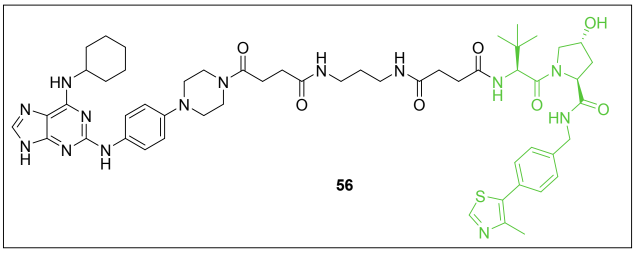

56

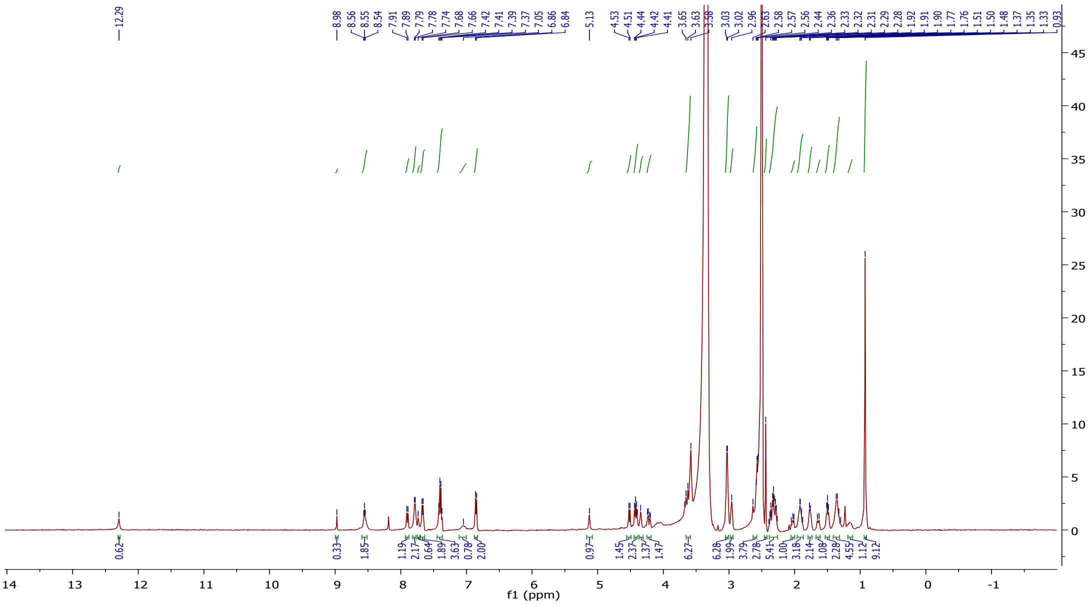

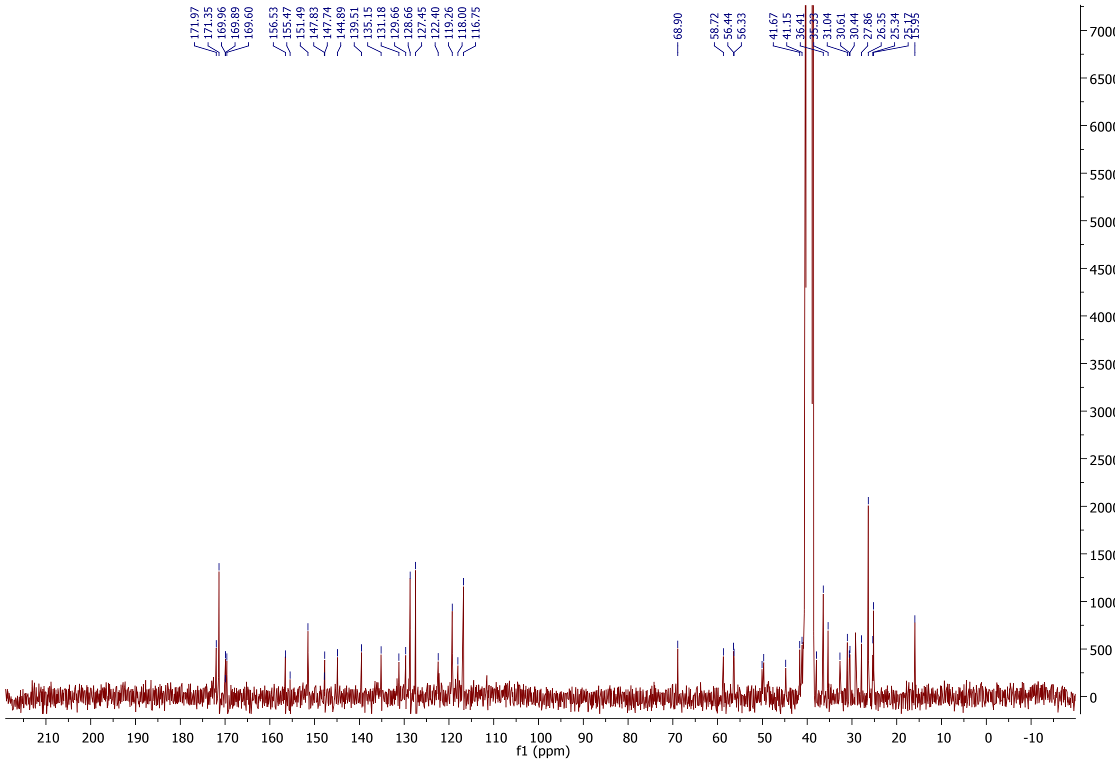

Figure S94

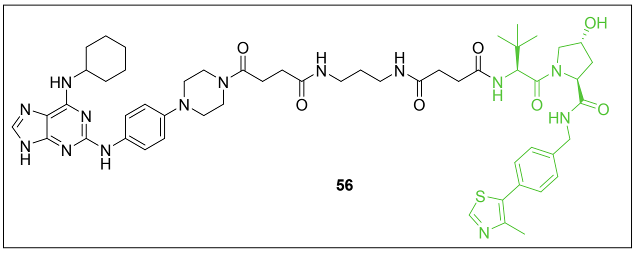

Analytical method 2

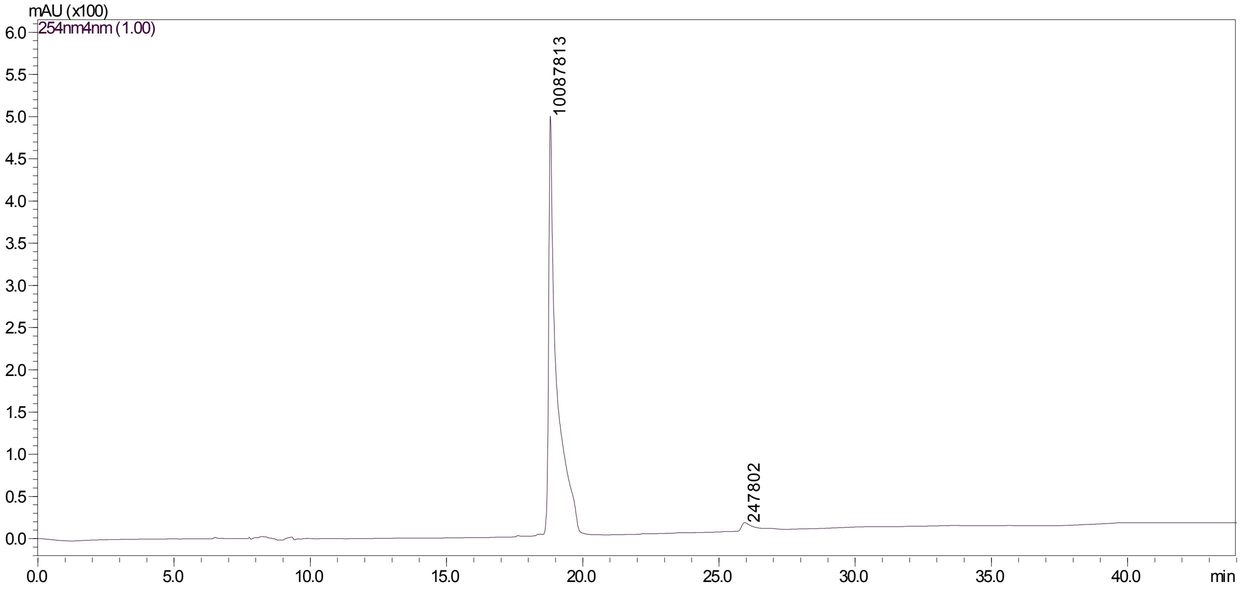

Area= 10087813

Total Area= 10335615

Purity (%)=97.6%

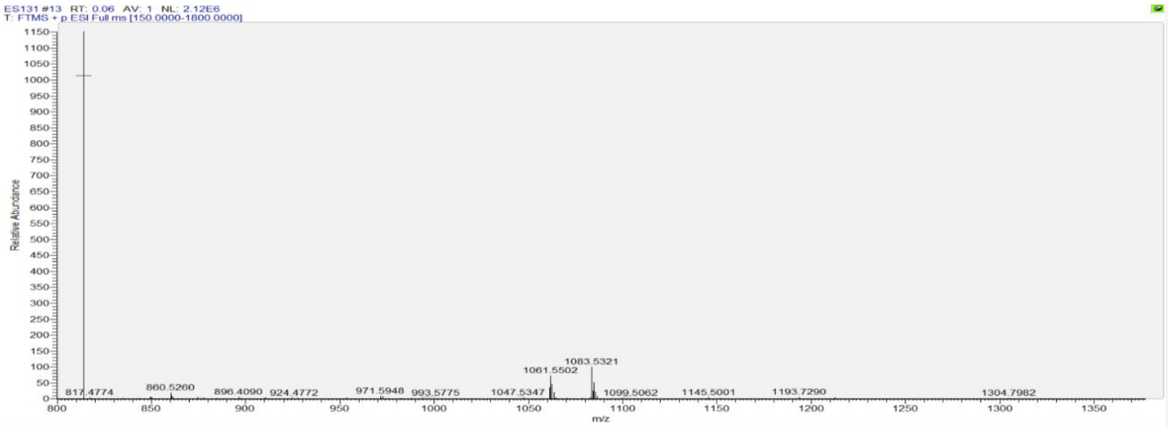

ESI-HRMS m/z for $C_{54}H_{73}N_{14}O_7S$ $[M+H]^+$ calcd 1061.5507, found 1061.5502.

Figure S95

Analytical methods for LC-MS / HPLC

Analytical method 1

LC/ESI-MS: LC20AD Shimadzu connected to Shimadzu LCMS-2010EV
Mobile Phase A: 0.1% formic acid in water
Mobile Phase B: 0.1% formic acid in acetonitrile
Column: SUPELCO Discovery (C18, 25 cm × 4.6 mm, 5 µm)
Flow rate: 0,4 mL/min
Run time: 35 min
Column temperature: 26 °C
UV detector: 254 nm MS detector: 1.65Kv

Table S1

Time (min)	H_2O (% v/v conc.)	ACN (% v/v conc.)
0	90	10
3	90	10
22	15	85
25	60	40
29	70	30
30	90	10

Analytical method 2

LC/ESI-MS: LC20AD Shimadzu connected to Shimadzu LCMS-2010EV
Mobile Phase A: 0.1% FA in water
Mobile Phase B: 0.1% FA in acetonitrile
Column: SUPELCO Discovery (C18, 25 cm × 4.6 mm, 5 µm)
Flow rate: 0,4 mL/min
Run time: 44 min
Column temperature: 26 °C
UV detector: 254 nm MS detector: 1.65kV

Table S2

Time (min)	H_2O (% v/v conc.)	ACN (% v/v conc.)
0	90	10
3	90	10
22	15	85
25	10	90
29	10	90
31	5	95
35	5	95
39	90	10

References

[1] Tannous BA, Kerami M, Van der Stoop PM, Kwiatkowski N, Wang J, Zhou W, Kessler AF, Lewandrowski G, Hiddingh L, Sol N, Lagerweij T, Wedekind L, Niers JM, Barazas M, Nilsson RJ, Geerts D, De Witt Hamer PC, Hagemann C, Vandertop WP, Van Tellingen O, Noske DP, Gray NS, Würdinger T. Effects of the selective MPS1 inhibitor MPS1-IN-3 on glioblastoma sensitivity to antimitotic drugs. J Natl Cancer Inst. 2013;105(17):1322-31.

[2] Lu J, Huang Y, Huang J, He R, Huang M, Lu X, Xu Y, Zhou F, Zhang Z, Ding K. Discovery of the First Examples of Threonine Tyrosine Kinase PROTAC Degraders. J Med Chem. 2022 Feb 10;65(3):2313-2328.

[3] Cheng B, Ren Y, Cao H, Chen J. Discovery of novel resorcinol diphenyl ether-based PROTAC-like molecules as dual inhibitors and degraders of PD-L1. Eur J Med Chem. 2020;199:112377.

[4] Konstantinidou M, Oun A, Pathak P, Zhang B, Wang Z, Ter Brake F, Dolga AM, Kortholt A, Dömling A. The tale of proteolysis targeting chimeras (PROTACs) for Leucine-Rich Repeat Kinase 2 (LRRK2). ChemMedChem. 2021;16(6):959-965.

[5] Fyles, Thomas M.; McGavin, Cynthia A.; Whitfield, Dennis M. Synthesis of lipophilic 18-crown-6 diacids for the membrane transport of alkaline-earth cations. J Org Chem, 1984; 49: 753 - 761.

[6] Ottis P, Toure M, Cromm PM, Ko E, Gustafson JL, Crews CM. Assessing Different E3 Ligases for Small Molecule Induced Protein Ubiquitination and Degradation. ACS Chem Biol. 2017;12(10):2570-2578.

[7] Current Patent Assignee: ARVINAS; YALE UNIVERSITY - WO2020/51564, 2020, A1

[8] Li W, Gao C, Zhao L, Yuan Z, Chen Y, Jiang Y. Phthalimide conjugations for the degradation of oncogenic PI3K. Eur J Med Chem. 2018 May 10;151:237-247.

[9] Current Patent Assignee: HINOVA PHARMACEUTICALS - EP3957633, 2022, A1

[10] Li Y, Yang J, Aguilar A, McEachern D, Przybranowski S, Liu L, Yang CY, Wang M, Han X, Wang S. Discovery of MD-224 as a First-in-Class, Highly Potent, and Efficacious Proteolysis Targeting Chimera Murine Double Minute 2 Degrader Capable of Achieving Complete and Durable Tumor Regression. J Med Chem. 2019;62(2):448-466.

[11] Han XR, Chen L, Wei Y, Yu W, Chen Y, Zhang C, Jiao B, Shi T, Sun L, Zhang C, Xu Y, Lee MR, Luo Y, Plewe MB, Wang J. Discovery of Selective Small Molecule Degraders of BRAF-V600E. J Med Chem. 2020;63(8):4069-4080.

[12] Current Patent Assignee: CULLGEN SHANGHAI - WO2020/38415, 2020, A1
Location in patent: Page/Page column 108-109

[13] Current Patent Assignee: HALDA THERAPEUTICS OPCO - WO2020/146470, 2020, A1
